# Integrated omics analysis reveals the alteration of gut microbiota and fecal metabolites in *Cervus elaphus kansuensis*

**DOI:** 10.1007/s00253-023-12841-5

**Published:** 2024-01-15

**Authors:** Zhenxiang Zhang, Changhong Bao, Zhaonan Li, Caixia He, Wenjie Jin, Changzhong Li, Yanxia Chen

**Affiliations:** 1 College of Eco-Environmental Engineering, Qinghai University, No. 251 Ningda Road, Xining, 810016 China; 2 Qinghai Provincial Key Laboratory of Adaptive Management on Alpine Grassland, Academy of Animal Science and Veterinary Medicine, Qinghai University, Xining, China

**Keywords:** Gansu red deer, *Cervus elaphus kansuensis*, Gut microbiota, Metabolomics, Environmental adaptation

## Abstract

**Abstract:**

The gut microbiota is the largest and most complex microecosystem in animals. It is influenced by the host’s dietary habits and living environment, and its composition and diversity play irreplaceable roles in animal nutrient metabolism, immunity, and adaptation to the environment. Although the gut microbiota of red deer has been studied, the composition and function of the gut microbiota in Gansu red deer (*Cervus elaphus kansuensis*), an endemic subspecies of red deer in China, has not been reported. In this study, the composition and diversity of the gut microbiome and fecal metabolomics of *C. elaphus kansuensis* were identified and compared for the first time by using 16S rDNA sequencing, metagenomic sequencing, and LC-MS/MS. There were significant differences in gut microbiota structure and diversity between wild and farmed *C. elaphus kansuensis*. The 16S rDNA sequencing results showed that the genus *UCRD-005* was dominant in both captive red deer (CRD) and wild red deer (WRD). Metagenomic sequencing showed similar results to those of 16S rDNA sequencing for gut microbiota in CRD and WRD at the phylum and genus levels. 16S rDNA and metagenomics sequencing data suggested that *Bacteroides* and *Bacillus* might serve as marker genera for CRD and WRD, respectively. Fecal metabolomics results showed that 520 metabolites with significant differences were detected between CRD and WRD and most differential metabolites were involved in lipid metabolism. The results suggested that large differences in gut microbiota composition and fecal metabolites between CRD and WRD, indicating that different dietary habits and living environments over time have led to the development of stable gut microbiome characteristics for CRD and WRD to meet their respective survival and reproduction needs.

**Key points:**

*• Environment and food affected the gut microbiota and fecal metabolites in red deer*

*• Genera Bacteroides and Bacillus may play important roles in CRD and WRD, respectively*

*• Flavonoids and ascorbic acid in fecal metabolites may influence health of red deer*

**Supplementary Information:**

The online version contains supplementary material available at 10.1007/s00253-023-12841-5.

## Introduction

Through long-term natural selection, animals have formed their own unique gut environments, where a large number of microorganisms survive. The gut microbiota has coevolved with the host over the long term, becoming part of the body’s composition and participating in numerous physiological activities such as signal transduction and gut development (Ley et al. 2006a), immune response, energy metabolism, and nutrient absorption (Chai et al. 2022). The microorganisms live in the intestinal tract and assist the host in completing a variety of physiological and biochemical functions, they contribute to the health of the host by maintaining internal stability and improving digestive efficiency. Similarly, there is a huge microbial community in the gastrointestinal tract of ruminants, which can degrade plant fibrous; synthesize vitamins, volatile fatty acids, and microbial proteins needed by the animal body; and provide energy and nutrients needed by the body for growth (Faniyi et al. 2019; Mizrahi et al. 2021). In species biology, the role of ruminant gut microbes is more prominent. Therefore, identification of gut microbiota will provide insight into the annual life cycle of animals, especially those that are vulnerable or endangered (Gao et al. 2021). The ecosystem of the animal gut is dynamic, and numerous factors influence the variety of the gut microbiota (Chen et al. 2021), such as genes (Fan et al. 2021), sex (Fransen et al. 2017), age (Zhang et al. 2018), habitat type (Barelli et al. 2020), climatic circumstances (Fontaine et al. 2018), seasonal changes (Baniel et al. 2021), and diet (Wang et al. 2019a).

The gut microbiota is a complex community with metabolic activity that produces a variety of metabolites that can directly affect host phenotypes. In addition, the detection of fecal metabolites not only reflects changes in metabolites but also provides insight into the gut microbiota (Org et al. 2017). Fecal metabolites are important links between the gut microbiota and host biological functions. The changes in the gut microbiota may alter the distribution of their metabolites, in turn leading to physiological changes in hosts (Cameron and Sperandio 2015).

Gansu red deer (*Cervus elaphus kansuensis*) is an endemic species of *Cervidae* in China (Chen et al. 2022b). Wild red deer (WRD) are protected by the Chinese government (type II). They are mainly distributed in eastern Tibet, western Sichuan, and the Qilian Mountains. *Cervus elaphus kansuensis* is a ruminant whose gut microbiota is critical to its survival. In summer, a wide range of natural plants thrive, providing the WRD with a plentiful food source. However, captive red deer (CRD) are fed mixed diets for a long time and have a relatively stable gut microbiota. The rumen microbiome of red deer in captivity (Qian et al. 2017), the influence of winter enclosures on the gut microbiome of red deer (Sun et al. 2021b), and the gut microbiomes of red deer and fallow deer in captivity (Wang et al. 2019b) have all been studied, whereas differences in gut microbiota and metabolites of the same species *C. elaphus kansuensis* living in different environments have not been characterized. The interaction of gut microbiota and metabolites, as well as their impact on host health and environmental adaptability of *C. elaphus kansuensis*, needs to be studied further.

In this study, the gut microbiota of wild and captive *C. elaphus kansuensis* populations were comparatively analyzed by 16S rDNA sequencing and metagenomics, and the dominant and differential gut microbiota in each population were explored and their associated metabolic pathways were analyzed. A metabolomics approach was then used to detect fecal metabolites in wild and captive *C. elaphus kansuensis* and a combined differential microbiota and metabolite analysis was conducted. The results of this study will contribute to a better understanding of the composition of gut microbiota and its impact on *C. elaphus kansuensis* with diverse food sources and living environments. The comparative study of gut microbiota and fecal metabolites between the two populations will not only assess the health status of both populations but also, more importantly, provide important suggestions for the breeding of CRD and the conservation of wild populations.

## Materials and methods

### Sample collection

All *C. elaphus kansuensis* samples were collected during the summer months (July to August, 2022). A total of 22 fecal samples from a captive population (CRD) were collected from Jiulongjiang Forest Farm in Zhangye City, Gansu Province. Nineteen fecal samples of the wild population (WRD) were collected from the Sidalong Nature Reserve Station in Zhangye City, Gansu Province. All samples were stored in freezers and sent back to the lab, where they were stored at − 80 °C.

### DNA extraction and 16S rDNA sequencing

Fecal DNA was extracted using the PowerFecal^TM^ DNA Isolation Kit (MOBIO Laboratories, Carlsbad, CA, USA) following the manufacturer’s instructions. The DNA concentration was determined using NanoDrop 2000 (Thermo Fisher, Waltham, MA, USA) and adjusted to a concentration of 1 ng/μL. PCR amplification of the 16S rDNA gene V3–V4 variable region in bacterial DNA was performed with primers 341F (5’′-CCTAYGGGRBGCASCAG-3′) and 806R (5′-GGACTACNNGGGTATCTAAT-3′). PCR products were identified using 2% agarose gel electrophoresis and samples were matched according to the concentration of PCR products. A TruSeq® DNA PCR free Sample Preparation Kit (Illumina, San Diego, CA, USA) was used for library construction. NovaSeq6000 (Illumina, San Diego, CA, USA) was used for sequencing after library qualification.

Raw data from Fastq were quality filtered using fastp (Version 0.20.0) software (Chen et al. 2018) and merged using FLASH software (V1.2.11, http://ccb.jhu.edu/software/FLASH/) (Magoc and Salzberg 2011), resulting in high-quality clean tags. Finally, Usearch software (Edgar 2010) was used to compare clean tags with databases to find and delete chimera, resulting in final effective data, specifically “effective tags.”

The program DADA2 (https://github.com/benjjneb/dada2) (Callahan et al. 2016) was used to process effective tags by filtering out low-quality sequences. Sequences with an exact match or 100% sequence similarity were selected as the final amplicon sequence variants (ASVs) and a table of characteristics was produced. Subsequently, the classify-sklearn module within the QIIME2 software (Version 2020.6.0, https://qiime2.org/) (Zhu et al. 2022) package was used to compare the collected ASVs with the Silva database (http://www.arb-silva.de/) (Quast et al. 2013) using a confidence level of 99% to determine the taxonomy of each ASV.

Alpha and beta diversities were analyzed using the software package QIIME2 (Version 2020.6.0). Beta diversity analysis was performed using both principal component analysis (PCA) and principal coordinates analysis (PCoA) based on the weighted/unweighted UniFrac distances and the matrix of distance, respectively. The Adonis algorithm and the anosim algorithm in the program QIIME2 were used to examine the significance of changes in community structure between groups. The linear discriminant analysis (LDA) effect size (LEfSe) technique (LEfSe software, Version 1.0) (Segata et al. 2011) was used to identify taxa with a significant difference between two groups, and a cutoff of log (LDA) scores was set to 4.

### Metagenomic sequencing and bioinformatics analysis

Metagenomics was performed on the same sample used for 16S rDNA sequencing to compare the results. Fecal DNA was obtained in the same way as 16S rDNA sequencing. Quantified libraries were sequenced on the Illumina NovaSeq 6000 (Illumina, San Diego, CA, USA) platform based on the effective concentration of the library and the volume of data needed.

Readfq (V8, https://github.com/cjfields/readfq) and the Bowtie2.2.4 software package (Bowtie2.2.4, http://bowtiebio.sourceforge.net/bowtie2/index.shtml) (Langmead and Salzberg 2012) were used to preprocess raw data from the Illumina NovaSeq 6000 sequencing platform to receive clean data.

High-quality sequences were spliced and assembled using MEGAHIT software (v1.0.4-beta) (Li et al. 2016), and contigs ˃ 500 bp were chosen as the final output of the assembly for further study. Contigs (> 500 bp) were subjected to open reading frame (ORF) prediction using MetaGeneMark software (V2.10, http://topaz.gatech.edu/GeneMark/) (Zhu et al. 2010) with default parameters. Genes ≥ 100 bp were selected for translation into amino acid sequences. CD-HIT software (V4.5.8, http://www.bioinformatics.org/cd-hit) (Fu et al. 2012) was used to cluster gene sequences with the parameters of 90% identification and 90% coverage, with the longest genes in the cluster as their representative sequences, to create a nonredundant set of genes. Reads that optimized for each sample were compared to their nonredundant gene sets using SOAPaligner software (version 2.21, http://soap.genomics.org.cn/) (Li et al. 2008) with parameters of 95% identity, and information on the abundance of genes within each sample was summarized statistically.

Amino acid sequences were aligned to the NR database by Diamond software (V0.9.9, https://github.com/bbuchfink/diamond/) (Buchfink et al. 2015) for species annotation information (BLASTP comparison parameters: e-value is 1e−5). LEfSe (the default LDA score is 3) (Segata et al. 2011) analysis was used to look for the different species between groups.

Functional annotation of microbial functions in the red deer gut was performed by the KEGG database (Version 2018.01, https://www.kegg.jp/), the eggNOG database (Version 4.5, http://eggnog45.embl.de/#/app/home), and the CAZy (Version 2018.01, http://www.cazy.org/) database. First, unigenes were aligned with each functional database using DIAMOND software (blastp, evalue < = 1e−5). Then, the alignment results with the highest score (one HSP (high-scoring segment pair) > 60 bits) were selected for subsequent analysis, and the relative abundance of different functional levels was counted.

### Untargeted liquid chromatography-tandem mass spectrometry (LC-MS/MS) metabolomics

The samples (100 mg) were individually ground with liquid nitrogen and the homogenate was resuspended with 400 μL of extraction solution (acetonitrile: methanol = 1:1) by vortexing well. The samples were incubated in an ice bath for 5 min, and centrifuged at 15,000 g, and 4 °C for 20 min. The supernatant was mixed with 120 μL compound solution (acetonitrile: water = 1:1), and centrifuged at 15,000 g, and 4 °C for 20 min. The supernatant was collected again, and the sample was injected into LC-MS system for analysis (Want et al. 2013).

Untargeted high-performance liquid chromatography–mass spectrometry (LC-MS/MS) analysis was performed at Novogene Co., Ltd. (Beijing, China) utilizing a Vanquish UHPLC system (Thermo Fisher, Waltham, MA, USA) coupled with an Orbitrap Q Exactive^TM^ HF mass spectrometer (Thermo Fisher, Waltham, MA, USA).

Chromatographic conditions: Samples were separated on a Hypesil Gold column (100 mm × 2.1 mm, 1.9 μm, Thermo Fisher, Waltham, MA, USA). The volume of each injection was 10 μL, the column temperature was adjusted to 40 °C, and the flow rate was 0.2 mL/min. Gradient elution was performed with water + 0.1% formic acid (A) and acetonitrile/isopropanol (1/1) (B) as the mobile phases. The gradient elution procedure was as follows: 0–3 min, A decreased from 98 to 0%, and B increased from 2 to 85%; 3–10 min, A was maintained at 0%, B increased from 85 to 100%; 10.0–10.1 min, A increased from 0 to 98%, and B decreased from 100 to 2%; 10.1 to 12 min, A and B were maintained at 98% and 2%, respectively.

Mass spectrometry conditions: The mass spectrometry signal of samples was collected by positive and negative ion scanning modes. The mass scanning range was m/z: 100–1500 Da. The ion spray voltage was 3.5 kV; sheath gas flow rate was 35 arb; aux gas flow rate was 10 arb; capillary temperature was 320 °C; S-lens radio frequency (RF) level was 60; aux gas heater temperature was 350 °C; polarity was positive and negative; and secondary MS/MS scans were data-dependent scans.

The raw data files generated by LC-MS/MS were processed using Compound Discoverer (version 3.1, ThermoFisher, Waltham, MA, USA) to perform peak alignment, peak picking, and quantitation for each metabolite. Then, peaks were matched with the mzCloud (https://www.mzcloud.org/), mzVault (Thermo Fisher, Waltham, MA, USA), and MassList (local database, Novogene Co., Ltd. Beijing, China) databases to obtain accurately qualitative and relatively quantitative results. Statistical analyses were performed using the statistical software R (version R-3.4.3, https://www.r-project.org/), Python (version 2.7.6, https://www.python.org/downloads/release/python-276/), and CentOS (release 6.6, Linux mainframe, RedHat, Raleigh, NC, USA). All identified metabolites were annotated using the KEGG (Release 103.1, https://www.genome.jp/kegg/pathway.html), HMDB (Version 5.0, https://hmdb.ca/metabolites), and LIPIDMaps (http://www.lipidmaps.org/) (O’Donnell et al. 2019) databases. Principal component analysis (PCA) and partial least squares discriminant analysis (PLS-DA) were performed to obtain the variable importance (VIP) of each metabolite. A *T*-test was conducted to calculate the statistical significance (*P*-value). The metabolites with VIP > 1, *P*-value < 0.05, and fold change (FC) ≥ 2 or FC ≤ 0.5 were considered to be differential metabolites (DMs). The screened DMs were imported into MetaboAnalyst 5.0 online software (http://www.metaboanalyst.ca) for clustering and metabolic pathway analysis.

## Results

### Assessment of 16S rDNA sequencing data

In this study, 41 fecal samples were sequenced using 16S rDNA sequencing technology. In the captive red deer group (CRD, 22 samples), 7561 ASVs were identified, averaging 343.68 per sample. In the wild red deer group (WRD, 19 samples), 6463 ASVs were obtained, averaging 340.16 ASVs per sample (Supplemental Table S1). Of these, 3033 ASVs were commonly obtained in both CRD and WRD, and 4528 and 3430 ASVs were specificly obtained in CRD and WRD, respectively (Supplemental Fig. S1).

### Alterations in gut microbiota composition in C. elaphus kansuensis based on 16S rDNA sequencing

**Fig. 1:**
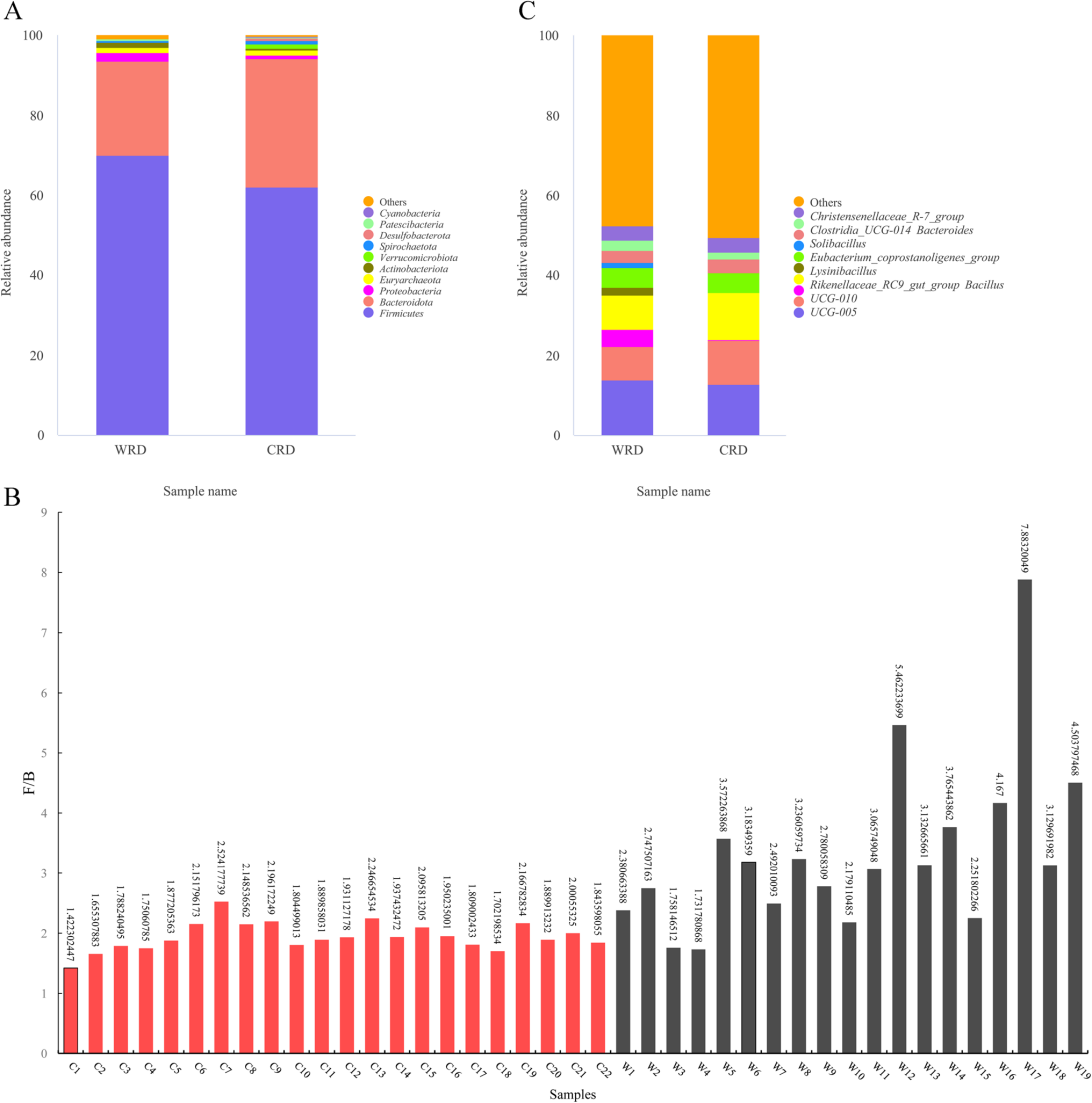
ASVs detected in CRD and WRD based on 16S rDNA sequencing. **A** Bar plot of gut microbiota at phylum level. **B** F/B values of gut microbiota detected by 16S rDNA sequencing. **C** Bar plot of gut microbiota at genus level

Based on the gut microbial species detected at different taxonomic levels, a relative abundance bar chart was developed to illustrate species composition and proportions for each sample at different taxonomic levels. ASVs were classified into 24 and 27 bacterial phyla in the CRD and WRD, respectively (Supplemental Table S2). *Firmicutes* (62.02%; 70.08%) and *Bacteroidetes* (32.17%; 23.53%) were the dominant bacterial phyla in both CRD and WRD, with *Verrucomicrobiota* (1.16%), *Euryarchaeota* (1.10%), and *Proteobacteria* (0.97%) following in CRD and *Proteobacteria* (2.16%), *Euryarchaeota* (1.25%), and *Actinobacteria* (1.22%) in WRD (Fig. 1A, Supplemental Table S3). The *Firmicutes*/*Bacteroidetes* (F/B) ratio is the most commonly used indicator for determining the composition of the gut microbiome (Mariat et al. 2009; Polak et al. 2021). The F/B ratio varied from 1.42 to 2.52 in CRD, with an average of 1.94. In the WRD, the F/B ratio ranged from 1.73 to 7.88, with an average of 3.34 (Fig. 1B).

The genera of these two groups were analyzed. At the genus level, *UCRD-005* (12.77%; 13.88%), *Rikenellaceae_RC9_gut_group* (11.71%; 8.66%), *UCRD-010* (11.04%; 8.47%), *Eubacterium_coprostanoligenes_group* (4.91%; 4.90%), *Christensenellaceae_R-7_group* (3.56%; 3.68%), *Bacteroides* (3.55%; 2.91%), *Alistipes* (3.41%; 2.31%), *Monoglobus* (2.54%; 2.84%), and *Clostridia_UCG-014* (1.60%; 2.54%) were dominant genera in both CRD and WRD. In addition, *Bacteroidales_RF16_group* (2.22%) was dominant in CRD, while *Bacillus* (4.18%) predominated in WRD (Fig. 1C, Supplemental Table S4). The dominant genera listed belong to *Firmicutes* and *Bacteroidota*.

### Alterations in gut microbiota diversity in C. elaphus kansuensis based on 16S rDNA sequencing

The diversity indices for each sample consisted of Chao1, dominance, good’s coverage, observed OTUs (operational taxonomic units), Pielou’s evenness (*E*’), Shannon, and Simpson in a survey of alpha diversity across groups. Among them, the depth index (good’s coverage) in CRD and WRD were 0.9990.0004 and 0.9990.0005, respectively, indicating that an average of 99.90% of microbes were annotated, showing that the data obtained in this study adequately reflect the bacterial species of the samples and that the sequencing findings were enough to reflect the true nature of the samples. The Chao1 index was used to determine the total number of species present in the samples. The Chao1 index in CRD (1500.47 ± 175.65) was higher than that in WRD (1077.14 ± 261.83), indicating that there were more low-abundance species in CRD than in WRD and that the species diversity in CRD was greater than that in WRD (Supplemental Fig. S2A).

The observed OTUs index of CRD (1491.72 ± 172.93) was greater than that of WRD (1073.26 ± 258.72), indicating that more species were observed in CRD than in WRD. There was a significant within-group difference in the observed OTUs index in WRD, with the upper and lower quartiles as well as the median compared to CRD (Supplemental Fig. S2B). The results showed that the number of species annotated in WRD varies from sample to sample and is on average smaller than that in CRD. Based on the Shannon and Simpson indices, the CRD group (9.66010.2221 and 0.99770.0008) exhibited a higher diversity than the WRD group (8.67380.7400 and 0.98990.0114) at *P* < 0.001 and *P* < 0.001, respectively. The results of the Shannon and Simpson indices also showed differences in gut microbial diversity between the CRD and WRD groups (Supplemental Fig. S2C, D).

PCoA analysis using the weighted/unweighted UniFrac distance found that the first principal component contributed 53.27% and 24.25% in the CRD and WRD samples, respectively. The second principal component contributed 11.55% and 11.85%, respectively. There was also significant segregation between the two groups, but the community structure similarity in CRD was relatively higher and both groups were divided into two subgroups. As shown in the figures (Supplemental Fig. S3A, B), all the CRD samples were closely clustered together except one (C18). However, the WRD samples were dispersed. The microbial species composition of the CRD was more similar than that of the WRD.

PCA (Supplemental Fig. S3C) indicated that the first and second principal components contributed 8.15 and 4.42%, respectively. The compositions of the gut microbiota in the CRD and WRD samples were clearly separated from each other, suggesting that the similarity between the two groups was lower than that within each group.

### Differential gut microbiota screened in C. elaphus kansuensis based on 16S rDNA sequencing

**Fig. 2:**
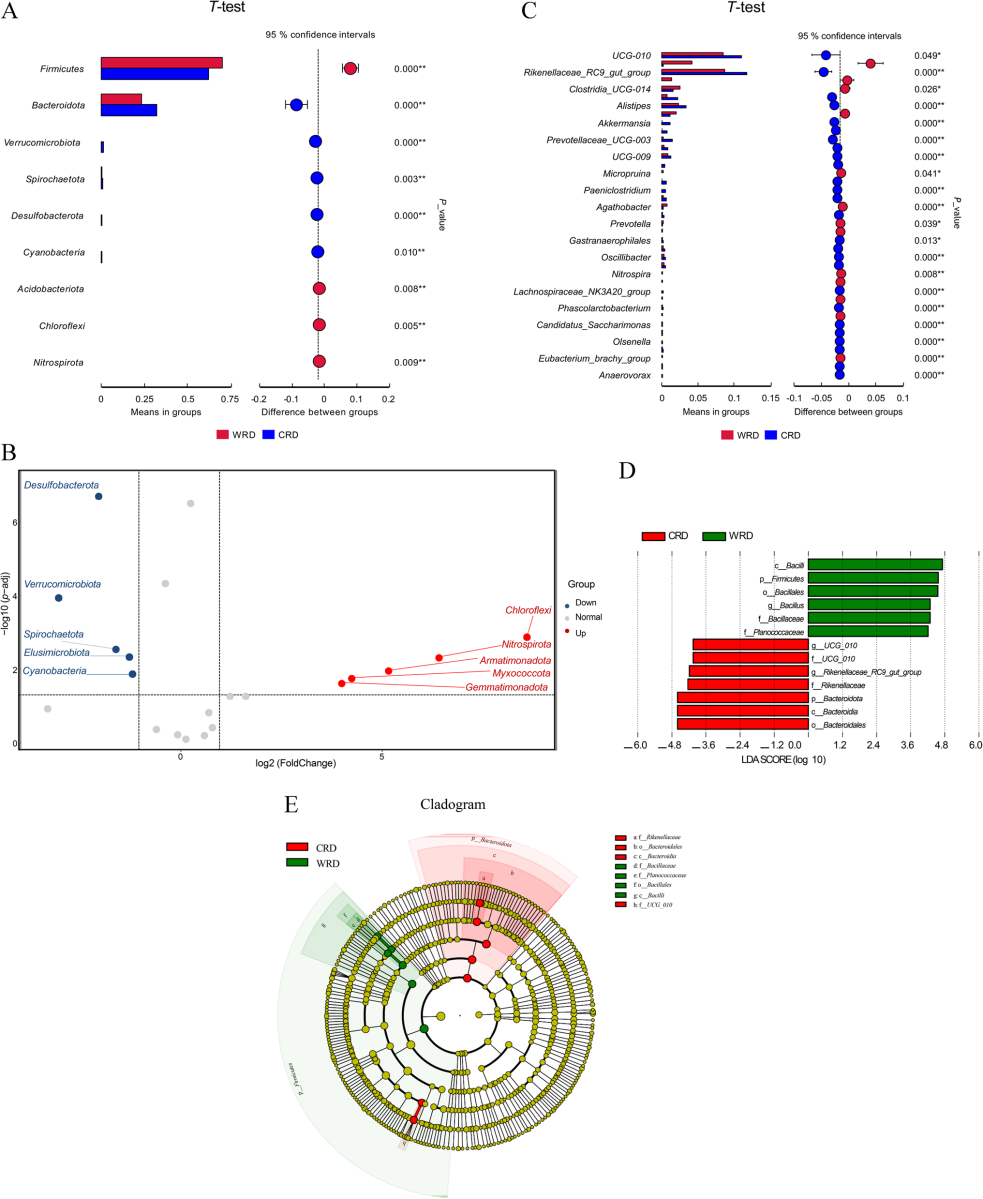
Differential analysis of gut microbiota detected by 16S rDNA sequencing. **A** *T*-test analysis of gut microbiota at phylum level. **B** Volcano plot of differential microbiota. **C** *T*-test analysis of gut microbiota at genus level. **D** LDA score diagram for different species. **E** Evolutionary branching diagram for different species

To identify bacterial differences between the two groups at the phylum and genus levels, we initially identified species with significant differences utilizing the *T*-test (*P* < 0.05). Fourteen phyla showed significant differences between CRD and WRD (*P* < 0.05), including *Firmicutes*, *Bacteroidetes*, *Verrucomicrobiota*, *Spirochaetota*, *Desulfobacterota*, *Cyanobacteria*, *Acidobacteriota*, *Chloroflexi*, and *Nitrospirota* (Fig. 2A, Supplemental Table S5). Among these, *Desulfobacterota*, *Verrucomicrobiota*, *Spirochaetota*, *Elusimicrobiota*, and *Cyanobacteria* were decreased in WRD, whereas *Chloroflexi*, *Nitrospirota*, *Armatimonadota*, *Myxococcota*, and *Gemmatimonadota* were increased in WRD (Fig. 2B).

Furthermore, the *T*-test revealed that 129 genera between CRD and WRD were significantly different (*P* < 0.05) (Fig. 2C), including *UCG-010*, *Rikenellaceae_ RC9_ gut*_ *Group*, *Alistipes*, *Akkermansia*, *Prevollaceae_ UCG-003*, *Treponema*, and *UCG-009*.

Linear discriminant analysis (LDA) effect size (LEfSe) diagrams indicated the key bacterial taxa causing the differences in the two groups (Fig. 2D). There was considerable enrichment of *Bacteroidota* in the CRD and significant enrichment of *Firmicutes* in the WRD. Within the phylum *Bacteroidota*, one genus, *Rikenellaceae_ RC9_ gut_ Group*, was enriched in the CRD group and the genus *Bacillus* was enriched in the WRD group. Within the phylum *Firmicutes*, the genus *UCG-010* was enriched in the CRD group. According to the mapped evolutionary branching diagram, f*_ Rikenellaceae*, o*_ Bacteroidales*, c*_Bacteroidia*, and f*_ UCG_ 010* might play important roles in CRD, while f*_ Bacillaceae*, f*_ Planococcaceae*, o*_ Bacillales*, and c*_ Bailli* might play important roles in WRD (Fig. 2E).

### Metagenomic data analysis

Samples used for 16S rDNA sequencing were also selected for metagenomics sequencing. Based on 16S rDNA sequencing results, six representative samples from 41 samples were selected for subsequent metagenomic sequencing, including C17, C18, and C19 in CRD and W17, W18, and W19 in WRD.

Following metagenomic sequencing, a total of 700,869 complete ORFs were detected in all samples (Supplemental Table S6). The number of genes in common between the two groups was 613,767 as determined by the Venn diagram, and 810,906 and 601,952 unique genes were specifically expressed in WRD and CRD, respectively (Supplemental Fig. S4A). The number of genes in the flower diagram showed that there were 81,534 genes found in all samples, with the total number of genes and unique genes in each sample being C17 (502,941/73,448), C18 (405,168/62,424), C19 (507,213/94,995), W17 (485,651/216,044), W18 (573031/167,070), and W19 (541,816/135,236) (Supplemental Fig. S4B).

### Metagenomic sequencing revealed differences in the species and abundance of gut microbiota between CRD and WRD

The alpha diversity was higher in the CRD population than in the WRD population, as measured by the Shannon and Simpson indices (Table 1). The PCoA based on the Bray-Curtis distance matrix uncovered striking differences in microbial composition between the CRD population and the WRD population at the phylum and genus levels (Supplemental Fig. S5A, B). The PCA results at the genus level demonstrated that the species similarity of the gut microbiota was greater in each sample of the CRD group, while the species differences of the gut microbiota were higher in the three samples of the WRD group, which was similar to the results of 16S rDNA sequencing (Supplemental Fig. S5C).

**Table 1:** Alpha diversity of gut microbiota detected in CRD and WRD based on metagenomic sequencing

	Phylum		Genus	
	WRD	CRD	WRD	CRD
Shannon	1.4554 ± 0.0649	1.7048 ± 0.2799	4.6590 ± 0.1203	4.8874 ± 0.0664
Simpson	0.5461 ± 0.0161	0.5998 ± 0.0665	0.8779 ± 0.0155	0.9088 ± 0.0044

**Fig. 3:**
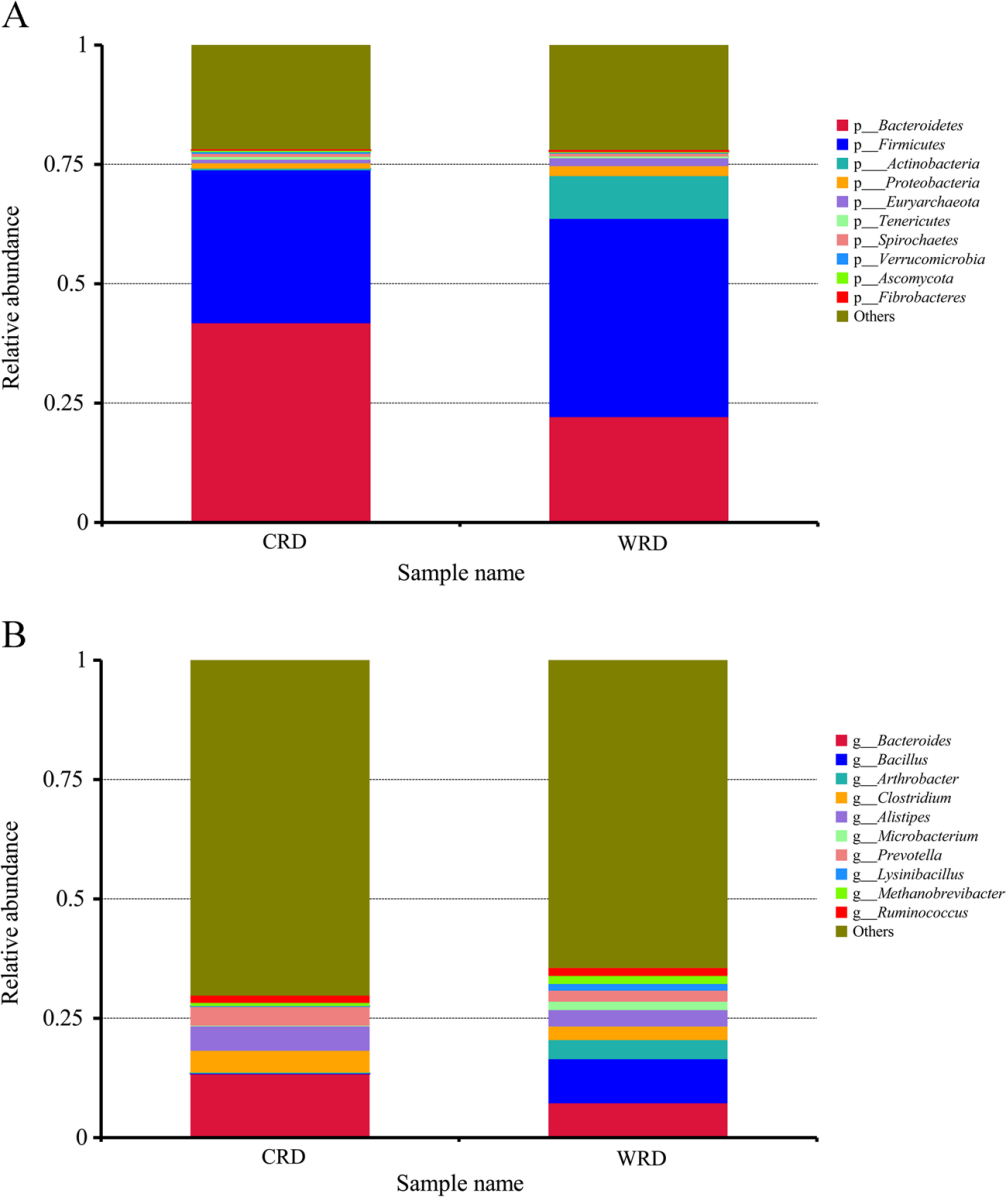
Bar plot of relative species abundance of gut microbiota detected by metagenomic sequencing. **A** Bar plot of gut microbiota at phylum level. **B** Bar plot of gut microbiota at genus level

The top 10 phyla and genera in CRD and WRD were analyzed based on the relative abundance of gut microbiota (Fig. 3A, B). The results showed that *Bacteroidetes* (41.69%) and *Firmicutes* (32.20%) were the most prevalent phyla in CRD, followed by *Proteobacteria* (1.14%), *Euryarchaeota* (0.72%), and *Spirochaetes* (0.69%). Correspondingly, *Firmicutes* (41.51%) and *Bacteroidetes* (22.05%) were the most prevalent phyla in the gut microbiota of WRD, followed by *Actinobacteria* (8.97%), *Proteobacteria* (2.07%), and *Euryarchaeota* (1.80%). These results were similar to those of the 16S rDNA sequencing. At the genus level, *Bacteroides* (13.40%) was the most prevalent genus in CRD, followed by *Alistipes* (5.24%), *Clostridium* (4.58%), and *Prevotella* (4.08%). *Bacillus* (9.22%) was the most abundant genus in WRD, followed by *Bacteroides* (7.16%), *Arthrobacter* (4.01%), and *Alistipes* (3.47%). The results of 16S rDNA sequencing and metagenomic data suggest that *Bacteroides* and *Bacillus* may serve as marker microbiota for CRD and WRD, respectively.

There were 306 genera with significant differences (*P* < 0.05) between CRD and WRD. Among them, *Bacteroides*, *Prevotella*, *Paraprevotella*, and *Parabacteroides* had significantly increased abundance in the CRD group compared with the WRD group (Supplemental Table S7), whereas, *Bacillus*, *Chlamydia*, *Selenomonas*, and *Enterococcus* had significantly decreased abundance in the CRD group compared with the WRD group (Supplemental Table S7).

**Fig. 4:**
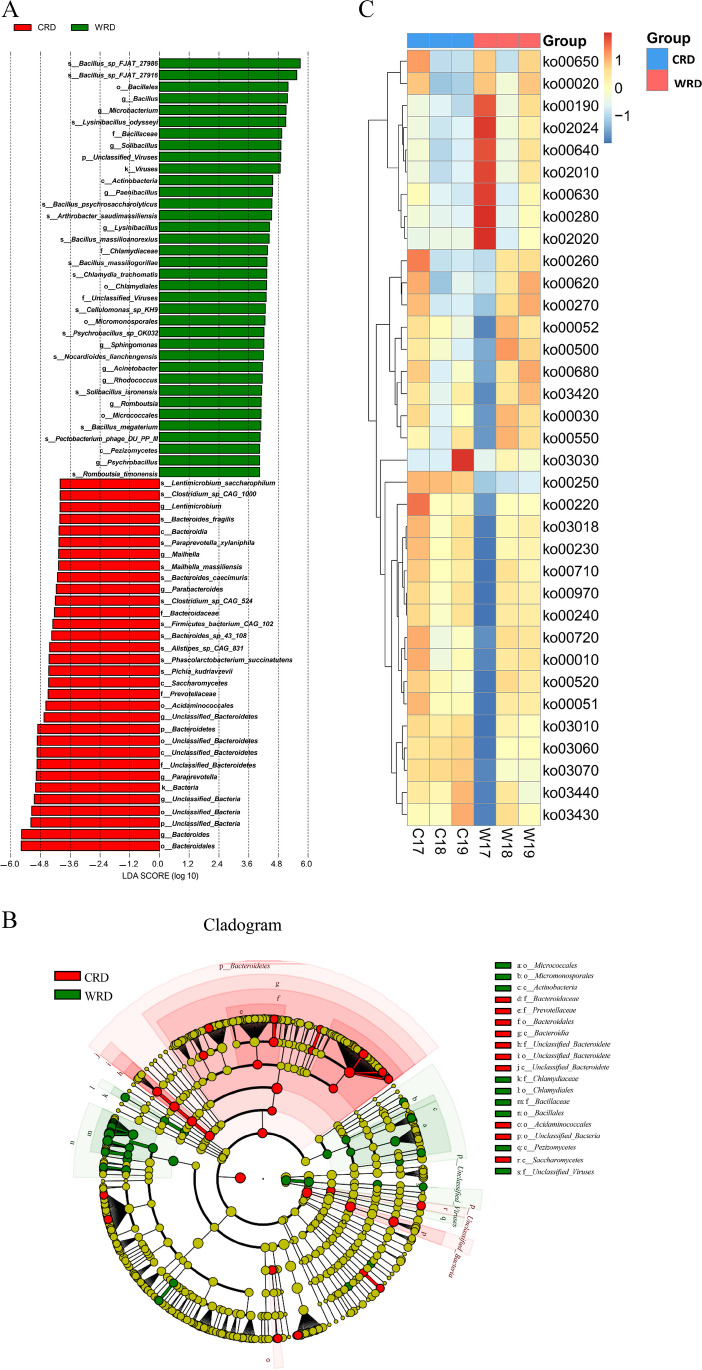
Differential analysis of gut microbiota detected by metagenomic sequencing. **A** LDA score diagram for different species. **B** Evolutionary branching diagram for different species. **C** Heat map of KEGG pathway annotation of gut microbiota detected in CRD and WRD based on metagenomic sequencing

LEfSe analysis showed that o*_ Bacteroidales* and c*_ Bacteroidia* played important roles in the CRD population, while f*_ Bacillaceae* and o*_ Bacillales* played important roles in WRD, which was similar to the results of 16S rDNA sequencing. In addition, other microbial groups also played important roles in CRD and WRD, such as g*_ Bacteroides* in CRD and g*_Bacillus* in WRD (Fig. 4A, B).

### Functional analysis of gut microbiota in C. elaphus kansuensis based on metagenomic sequencing

Functional analysis of the gut microbiota in *C. elaphus kansuensis* was conducted based on the metagenome. Statistics revealed that 729,995 unigenes were annotated to the KEGG pathway database. Of these, “metabolism” annotated the most genes (426,755, 58.46%), followed by “genetic information processing” (121,399, 16.63%), “environmental information processing” (60,651, 8.31%), “cellular processes” (57,428, 7.87%), “human diseases” (42,173, 5.78%), and “organismal systems” (21,589, 2.96%) (Table 2). With respect to functional data, most annotated genes were related to metabolism, such as carbohydrate metabolism, amino acid metabolism, nucleotide metabolism, and energy metabolism. There were also 17,575 genes annotated to “drug resistance: antimicrobial,” which represented the largest number of genes that were associated with human disease.

**Table 2:** KEGG pathway annotation of gut microbiota detected based on metagenomic sequencing

KO pathways on level 1	KO pathways on level 2	Gene numbers	Total gene numbers in KO-pathway level 1
Cellular processes	Cell growth and death	16,664	57,428
Cellular processes	Cell motility	4429	
Cellular processes	Cellular community—eukaryotes	65	
Cellular processes	Cellular community—prokaryotes	29,575	
Cellular processes	Transport and catabolism	6695	
Environmental information processing	Membrane transport	40,327	60,651
Environmental information processing	Signal transduction	20,122	
Environmental information processing	Signaling molecules and interaction	202	
Genetic information processing	Folding, sorting, and degradation	24,258	121,399
Genetic information processing	Replication and repair	41,522	
Genetic information processing	Transcription	5659	
Genetic information processing	Translation	49,960	
Human diseases	Cancers: overview	5595	42,173
Human diseases	Cancers: specific types	1358	
Human diseases	Cardiovascular diseases	2374	
Human diseases	Drug resistance: antimicrobial	13,789	
Human diseases	Drug resistance: antineoplastic	3786	
Human diseases	Endocrine and metabolic diseases	4451	
Human diseases	Immune diseases	614	
Human diseases	Infectious diseases: bacterial	6916	
Human diseases	Infectious diseases: parasitic	349	
Human diseases	Infectious diseases: viral	1110	
Human diseases	Neurodegenerative diseases	1787	
Human diseases	Substance dependence	44	
Metabolism	Amino acid metabolism	73,154	426,755
Metabolism	Biosynthesis of other secondary metabolites	16,638	
Metabolism	Carbohydrate metabolism	92,466	
Metabolism	Energy metabolism	47,676	
Metabolism	Glycan biosynthesis and metabolism	27,316	
Metabolism	Lipid metabolism	23,532	
Metabolism	Metabolism of cofactors and vitamins	46,517	
Metabolism	Metabolism of other amino acids	21,079	
Metabolism	Metabolism of terpenoids and polyketides	13,322	
Metabolism	Nucleotide metabolism	54,866	
Metabolism	Xenobiotics biodegradation and metabolism	10,189	
Organismal systems	Aging	4804	21,589
Organismal systems	Circulatory system	143	
Organismal systems	Development	20	
Organismal systems	Digestive system	1229	
Organismal systems	Endocrine system	8283	
Organismal systems	Environmental adaptation	1796	
Organismal systems	Excretory system	728	
Organismal systems	Immune system	2164	
Organismal systems	Nervous system	2411	
Organismal systems	Sensory system	11	
Total number of annotated genes		729,995	729,995

Based on abundance information, the KEGG orthologs with the highest relative abundance in CRD included K03088 (RNA polymerase sigma-70 factor, ECF subfamily), K02337 (DNA polymerase III subunit alpha), K21572 (starch-binding outer membrane protein, SusD/RagB family), K07133 (uncharacterized protein), K03497 (chromosome partitioning protein, ParB family), K01915 (glutamine synthetase), K01153 (type I restriction enzyme, R subunit), and K01955 (carbamoyl-phosphate synthase large subunit). In the WRD group, KEGG orthologs with high relative abundance in gut microbiota included K06147 (ATP-binding cassette, subfamily B, bacterial), K01992 (ABC-2 type transport system permease protein), K01990 (ABC-2 type transport system ATP-binding protein), and K03466 (DNA segregation ATPase FtsK/SpoIIIE, S-DNA-T family). The gut microbiota of CRD was mainly involved in protein metabolism, while the gut microbiota of WRD was mainly involved in energy metabolism (Fig. 4C).

### Fecal metabolites detected in C. elaphus kansuensis based on LC-MS/MS 

The LC-MS/MS raw data were processed, and 989 metabolites were identified in all samples. Of these, 570 and 419 metabolites were generated using positive and negative ion modes, respectively (Supplemental Table S8). All these metabolites were present in six samples from CRD and six from WRD.

**Fig. 5:**
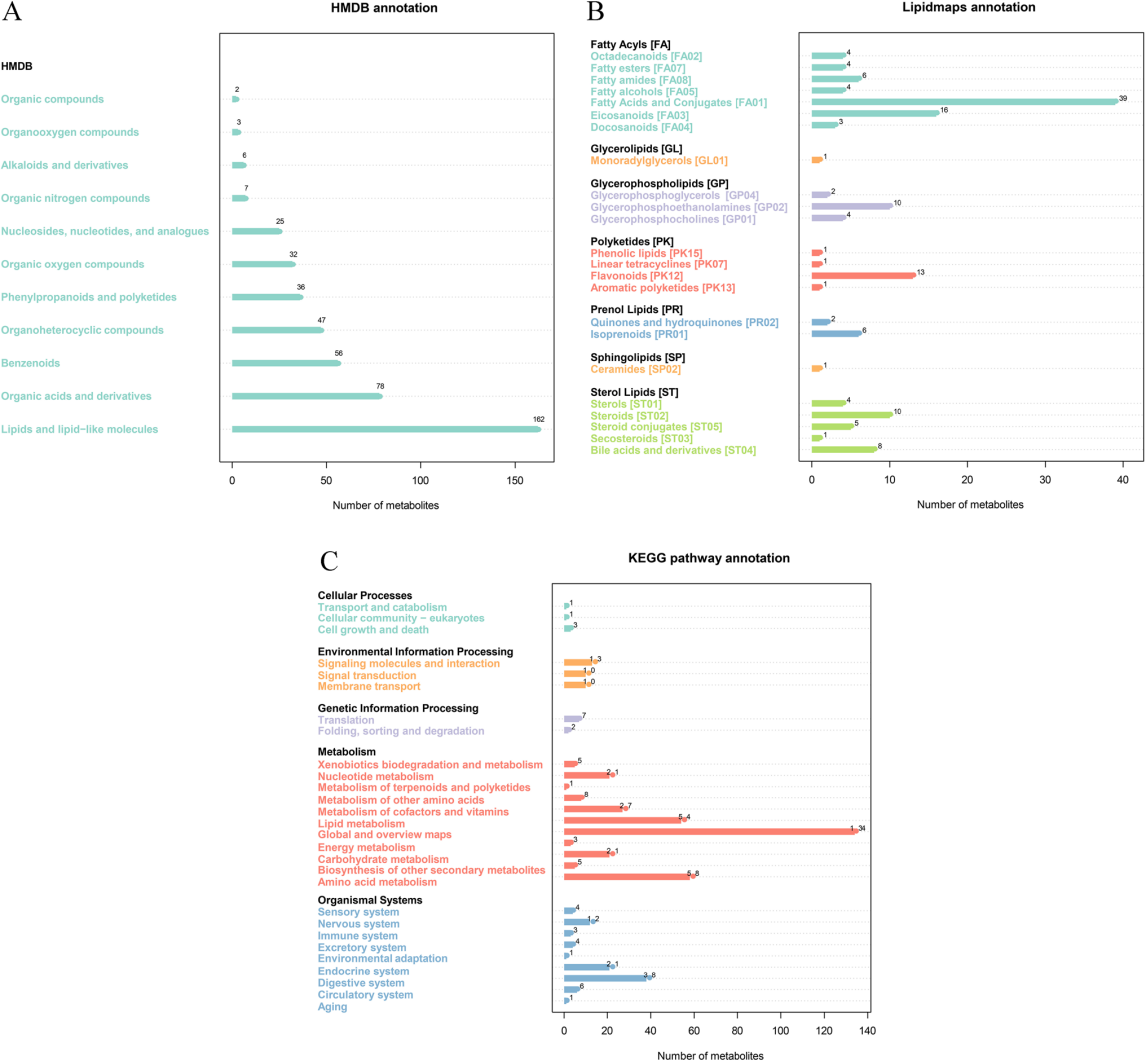
Annotation of all fecal metabolites detected in CRD and WRD. **A** P HMDB annotation of all metabolites. **B** Lipidmaps annotation of all metabolites. **C** KEGG annotation of all metabolites

The results of HMDB annotation showed that “lipids and functional-like molecules” annotated the most metabolites (162 metabolites), followed by “organic acids and derivatives” (78 metabolites), “benzenoids” (56 metabolites), and “organoheterocyclic compounds” (47 metabolites). “Organic compounds” annotated the fewest metabolites (2 metabolites) (Fig. 5A). Based on the results of Lipidmaps annotation, “fatty acyls” [FA] annotated the most metabolites (36 metabolites), followed by “sterol lipids” [ST] (28 metabolites), “glycerophospholipids” [GP] (16 metabolites), and “polyketides” [PK] (16 metabolites). “Glycerolipids” [GL] and “sphingolipids” [SP] annotated the fewest metabolites, with only 1 (Fig. 5B). In the KEGG metabolic pathway categories, the “metabolism” type annotated the highest number of metabolites (337 metabolites), which included “amino acid metabolism” (58 metabolites), “lipid metabolism” (54 metabolites), “digestive system” (38 metabolites), “metabolism of cofactors and vitamins” (27 metabolites), “carbohydrate metabolism” (21 metabolites), and “nucleotide metabolism” (21 metabolites) (Fig. 5C).

### Differential analysis and functional annotation of fecal metabolites in C. elaphus kansuensis

The overall distribution of the two sets of samples was observed using the PCA approach (Supplemental Fig. S6A). The results showed that the fecal samples of the CRD group had good cluster, while the fecal samples of the WRD group were relatively scattered, indicating that the metabolite differences among samples in CRD were smaller, while the metabolite differences among samples in WRD were larger. Plots of PCA scores revealed significant differences between the CRD and WRD groups, indicating that fecal metabolites in *C. elaphus kansuensis* were altered in a remarkable manner.

**Fig. 6:**
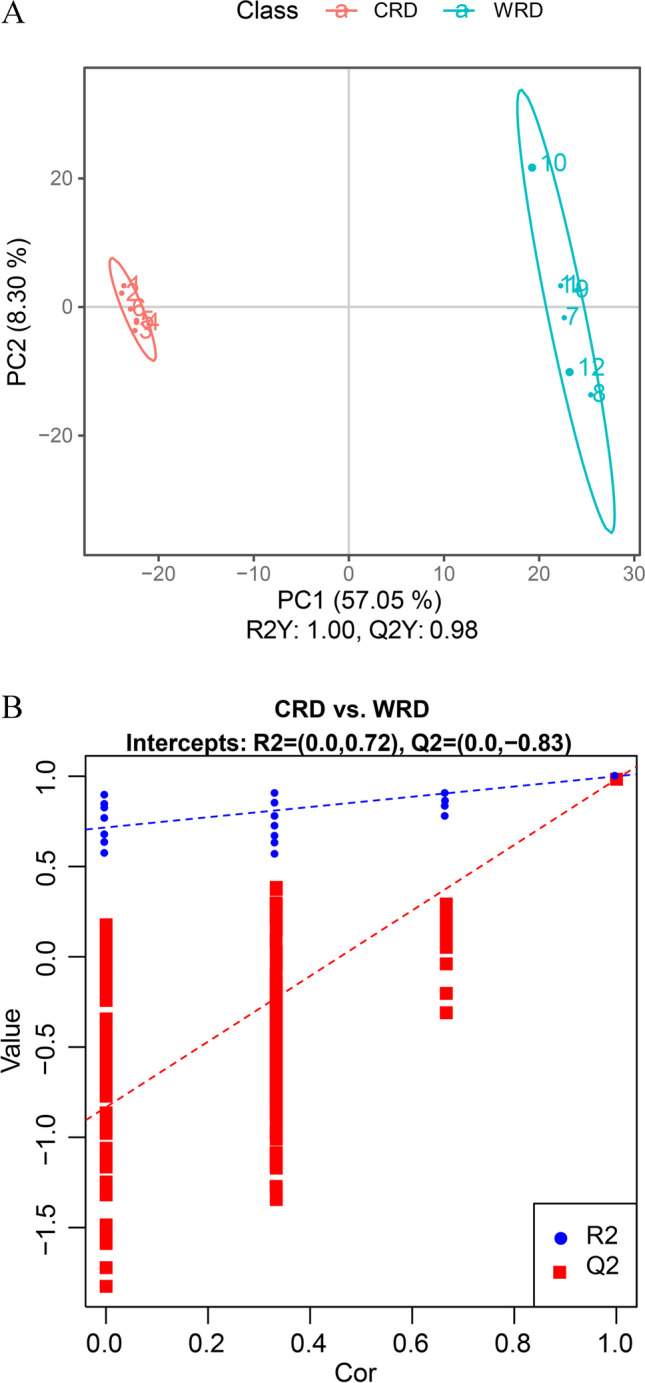
Differential analysis of fecal metabolites detected in CRD and WRD based on LC-MS/MS. **A** PLSDA score scatter plot of differential metabolites. **B** PLSDA validation diagram of differential metabolites

Differential metabolites were then identified using the supervised PLS-DA model with VIP > 1.0, FC > 2 or FC < 0.5, and *P* values < 0.05 (*T*-test) (Fig. 6A, B). The results revealed that 520 metabolites with significant differences were detected in the CRD vs. WRD, of which 316 metabolites were increased and 204 metabolites were decreased (Supplemental Table S9, Supplemental Fig. S6B) in the Hierarchical clustering analysis (HCA) of significantly different metabolites was performed to display the expression patterns of metabolites detected in samples within the CRD and WRD groups (Supplemental Fig. S6C). Specifically, compared with the WRD group, the levels of 2-[2-(2-pyridyloxy)ethoxy]pyridine, theophylline, harmine, (+/−)-CP 47,497-C7-hydroxy metabolite, 13,14-dihydro-15-keto prostaglandin A2, *O*-desmethylnaproxen, L-tyrosinemethylester, D-δ-tocopherol, fatty acid esters of hydroxy fatty acid (FAHFA) (2:0/23:0), and gamma-glutamylmethionine were significantly increased in CRD (*P <* 0.05), while the levels of morphine, (+/−)5(6)- epoxyeicosatrienoic acid (EET) ethanolamide, 18-β-glycyrrhetinic acid, normorphine, prostaglandin A3, human kallikrein (HKK), 2-hydroxy-6-[(8*Z*,11*Z*)-pentadeca-8,11,14-trien-1-yl]benzoic acid, 1-acetyl-3-(2-furylmethylene)tetrahydropyrazine-2,5-dione, norbutorphanol, and 5α-dihydrotestosterone were significantly decreased in the CRD group (*P* < 0.05).

**Fig. 7:**
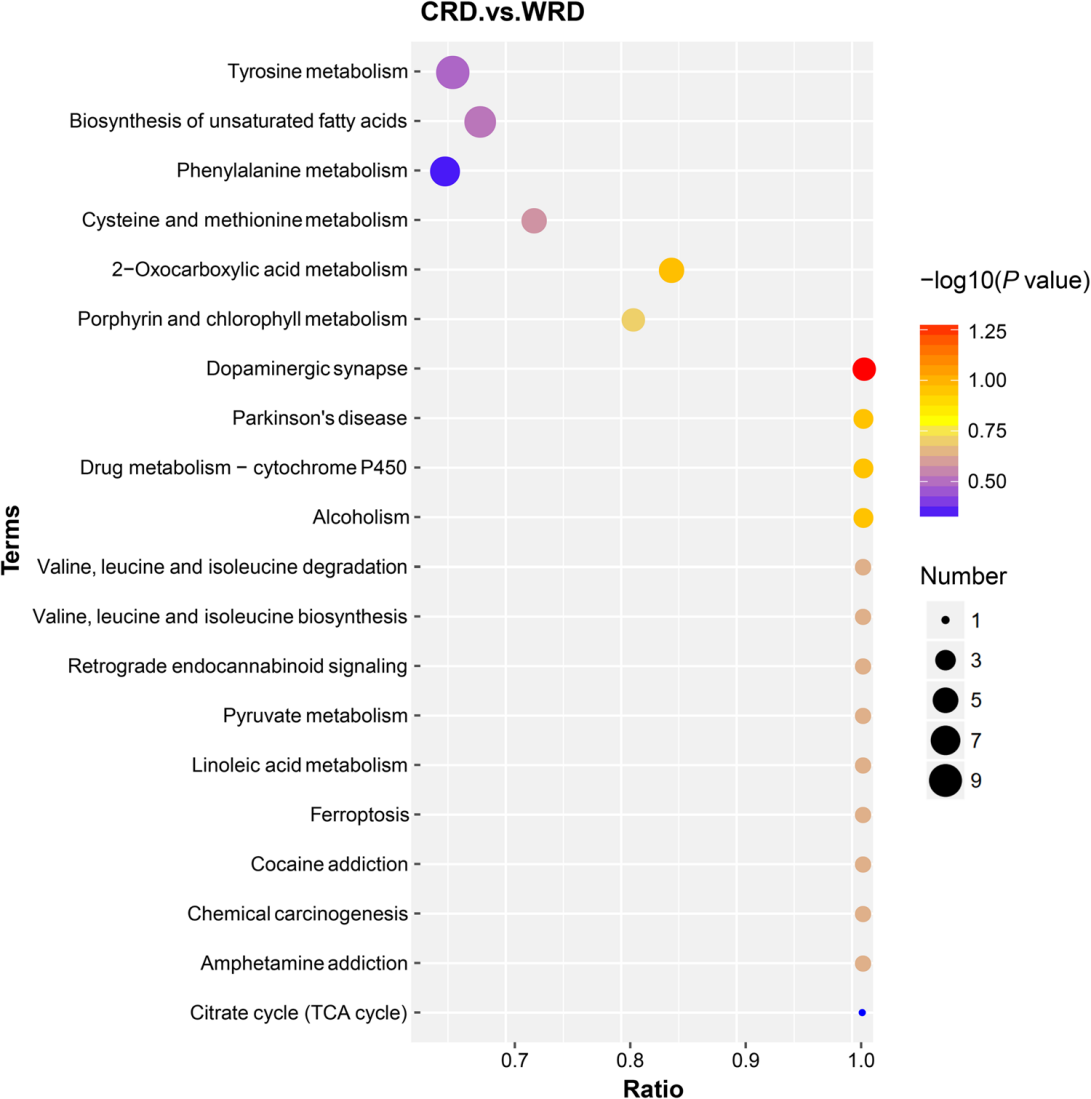
Bubble map of the enriched KEGG pathways of differential metabolites

In the HMDB annotation, most differential metabolites (DMs) were annotated to “lipids and lipid-like molecules” (82 DMs), followed by “organic acids and derivatives” (37 DMs), “benzenoids” (26 DMs), “organoheterocyclic compounds” (25 DMs), and “phenylpropanoids and polyketides” (25 DMs) (Supplemental Fig. S7A). In Lipidmaps annotation, most of the differential metabolites were annotated to “fatty acyls [FA]” (36 DMs), followed by “polyketides [PK]” (15 DMs) and “sterol lipids [ST]” (14 DMs) (Supplemental Fig. S7B). The functions of these altered metabolites were then identified by using KEGG pathway analyses. The results indicated that all the differential metabolites were enriched into 64 metabolic pathways. Of these, “tyrosine metabolism” (9 DMs), “biosynthesis of amino acids” (9 DMs), “steroid hormone biosynthesis” (9 DMs), “biosynthesis of unsaturated fatty acids” (8 DMs), and “phenylalanine metabolism” (7 DMs) were annotated the most differential metabolites (Fig. 7).

HMDB, Lipidmaps, and KEGG annotation showed that most differential metabolites were involved in lipid metabolism. We were concerned with the differential metabolites annotated in “lipids and lipid-like molecules” (HMDB), “fatty acyls [FA]” (Lipidmaps), and “biosynthesis of unsaturated fatty acids” (KEGG), including adrenic acid, arachidic acid, arachidonic acid, docosahexaenoic acid, lignoceric acid, nervonic acid, and palmitic acid. Furthermore, we also focused on the “dopaminergic synapse pathway,” which significantly annotated (*P* < 0.05) and may be related to the health, environmental adaptation, and estrus of *C. elaphus kansuensis*. There were three differential metabolites annotated to this pathway, including homovanillic acid, L-Dopa (levodopa), and 3-methoxytyramine.

### Combined analysis of gut microbiota with fecal metabolites

**Fig. 8:**
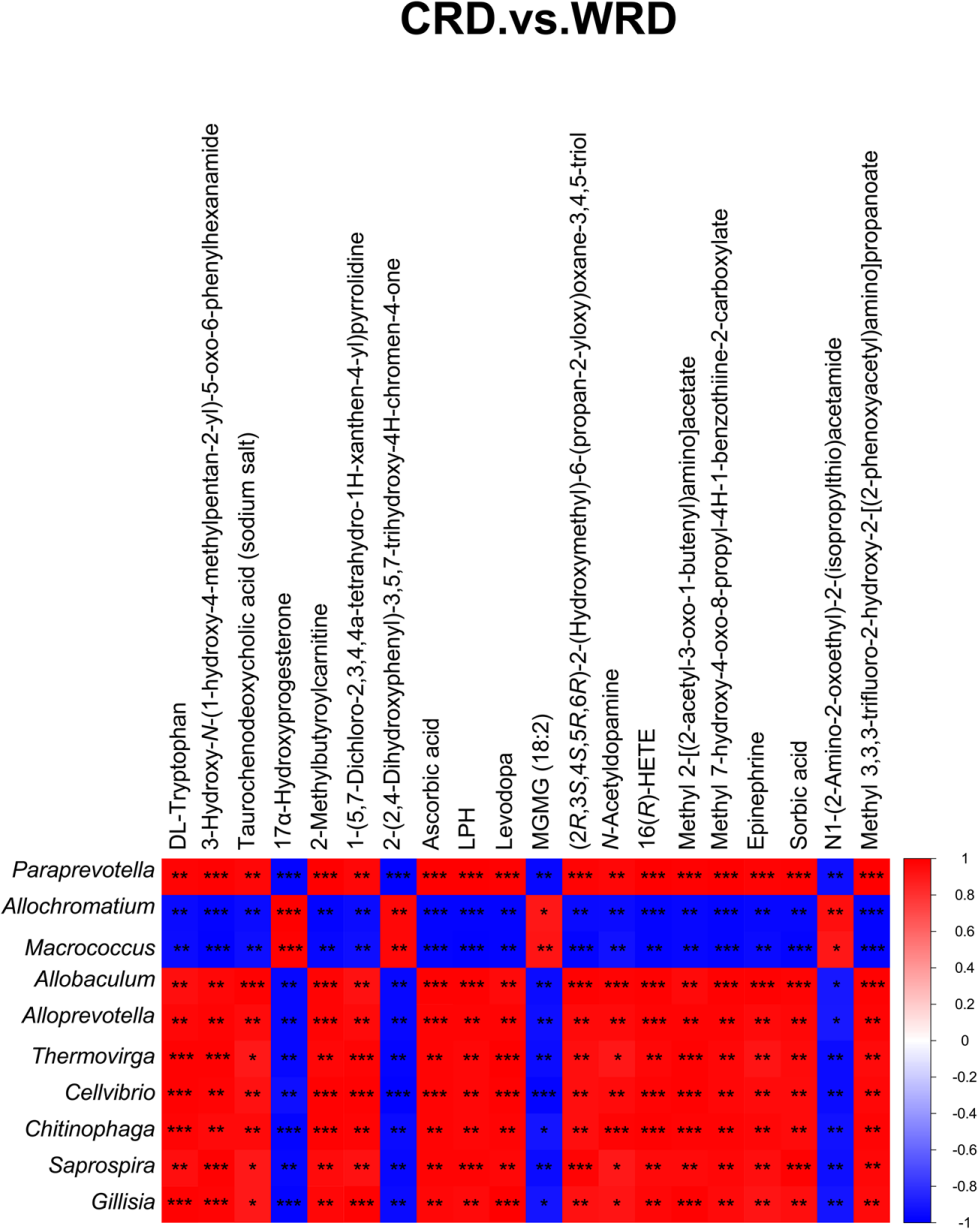
Correlation analysis of the gut microbiome and fecal metabolites. Correlation analysis of significantly different metabolites with significantly different gut microbiota at genus level. The results of were presented as a heatmap. *P < 0.05, **P < 0.01, ***P < 0.001 denoted statistical significance between bacterial taxa and metabolites

To further explore the relationship between the gut microbiota and fecal metabolites, Spearman’s correlation analysis was conducted on the differential host gut microbiota at the genus level and differential fecal metabolites identified between captive and wild red deer (Fig. 8). Differential metabolites were ranked from small to large according to the *P* value, and the top 20 metabolites were selected for correlation analysis. Similarly, the different genera were ranked from small to large according to the *P* value, and the top 10 genera were selected for correlation analysis. The results showed that the differential gut microbiota of *Paraprevotella*, *Allobaculum*, *Alloprevotella*, *Thermovirga*, *Cellvibrio*, *Chitinophaga*, *Saprospiraa*, and *Gillisia* showed significant positive correlation with DL-tryptophan, 3-hydroxy-*N*-(1-hydroxy-4-methylpentan-2-yl)-5-oxo-6-phenylhexanamide, taurochenodeoxycholic acid (sodium salt), 2-methylbutyroylcarnitine, 1-(5,7-dichloro-2,3,4,4a-tetrahydro-1H-xanthen-4-yl)pyrrolidine, ascorbic acid, lactase phlorizin hydrolase (LPH), aevodopa, (2*R*,3*S*,4*S*,5*R*,6*R*)-2-(hydroxymethyl)-6-(propan-2-yloxy)oxane-3,4,5-triol, *N*-acetyldopamine, 16(*R*)-hydroxyeicosatetraenoic acid (16(*R*)-HETE), methyl 2-[(2-acetyl-3-oxo-1-butenyl)amino]acetate, methyl 7-hydroxy-4-oxo-8-propyl-4H-1-benzothiine-2-carboxylate, epinephrine, sorbic acid, and methyl 3,3,3-trifluoro-2-hydroxy-2-[(2-phenoxyacetyl)amino]propanoate (*P* < 0.05), while those differential gut microbiota were significantly negatively correlated with 17α-hydroxyprogesterone,2-(2,4-dihydroxyphenyl)-3,5,7-trihydroxy-4H-chromen-4-one, monogalactosylmonoacylglycerol (MGMG) (18:2), and *N*1-(2-amino-2-oxoethyl)-2-(isopropylthio)acetamide. In contrast, *Allochromatium* and *Macrococcus* were significantly negatively correlated with DL-tryptophan, 3-hydroxy-*N*-(1-hydroxy-4-methylpentan-2-yl)-5-oxo-6-phenylhexanamide, taurochenodeoxycholic acid (sodium salt), 2-methylbutyroylcarnitine, 1-(5,7-dichloro-2,3,4,4a-tetrahydro-1H-xanthen-4-yl)pyrrolidine, ascorbic acid, LPH, levodopa, (2*R*,3*S*,4S,5*R*,6*R*)-2-(hydroxymethyl)-6-(propan-2-yloxy)oxane-3,4,5-triol, *N*-acetyldopamine, 16(*R*)-HETE, methyl 2-[(2-acetyl-3-oxo-1-butenyl)amino]acetate, methyl 7-hydroxy-4-oxo-8-propyl-4H-1-benzothiine-2-carboxylate, methyl 7-hydroxy-4-oxo-8-propyl-4H-1-benzothiine-2-carboxylate, epinephrine, sorbic acid, and methyl 3,3,3-trifluoro-2-hydroxy-2-[(2-phenoxyacetyl)amino]propanoate (*P* < 0.05), and significantly positively correlated with 17α-hydroxyprogesterone, 2-(2,4-dihydroxyphenyl)-3,5,7-trihydroxy-4H-chromen-4-one, MGMG (18:2), and *N*1-(2-amino-2-oxoethyl)-2-(isopropylthio)acetamide.

## Discussion

The gut microbiota is the result of long-term coevolution between microbes and their hosts. It affects the physiological function of the host and is influenced by the living environment and feeding habits of the host animal (Spor et al. 2011). High-throughput sequencing techniques are commonly used to analyze gut microbiota structure and diversity in a variety of endangered species (Antwis et al. 2019; Guo et al. 2020; Huang et al. 2022; Wang et al. 2022c). Compared to other types of samples, feces are easy to collect and does not harm endangered animals. In addition, feces can demonstrate the composition and activity of the hosts’ gut microbiota (Rounge et al. 2018). This non-injurious sampling method is widely used in wildlife conservation biology research, avoiding the impact of the traditionally injurious sampling methods, and is well suited for studies of endangered animals (Knutie and Gotanda 2018; Ning et al. 2020).

### Alterations in the gut microbiota composition of C. elaphus kansuensis based on 16S rDNA sequencing

Alpha diversity, a quantitative measure of the diversity, stability, and composition of the host gut microbiota, is a critical predictor of host health status (Haifer et al. 2022; Wei et al. 2022). High alpha diversity suggests a diverse and stable gut microbiota that is less impacted by dietary fluctuation and more resilient to perturbations in the environment. Thus, higher alpha diversity supports host fitness because hosts can adapt and manage their homeostasis (Lang et al. 2018). Alpha diversity is closely related to dietary differences, and if the diversity of diet is increased, more metabolic ecological niches can be created for microbial communities, resulting in increased diversity of microbial communities (Schluter and Foster 2012). In this study, CRD had greater alpha diversity than WRD in the same season. This may be because the food source of captive red deer is richer in summer, including straw feed, mowed green feed, and concentrate feed, and the food ecological niche of CRD is wide, resulting in a higher diversity of gut microbiota.

The relative abundances of bacteria in this study differed between CRD and WRD. *Bacteroidetes* and *Firmicutes* were the two most prevalent phyla of gut microbiota in *C. elaphus kansuensis*, and both of these phyla were also the dominant microbes in the gut microbiota of numerous mammalian and herbivorous species (Chen et al. 2017; Guan et al. 2017; Jiang et al. 2022). The *Firmicutes*/*Bacteroidetes* (F/B) ratio has been previously proposed to be marker of metabolic disease (Magne et al. 2020). There is a strong relationship between obesity and the F/B abundance ratio (Stojanov et al. 2020). A high F/B ratio will lead to obesity in humans, pigs, mice, and other animals (Guo et al. 2008; Indiani et al. 2018). Gut microbes with a high F/B ratio have a higher fermentation efficiency and capture more energy from food, thus promoting fat deposition (Ley et al. 2006a). In the present study, the F/B ratio was higher in WRD than in CRD. The Qilian Mountains have abundant forage growth in summer, in which the carbohydrate content reaches its peak, providing a sufficient energy source for WRD (Purdy et al. 2014). The increased F/B ratio in the gut microbiota of wild red deer enables them to consume as much energy from food as possible to sustain their body’s demands, offset the energy consumption of foraging and escaping predators, and store energy for future generations.

*Bacteroidetes*, one of the dominant phyla in the mammalian gut, plays a role in breaking down macromolecules in the gut and is mainly responsible for degrading polysaccharides and dietary fibers (Diamant et al. 2011). Studies have shown that *Bacteroidetes* and *Firmicutes* are associated with body fat, and there is a negative correlation between *Bacteroidetes* abundance and body fat (Guo et al. 2008). Human individuals who are overweight have an increased abundance of *Firmicutes* and decreased abundance of *Bacteroidetes* in their gut (Ley et al. 2006b). The relative abundances of *Bacteroidetes* in the CRD and WRD were 32.17% and 23.53%, respectively. The food of CRD mainly consisted of straw, various grains, and soybean meal. CRD has more protein and fiber in its diet than WRD, which can promote growth and reduce fat deposition in CRD.

At the genus level, *Bacillus* was the genus with the most significant difference, with relative abundances of 0.16% and 4.18% in the CRD and WRD, respectively. *Bacillus* is more resistant to various substances, such as heat, UV light, and other environmental stresses (Ryu and Beuchat 2005). It is widely distributed in nature, and its habitats are soil, plant surfaces, food, and water (Adeniji et al. 2019). *Bacillus subtilis* is a common probiotic that can be directly fed to animals (Kovacs 2019). It has prebiotic functions such as regulating the gastrointestinal microbes of animals, promoting the digestion and absorption of nutrients, and enhancing animal immunity (Wang et al. 2022a). The antibiotic subtilin is produced by *B. subtilis*. It can inhibit fungal growth and be used as a natural food preservative (Geiger et al. 2019). *B. subtilis* powder can be used as a feed additive for animals (Feedap et al. 2022), which can promote their growth and development (Xu et al. 2021). *Bacillus coagulans* is another species of the genus *Bacillus*. It can be used as a probiotic in animal feed and has broad application prospects in animal husbandry (Feedap et al. 2022). Recent scientific research and production practices have shown that *B. coagulans* can inhibit harmful gut bacteria and regulate gut microbiota in livestock and poultry (Lee et al. 2019; Zhou et al. 2020). The powder of *B. coagulans* has an obvious regulatory function on the gut tract and can be used as a biological health product and functional food additive (Konuray and Erginkaya 2018). *Bacillus natto* was found to be a key bacterium used for natto production, a traditional Japanese fermented food, and has the effect of lowering blood pressure, antitumor activity, thrombolytic activity, etc. (Ruiz Sella et al. 2021). A higher proportion of *Bacillus* was found here in WRD compared with CRD, which may play a role in promoting the development of the gastrointestinal tract, enhancing the rumen function and growth performance of WRD, and resisting invasion of harmful microorganisms from nature.

### Metagenomics sequencing revealed functional differences in the gut microbiota in C. elaphus kansuensis

Metagenomics is genome sequencing based on whole genome datasets. Large-scale sequence analysis and bioinformatics allow access to microbial species annotation and functional annotation information, which is more advantageous in terms of sequencing depth than 16S rDNA sequencing (Zhernakova et al. 2016). The functional differences in the gut microbiota of CRD and WRD were analyzed by metagenome sequencing to provide a comprehensive understanding of the impact of environmental, food, and other factors on the gut microbiota of *C. elaphus kansuensis*.

Comparing the results of both methods, the annotated species at each taxon level were relatively similar, but the relative abundance of gut microbiota differed somewhat. In the metagenomic data, *Firmicutes* and *Bacteroidetes* were also found to be the two phyla with the highest abundance in *C. elaphus kansuensis*. At the genus level, *Bacteroides* was the dominant bacteria in CRD, while *Bacillus* was dominant in WRD, which was consistent with the results of 16S rDNA sequencing.

For the CRD group, *Proteobacteria* was the most abundant phyla in CRD except for *Bacteroidetes* and *Firmicutes*. Studies have shown that *Proteobacteria* has many physiological functions (Samanta et al. 2012; Sun et al. 2021a). *Proteobacteria* with high abundance in the gut microbiota of CRD may play an important role in maintaining the homeostasis of gut microbiota and the healthy growth and development of CRD. The highest relative abundance genera in the CRD group were *Bacteroides* and *Alistipes*, which are genera in the phylum *Bacteroidetes*. Bacteria with high abundance in *Bacteroides* and *Alistipes* may also promote CRD growth while reducing fat deposition.

For the WRD group, *Firmicutes*, *Bacteroidetes*, *Actinobacteria*, and *Proteobacteria* were the most abundant phyla in WRD. These four primary phyla comprise the most human microorganisms. More than 90% of the gut microbiota is made up of *Firmicutes* and *Bacteroidetes*, and their interaction is crucial for maintaining gut homeostasis. The remaining 10% is made up of *Actinobacteria* and *Proteobacteria* (Arumugam et al. 2011; Segata et al. 2012).

*Firmicutes* was very abundant in WRD. Studies have shown that *Firmicutes* could produce available energy, which may be associated with the development of diabetes and obesity (Komaroff 2017). The greater prevalence of *Firmicutes* in WRD may play a role in energy metabolism and affect certain physiological functions of wild red deer. *Actinomycetes* is one of the four major phyla of gut microbiota and play a crucial role in maintaining gut homeostasis, although they only possess a small percentage. According to Belizário and Napolitano (2015), *Actinobacteria* are group of Gram-positive bacteria, comprising the anaerobic genera *Bifidobacteria*, *Propionibacteria*, and *Corynebacteria* as well as the aerobic genus *Streptomyces. Actinobacteria* are one of the most common symbiotic bacteria in the small and large human intestines throughout adulthood and maintain a stable percentage of approximately 8% (D’Argenio and Salvatore 2015). This phylum of bacteria, especially *Bifidobacteria*, is widely used as a probiotic and has shown beneficial effects in many pathologic conditions (Binda et al. 2018). The genus *Bifidobacteria* is essential for maintaining the homeostasis of the gut barrier (Sivan et al. 2015; Tang et al. 2022). In addition, *Actinomycetes* can create numerous bioactive metabolites and derived antibiotics, and they are widely distributed in both aquatic and terrestrial habitats (Servin et al. 2008; Tenaillon et al. 2010). The high prevalence of *Actinomycetes* may have a significant impact on WRD health.

The functional analysis of gut microbiota showed that the gut microbiota in CRD were mainly related to “gene expression and regulation,” whereas genes in WRD were primarily related to “energy metabolism.” In summer, WRD are confronted with richness in food sources and eat many grasses and herbs, which have a higher content of carbohydrates (Purdy et al. 2014). Captive deer, on the other hand, are mostly fed straw. Therefore, the gut microbiota genes of WRD annotated to energy metabolism related pathways may be associated with the high consumption of grasses and herbs.

### Metabolomic analysis revealed metabolic alterations in C. elaphus kansuensis

The gut microbiota is the largest biometabolic library of human and animal organisms. It can not only decompose large molecular substrates such as sugars, lipids, and proteins into small molecule metabolites such as short-chain fatty acids (SCFAs) and amino acids but also participate in the metabolic cycle of bile acids and bilirubin in the intestine, which has an important impact on host metabolism (Zheng et al. 2016). Fecal metabolomics can be used to define the characteristics of the host gut microbiota and identify and quantify the small molecular compounds produced by the gut microbiota (Hung et al. 2022). Metabolomics and metabolite profiling have been extensively employed in the identification of biomarkers for disease, and untargeted analysis offers the chance to thoroughly examine metabolic alterations and find potential biomarkers in health and disease (Pu et al. 2021; Qu et al. 2021).

Most of the differential metabolites found in this investigation. They were mainly annotated to “lipid metabolism” and the “dopaminergic synapse pathway”. These metabolites and signaling pathways were of concern to us.

Lipids include fats (triglycerides) and lipids (phospholipids, sterols), which provide essential fatty acids and energy (Fahy et al. 2009). Oxidation is the primary function of lipids in body, and adipose tissue is the body’s store of energy. Lipid metabolism has been shown to play a role in the control of a variety of cellular functions, including cell division, survival, apoptosis, inflammation, motility, membrane homeostasis, chemotherapeutic response, and drug resistance (Huang and Freter 2015). Fatty acids are associated with nutritional and oxidative stress and can act as the only source of carbon and energy (Wang et al. 2022b). Disorders of lipid metabolism are metabolic features of many malignant tumors (Maan et al. 2018; Zhang et al. 2021). Thus, lipid metabolism is an important aspect in evaluating host health. Lipid metabolism is controlled by multiple signaling pathways, which also results in a wide range of bioactive lipid compounds (Hirsch et al. 2010). This is an important aspect of future research aimed at exploring regulatory genes related to lipid metabolism in *C. elaphus kansuensis*.

The mammalian gut microbiota breaks down amino acids to produce dopamine (Wall et al. 2014). Dopaminergic neuron firing is closely correlated with target region modulation and reinforcement processing (Menegas et al. 2018). Dopamine regulates a variety of activities in invertebrate species, including those related to learning, motor control, and sensory perception (Verlinden 2018). It interacts with a wide variety of receptors at the molecular level, modulating circuit excitability on timescales ranging from seconds to minutes (Beaulieu and Gainetdinov 2011). Dopamine undoubtedly plays a role in nearly every mammalian cortical/pallial circuit, yet the architecture differs dramatically both molecularly and anatomically. It is a class of catecholamines found in both the plant and animal kingdoms (Liu et al. 2020). In humans, dopamine carries out a wide range of specific tasks, including those related to reward, coordination of movement, metabolism, and the production of hormones (Howe and Dombeck 2016). According to Lerner et al. (2021), dopamine is a key regulator of learning and motivation. It rises in response to signals directing movement but not in response to signals directing immobility (Syed et al. 2016). The motor cortex, visual cortex, auditory cortex, olfactory cortex, taste cortex, and soluble solids content (SSC) have all been demonstrated in studies to be able to become more malleable in response to dopamine (Macedo-Lima and Remage-Healey 2021). The differential metabolites annotated to the dopamine signaling pathway may be associated with the difference in physical activity between CRD and WRD. Numerous studies on plants have shed light on the physiological, pharmacological, and stress reactions of this molecule (Liu et al. 2020; Liu et al. 2022). Dopamine might be involved in the growth and development of plants (Akula and Mukherjee 2020; Du et al. 2021). Additionally, dopamine, whether endogenously or exogenously given, can increase the resistance to a number of abiotic stressors, including salt, nutrition, and drought stress (Liu et al. 2020). Differential metabolites significantly annotated to dopamine signaling pathways may also be involved in environmental responses of *C. elaphus kansuensis*.

### Combined analysis of gut microbiota with fecal metabolites

In this study, the 10 genera with the most significant differences between the CRD and WRD groups in the metagenomic data and the 20 metabolites with the most significant differences between the CRD and WRD groups in the metabolomic data were selected for combined analysis. Among those differential metabolites, 2-(2,4-dihydroxyphenyl)-3,5,7-trihydroxy-4H-chromen-4-one, ascorbic acid, DL-tryptophan, and levodopa were annotated to as flavones, furanones, indolyl carboxylic acids and derivatives and amino acids, peptides, and analogs, respectively. *N*-acetyldopamine and epinephrine were annotated as benzenediols. 16(*R*)-HETE, 2-methylbutyroylcarnitine and sorbic acid were annotated as fatty acyls.

Combined analysis showed that the abundance of 2-(2,4-dihydroxyphenyl)-3,5,7-trihydroxy-4H-chromen-4-one was negatively related to *Allochromatium* and *Macrococcus*. Flavonoids are widespread throughout the plant kingdom. Flavones are a class of flavonoids that have broad biological activities in plants, humans, and animals (Chen et al. 2022a), including anti-inflammatory activity (Wang et al. 2021). *Allochromatium* belongs to the *Chromatiaceae* family. The biological functions of this genus in humans and animals have not been reported. *Macrococcus* have been reported to be not directly related to human disease, but these organisms are capable of transferring antibiotic resistance genes into *Staphylococcus* and other commensal pathogens (Fernandez et al. 2021). The higher abundance of *Macrococcus* bacteria in WRD may result in a decrease in immunity and an increase in the drug resistance of pathogenic bacteria in vivo in wild red deer. In contrast, wild red deer populations are well adapted to the field environment. Although wild deer have access to more abundant food in summer, the species still faces multiple pressures from the harsh environment, in which it lives for a long time. Conservation of wild *C. elaphus kansuensis* is necessary.

In addition, ascorbic acid (AA, also known as vitamin C) also differed significantly between CRD and WRD and was more abundant in CRD than in WRD. AA is very popular for its antioxidant properties (Padayatty and Levine 2016) and plays roles in the health maintenance of humans (Granger and Eck 2018). AA is annotated to the hypoxia-inducible factor-1 (HIF-1) signaling pathway and acts as an oxidative stress sensor to play roles in HIF-1 hydroxylation (Grano and De Tullio 2007; Padayatty and Levine 2016). This further indicated that WRD face stronger environmental stresses such as low oxygen than CRD, which might make it more difficult for WRD to survive in the non-grassy season.

In conclusion, the composition and abundance of gut microbiota and fecal metabolites differed between captive and wild Gansu red deer. The predominant phyla in the gut microbiome of Gansu red deer were *Firmicutes* and *Bacteroidetes*. Notably, *Bacteroides* was the dominant gut microbiota in the captive population, while *Bacillus* was the dominant gut microbiota in the wild population. Among the fecal metabolites found in CRD and WRD, the differential metabolites annotated to lipid metabolism and the “dopaminergic synapse pathway” might play important roles in the host’s adaptation to high-altitude survival for WRD and to captive environment for CRD.

## Supplementary information

ESM 1 (PDF 2.68 mb)

## Data Availability

The raw data generated with 16S rDNA sequencing and metagenomic sequencing in this study are available in the NCBI SRA database under BioProject Accession Numbers PRJNA884655 and PRJNA884819. Available online: (https://dataview.ncbi.nlm.nih.gov/object/PRJNA884655?reviewer=ba34k6nnpvt0ll71245ot0koen; https://dataview.ncbi.nlm.nih.gov/object/PRJNA884819?reviewer=u6s89vefh7r7jv4i179rjf4u8m). The LC-MS/MS data have been deposited in the MetaboLights database (https://www.ebi.ac.uk/metabolights/) with the dataset identifier MTBLS6121 (https://www.ebi.ac.uk/metabolights/MTBLS6121).
