# Supplementary material for: Integrated omics analysis reveals the alteration of gut microbiota and fecal metabolites in Cervus elaphus kansuensis
Source: Appl Microbiol Biotechnol. 2024 Jan 15;108:125. doi: 10.1007/s00253-023-12841-5 (PMC10789680; doi:10.1007/s00253-023-12841-5)
Supplement: Supplementary file 1 [file 253_2023_12841_MOESM1_ESM.pdf]

Article Title: Integrated omics analysis reveals the alteration of gut microbiota and fecal metabolites in *Cervus elaphus kansuensis*

Journal Name: Applied Microbiology and Biotechnology

File: Supplemental material

Authors: Zhenxiang Zhang [1, 2], Changhong Bao [1], Zhaonan Li [1], Caixia He [1], Wenjie Jin [1], Changzhong Li [1, *], Yanxia Chen [1, *]

Affiliations:

[1] College of Eco-Environmental Engineering, Qinghai University, Xining, China;

[2] Qinghai Provincial Key Laboratory of Adaptive Management on Alpine Grassland, Academy of Animal Science and Veterinary Medicine, Qinghai University, Xining, China

Corresponding Authors:

Yanxia Chen, E-mail: chenyanxia2021@qhu.edu.cn

Changzhong Li, E-mail: lichangzhong@qhu.edu.cn

Supplemental Table S1: General characteristics of 16S rDNA sequencing

CRD				WRD			
Samples	Raw tags	Effective tags	ASVs	Samples	Raw tags	Effective tags	ASVs
C1	110,435	62,021	1,608	W1	101,297	63,916	1,374
C2	104,703	58,579	1,569	W2	102,579	65,779	1,273
C3	101,725	62,202	1,580	W3	105,724	69,542	1,294
C4	102,471	61,627	1,508	W4	114,024	71,645	1,267
C5	99,123	56,717	1,459	W5	83,665	48,339	945
C6	107,389	64,691	1,567	W6	74,832	44,425	833
C7	116,317	67,213	1,650	W7	102,870	63,449	1,261
C8	107,645	64,350	1,171	W8	81,717	50,562	981
C9	103,295	68,031	1,645	W9	107,226	63,848	1,358
C10	102,882	62,093	1,477	W10	100,488	69,919	1,315
C11	103,334	55,695	1,392	W11	114,428	70,355	1,302
C12	109,557	63,007	1,577	W12	87,503	53,777	749
C13	107,085	56,320	1,563	W13	81,848	47,747	996
C14	105,037	59,644	1,491	W14	79,109	46,495	849
C15	99,636	59,933	1,636	W15	111,907	60,195	1,306
C16	111,427	62,233	1,506	W16	80,701	48,632	784
C17	114,035	65,666	1,560	W17	90,168	65,245	508
C18	74,990	45,163	866	W18	64,053	40,988	796
C19	112,979	68,196	1,613	W19	106,106	67,270	1,201
C20	103,949	58,423	1,451				
C21	100,109	57,734	1,463				
C22	103,097	63,058	1,467				

Supplemental Table S2: Statistics of 16S rDNA sequencing

Taxon	C.1	C.2	C.3	C.4	C.5	C.6	C.7	C.8	C.9	C.10	C.11	C.12	C.13	C.14	C.15	C.16	C.17	C.18	C.19	C.20	C.21	C.22	Average_C	W.1	W.2	W.3	W.4	W.5	W.6	W.7	W.8	W.9	W.10	W.11	W.12	W.13	W.14	W.15	W.16	W.17	W.18	W.19	Average_W
Acidobacteriota	0	0	0	0	0	0	0	0	0	0	0	0	0	0	0	0	0	0	0	0	0	0	0	0	0	0	0	264	114	0	96	0	0	0	124	192	167	0	138	0	0	0	57.63157895
Actinobacteriota	424	287	285	241	71	88	81	90	102	72	141	242	404	231	199	103	64	237	91	60	56	96	166.5909091	142	53	26	33	566	470	29	262	36	75	66	1279	157	1343	104	528	1137	51	1496	413.3157895
Armatimonadota	1	0	0	0	0	0	0	0	0	0	0	1	0	0	0	0	0	0	0	0	0	0	0.090909091	0	0	0	0	21	18	0	10	0	0	0	8	43	14	0	15	0	0	3	6.947368421
Bacteroidota	12465	11709	11310	11516	11336	10244	9182	10489	10032	11647	10777	10788	9341	10181	9936	10851	11508	12008	10043	11064	10845	11106	10835.36364	9557	8725	11999	11938	6670	7488	9512	7299	8575	9938	8137	4647	7289	6297	9710	6000	3262	8019	5530	7925.894737
Bdellovibrionota	0	0	3	0	0	0	0	0	0	0	0	0	0	1	4	0	0	0	0	0	0	0	0.363636364	0	0	0	0	0	0	0	0	0	0	0	0	9	0	0	4	0	0	0	0.684210526
Campilobacterota	115	58	65	0	0	0	0	0	0	0	0	0	86	33	53	0	0	21	0	0	0	0	19.59090909	36	0	0	0	152	141	0	274	0	0	0	0	0	0	0	0	0	0	2	31.84210526
Chloroflexi	0	1	0	0	0	0	0	0	0	0	0	0	0	0	2	0	0	0	0	0	0	0	0.136363636	0	0	0	4	165	127	0	216	0	0	0	163	132	163	4	115	0	0	3	57.47368421
Cyanobacteria	201	149	146	278	75	40	44	19	61	61	38	123	244	94	69	144	57	31	28	25	32	105	93.81818182	81	30	32	24	25	0	53	15	115	50	40	6	167	7	31	0	3	22	108	42.57894737
Deferribacterota	14	18	22	0	0	0	0	0	0	0	44	14	14	17	14	0	0	0	0	0	0	0	7.136363636	12	0	0	0	0	29	1	11	0	0	0	0	0	0	31	0	0	0	14	5.157894737
Desulfobacterota	496	233	243	290	224	138	106	53	211	213	109	146	228	202	251	302	196	151	117	143	173	308	206.0454545	80	52	38	64	5	59	43	52	99	59	34	30	124	31	74	24	19	8	66	50.57894737
Elusimicrobiota	51	48	57	79	11	16	15	12	33	13	5	11	37	11	13	30	11	19	9	17	34	16	24.90909091	20	23	4	8	13	7	8	0	26	41	0	0	7	0	11	0	6	16	5	10.26315789
Euryarchaeota	71	166	135	123	155	573	354	353	529	143	350	437	423	590	807	515	313	65	703	397	323	599	369.2727273	540	683	304	713	59	102	215	153	704	644	400	86	33	122	1513	111	480	100	1025	420.3684211
Fibrobacterota	13	22	5	0	5	3	10	0	4	14	0	0	2	0	0	3	7	0	9	4	0	0	4.590909091	3	5	52	26	0	0	0	3	2	2	0	0	0	0	0	2	0	53	0	7.789473684
Firmicutes	17729	19382	20225	20160	21280	22043	23177	22536	22032	21017	20367	20833	20986	19725	20824	21162	20818	20440	21761	20910	21696	20475	20889.90909	22752	23972	21096	20674	23827	23838	23704	23620	23839	21656	24946	25383	22834	23711	21865	25002	25715	25097	24906	23601.94737
Gemmatimonadota	0	2	0	0	0	0	0	0	0	0	0	2	1	9	0	0	0	0	0	0	0	0	0.636363636	3	0	0	0	60	17	0	21	0	0	0	19	15	27	0	15	0	0	0	9.315789474
Halobacterota	0	0	8	5	0	0	0	0	0	0	0	13	2	0	0	1	0	0	5	0	0	0	1.545454545	0	0	0	0	0	0	0	0	0	0	0	0	2	0	0	0	0	0	0	0.105263158
Latescibacterota	0	0	0	0	0	0	0	0	0	0	0	0	0	0	0	0	0	0	0	0	0	0	0	0	0	0	0	10	0	0	0	0	0	0	0	4	7	0	0	0	0	0	1.105263158
Myxococcota	0	8	0	1	0	0	1	0	0	0	0	0	0	15	1	2	0	0	0	0	2	0	1.363636364	0	0	0	0	150	49	0	108	0	0	0	62	64	41	0	47	0	0	0	27.42105263
NB1-j	0	0	0	0	0	0	0	0	0	0	0	0	0	0	0	0	0	0	0	0	0	0	0	0	0	0	0	9	0	0	2	0	0	0	0	0	4	0	0	0	0	0	0.789473684
Nitrospirota	0	2	0	0	0	0	0	0	0	0	0	0	0	2	3	0	0	0	0	0	0	0	0.318181818	0	0	0	0	107	76	0	66	0	0	0	157	195	173	0	133	0	0	0	47.73684211
Patescibacteria	77	125	34	22	25	55	73	31	45	22	51	69	44	98	47	28	37	0	87	42	44	82	51.72727273	13	13	10	28	116	174	23	21	11	102	1	143	26	459	21	481	47	28	30	91.94736842
Proteobacteria	1002	600	753	173	167	131	88	77	260	125	119	393	889	505	610	115	98	445	102	122	117	287	326.2727273	405	55	63	104	1324	835	42	1224	122	318	32	1519	2122	1037	96	972	2945	235	403	729.1052632
SAR324_clade(Marine_group_B)	5	6	6	0	0	2	2	0	0	0	0	0	0	0	2	3	0	0	0	0	1	3	1.363636364	0	0	0	0	8	3	0	13	0	0	0	5	0	0	0	5	0	0	1	1.842105263
Spirochaetota	807	541	80	172	289	305	247	12	305	223	370	354	191	822	276	133	100	99	79	323	126	468	287.3636364	7	49	53	51	19	94	34	118	118	787	22	17	66	30	116	78	65	31	44	94.68421053
Synergistota	0	0	0	0	0	0	0	0	0	0	0	0	6	0	0	0	0	0	3	0	0	3	0.545454545	0	0	0	0	0	0	0	0	0	0	0	0	0	0	0	0	0	0	0	0
Thermoplasmatota	0	0	0	6	0	0	0	0	3	1	0	3	4	1	0	8	0	0	0	2	8	10	2.090909091	0	0	0	0	0	0	0	0	0	0	0	0	0	0	0	0	0	0	0	0
Verrucomicrobiota	210	324	304	615	43	43	301	9	64	130	1310	250	777	1144	570	281	472	165	644	572	224	123	389.7727273	28	21	4	10	106	32	17	74	23	8	3	29	191	48	98	4	2	21	45	40.21052632
WPS-2	0	0	0	0	0	0	0	0	0	0	0	0	0	0	0	0	0	0	0	0	0	0	0	0	0	0	0	5	8	0	6	0	0	0	0	9	0	0	0	0	0	0	1.473684211
Zixibacteria	0	0	0	0	0	0	0	0	0	0	0	0	0	0	0	0	0	0	0	0	0	0	0	0	0	0	0	0	0	0	0	0	0	0	4	0	0	0	7	0	0	0	0.578947368

Supplemental Table S3: Relative abundance of gut microbiota detected by 16S rDNA sequencing after homogenization at phylum level

Taxonomy	WRD	CRD	Tax_detail
Firmicutes	0.70075	0.620228	k__*Bacteria* ;p__*Firmicutes* ;
Bacteroidota	0.235322	0.321706	k__*Bacteria* ;p__*Bacteroidota* ;
Proteobacteria	0.021647	0.009687	k__*Bacteria* ;p__*Proteobacteria* ;
Euryarchaeota	0.012481	0.010964	k__*Archaea* ;p__*Euryarchaeota* ;
Actinobacteriota	0.012271	0.004946	k__*Bacteria* ;p__*Actinobacteriota* ;
Verrucomicrobiota	0.001194	0.011572	k__*Bacteria* ;p__*Verrucomicrobiota* ;
Spirochaetota	0.002811	0.008532	k__*Bacteria* ;p__*Spirochaetota* ;
Desulfobacterota	0.001502	0.006118	k__*Bacteria* ;p__*Desulfobacterota* ;
Patescibacteria	0.00273	0.001536	k__*Bacteria* ;p__*Patescibacteri* a;
Cyanobacteria	0.001264	0.002785	k__*Bacteria* ;p__*Cyanobacteria* ;
Campilobacterota	0.000945	0.000582	k__*Bacteria* ;p__*Campilobacterota* ;
Acidobacteriota	0.001711	0	k__*Bacteria* ;p__*Acidobacteriota* ;
Chloroflexi	0.001706	0.000004	k__*Bacteria* ;p__*Chloroflexi* ;
Nitrospirota	0.001417	0.000009	k__*Bacteria* ;p__*Nitrospirota* ;
Myxococcota	0.000814	0.00004	k__*Bacteria* ;p__*Myxococcota* ;
Elusimicrobiota	0.000305	0.00074	k__*Bacteria* ;p__*Elusimicrobiota* ;
Gemmatimonadota	0.000277	0.000019	k__*Bacteria* ;p__*Gemmatimonadota* ;
Fibrobacterota	0.000231	0.000136	k__*Bacteria* ;p__*Fibrobacterota* ;
Deferribacterota	0.000153	0.000212	k__*Bacteria* ;p__*Deferribacterota* ;
Armatimonadota	0.000206	0.000003	k__*Bacteria* ;p__*Armatimonadota* ;
Halobacterota	0.000003	0.000046	k__*Archaea* ;p__*Halobacterota* ;
SAR324_clade(Marine_group_B)	0.000055	0.00004	k__*Bacteria* ;p__*SAR324_clade(Marine_group_B)* ;
Thermoplasmatota	0	0.000062	k__*Archaea* ;p__*Thermoplasmatota* ;
Latescibacterota	0.000033	0	k__*Bacteria* ;p__*Latescibacterota* ;
Bdellovibrionota	0.00002	0.000011	k__*Bacteria* ;p__*Bdellovibrionota* ;
NB1-j	0.000023	0	k__*Bacteria* ;p__*NB1-j* ;
WPS-2	0.000044	0	k__Bacteria;p__*WPS-2* ;
Zixibacteria	0.000017	0	k__*Bacteria* ;p__*Zixibacteria* ;
Synergistota	0	0.000016	k__*Bacteria* ;p__*Synergistota* ;
Others	0.000066	0.000005	Others

Supplemental Table S4: Relative abundance of gut microbiota detected by 16S rDNA sequencing after homogenization at genus level

Taxonomy	WRD	CRD
Others	0.192426	0.182979
UCG-005	0.138798	0.127736
Rikenellaceae_RC9_gut_group	0.086585	0.117099
UCG-010	0.084722	0.11039
Eubacterium_coprostanoligenes_group	0.04903	0.049079
Christensenellaceae_R-7_group	0.036849	0.035573
Bacteroides	0.02914	0.035518
Alistipes	0.023091	0.034067
Monoglobus	0.028429	0.025419
Bacteroidales_RF16_group	0.007626	0.022234
Clostridia_UCG-014	0.025421	0.015964
dgA-11_gut_group	0.02123	0.015631
Prevotellaceae_UCG-003	0.002147	0.015183
UCG-009	0.007902	0.012313
Prevotellaceae_UCG-004	0.020025	0.011599
Akkermansia	0.001061	0.01133
Methanobrevibacter	0.011362	0.010853
Romboutsia	0.00966	0.009456
Muribaculaceae	0.014665	0.008524
Treponema	0.002785	0.008338
Clostridia_vadinBB60_group	0.007118	0.00829
F082	0.000674	0.00784
NK4A214_group	0.007435	0.007359
UCG-002	0.001999	0.006794
Ruminococcus	0.00636	0.00612
Mailhella	0.001297	0.005837
Oscillibacter	0.003528	0.005159
Lachnospiraceae_UCG-010	0.00275	0.005088
Paeniclostridium	0.000422	0.00476
Family_XIII_AD3011_group	0.001677	0.004628
p-2534-18B5_gut_group	0.000117	0.003939
p-251-o5	0.004855	0.003912
Candidatus_Soleaferrea	0.002897	0.002969
Clostridium_sensu_stricto_1	0.000588	0.002802
Phascolarctobacterium	0.00098	0.002502
Gastranaerophilales	0.001088	0.002401
Incertae_Sedis	0.002072	0.002296
RF39	0.002166	0.002285
Agathobacter	0.00709	0.001785
Papillibacter	0.000772	0.001679
Bacillus	0.041751	0.001641
Lachnospiraceae_NK3A20_group	0.000239	0.001617
Eubacterium_ruminantium_group	0.001213	0.001613
Hydrogenoanaerobacterium	0.0006	0.001565
Candidatus_Saccharimonas	0.000616	0.001536
Prevotellaceae_UCG-001	0.000541	0.001439
Izemoplasmatales	0.001074	0.001186
Turicibacter	0.001184	0.00118
Dorea	0.000953	0.001104
Anaerorhabdus_furcosa_group	0.000859	0.001085
Olsenella	0.000186	0.001053
Anaerovorax	0.000473	0.001032
Ruminococcaceae	0.00142	0.000955
Cellulosilyticum	0.000048	0.000931
Defluviitaleaceae_UCG-011	0.000703	0.000885
Terrisporobacter	0.000461	0.000827
Pseudomonas	0.000258	0.000789
Elusimicrobium	0.000294	0.00074
Eubacterium_ventriosum_group	0.000178	0.000727
Prevotella	0.002111	0.000723
Arthrobacter	0.004535	0.000718
Eubacterium_nodatum_group	0.000934	0.00068
Bacteroidales_UCG-001	0.000028	0.00068
Sporichthyaceae	0.000073	0.000648
Pygmaiobacter	0.000692	0.000644
Lachnospiraceae_NK4A136_group	0.001491	0.000637
Alloprevotella	0.000278	0.000623
Flavobacterium	0.000147	0.000619
Rheinheimera	0.000139	0.000613
M2PB4-65_termite_group	0.000014	0.000598
Saccharofermentans	0.000248	0.000557
Oscillospiraceae	0.000263	0.000547
Pseudarcobacter	0.000061	0.000528
Eubacterium_siraeum_group	0.001575	0.000507
Colidextribacter	0.000947	0.000499
Odoribacter	0.000167	0.000471
Lactobacillus	0.002807	0.000464
Parasutterella	0.000613	0.000448
Oscillospira	0.000838	0.000441
Lysinibacillus	0.019453	0.000437
Acetitomaculum	0.000091	0.000436
Escherichia-Shigella	0.000073	0.000433
Sphingorhabdus	0.000069	0.000402
Eubacterium_brachy_group	0.001108	0.000375
hgcI_clade	0.000052	0.000366
Parabacteroides	0.000058	0.000351
Incertae_Sedis	0.000041	0.000335
V9D2013_group	0.000063	0.000331
Clostridium_sensu_stricto_6	0.000003	0.000323
Sutterella	0.000022	0.000286
DNF00809	0.00023	0.000282
Rikenella	0.000044	0.000281
gir-aah93h0	0.000009	0.000274

Eubacterium_hallii_group	0.000102	0.000271
Aeromonas	0.00012	0.00027
Bacteroidales	0.000011	0.000265
Paludicola	0.000102	0.000263
Mogibacterium	0.000144	0.000258
Caulobacter	0.000067	0.000246
Limnohabitans	0.000034	0.000242
Moryella	0.000134	0.000236
Solibacillus	0.013153	0.000228
Negativibacillus	0.000352	0.000227
Polynucleobacter	0.000016	0.000225
Lachnospiraceae_UCG-002	0.000078	0.000223
Chloroplast	0.000147	0.000221
Corynebacterium	0.000016	0.000221
XBB1006	0.000061	0.00022
Coprococcus	0.000053	0.00022
Mucispirillum	0.000153	0.000212
Prevotellaceae_Ga6A1_group	0.000003	0.000211
Succinivibrio	0.000019	0.000209
Candidatus_Planktophila	0.000028	0.000204
Shewanella	0.000025	0.0002
Lachnospiraceae_NK4B4_group	0.000102	0.000192
Enterorhabdus	0.000131	0.000185
Polaromonas	0.000041	0.000184
Pedobacter	0.000022	0.000179
Tuzzerella	0.000095	0.000178
Brevundimonas	0.000053	0.000174
Algoriphagus	0.000027	0.000174
Caproiciproducens	0.000106	0.000173
Catenibacillus	0	0.000166
Eubacterium_xylanophilum_group	0.000283	0.000158
Acinetobacter	0.005385	0.000155
Family_XIII_UCG-001	0.000139	0.000154
Desulfovibrio	0.000088	0.000151
Roseburia	0.000994	0.000147
Blautia	0.000184	0.000147
GWE2-31-10	0.000003	0.000143
Sediminibacterium	0.000011	0.000142
Cyanobium_PCC-6307	0.000019	0.000138
Fibrobacter	0.000231	0.000136
Klebsiella	0	0.000131
UCG-007	0.000086	0.000126
Rhodoferax	0.000038	0.000123
DTU014	0.000009	0.000123
Clostridium_methylpentosum_group	0.000044	0.000119
UCG-004	0.000095	0.000115
Frisingicoccus	0.000103	0.000113
Salinicoccus	0.000005	0.000101
Bifidobacterium	0.000089	0.000099
Methanosphaera	0.001119	0.000096
Ruminiclostridium	0.00003	0.000094
Peptococcus	0.000019	0.000094
UKL13-1	0	0.000094
Pseudarcicella	0.000014	0.000092
Faecalibacterium	0.000217	0.000089
Dubosiella	0.000145	0.000089
Coriobacteriaceae_UCG-002	0.00003	0.000089
Intestinimonas	0.000303	0.000086
Aeriscardovia	0.000006	0.000084
Succiniclasticum	0.000003	0.000084
Candidatus_Limnoluna	0	0.00008
Ruminobacter	0.000003	0.000078
Dechloromonas	0.0004	0.000077
Mitochondria	0.000022	0.000077
Lachnospiraceae_AC2044_group	0.000013	0.000077
Candidatus_Planktoluna	0.000013	0.000073
Parvibacter	0.000286	0.00007
Hydrogenophaga	0.000355	0.000067
Psychrobacillus	0.004936	0.000066
Allobaculum	0.000038	0.000066
Rhodobacter	0.000423	0.000063
Dinghuibacter	0.000028	0.000063
Howardella	0.000014	0.000063
Victivallaceae	0.000006	0.000063
Incertae_Sedis	0	0.000063
Planococcus	0.000003	0.000062
GKS98_freshwater_group	0.000006	0.000059
WCHB1-41	0.000002	0.000058
Erysipelatoclostridium	0.00015	0.000057
Acetobacter	0.000009	0.000057
Sanguibacteroides	0.000005	0.000055
Candidatus_Aquirestis	0	0.000055
Rurimicrobium	0.000002	0.000054
CL500-29_marine_group	0.000144	0.000053
Butyrivibrio	0.000011	0.000049
Anaerotruncus	0.000208	0.000047
Lacihabitans	0.000003	0.000046
Methanocorpusculum	0.000003	0.000046
Novosphingobium	0.000134	0.000045
Atopobium	0.000067	0.000045
Erysipelothrix	0.000009	0.000043
Citricoccus	0	0.000043
SAR324_clade(Marine_group_B)	0.000055	0.00004
Sphaerochaeta	0.000002	0.00004
NS11-12_marine_group	0	0.00004
Prevotellaceae_NK3B31_group	0.000041	0.000039

env.OPS_17	0.000006	0.000039
Fluviicola	0.000006	0.000036
Acidovorax	0.00032	0.000035
OLB12	0.000156	0.000035
Roseomonas	0.000041	0.000035
Dielma	0.000333	0.000034
Flavonifractor	0.000213	0.000034
Erysipelotrichaceae	0.000209	0.000032
Kocuria	0	0.000032
EMP-G18	0.000092	0.000031
Luteolibacter	0.000006	0.000031
Enteractinococcus	0.000003	0.000031
Methylotenera	0	0.000031
Tabrizicola	0	0.000031
Paraprevotella	0.000103	0.000028
Hyphomonas	0.000019	0.000028
Campylobacter	0	0.000028
Arenimonas	0.000111	0.000027
hoa5-07d05_gut_group	0.000014	0.000027
Lachnospiraceae_XPB1014_group	0.000006	0.000027
Malikia	0	0.000027
Salinimicrobium	0	0.000027
Candidatus_Aquiluna	0	0.000027
Helicobacter	0.000838	0.000026
Anaeroplasma	0.000405	0.000026
Breznakia	0.000045	0.000026
Kapabacteriales	0.00003	0.000026
Solitalea	0.000008	0.000026
Lactococcus	0.000005	0.000026
MWH-CFBk5	0	0.000026
Muribaculum	0.000203	0.000023
Lachnospiraceae_UCG-001	0.000181	0.000023
LD29	0.000005	0.000023
Ignavibacterium	0	0.000023
Pantoea	0.000713	0.000022
Microcystis_PCC-7914	0	0.000019
Comamonas	0.000506	0.000018
Lachnospira	0.000297	0.000018
Denitratisoma	0.000158	0.000018
PeM15	0.000084	0.000018
Caldicoprobacter	0	0.000018
Paludibacter	0	0.000016
Marvinbryantia	0.000073	0.000015
Anaerofustis	0.000006	0.000015
Pseudorhodobacter	0	0.000015
Rhodococcus	0.000392	0.000013
Solobacterium	0.000095	0.000013
Streptococcus	0.000045	0.000013
Cerasicoccus	0.000033	0.000013
Victivallis	0.000005	0.000013
Sandarakinorhabdus	0.000003	0.000013
Faecalitalea	0	0.000013
UCG-001	0.000048	0.000012
Sulfuritalea	0.000048	0.000012
Anaerovibrio	0.000008	0.000012
Ornithinimicrobium	0	0.000012
OC31	0	0.000012
COB_P4-1_termite_group	0	0.000012
Lachnospiraceae_UCG-006	0.000016	0.000011
OM27_clade	0.000006	0.000011
Desulfatiglans	0	0.000011
Lysobacter	0.00002	0.000009
Harryflintia	0.000014	0.000009
Phenylobacterium	0.000008	0.000009
Ileibacterium	0.000006	0.000009
Bilophila	0.000002	0.000009
Garicola	0	0.000009
Jeotgalicoccus	0	0.000009
Ferruginibacter	0.000266	0.000008
Syntrophococcus	0.000014	0.000008
Hirschia	0.000008	0.000008
Paenisporosarcina	0.000006	0.000008
Thiobacillus	0	0.000008
Vogesella	0	0.000008
Tyzzerella	0	0.000008
BSV26	0	0.000008
Sulfurifustis	0	0.000007
Rhodovastum	0	0.000007
UCG-008	0	0.000007
Georgenia	0	0.000007
Oceanobacillus	0	0.000007
Clostridium_sensu_stricto_13	0	0.000007
Eubacterium	0	0.000007
Catenibacterium	0	0.000007
Ornithinibacillus	0	0.000007
Sericytochromatia	0	0.000007
LD-RB-34	0	0.000007
Ellin6067	0.000158	0.000005
P3OB-42	0.000042	0.000005
Erysipelotrichaceae_UCG-009	0.000005	0.000005
Laceyella	0	0.000005
Aliicoccus	0	0.000005
Anaerosporobacter	0	0.000005
Altererythrobacter	0	0.000005
Aliidiomarina	0	0.000005
Sva0081_sediment_group	0	0.000005

Bacteroidales_BS11_gut_group	0	0.000005
Hoeflea	0	0.000005
Brevibacillus	0	0.000005
Isoptericola	0	0.000005
BIrii41	0	0.000005
Acetatifactor	0	0.000005
Paenibacillus	0.00065	0.000004
Terrimonas	0.000241	0.000004
A0839	0.000064	0.000004
Butyricicoccus	0.000059	0.000004
Candidatus_Stoquefichus	0.000016	0.000004
Devosia	0.000006	0.000004
Undibacterium	0.000006	0.000004
Eubacterium_oxidoreducens_group	0.000005	0.000004
Pelagibacterium	0.000002	0.000004
Christensenellaceae	0	0.000004
Azospirillum	0	0.000004
Atopostipes	0	0.000004
Cellvibrio	0	0.000004
Luteimonas	0	0.000004
GCA-900066575	0.000252	0.000003
Nocardioides	0.000147	0.000003
Armatimonas	0.000005	0.000003
Aliihoeflea	0	0.000003
CAG-352	0	0.000003
Halomonas	0.000011	0.000001
Aureimonas	0.000005	0.000001
Micropruina	0.002322	0
Nitrospira	0.001417	0
Saccharimonadales	0.001361	0
Dokdonella	0.001241	0
Ahniella	0.000655	0
Zoogloea	0.000598	0
Nakamurella	0.000586	0
Tessaracoccus	0.000497	0
TM7a	0.000484	0
SWB02	0.000366	0
Pseudoxanthomonas	0.000305	0
Ottowia	0.000302	0
Nitrosomonas	0.000302	0
DS-100	0.000295	0
Amaricoccus	0.00028	0
Haliangium	0.000248	0
Subgroup_17	0.000239	0
Thauera	0.000236	0
JGI_0001001-H03	0.000223	0
UTCFX1	0.000206	0
Fimbriimonadaceae	0.000202	0
Trichococcus	0.0002	0
IMCC26207	0.000184	0
Paracoccus	0.000183	0
Hyphomicrobium	0.000178	0
SC-I-84	0.000173	0
Candidatus_Alysiosphaera	0.000173	0
Ferribacterium	0.000166	0
PHOS-HE36	0.000163	0
Kineosphaera	0.000153	0
Candidatus_Accumulibacter	0.000152	0
Vicinamibacteraceae	0.00015	0
Candidatus_Competibacter	0.000145	0
Microbacterium	0.000136	0
Pseudoflavonifractor	0.000131	0
Subgroup_10	0.000131	0
Bradyrhizobium	0.000117	0
OLB14	0.000113	0
ASF356	0.000111	0
Bryobacter	0.000102	0
A4b	0.000102	0
AAP99	0.0001	0
AKYH767	0.000097	0
Cellulomonas	0.000091	0
RBG-13-54-9	0.000078	0
Defluviicoccus	0.000077	0
SJA-28	0.000073	0
Gemmobacter	0.000072	0
Thermomonas	0.000072	0
Nannocystis	0.000069	0
Mesorhizobium	0.000069	0
NS9_marine_group	0.000067	0
UBA1819	0.000066	0
Aeromonadales	0.000064	0
Kouleothrix	0.000063	0
BD1-7_clade	0.000058	0
Clostridium_sensu_stricto_3	0.000056	0
Polyangium	0.000056	0
Azospira	0.000055	0
Massilia	0.000053	0
Mycobacterium	0.000053	0
Aquimonas	0.000052	0
Micromonospora	0.000052	0
Defluviimonas	0.00005	0
Aquabacterium	0.00005	0
Arcobacter	0.000047	0
DEV007	0.000047	0
Sandaracinus	0.000047	0
mle1-27	0.000045	0

Pajaroellobacter	0.000045	0
Iamia	0.000044	0
WPS-2	0.000044	0
Propioniciclava	0.000042	0
Oerskovia	0.000036	0
Latescibacterota	0.000033	0
IMCC26256	0.000033	0
Proteiniclasticum	0.00003	0
Longivirga	0.000028	0
Inhella	0.000027	0
Shinella	0.000027	0
Amb-16S-1323	0.000027	0
Bosea	0.000027	0
UCG-003	0.000025	0
Lachnospiraceae_FCS020_group	0.000025	0
SBR1031	0.000025	0
Candidatus_Contendobacter	0.000023	0
Methylocystis	0.000023	0
NB1-j	0.000023	0
Subgroup_7	0.000023	0
Stella	0.000023	0
JG30-KF-CM45	0.000023	0
CCM19a	0.000023	0
C10-SB1A	0.000023	0
Mycetocola	0.000022	0
966-1	0.000022	0
Turneriella	0.000022	0
Psychrobacter	0.00002	0
Oikopleura	0.000019	0
Acetoanaerobium	0.000019	0
Coprobacillus	0.000019	0
Zixibacteria	0.000017	0
Pedomicrobium	0.000017	0
Flexilinea	0.000017	0
Lautropia	0.000016	0
Enhydrobacter	0.000014	0
Hahella	0.000014	0
Blfdi19	0.000014	0
Bdellovibrio	0.000014	0
Cloacibacterium	0.000014	0
Macellibacteroides	0.000014	0
Sandaracinobacter	0.000014	0
Candidatus_Jidaibacter	0.000014	0
Aridibacter	0.000013	0
Dermatophilaceae	0.000013	0
Elev-16S-1166	0.000013	0
Piscinibacter	0.000013	0
Thiothrix	0.000013	0
A21b	0.000011	0
Candidatus_Obscuribacter	0.000011	0
MVP-88	0.000011	0
Proteocatella	0.000011	0
OLB8	0.000011	0
Plasticicumulans	0.000009	0
LWQ8	0.000009	0
Mumia	0.000009	0
Clostridium_sensu_stricto_5	0.000009	0
Longilinea	0.000008	0
Pannonibacter	0.000008	0
Prosthecobacter	0.000008	0
A2	0.000008	0
Blastococcus	0.000008	0
37-13	0.000008	0
PB19	0.000008	0
Crenothrix	0.000006	0
Phaselicystis	0.000006	0
Methylophilus	0.000006	0
Collinsella	0.000006	0
Schwartzia	0.000005	0
Pseudobutyrivibrio	0.000005	0
Absconditabacteriales_(SR1)	0.000005	0
Selenomonas	0.000005	0
Quinella	0.000005	0
Oribacterium	0.000003	0

Supplemental Table S5: *T*-test of gut microbiota in captive and wild *Cervus elaphus kansuensis* at phylum level

Taxa	Average (C)	SD (C)	Average (W)	SD (W)	*P*_value	*Q*_values	Interval lower	Interval upper
k__*Bacteria*;p__*Firmicutes*;	0.62044	0.034241	0.700926	0.04343	1.83E-07	-0.03505	0.055387	0.105585
k__*Bacteria*;p__*Bacteroidota*;	0.321488	0.025311	0.23526	0.067591	2.74E-05	-0.03505	-0.12025	-0.05221
k__*Bacteria*;p__*Proteobacteria*;	0.009679	0.008408	0.021544	0.024064	0.053209	-0.03505	-0.00018	0.02391
k__*Archaea*;p__*Euryarchaeota*;	0.010956	0.006303	0.012461	0.01161	0.61808	-0.03505	-0.00462	0.007628
k__*Bacteria*;p__*Actinobacteriota*;	0.004947	0.003316	0.012267	0.015168	0.052871	-0.03505	-0.0001	0.014739
k__*Bacteria*;p__*Verrucomicrobiota*;	0.011605	0.01035	0.001192	0.001418	0.000119	-0.03505	-0.01504	-0.00579
k__*Bacteria*;p__*Spirochaetota*;	0.008535	0.006372	0.002819	0.005093	0.002818	-0.03505	-0.00934	-0.00209
k__*Bacteria*;p__*Desulfobacterota*;	0.006095	0.002779	0.001499	0.000914	8.89E-08	-0.03505	-0.00589	-0.0033
k__*Bacteria*;p__*Patescibacteria*;	0.00154	0.000849	0.002722	0.004217	0.244259	-0.03505	-0.00088	0.003241
k__*Bacteria*;p__*Campilobacterota*;	0.000579	0.001005	0.000952	0.00223	0.507982	-0.03505	-0.00077	0.001517
k__*Bacteria*;p__*Cyanobacteria*;	0.002784	0.002165	0.001272	0.001336	0.009814	-0.03505	-0.00264	-0.00039
k__*Bacteria*;p__*Acidobacteriota*;	0	0	0.0017	0.002486	0.008018	-0.03505	0.000502	0.002899
k__*Bacteria*;p__*Chloroflexi*;	4.05E-06	1.39E-05	0.001717	0.002349	0.005199	-0.03505	0.000581	0.002846
k__*Bacteria*;p__*Nitrospirota*;	1.35E-05	3.52E-05	0.001416	0.002085	0.008933	-0.03505	0.000397	0.002408
k__*Bacteria*;p__*Myxococcota*;	4.59E-05	0.00011	0.000808	0.001286	0.018977	-0.03505	0.000141	0.001383
k__*Bacteria*;p__*Elusimicrobiota*;	0.000738	0.000565	0.000294	0.000312	0.003211	-0.03505	-0.00073	-0.00016
k__*Bacteria*;p__*Gemmatimonadota*;	1.62E-05	5.16E-05	0.000277	0.000458	0.023772	-0.03505	3.88E-05	0.000482
k__*Bacteria*;p__*Fibrobacterota*;	0.000136	0.000175	0.000239	0.0005	0.40316	-0.03505	-0.00015	0.000353
k__*Bacteria*;p__*Deferribacterota*;	0.000206	0.000329	0.000152	0.000287	0.571447	-0.03505	-0.00025	0.00014
k__*Bacteria*;p__*Armatimonadota*;	5.40E-06	1.97E-05	0.000213	0.000344	0.017196	-0.03505	4.13E-05	0.000373
k__Archaea;p__Halobacterota;	4.86E-05	0.000115	4.69E-06	2.04E-05	0.093199	-0.03505	-9.58E-05	7.96E-06
k__*Bacteria*;p__*SAR324_clade(Marine_group_B)*;	4.59E-05	7.14E-05	5.63E-05	0.000102	0.713373	-0.03505	-4.67E-05	6.74E-05

k__*Archaea*;p__*Thermoplasmatota*;	6.34E-05	8.90E-05	0	0	0.003078	-0.03505	-0.0001	-2.40E-05

Supplemental Table S6: General characteristics of metagenomic sequencing data

Sample ID	Raw data	Clean data	Effective (%)	ORFs NO.	Integrity ORFs	Total length (Mbp)	Average length (bp)	GC
C17	6,608.30	6,597.13	99.831	502,941	123,924 (24.64 %)	279.9	556.52	47.1
C18	5,902.33	5,890.88	99.806	405,168	104,961 (25.91 %)	224.06	553.02	44.5
C19	6,671.23	6,656.41	99.778	507,213	139,382 (27.48 %)	283.06	558.08	44.66
W17	6,320.78	6,315.53	99.917	485,651	161,574 (33.27 %)	260.21	535.79	50.25
W18	6,671.93	6,652.07	99.702	573,031	166,493 (29.05 %)	317.02	553.23	46.3
W19	6,745.71	6,728.67	99.747	541,816		307.48	567.51	46.31

Supplemental Table S7: All differential gut microbiota at genus level based on metagenomic sequencing

Taxa	Mean (group 1)	standard error (group 1)	Mean (group 2)	standard error (group 2)	*P* value	*Q* value
f__Prevotellaceae;g__Paraprevotella	0.013103322	0.000147986	0.002110989	0.000586958	0.000124	0.165747
f__Chromatiaceae;g__Allochromatium	0	0	2.43E-06	2.44E-07	0.000628	0.165747
f__Staphylococcaceae;g__Macrococcus	6.57E-07	1.60E-07	3.50E-06	2.37E-07	0.000666	0.165747
f__Erysipelotrichaceae;g__Allobaculum	0.000723546	4.37E-05	0.000109777	4.66E-05	0.000705	0.165747
f__Prevotellaceae;g__Alloprevotella	0.00090268	1.69E-05	0.000188663	7.50E-05	0.000784	0.165747
f__Synergistaceae;g__Thermovirga	2.15E-06	2.33E-07	0	0	0.000823	0.165747
f__Cellvibrionaceae;g__Cellvibrio	3.75E-06	2.59E-07	2.76E-07	2.76E-07	0.000861	0.165747
f__Chitinophagaceae;g__Chitinophaga	0.000177543	8.68E-06	9.20E-05	4.38E-06	0.000945	0.165747
f__Saprospiraceae;g__Saprospira	8.07E-06	9.27E-07	1.07E-07	7.00E-08	0.001025	0.165747
f__Flavobacteriaceae;g__Gillisia	2.73E-05	2.03E-06	8.15E-06	1.06E-06	0.001154	0.165747
f__Budviciaceae;g__Leminorella	2.88E-06	3.36E-07	4.27E-08	4.27E-08	0.001192	0.165747
f__Flavobacteriaceae;g__Polaribacter	8.58E-05	7.03E-06	2.49E-05	2.61E-06	0.001498	0.173312
f__Kordiimonadaceae;g__Kordiimonas	8.56E-06	3.27E-07	1.23E-06	8.66E-07	0.001536	0.173312
f__Prevotellaceae;g__Prevotellamassilia	0.000268921	6.07E-06	6.46E-05	2.64E-05	0.001617	0.173312
f__Flavobacteriaceae;g__Maribacter	6.49E-05	6.80E-06	1.47E-05	1.05E-06	0.001782	0.173312
f__Salinivirgaceae;g__Salinivirga	8.38E-05	7.92E-06	1.35E-05	5.74E-06	0.001952	0.173312
f__Flammeovirgaceae;g__Marivirga	3.58E-05	1.36E-06	1.45E-05	2.64E-06	0.00199	0.173312
f__Siphoviridae;g__P70virus	0	0	1.55E-06	2.21E-07	0.002117	0.173312
f__Podoviridae;g__Cba41virus	1.10E-06	7.87E-07	7.20E-06	4.23E-07	0.002246	0.173312
f__Desulfobacteraceae;g__Desulfatirhabdium	4.79E-06	5.54E-07	3.57E-07	3.57E-07	0.002284	0.173312
f__Cellulomonadaceae;g__Paraoerskovia	4.56E-07	4.56E-07	4.77E-06	5.00E-07	0.002502	0.173312
f__Unclassified;g__Candidatus Adiutrix	4.45E-06	6.94E-07	3.97E-08	3.97E-08	0.002583	0.173312
f__Acidaminococcaceae;g__Phascolarctobacterium	0.004660239	0.000486723	0.000764635	0.000391394	0.002662	0.173312
f__Rhodocyclaceae;g__Candidatus Dactylopiibacterium	4.46E-06	6.67E-07	3.12E-07	1.57E-07	0.002832	0.173312
f__Erysipelotrichaceae;g__Eggerthia	7.52E-06	5.08E-07	1.32E-05	8.04E-07	0.002962	0.173312
f__Carnobacteriaceae;g__Jeotgalibaca	7.41E-06	4.94E-08	2.09E-06	9.07E-07	0.003085	0.173312
f__Halomonadaceae;g__Chromohalobacter	1.96E-06	3.37E-07	0	0	0.003209	0.173312
f__Mesoaciditogaceae;g__Mesoaciditoga	3.08E-06	2.87E-07	6.70E-07	3.15E-07	0.003371	0.173312
f__Bacillaceae;g__Pontibacillus	1.34E-05	1.31E-06	6.86E-05	9.90E-06	0.003643	0.173312
f__Unclassified;g__Candidatus Fonsibacter	1.49E-06	2.63E-07	3.49E-08	3.49E-08	0.003818	0.173312
f__Halobacteroidaceae;g__Candidatus Frackibacter	5.55E-06	4.93E-07	1.76E-05	2.17E-06	0.00399	0.173312
f__Flavobacteriaceae;g__Chryseobacterium	0.000441008	2.69E-05	0.000282223	1.22E-05	0.004074	0.173312
f__Pectobacteriaceae;g__Pectobacterium	1.46E-06	1.13E-07	3.06E-06	2.79E-07	0.004282	0.173312
f__Planctomycetaceae;g__Mariniblastus	2.68E-06	2.15E-07	7.59E-07	2.98E-07	0.004411	0.173312
f__Rhodospirillaceae;g__Nisaea	2.19E-06	4.24E-07	3.23E-08	3.23E-08	0.004716	0.173312
f__Chitinophagaceae;g__Parafilimonas	6.78E-06	7.00E-07	1.41E-06	8.07E-07	0.004754	0.173312
f__Crocinitomicaceae;g__Fluviicola	9.59E-05	1.35E-05	1.60E-05	8.62E-06	0.004933	0.173312
f__Halobacteroidaceae;g__Selenihalanaerobacter	2.83E-06	3.71E-07	5.32E-07	2.79E-07	0.005067	0.173312
f__Prolixibacteraceae;g__Tangfeifania	5.80E-05	2.45E-06	1.88E-05	7.59E-06	0.005233	0.173312
f__Schleiferiaceae;g__Schleiferia	5.96E-06	8.54E-07	1.02E-06	5.33E-07	0.005271	0.173312
f__Idiomarinaceae;g__Idiomarina	9.49E-06	8.97E-07	3.38E-06	8.71E-07	0.005407	0.173312
f__Flavobacteriaceae;g__Riemerella	2.11E-05	9.09E-08	3.07E-05	1.97E-06	0.005446	0.173312
f__Flavobacteriaceae;g__Winogradskyella	2.22E-05	3.14E-06	6.76E-06	5.70E-07	0.005565	0.173312
f__Chlamydiaceae;g__Chlamydia	0.000822852	0.00020969	0.002424575	0.000256187	0.005603	0.173312
f__Lentimicrobiaceae;g__Lentimicrobium	0.001017613	0.000187655	8.85E-05	4.88E-05	0.005687	0.173312
f__Sporocadaceae;g__Pestalotiopsis	0	0	2.52E-06	5.28E-07	0.005725	0.173312
f__Flavobacteriaceae;g__Aequorivita	8.82E-05	7.11E-06	3.80E-05	7.93E-06	0.005985	0.173312
f__Methanobacteriaceae;g__Methanosphaera	3.08E-05	8.43E-06	0.000604211	0.000121429	0.006023	0.173312
f__Thermoanaerobacteraceae;g__Thermacetogenium	1.82E-05	3.28E-06	1.96E-06	1.03E-06	0.006061	0.173312
f__Unclassified;g__Phocaeicola	3.87E-05	1.90E-06	1.78E-05	4.06E-06	0.006189	0.173312
f__Desulfovibrionaceae;g__Bilophila	8.59E-05	9.84E-06	3.23E-05	6.01E-06	0.006227	0.173312
f__Desulfobulbaceae;g__Desulfocapsa	1.77E-06	3.85E-07	0	0	0.006305	0.173312
f__Thermoanaerobacteraceae;g__Desulfovirgula	2.71E-06	2.75E-07	6.55E-07	3.58E-07	0.00643	0.173312
f__Rubricoccaceae;g__Rubrivirga	1.31E-07	1.31E-07	2.01E-06	3.92E-07	0.006468	0.173312
f__Thermodesulfobacteriaceae;g__Thermodesulfobacterium	1.04E-06	7.13E-07	4.47E-06	2.74E-07	0.006546	0.173312
f__Lentisphaeraceae;g__Lentisphaera	8.45E-06	8.88E-07	1.96E-06	1.15E-06	0.00663	0.173312
f__Ruminococcaceae;g__Bittarella	1.53E-05	1.25E-06	2.99E-05	3.05E-06	0.006668	0.173312
f__Glomeraceae;g__Rhizophagus	7.27E-07	6.48E-07	9.37E-06	1.85E-06	0.006706	0.173312
f__Erysipelotrichaceae;g__Turicibacter	0.000262125	9.87E-06	0.000123911	3.00E-05	0.00679	0.173312
f__Paenibacillaceae;g__Saccharibacillus	1.50E-05	1.44E-06	3.03E-05	3.19E-06	0.006828	0.173312
f__Synergistaceae;g__Aminiphilus	3.68E-07	3.68E-07	3.05E-06	4.95E-07	0.006911	0.173312
f__Hyellaceae;g__Pleurocapsa	1.25E-06	2.91E-07	0	0	0.007275	0.179495
f__Holosporaceae;g__Holospora	1.74E-06	4.08E-07	1.22E-08	1.22E-08	0.007458	0.181091
f__Haloferacaceae;g__Halogranum	0	0	1.09E-06	2.67E-07	0.007887	0.18497
f__Coxiellaceae;g__Candidatus Berkiella	1.74E-06	4.26E-07	0	0	0.007968	0.18497
f__Desulfovibrionaceae;g__Mailhella	0.001236471	0.000280725	8.83E-05	4.24E-05	0.008253	0.18497
f__Marinilabiliaceae;g__Labilibacter	0.000104741	4.11E-06	4.30E-05	1.48E-05	0.00833	0.18497
f__Sphingomonadaceae;g__Sphingorhabdus	8.47E-07	1.93E-07	4.70E-08	4.70E-08	0.008368	0.18497
f__Unclassified;g__Candidatus Planktophila	1.61E-06	3.96E-07	3.67E-08	3.67E-08	0.008626	0.18497
f__Dysgonamonadaceae;g__Dysgonomonas	0.001275463	0.000115199	0.00061779	0.000121893	0.008878	0.18497
f__Flavobacteriaceae;g__Psychroserpens	6.12E-05	1.20E-05	9.88E-06	5.36E-06	0.009004	0.18497
f__Cytophagaceae;g__Dyadobacter	9.38E-05	6.77E-06	5.49E-05	7.49E-06	0.009353	0.18497
f__Flavobacteriaceae;g__Arenibacter	6.39E-05	1.07E-05	1.92E-05	4.53E-06	0.009391	0.18497
f__Cytophagaceae;g__Flexibacter	2.36E-05	4.19E-06	4.83E-06	2.53E-06	0.009429	0.18497
f__Rikenellaceae;g__Rikenella	0.000174023	1.66E-05	6.61E-05	2.27E-05	0.009467	0.18497
f__Unclassified;g__Chloracidobacterium	1.94E-06	5.07E-07	0	0	0.009551	0.18497
f__Rhizobiaceae;g__Agrobacterium	3.60E-06	1.13E-06	1.25E-05	2.06E-06	0.009632	0.18497
f__Desulfobacteraceae;g__Desulfatibacillum	1.19E-05	1.16E-06	3.68E-06	1.85E-06	0.009856	0.18497
f__Ectothiorhodospiraceae;g__Ectothiorhodospira	1.20E-06	3.19E-07	0	0	0.010072	0.18497
f__Desulfovibrionaceae;g__Desulfovibrio	0.000180012	5.44E-06	8.99E-05	2.34E-05	0.0102	0.18497
f__Leptotrichiaceae;g__Leptotrichia	5.51E-05	1.98E-06	4.48E-05	1.90E-06	0.010321	0.18497
f__Porphyromonadaceae;g__Sanguibacteroides	7.13E-05	8.81E-06	2.63E-05	8.29E-06	0.010446	0.18497
f__Myxococcaceae;g__Myxococcus	1.90E-06	8.96E-07	6.37E-06	8.10E-07	0.010622	0.18497
f__Nitrospiraceae;g__Candidatus Magnetobacterium	6.69E-07	4.22E-07	9.09E-06	2.25E-06	0.010756	0.18497
f__Flammeovirgaceae;g__Fulvivirga	4.61E-05	8.67E-06	9.76E-06	4.72E-06	0.010795	0.18497
f__Hymenobacteraceae;g__Pontibacter	8.72E-05	1.27E-05	3.89E-05	3.48E-06	0.010876	0.18497
f__Methylococcaceae;g__Methyloglobulus	2.31E-06	5.20E-07	3.05E-07	1.72E-07	0.010914	0.18497
f__Flavobacteriaceae;g__Psychroflexus	2.71E-05	2.28E-06	1.20E-05	3.42E-06	0.010952	0.18497
f__Rhodobacteraceae;g__Phaeobacter	7.49E-06	1.95E-06	3.67E-07	1.11E-07	0.01099	0.18497
f__Rikenellaceae;g__Millionella	0.00022548	1.08E-05	0.000107488	3.07E-05	0.011198	0.18497
f__Chitinophagaceae;g__Niabella	9.52E-05	1.70E-05	2.71E-05	8.08E-06	0.011363	0.18497
f__Archaeoglobaceae;g__Archaeoglobus	9.48E-07	4.87E-07	3.31E-06	4.43E-07	0.011633	0.18497
f__Saccharospirillaceae;g__Reinekea	1.53E-06	2.93E-07	3.02E-07	1.82E-07	0.011759	0.18497
f__Halomonadaceae;g__Candidatus Carsonella	1.27E-06	3.34E-07	5.94E-08	5.94E-08	0.011797	0.18497
f__Unclassified;g__Thermosulfidibacter	0	0	2.35E-06	6.60E-07	0.011888	0.18497
f__Chitinophagaceae;g__Flavisolibacter	2.00E-05	4.56E-06	3.03E-06	1.36E-06	0.011926	0.18497
f__Flavobacteriaceae;g__Algibacter	1.49E-05	2.76E-06	4.80E-06	6.38E-07	0.011964	0.18497
f__Lachnospiraceae;g__Eisenbergiella	0.00017781	9.57E-06	6.82E-05	2.93E-05	0.012003	0.18497
f__Piscirickettsiaceae;g__Sulfurivirga	3.61E-06	8.94E-07	3.91E-07	1.33E-07	0.012041	0.18497
f__Cyclobacteriaceae;g__Cyclobacterium	5.06E-05	3.16E-06	2.02E-05	7.96E-06	0.012248	0.18497
f__Thermoanaerobacteraceae;g__Moorella	1.75E-05	1.44E-06	2.94E-05	3.04E-06	0.012329	0.18497
f__Erysipelotrichaceae;g__Merdibacter	3.61E-05	3.72E-06	1.20E-05	5.72E-06	0.012413	0.18497
f__Flavobacteriaceae;g__Aquimarina	7.17E-05	9.03E-06	3.00E-05	7.67E-06	0.012495	0.18497

f__Rhodobiaceae ;g__Tepidicaulis	2.77E-06	4.02E-07	7.93E-07	3.97E-07	0.012575	0.18497
f__Balneolaceae ;g__Gracilimonas	4.93E-08	4.93E-08	1.94E-06	5.43E-07	0.013	0.188797
f__Chromobacteriaceae ;g__Jeongeupia	6.42E-06	1.67E-06	5.60E-07	2.42E-07	0.013082	0.188797
f__Methylococcaceae ;g__Methylosarcina	1.81E-06	5.28E-07	0	0	0.013527	0.190344
f__Eubacteriaceae ;g__Anaerofustis	5.21E-05	2.16E-06	0.000110387	1.69E-05	0.013565	0.190344
f__Acidaminococcaceae ;g__Succinispira	7.37E-06	1.63E-06	1.49E-06	5.49E-07	0.013649	0.190344
f__Bacillaceae ;g__Salipaludibacillus	4.98E-07	4.98E-07	6.21E-06	1.60E-06	0.013687	0.190344
f__Selenomonadaceae ;g__Anaerovibrio	3.52E-05	7.69E-06	6.18E-05	1.38E-06	0.013853	0.190916
f__Prolixibacteraceae ;g__Draconibacterium	0.000214685	3.11E-05	8.68E-05	2.12E-05	0.014015	0.191424
f__Ruminococcaceae ;g__Agathobaculum	0.000265862	1.32E-05	0.000110439	4.41E-05	0.014223	0.191942
f__Rhodospirillaceae ;g__Oceanibaculum	6.10E-06	3.78E-07	2.07E-06	1.13E-06	0.014304	0.191942
f__Erwiniaceae ;g__Phaseolibacter	0	0	7.18E-07	2.15E-07	0.014576	0.193899
f__Debaryomycetaceae ;g__Yamadazyma	3.30E-07	9.95E-08	0	0	0.014962	0.19425
f__Micromonosporaceae ;g__Catenuloplanes	1.28E-06	8.19E-07	6.13E-06	1.22E-06	0.015135	0.19425
f__Unclassified;g__Leptothrix	1.43E-06	4.34E-07	0	0	0.015265	0.19425
f__Opitutaceae ;g__Cephaloticoccus	2.92E-06	8.01E-07	2.40E-07	1.62E-07	0.01539	0.19425
f__Barnesiellaceae ;g__Barnesiella	0.001874033	0.000190114	0.000808768	0.000262859	0.015481	0.19425
f__Lactobacillaceae ;g__Pediococcus	3.08E-06	9.41E-07	1.43E-05	3.29E-06	0.015519	0.19425
f__Nostocaceae ;g__Richelia	1.53E-06	4.70E-07	0	0	0.015937	0.19425
f__Myoviridae ;g__Cp8virus	1.31E-06	4.03E-07	0	0	0.015975	0.19425
f__Desulfobacteraceae ;g__Desulfococcus	1.23E-05	3.33E-06	1.29E-06	6.74E-07	0.016102	0.19425
f__Cytophagaceae ;g__Emticicia	4.07E-05	9.04E-06	1.07E-05	2.27E-06	0.01614	0.19425
f__Acetobacteraceae ;g__Gluconobacter	1.44E-06	4.49E-07	0	0	0.016179	0.19425
f__Carnobacteriaceae ;g__Alkalibacterium	1.81E-05	1.25E-06	2.75E-05	2.63E-06	0.016217	0.19425
f__Cyclobacteriaceae ;g__Echinicola	1.64E-05	2.60E-06	5.44E-06	2.23E-06	0.016297	0.19425
f__Enterococcaceae ;g__Vagococcus	5.08E-06	1.27E-06	6.40E-07	5.64E-07	0.016423	0.19425
f__Cyclobacteriaceae ;g__Algoriphagus	0.000140151	1.42E-05	5.90E-05	2.12E-05	0.016507	0.19425
f__Enterococcaceae ;g__Enterococcus	0.0002502	2.31E-06	0.000453796	6.41E-05	0.016818	0.196331
f__Selenomonadaceae ;g__Selenomonas	0.000316382	8.93E-06	0.000680203	0.000114873	0.016987	0.196331
f__Porphyromonadaceae ;g__Petrimonas	0.000216136	1.19E-05	9.67E-05	3.60E-05	0.017069	0.196331
f__Methanobacteriaceae ;g__Methanobacterium	3.26E-05	7.20E-06	9.74E-05	1.94E-05	0.017243	0.19685
f__Odoribacteraceae ;g__Butyricimonas	0.001901956	9.56E-05	0.000952319	0.000290101	0.017639	0.198801
f__Flavobacteriaceae ;g__Cloacibacterium	3.00E-05	7.74E-06	5.36E-06	2.15E-06	0.018164	0.198801
f__Desulfohalobiaceae ;g__Desulfonatronovibrio	9.88E-06	1.70E-06	4.23E-06	7.20E-07	0.018244	0.198801
f__Sneathiellaceae ;g__Sneathiella	1.33E-06	4.34E-07	0	0	0.018282	0.198801
f__Flavobacteriaceae ;g__Salegentibacter	3.59E-05	6.02E-06	1.32E-05	4.39E-06	0.018366	0.198801
f__Methanoregulaceae ;g__Methanosphaerula	1.79E-06	5.89E-07	0	0	0.01849	0.198801
f__Neisseriaceae ;g__Alysiella	1.53E-06	4.39E-07	1.69E-07	9.64E-08	0.018581	0.198801
f__Hyphomonadaceae ;g__Maricaulis	1.39E-07	1.39E-07	1.37E-06	3.84E-07	0.018711	0.198801
f__Peptostreptococcaceae ;g__Clostridioides	0.000170339	1.12E-05	0.000337173	5.41E-05	0.018749	0.198801
f__Rikenellaceae ;g__Mucinivorans	0.000426894	4.12E-05	0.000212596	5.85E-05	0.019033	0.198801
f__Flavobacteriaceae ;g__Spongiibacterium	3.53E-07	3.53E-07	2.03E-06	4.38E-07	0.019117	0.198801
f__Pasteurellaceae ;g__Rodentibacter	1.72E-05	3.74E-06	5.23E-06	1.41E-06	0.019155	0.198801
f__Marinilabiliaceae ;g__Saccharicrinis	2.55E-05	3.07E-06	1.04E-05	3.99E-06	0.019194	0.198801
f__Prolixibacteraceae ;g__Sunxiuqinia	0.000120171	1.64E-05	5.41E-05	1.51E-05	0.019535	0.198801
f__Sclerotiniaceae ;g__Botrytis	0	0	1.00E-05	3.38E-06	0.019573	0.198801
f__Lactobacillaceae ;g__Lactobacillus	0.000240399	3.21E-06	0.000438868	6.69E-05	0.019612	0.198801
f__Crocinitomicaceae ;g__Crocinitomix	3.55E-05	7.86E-06	9.15E-06	4.17E-06	0.019909	0.198801
f__Clostridiaceae ;g__Clostridiisalibacter	3.72E-05	1.05E-06	1.58E-05	7.17E-06	0.020213	0.198801
f__Carnobacteriaceae ;g__Desemzia	1.11E-06	4.43E-07	3.59E-06	7.22E-07	0.020293	0.198801
f__Unclassified;g__Hydrogenimonas	1.49E-06	5.09E-07	0	0	0.020331	0.198801
f__Cyclobacteriaceae ;g__Belliella	1.56E-05	2.67E-06	5.48E-06	2.19E-06	0.020415	0.198801
f__Flavobacteriaceae ;g__Mesonia	2.09E-05	5.49E-06	4.53E-06	1.22E-06	0.020683	0.198801
f__Tannerellaceae ;g__Parabacteroides	0.007361119	0.000647167	0.003581468	0.001127887	0.020893	0.198801
f__Actinomycetaceae ;g__Varibaculum	3.44E-05	4.24E-06	1.51E-05	5.16E-06	0.021287	0.198801
f__Flavobacteriaceae ;g__Tenacibaculum	6.25E-05	4.94E-06	2.93E-05	1.04E-05	0.021371	0.198801
f__Hyphomonadaceae ;g__Robiginitomaculum	2.85E-06	8.73E-07	7.16E-06	1.22E-06	0.021409	0.198801
f__Porphyromonadaceae ;g__Porphyromonas	0.001197301	9.51E-05	0.000615863	0.000178246	0.021447	0.198801
f__Odoribacteraceae ;g__Odoribacter	0.001483515	0.000171947	0.000713218	0.000205255	0.021531	0.198801
f__Spirochaetaceae ;g__Marispirochaeta	1.70E-05	2.58E-06	6.49E-06	2.60E-06	0.021657	0.198801
f__Gemmataceae ;g__Zavarzinella	9.55E-07	4.78E-07	4.39E-06	1.10E-06	0.021737	0.198801
f__Unclassified;g__Candidatus Symbiothrix	0.000242518	7.05E-06	0.000157629	2.87E-05	0.021775	0.198801
f__Sporomusaceae ;g__Pelosinus	1.89E-05	2.78E-06	4.02E-05	6.89E-06	0.021859	0.198801
f__Vibrionaceae ;g__Grimontia	2.63E-06	9.17E-07	0	0	0.022029	0.198801
f__Pseudonocardiaceae ;g__Actinokineospora	2.22E-07	2.22E-07	3.16E-06	1.00E-06	0.022067	0.198801
f__Sporomusaceae ;g__Anaeromusa	2.29E-05	4.73E-06	6.64E-06	3.17E-06	0.022144	0.198801
f__Methylophilaceae ;g__Methylophilus	1.73E-06	4.81E-07	2.21E-07	2.21E-07	0.022225	0.198801
f__Schizoporaceae ;g__Schizopora	4.08E-06	1.14E-06	6.88E-07	3.46E-07	0.022362	0.198801
f__Flavobacteriaceae ;g__Capnocytophaga	0.000292611	2.83E-05	0.000171319	3.18E-05	0.022485	0.198801
f__Neisseriaceae ;g__Amantichitinum	1.02E-06	3.04E-07	1.08E-07	1.08E-07	0.022696	0.198801
f__Siphoviridae ;g__Sextaecvirus	1.08E-06	8.29E-07	5.56E-06	1.35E-06	0.022823	0.198801
f__Synechococcaceae ;g__Dactylococcopsis	1.71E-06	5.89E-07	3.93E-08	3.93E-08	0.022905	0.198801
f__Myoviridae ;g__Msw3virus	2.50E-08	2.50E-08	2.51E-06	8.78E-07	0.022943	0.198801
f__Deferribacteraceae ;g__Geovibrio	4.37E-06	1.20E-06	7.36E-07	4.75E-07	0.023021	0.198801
f__Alteromonadaceae ;g__Alteromonas	6.86E-06	1.96E-07	1.33E-05	2.28E-06	0.023194	0.198801
f__Choanephoraceae ;g__Choanephora	0	0	1.74E-06	6.22E-07	0.023321	0.198801
f__Sporomusaceae ;g__Anaerosporomusa	1.69E-05	2.05E-06	6.54E-06	3.09E-06	0.023451	0.198801
f__Methanosarcinaceae ;g__Methanolobus	4.47E-06	7.21E-07	2.00E-06	5.09E-07	0.023529	0.198801
f__Unclassified;g__Candidatus Izimaplasma	7.76E-05	1.85E-05	0.000166519	2.60E-05	0.023699	0.198801
f__Peptoniphilaceae ;g__Anaerococcus	2.79E-05	4.62E-06	4.25E-05	2.54E-06	0.023782	0.198801
f__Chlorobiaceae ;g__Chlorobaculum	8.95E-06	2.75E-06	1.26E-06	4.00E-07	0.024132	0.200633
f__Hyphomicrobiaceae ;g__Cucumibacter	0	0	6.88E-07	2.50E-07	0.024526	0.202393
f__Rhodobacteraceae;g__Maribius	1.62E-06	5.90E-07	0	0	0.024608	0.202393
f__Unclassified;g__Candidatus Vecturithrix	1.05E-05	2.05E-06	1.79E-05	1.76E-06	0.024917	0.203061
f__Halobacteriaceae ;g__Haladaptatus	1.01E-06	5.11E-07	3.10E-06	5.65E-07	0.024955	0.203061
f__Sphingobacteriaceae ;g__Solitalea	5.69E-05	1.76E-05	7.92E-06	3.90E-06	0.02526	0.203684
f__Flavobacteriaceae ;g__Galbibacter	5.95E-06	1.48E-06	1.26E-06	8.94E-07	0.025298	0.203684
f__Porphyromonadaceae ;g__Lascolabacillus	7.99E-05	1.80E-05	2.72E-05	7.41E-06	0.025589	0.204949
f__Leptospiraceae ;g__Turneriella	1.50E-06	2.03E-08	6.26E-07	3.26E-07	0.026118	0.206564
f__Eggerthellaceae ;g__Gordonibacter	4.92E-05	8.57E-06	9.01E-05	1.26E-05	0.026156	0.206564
f__Legionellaceae ;g__Legionella	1.55E-05	2.97E-06	6.77E-06	1.31E-06	0.026331	0.206564
f__Eggerthellaceae ;g__Cryptobacterium	4.13E-06	8.02E-07	1.72E-05	4.86E-06	0.026784	0.206564
f__Flavobacteriaceae ;g__Gaetbulibacter	8.76E-06	1.83E-06	2.88E-06	1.25E-06	0.026911	0.206564
f__Prevotellaceae ;g__Prevotella	0.040816396	0.00162042	0.023349739	0.00640132	0.027119	0.206564
f__Desulfobacteraceae ;g__Candidatus Magnetomorum	9.01E-06	2.83E-06	1.33E-06	6.85E-07	0.02729	0.206564
f__Bacillaceae ;g__Amphibacillus	1.03E-05	3.78E-06	2.05E-05	7.31E-07	0.027371	0.206564
f__Prolixibacteraceae ;g__Prolixibacter	8.51E-05	2.14E-05	2.47E-05	8.35E-06	0.027494	0.206564
f__Flavobacteriaceae ;g__Wenyingzhuangia	1.74E-05	2.70E-06	8.82E-06	1.87E-06	0.027532	0.206564
f__Thermodesulfobacteriaceae ;g__Thermosulfurimonas	5.08E-06	1.40E-06	1.39E-06	6.70E-08	0.027708	0.206564
f__Unclassified;g__Pseudohongiella	1.13E-06	4.30E-07	0	0	0.027746	0.206564
f__Porphyromonadaceae ;g__Fermentimonas	9.40E-05	1.27E-05	4.39E-05	1.43E-05	0.027784	0.206564
f__Colwelliaceae ;g__Thalassotalea	2.99E-06	1.01E-06	2.82E-07	2.17E-07	0.027875	0.206564
f__Flavobacteriaceae ;g__Lutibacter	9.39E-05	1.61E-05	4.25E-05	1.12E-05	0.027913	0.206564
f__Coleofasciculaceae ;g__Geitlerinema	6.70E-07	2.56E-07	0	0	0.027951	0.206564
f__Solibacteraceae ;g__Candidatus Solibacter	8.64E-07	2.08E-07	2.45E-06	5.74E-07	0.028456	0.209281
f__Peptostreptococcaceae ;g__Paeniclostridium	6.86E-05	1.34E-06	0.000225249	6.02E-05	0.028684	0.20995
f__Cryomorphaceae ;g__Owenweeksia	3.79E-05	7.81E-06	1.49E-05	4.14E-06	0.028859	0.210226

f__Rhodobacteraceae;g__Pannonibacter	1.18E-06	3.57E-07	1.64E-07	1.64E-07	0.029124	0.211149
f__Methanomicrobiaceae;g__Methanofollis	2.98E-06	1.06E-06	2.01E-07	2.01E-07	0.029306	0.211468
f__Bacillaceae;g__Bacillus	0.001816245	0.000396949	0.092243501	0.035257906	0.029609	0.212306
f__Haloferacaceae;g__Halobellus	1.43E-06	3.01E-07	2.98E-06	5.26E-07	0.0297	0.212306
f__Cytophagaceae;g__Runella	2.28E-05	3.08E-06	1.29E-05	2.34E-06	0.029871	0.212537
f__Synergistaceae;g__Aminomonas	3.57E-06	8.81E-07	9.22E-07	5.57E-07	0.030179	0.212951
f__Peptostreptococcaceae;g__Asaccharospora	7.65E-06	1.23E-06	1.78E-05	3.82E-06	0.030217	0.212951
f__Rickettsiaceae;g__Rickettsia	8.62E-06	8.24E-07	3.98E-06	1.63E-06	0.030347	0.212951
f__Chitinophagaceae;g__Sediminibacterium	1.51E-05	4.35E-06	3.65E-06	1.22E-06	0.0308	0.214742
f__Pasteurellaceae;g__Chelonobacter	4.18E-06	1.36E-06	6.01E-07	3.66E-07	0.030883	0.214742
f__Methylothermaceae;g__Methylohalobius	1.45E-06	5.83E-07	0	0	0.031895	0.220778
f__Rhodospirillaceae;g__Thalassospira	3.76E-05	7.04E-06	1.32E-05	6.83E-06	0.032113	0.221286
f__Yersiniaceae;g__Ewingella	5.14E-08	5.14E-08	7.11E-07	2.62E-07	0.032627	0.223411
f__Marinifilaceae;g__Marinifilum	6.96E-05	1.89E-05	1.96E-05	7.43E-06	0.032838	0.223411
f__Spirochaetaceae;g__Alkalispirochaeta	3.17E-06	4.85E-07	1.94E-06	1.26E-07	0.032922	0.223411
f__Bacteroidaceae;g__Bacteroides	0.133964951	0.010246608	0.071565603	0.02322556	0.033006	0.223411
f__Bacteroidaceae;g__Anaerorhabdus	0.000158152	2.58E-05	6.26E-05	2.91E-05	0.033174	0.22356
f__Bacillaceae;g__Salibacterium	2.23E-06	1.76E-06	1.11E-05	3.15E-06	0.033722	0.224783
f__Acidobacteriaceae;g__Acidobacterium	1.62E-06	1.87E-07	6.67E-07	3.40E-07	0.03376	0.224783
f__Oceanospirillaceae;g__Marinobacterium	1.23E-06	3.65E-07	3.99E-06	1.07E-06	0.033926	0.224783
f__Streptomycetaceae;g__Kitasatospora	2.24E-06	5.34E-07	6.79E-06	1.79E-06	0.034216	0.224783
f__Rhodobiaceae;g__Afifella	1.76E-06	4.18E-07	4.06E-07	3.71E-07	0.034518	0.224783
f__Methanomassiliicoccaceae;g__Candidatus Methanomethylophilus	4.79E-05	1.54E-05	8.98E-06	4.58E-06	0.034556	0.224783
f__Unclassified;g__Candidatus Azobacteroides	7.82E-06	2.63E-06	1.00E-06	1.00E-06	0.034731	0.224783
f__Tannerellaceae;g__Tannerella	0.000957134	0.000153259	0.000444847	0.000146239	0.034903	0.224783
f__Desulfomicrobiaceae;g__Desulfomicrobium	5.60E-06	1.91E-06	7.65E-07	6.02E-07	0.034941	0.224783
f__Unclassified;g__Fenollaria	5.57E-06	1.87E-06	8.87E-07	5.30E-07	0.035151	0.224783
f__Thiotrichaceae;g__Beggiatoa	8.22E-06	1.85E-06	3.36E-06	7.98E-07	0.035189	0.224783
f__Neisseriaceae;g__Snodgrassella	5.02E-06	1.02E-06	1.78E-06	8.76E-07	0.035227	0.224783
f__Succinivibrionaceae;g__Ruminobacter	2.68E-05	1.01E-06	1.37E-05	5.38E-06	0.035266	0.224783
f__Thermoanaerobacterales Family III. Incertae Sedis;g__Thermovenabulum	1.75E-06	9.02E-07	7.03E-06	2.02E-06	0.035842	0.226686
f__Fusobacteriaceae;g__Psychrilyobacter	2.73E-06	9.50E-07	4.53E-07	1.20E-07	0.035881	0.226686
f__Listeriaceae;g__Listeria	4.65E-05	5.59E-06	0.000145294	4.13E-05	0.036009	0.226686
f__Chamaesiphonaceae;g__Chamaesiphon	9.74E-07	4.12E-07	0	0	0.036312	0.227517
f__Hyphomicrobiaceae;g__Filomicrobium	2.54E-06	7.48E-07	6.19E-07	3.20E-07	0.036529	0.227517
f__Veillonellaceae;g__Veillonella	8.22E-05	8.05E-06	0.000287861	8.67E-05	0.036659	0.227517
f__Flavobacteriaceae;g__Seonamhaeicola	1.36E-05	3.80E-06	3.69E-06	1.85E-06	0.037025	0.227517
f__Balneolaceae;g__Aliifodinibius	7.72E-06	1.15E-06	3.06E-06	1.62E-06	0.037064	0.227517
f__Lachnospiraceae;g__Lachnoanaerobaculum	5.18E-05	5.41E-06	3.59E-05	4.06E-06	0.037102	0.227517
f__Rhodobacteraceae;g__Jannaschia	1.15E-06	3.36E-07	2.13E-07	2.13E-07	0.037182	0.227517
f__Rhodospirillaceae;g__Magnetospirillum	2.90E-05	6.81E-06	1.23E-05	2.12E-06	0.037348	0.227622
f__Cytophagaceae;g__Spirosoma	8.05E-05	1.03E-05	5.14E-05	6.99E-06	0.037986	0.230596
f__Thermoanaerobacterales Family III. Incertae Sedis;g__Thermosediminibacter	3.14E-06	6.70E-07	8.50E-06	2.20E-06	0.038293	0.23103
f__Ectothiorhodospiraceae;g__Halorhodospira	2.03E-06	1.80E-07	5.93E-07	5.93E-07	0.03842	0.23103
f__Ustilaginaceae;g__Ustilago	0	0	2.67E-06	1.15E-06	0.038511	0.23103
f__Bacillaceae;g__Anoxybacillus	3.13E-05	1.09E-05	0.00020641	7.50E-05	0.039082	0.233537
f__Flammeovirgaceae;g__Fabibacter	1.89E-05	6.46E-06	3.50E-06	1.75E-06	0.039255	0.233663
f__Natrialbaceae;g__Natronorubrum	0	0	6.37E-07	2.77E-07	0.039601	0.2347
f__Flavobacteriaceae;g__Bizionia	2.52E-05	9.35E-06	3.43E-06	1.49E-06	0.039809	0.2347
f__Sordariaceae;g__Sordaria	0	0	3.61E-06	1.58E-06	0.03989	0.2347
f__Clavicipitaceae;g__Metarhizium	6.87E-08	6.87E-08	9.21E-06	4.00E-06	0.04011	0.235089
f__Unclassified;g__Neofamilia	4.89E-06	3.34E-07	2.62E-06	9.39E-07	0.040288	0.23523
f__Unclassified;g__Ndongobacter	1.34E-05	1.83E-06	6.15E-06	2.60E-06	0.04055	0.235865
f__Pseudobacteriovoracaceae;g__Pseudobacteriovorax	7.01E-06	1.63E-06	2.30E-06	1.28E-06	0.041132	0.237901
f__Unclassified;g__Sulfurovum	1.03E-05	2.97E-06	3.15E-06	1.00E-06	0.041212	0.237901
f__Synergistaceae;g__Cloacibacillus	8.18E-05	7.98E-06	4.45E-05	1.45E-05	0.041608	0.23818
f__Chromatiaceae;g__Thiohalocapsa	1.70E-06	7.20E-07	7.31E-08	7.31E-08	0.041689	0.23818
f__Actinomycetaceae;g__Actinobaculum	0	0	2.73E-06	1.21E-06	0.041727	0.23818
f__Erysipelotrichaceae;g__Solobacterium	3.59E-05	3.63E-06	7.93E-05	1.90E-05	0.042124	0.238629
f__Cyclobacteriaceae;g__Lunatimonas	4.94E-06	1.27E-06	1.77E-06	6.18E-07	0.042162	0.238629
f__Bacillaceae;g__Terribacillus	7.45E-06	2.65E-06	6.08E-05	2.37E-05	0.042421	0.238629
f__Ruminococcaceae;g__Papillibacter	0.000307285	2.15E-05	0.000144862	6.93E-05	0.042504	0.238629
f__Aerococcaceae;g__Eremococcus	4.12E-06	4.84E-07	9.87E-06	2.52E-06	0.042586	0.238629
f__Kosmotogaceae;g__Kosmotoga	5.62E-06	1.02E-06	1.60E-06	1.49E-06	0.043155	0.23875
f__Coriobacteriaceae;g__Olegusella	2.28E-06	3.61E-07	1.35E-06	2.09E-07	0.043193	0.23875
f__Ruminococcaceae;g__Sporobacter	0.0006793	5.27E-05	0.000363547	0.000132284	0.043328	0.23875
f__Unclassified;g__Thermoflexibacter	6.04E-05	2.27E-05	9.13E-06	4.52E-06	0.043452	0.23875
f__Cytophagaceae;g__Ohtaekwangia	2.06E-05	6.95E-06	4.69E-06	1.77E-06	0.043578	0.23875
f__Selenomonadaceae;g__Centipeda	1.10E-06	7.26E-07	3.97E-06	1.08E-06	0.043802	0.23875
f__Enterobacteriaceae;g__Enterobacter	2.91E-06	6.57E-07	7.29E-06	1.87E-06	0.043928	0.23875
f__Tolypothrichaceae;g__Hassallia	2.21E-05	8.33E-06	3.49E-06	1.13E-06	0.044009	0.23875
f__Nitrosomonadaceae;g__Nitrosomonas	2.75E-05	8.26E-06	8.71E-06	2.12E-06	0.044345	0.23875
f__Streptococcaceae;g__Streptococcus	0.000266195	2.48E-05	0.000353067	3.07E-05	0.044384	0.23875
f__Flavobacteriaceae;g__Croceitalea	1.51E-06	6.60E-07	5.30E-08	5.30E-08	0.044475	0.23875
f__Paenibacillaceae;g__Cohnella	6.19E-05	1.26E-05	0.000124991	2.59E-05	0.044552	0.23875
f__Enterococcaceae;g__Bavariicoccus	5.59E-06	2.08E-06	8.93E-07	5.01E-07	0.044636	0.23875
f__Rhodobacteraceae;g__Donghicola	1.01E-06	4.45E-07	3.45E-08	3.45E-08	0.045145	0.239997
f__Bernardetiaceae;g__Hugenholtzia	3.78E-05	9.93E-06	1.34E-05	5.11E-06	0.045183	0.239997
f__Caldilineaceae;g__Litorilinea	2.57E-06	6.86E-07	7.97E-07	4.45E-07	0.045758	0.241631
f__Flavobacteriaceae;g__Bergeyella	2.03E-05	5.27E-06	6.80E-06	3.37E-06	0.045883	0.241631
f__Sordariaceae;g__Neurospora	0	0	8.84E-06	4.09E-06	0.045964	0.241631
f__Halococcaceae;g__Halococcus	4.89E-07	4.89E-07	2.29E-06	6.86E-07	0.046546	0.242391
f__Peptococcaceae;g__Desulfotomaculum	0.000122808	4.56E-06	0.000203325	3.73E-05	0.046628	0.242391
f__Rhodobacteraceae;g__Oceanicella	6.60E-07	2.57E-07	8.21E-08	8.21E-08	0.046709	0.242391
f__Ophiostomataceae;g__Sporothrix	0	0	4.86E-06	2.28E-06	0.046933	0.242391
f__Ruminococcaceae;g__Massilimaliae	0.000563335	3.60E-05	0.000284571	0.000125872	0.047054	0.242391
f__Rickettsiaceae;g__Orientia	9.93E-07	4.66E-07	0	0	0.047134	0.242391
f__Kosmotogaceae;g__Mesotoga	9.05E-06	5.51E-07	5.94E-06	1.36E-06	0.047218	0.242391
f__Flavobacteriaceae;g__Olleya	7.57E-06	3.08E-06	9.67E-07	4.20E-07	0.04743	0.242663
f__Rhizobiaceae;g__Neorhizobium	0	0	9.48E-07	4.48E-07	0.047691	0.242963
f__Propionibacteriaceae;g__Acidipropionibacterium	1.11E-06	5.18E-07	5.12E-06	1.82E-06	0.047864	0.242963
f__Alcaligenaceae;g__Pelistega	1.23E-06	2.67E-07	3.89E-07	2.95E-07	0.048086	0.242963
f__Gemmataceae;g__Gemmata	1.46E-05	3.57E-06	5.56E-06	2.34E-06	0.048124	0.242963
f__Colwelliaceae;g__Thalassomonas	3.97E-08	3.97E-08	2.64E-06	1.24E-06	0.048622	0.244671
f__Rhodobacteraceae;g__Litoreibacter	3.40E-06	9.67E-07	1.03E-06	5.92E-07	0.049735	0.249227
f__Gallionellaceae;g__Gallionella	9.92E-07	4.76E-07	0	0	0.049859	0.249227

Supplemental Table S8: All fecal metabolites detected by LC-MS/MS

Compound ID	Name	Other_name(Kegg_name)	Formula	Molecular weight	RT [min]	m/z	All_C1	All_C2	All_C3	All_C4	All_C5	All_C6	All_W1	All_W2	All_W3	All_W4	All_W5	All_W6	All_QC1	All_QC2	All_QC3
Com_34_neg	Dodecanedioic acid	Dodecanedioic acid	C12 H22 O4	230.15142	6.761	229.14404	3127816623	2932553900	2179213835	2129166825	2636019583	2397892464	890192961.3	2321712817	739409300.5	871128999.9	511398889.1	2435500729	1720356019	2013139574	2008701846
Com_37_neg	Cholic acid	Cholic acid; Cholate; 3alpha,7alpha,12alpha-Trihydroxy-5beta-cholanate; 3alpha,7alpha,12alpha-Trihydroxy-5beta-cholanic	C24 H40 O5	408.28712	7.586	407.27954	578743763.1	420719251.8	1001977187	1257836216	522675394.1	581788967.2	943898045.9	1224566231	763026721.2	1807327223	1358395490	1259114549	939552645.1	948751360.5	886653075.8
Com_145_neg	α,α-Trehalose	alpha,alpha-Trehalose; alpha,alpha'-Trehalose; Trehalose	C12 H22 O11	342.1159	1.366	341.1087	191345632.1	77428386.19	159785842	101177483.8	264456137.4	305718943.1	174246278.1	482312985	428855167.4	120683982	617703534.5	253429637.3	198747495	262761500.6	234000162.7
Com_203_neg	DL-α-Methoxyphenylacetic	--	C9 H10 O3	166.06272	5.701	165.05536	15519285.13	21309359.77	20366961.13	40037283.59	18283332.75	20783464.33	524239749.9	16938999.44	78729666.33	80053509.98	29345184.39	25139079.08	47875341.87	47326543.43	47810393.31
Com_228_neg	Sucrose	Sucrose; Cane sugar; Saccharose; 1-alpha-D-Glucopyranosyl-2-beta-D-fructofuranoside	C12 H22 O11	342.116	1.458	341.10855	12072949.77	4292920.391	12684571.52	7163789.233	17401627.11	17545978.62	63740259.55	24407807.3	129667461	7261788.183	401300972.1	43718916.57	17364855.19	16062680.56	17264561.05
Com_279_neg	Salicylic acid	Salicylate; o-Hydroxybenzoic acid;	C7 H6 O3	138.03149	5.551	137.02429	175404315.3	161665417	117942041.2	105603852.6	158199016.4	122905899.8	99357109.97	111666992.4	285919658.4	279635695.5	153021406.2	122457723.5	143217522.2	142451809.5	143581281.8
Com_306_neg	Adipic acid	Adipate; Adipic acid; Hexanedioate; Hexan-1,6-dicarboxylate	C6 H10 O4	146.05772	5.236	145.05049	85109850.06	88214927.11	149424635.8	165739303.6	84275737.78	90898421.81	289385023.6	224837635.1	226197805.5	189257556	300312736.9	37632728.47	169146874.3	176689076.9	171643914.1
Com_319_neg	3-Methyladipic acid	--	C7 H12 O4	160.0733	5.528	159.06627	222544959.6	204269003.6	173959509.5	185030327.7	221480873.1	216097645.1	179830954.6	123565848.1	203423726.4	174167313	287444038.5	135187739.6	168471891.5	185310621.8	172771089.8
Com_394_neg	D-(-)-Quinic acid	--	C7 H12 O6	192.06317	1.462	191.05579	75468256.02	53912326.06	90178239.25	139323667.6	160333119.1	99376957.12	133501677.9	239481231.1	107329539	171260786.6	158960953.3	167602531.7	90602256.41	96307671.34	135647554.2
Com_473_neg	3,3-Dimethylglutaric acid	--	C7 H12 O4	160.07322	5.748	159.06586	85626660.18	90009230.05	65302554.37	65655479.37	78855450.61	73506498.47	74448302.48	180265567.6	149033478	132487090.2	57534211.13	195618474.9	85035399.5	85220963.56	77829405.43
Com_588_neg	Suberic acid	Suberic acid; Cork acid; 1,8-Octanedioic acid; Octanedioic acid; 1,6-Hexanedicarboxylic acid	C8 H14 O4	174.08886	5.767	173.0815	188565396.2	172377546.4	79015458.4	82808959.42	161387337.9	159950502.5	63882088.91	39576742.01	72192468.74	60977388.87	95421738.89	49615769.16	98020611.42	105450865.4	106003573
Com_595_neg	Glutaric acid	Glutarate; Glutaric acid; Pentanedioic acid; 1,3-Propanedicarboxylic acid	C5 H8 O4	132.04206	4.712	131.03496	24513229.72	14955621.06	115062533.8	159764073.4	102956344.4	50940601.63	113714966.6	35747927.38	44249027.65	41787024.38	52544542.33	87060449.65	89933449.58	92877405.56	70407289.62
Com_638_neg	L-Glutamic acid	L-Glutamate; L-Glutamic acid; L-Glutaminic acid;	C5 H9 N O4	147.05293	1.309	146.0455	46602157.68	60057299.41	53906337.13	55273884.05	89757817.24	84271024.97	34005911.08	123228948.8	53761709.08	111432737.7	31522895.42	100954114.2	64702687.5	89633579.5	78233751.4
Com_717_neg	Methylsuccinic acid	--	C5 H8 O4	132.04205	5.032	131.03481	48476953.01	38991075.93	54273813.34	41395129.02	43928144.64	49951723.02	55875599.38	71875875.71	87495512.47	95656426.62	55352324.98	79947558.74	46482737.01	47976470.59	50942044.12
Com_721_neg	D-(-)-Mannitol	--	C6 H14 O6	182.07882	1.328	181.07153	282974.3171	286214.0669	3928192.542	872592.6178	20260843.17	1018655.484	22115809.62	36394873.56	34838049.55	22753167.51	116387676.3	35311808.09	26639269.22	32744288.32	31210885.47
Com_744_neg	Uridine	Uridine	C9 H12 N2 O6	244.06927	2.315	243.06204	52546933.27	73424952.11	61322555.22	34029189.99	144611655.9	82926610.34	2500887.91	9444245.298	13856710.27	7294881.538	5022335.281	13946500.66	22299190.7	20813247.26	22682575.99
Com_828_neg	3,5-Dihydroxybenzoic acid	--	C7 H6 O4	154.02643	7.443	153.01903	97818880.2	149391083.4	77021135.24	75994480.85	78256609.57	95991665.87	20252179.31	19640064.36	44590780.28	66255465.49	7294667.841	69576695.84	48661272.11	50507617.88	57077629.44
Com_973_neg	Resveratrol	Resveratrol; 3,4',5-Trihydroxystilbene; trans-Resveratrol	C14 H12 O3	228.07842	5.906	227.07112	82751454.44	86364391.65	87962657.19	93159557.34	75590578.65	78457895.59	52636612.02	32381875.61	36813808.01	37760675.45	36357038.71	31351183.05	60396827.14	66121503.65	60185558.6
Com_1153_neg	Homovanillic acid	Homovanillate; Homovanillic acid; 3-Methoxy-4-hydroxyphenylacetate	C9 H10 O4	182.05773	5.235	181.05038	60041253.53	77900352.39	88036439.64	73095549.2	47205022.27	51034132.35	23428358.75	33798339.48	30686675	29780621.1	39624000.61	20784870.31	48056345.38	51090626.71	51877517.42
Com_1207_neg	2-Hydroxyphenylacetic acid	2-Hydroxyphenylacetate; 2-Hydroxyphenylacetic acid	C8 H8 O3	152.04704	5.478	151.03978	5667469.649	6909195.14	6134270.434	6614160.987	5407482.373	5163646.068	20408883.31	48829479.88	59598064.33	55650862.07	18126405.78	54598363.98	20828233.43	35332837.37	30751357.82
Com_1296_neg	3-Methylglutaric acid	--	C6 H10 O4	146.05771	5.368	145.05032	68937603.17	60398965.6	47988207.62	52608044.95	71443992.45	51332876.2	70908417.94	64898050.06	58240129.68	51589238.68	58233362.3	59052349.97	43404009.65	46061587.44	50939711.31
Com_1320_neg	β-D-Glucopyranuronic acid	beta-D-Glucopyranuronic acid; beta-D-Glucuronic acid	C6 H10 O7	194.04228	1.474	193.03491	4100619.677	5168591.972	6273937.8	5411268.665	8865581.055	6405916.049	9452995.215	63900699.8	7386426.002	1939676.255	7261297.391	39010214.97	9112000.046	10343849.4	8574434.789
Com_1386_neg	D-α-Hydroxyglutaric acid	--	C5 H8 O5	148.0371	1.434	147.02994	30187271.43	26084404.52	36303102.95	33435035.41	73891701.02	35276031.85	9947412.594	12550492.75	7826310.515	13382116.92	9156929.951	9478897.425	20597374.29	22055588.34	20688867.1
Com_1393_neg	3-Hydroxybenzoic acid	3-Hydroxybenzoate; 3-Hydroxybenzoic acid; m-Hydroxybenzoic acid	C7 H6 O3	138.03142	5.402	137.02429	28473340.1	31540865.14	33595402.16	40276757.44	40375777.68	32136403.02	30795368.37	25588103.63	32746234.49	46839258.23	20290291.09	45153236.52	33429438.34	35838298.86	34504656.57
Com_1396_neg	δ-Ribono-1,4-lactone	--	C5 H8 O5	148.03698	2.077	147.02962	19037447.67	21794151.04	7371815.044	40579487.94	72975011.5	36612576.85	7580359.948	8226915.006	4638993.683	16367642.47	5727079.541	6618528.486	21074996.67	23193441.91	18717192.01
Com_1513_neg	(±)12(13)-DiHOME	--	C18 H34 O4	314.24528	8.781	313.23807	15341097.71	14197293.29	16779927.94	30580422.97	10278560.51	7298906.987	41115747.57	20823107.25	37395873.04	26865301.44	51894698.15	23819545	29090996.13	21710381.66	28395614.93
Com_1664_neg	2,5-Dihydroxybenzaldehyde	Gentisate aldehyde; 2,5-Dihydroxybenzaldehyde	C7 H6 O3	138.03144	5.303	137.02419	17857603.93	27092994.31	25006573.76	19390925.91	19824507.63	23061800.28	43662121.54	45670814.34	50135876.55	29472907.07	42681533.85	35178468.7	18077593.75	27978538.47	25610700.36
Com_1850_neg	2-Anisic acid	--	C8 H8 O3	152.047	5.163	151.03973	10919307.42	11110950.68	15741854.26	13580684.96	17310395.11	14604869.52	9211574.158	12626189.45	11366236.23	13840546.13	41285481.03	9093259.257	10572674.53	11860883.83	12940619.88
Com_1873_neg	DL-Malic acid	--	C4 H6 O5	134.02137	1.543	133.01407	6585134.043	6097319.983	9180433.949	8833354.182	12645118.54	11267937.59	3805802.574	41966043.18	8957184.725	23166298.05	7216643.347	17802184.61	11025764.08	11666332.97	10011956.48
Com_1925_neg	3-Methoxyphenylacetic acid	--	C9 H10 O3	166.06274	5.225	165.05536	27791248.38	17250623.16	11698691.44	34687548.49	8090580.758	9898926.803	46073517.38	10361017.96	13709889.83	14442821.34	13050974.57	14044726.26	13874274.07	12758562.63	12091255.07
Com_2138_neg	D-Saccharic acid	D-Glucarate; D-Glucaric acid; L-Gularic acid; D-Saccharic acid; D-Glucosaccharic acid; Glucaric	C6 H10 O8	210.03734	1.514	209.03001	19603679.3	14096374.17	31253977.5	25578942.46	44125341.83	28605909.17	2182179.446	11838993.43	3463080.461	5520028.043	9641842.308	8884602.986	13038186.14	18985254.59	16694801.49
Com_2421_neg	L-Aspartic acid	L-Aspartate; L-Aspartic acid; 2-Aminosuccinic acid; L-Asp	C4 H7 N O4	133.03737	1.321	132.0302	2553229.251	3349955.641	4869165.407	5876478.386	4908948.396	4870966.29	3642100.292	30634572.96	9093386.744	7384453.981	3057129.208	15482516.43	5181690.732	6740763.469	5475441.703
Com_2452_neg	2-Isopropylmalic acid	alpha-Isopropylmalate; (2S)-2-Isopropylmalate; (2S)-2-Hydroxy-2-isopropylsuccinic acid; 2-Isopropylmalic acid; 3-Carboxy-3-hydroxy-4-methylpentanoate; 3-Carboxy-3-	C7 H12 O5	176.06824	4.87	175.061	32530695.92	42517390.61	29560148.08	12260533.18	37031251.02	12306505.49	6868404.693	11469357.42	6873329.51	10089911.74	8838392.759	13226483.66	7674903.702	9090552.487	7093223.27
Com_2505_neg	Citric acid	Citrate; Citric acid; 2-Hydroxy-1,2,3-propanetricarboxylic acid; 2-Hydroxytricarballylic acid	C6 H8 O7	192.02688	2.233	191.01961	20104358.64	13467019.94	20801669.74	21515091.3	36369018.11	28525156.8	6739833.429	12251503.41	6724329.631	5113760.325	5323945.916	7502300.53	12304014.25	13491081.59	12754152.09
Com_2562_neg	Gluconic acid	--	C6 H12 O7	196.05808	1.324	195.05083	21361482.89	10582032.52	13285087.93	10788281.71	21954279.68	25595404.09	7068065.384	12395406.57	8904325.651	22478677.8	24359846.83	6392067.416	15838501.23	18794091.9	12037995.46
Com_2656_neg	δ-Gluconic acid δ-lactone	--	C6 H10 O6	178.04754	1.43	177.04037	9644655.52	14293897.26	20019822.19	11108952.43	20657805.69	14195889.87	8914302.582	14879108.01	11756361.48	12463280.83	26061515.72	13311543.52	13071203.35	14887927.49	12621032.37
Com_2660_neg	2'-Deoxyinosine	--	C10 H12 N4 O4	252.08555	3.913	251.07834	4100636.176	6660014.197	3397581.629	6897565.247	28077090.24	36091792.03	765760.7488	5903352.196	4009877.696	2898887.166	812215.0296	7068062.265	7296510.262	7042058.836	6369800.814
Com_3283_neg	N-Acetyl-DL-glutamic acid	--	C7 H11 N O5	189.06356	1.452	188.05629	25248793.29	13640893.1	12195991.9	12465481.71	23975415	14858049.4	3990020.272	5469759.755	4706522.004	5962784.203	3810995.686	8225981.051	11565148.96	12900257.13	11940820.26
Com_3470_neg	Uric acid	Urate; Uric acid	C5 H4 N4 O3	168.02809	1.388	167.02071	12481573.71	15155552.56	22836958.26	12897144.11	12391089.92	14579919.06	223431.7398	1747334.927	714698.3933	4830672.676	275397.8976	602050.3547	3959376.733	6742491.222	7377214.436
Com_3619_neg	Threonine	--	C4 H9 N O3	119.05802	1.308	118.05067	237770.7345	263502.1504	1153453.859	1198631.102	1114194.784	1010808.291	808436.5526	10336179.45	4128851.198	14154620.75	1218233.605	5794263.68	3675405.1	3773109.397	3343155.439
Com_3870_neg	Glycerophospho-N-palmitoyl ethanolamine	--	C21 H44 N O7 P	453.28526	9.433	452.27805	9128801.331	23082305.34	15595314.26	5911059.019	19144643.02	6719129.624	5090780.66	1461694.243	1002048.03	1065935.742	732147.6779	1524217.881	7759820.104	7179494.699	6948004.03
Com_4098_neg	(2R)-2,3-Dihydroxypropanoic acid	--	C3 H6 O4	106.02652	1.474	105.01926	12260279.96	6751965.778	8714867.897	7528287.048	14688689.38	13634901.11	9770526.752	12182564.4	11584402.33	8474611.787	14525839.04	7617399.96	8619887.329	10726947.05	8891565.374
Com_4156_neg	Xylitol	Xylitol	C5 H12 O5	152.06824	1.337	151.06097	634709.2209	636003.0688	515669.2747	399880.4079	655506.7159	592676.337	4160915.218	7898238.643	11351217.21	6869422.896	14163376.29	8280827.854	525542.2341	680714.3227	682720.0856
Com_4293_neg	N-Acetylaspartic acid	--	C6 H9 N O5	175.04792	1.388	174.04045	8867569.682	7130875.35	7292012.781	9223981.986	17137388.56	9571943.151	4031386.481	4595172.988	5148753.618	9577857.964	5322471.214	4801993.277	6133290.363	7971924.129	7546963.529
Com_4326_neg	N-Acetylvaline	--	C7 H13 N O3	159.08934	4.981	158.08195	11007011.23	6667403.37	10362619.38	14465522.66	15261049.17	16512509.06	5278220.082	5135176.343	5451268.739	6454460.246	3018577.691	5336954.989	7395619.123	8322513.601	8756346.046
Com_4412_neg	trans-Cinnamic acid	trans-Cinnamate; trans-Cinnamic acid; (E)-Cinnamate; (2E)-3-	C9 H8 O2	148.05216	5.54	147.04474	16260469.78	17293370.72	15455844.75	13032437.29	12520505.08	12744571.06	9658969.108	6130653.731	8302066.876	7070366.518	6612937.518	5817559.96	9878474.444	10340882.36	13913277.5
Com_4648_neg	D-(-)-Fructose	--	C6 H12 O6	180.06325	1.448	179.05598	7785008.921	4637556.979	9330202.959	6156830.783	9298041.922	8356935.672	3764382.867	6469573.187	5621245.131	9588410.314	10297549.1	4097465.872	6600200.099	7663730.051	6305914.821
Com_4811_neg	Guanine	Guanine; 2-Amino-6-hydroxypurine	C5 H5 N5 O	151.04921	1.399	150.04185	1859398.205	1163609.682	2418776.476	2669767.88	2040171.377	2113361.627	2214808.784	11464957.73	3686325.184	1102088.238	1653076.009	7272085.058	2797994.635	3199006.779	3326904.444
Com_5107_neg	Rutin	Rutin; 3-[[6-O-(6-Deoxy-alpha-L-mannopyranosyl)-beta-D-glucopyranosyl]oxy]-2-(3,4-dihydroxyphenyl)-5,7-dihydroxy-4H-1-benzopyran-4-one; Quercetin 3-rutinoside; Rutoside;	C27 H30 O16	610.15278	5.594	609.14551	1063935.211	919828.4974	876520.9235	1008733.87	1075877.952	1133280.362	3618476.206	10441778.58	1762859.79	378433.5586	1940155.214	1853646.716	839695.9676	740736.7593	697430.5871
Com_5409_neg	L-Phenylalanine	L-Phenylalanine; (S)-alpha-Amino-beta-phenylpropionic acid	C9 H11 N O2	165.07869	5.123	164.07144	11763610.86	10351438.7	10889638.38	7691853.084	10340426.99	10637770.18	4589987.044	4728654.279	4484752.908	5180978.502	6486575.327	5213296.621	8124843.118	8180578.658	5816496.062
Com_7134_neg	L-(-)-Arabitol	--	C5 H12 O5	152.0682	1.454	151.06093	598152.7587	494774.6368	565052.3904	598056.6697	1611763.743	910571.7239	1228083.713	2550733.995	3029503.177	1931735.497	5678180.724	2080206.699	1047219.184	926059.9106	884253.1498
Com_7726_neg	4-Pyridoxic acid	4-Pyridoxate; 4-Pyridoxic	C8 H9 N O4	183.05296	2.228	182.04568	897375.4337	1111184.455	1236961.766	831315.2673	1249640.044	701349.9354	1452712.285	3178140.572	2659406.07	4051642.74	2735984.287	2203340.595	1747803.942	1234747.64	1324074.725
Com_9910_neg	L-Cysteic acid	L-Cysteate; L-Cysteic acid; 3-Sulfoalanine; 2-Amino-3-sulfopropionic acid	C3 H7 N O5 S	169.00421	1.564	167.99693	249251.367	503542.59	607133.2499	570183.249	853068.6385	682980.0704	1537526.48	2441536.406	1409137.792	1079358.001	894754.7635	3088867.827	998634.2509	927869.2698	743907.4589
Com_10015_neg	Xanthosine	Xanthosine	C10 H12 N4 O6	284.07529	4.738	283.0679	3419719.387	1636256.786	1849866.183	1413749.177	3687946.837	3246271.907	1064829.379	2476794.636	734134.3532	1380829.676	1130372.606	1364683.28	1614193.347	1899705.951	1542001.307
Com_11962_neg	2,4-Dihydroxybenzoic acid	--	C7 H6 O4	154.02651	0.584	153.01932	2181281.195	855699.1443	1452684.763	800120.4656	883021.2612	972584.285	1336498.347	854107.2464	926422.4034	1479164.619	1020588.113	446413.1456	1176717.475	1331844.892	1366038.024
Com_469_neg	N-Acetylneuraminic acid	N-Acetylneuraminate; N-Acetylneuraminic acid; 5-Acetamido-3,5-dideoxy-D-glycero-D-galacto-2-nonulosonic acid; Neu5Ac	C11 H19 N O9	309.10567	1.542	308.09824	52440577.32	62304461.4	135193343	60799165.45	118452409.2	178863016.1	11153918.76	73539679.44	24286764.27	154097122.4	94835159.28	33016641.82	87077032.45	105242490	98157709.57
Com_573_neg	Guanosine	Guanosine	C10 H13 N5 O5	283.09132	3.284	282.08408	63027701.65	73749868.99	66949964.56	51759510.9	194326938.6	135635145.8	4758861.495	70551909.27	36823451.74	4877658.031	7371180.197	52590731.11	49028833.37	52012325.77	48817694.51
Com_626_neg	4-Hydroxybenzaldehyde	4-Hydroxybenzaldehyde; p-Hydroxybenzaldehyde	C7 H6 O2	122.03666	5.57	121.02953	101062855.8	103394711.5	94693911.43	102510161.9	99269468.32	103653766	118071494	87090048.84	123091436.6	103721817.2	138309646.8	93300128.67	92836259.62	110922116.7	84438206.81
Com_1240_neg	Kynurenic acid	4-Hydroxy-2-quinolinecarboxylic acid; Kynurenic acid; Kynurenate	C10 H7 N O3	189.04236	5.828	188.03508	85088655.43	80176667.47	53272918.52	59336171.29	79305828.51	76915027.31	55795239.65	4669970.057	10132441.4	5466581.134	24181684.5	7048231.766	40271022.62	40209166.62	41817406.93
Com_537_neg	Thymidine	Thymidine; Deoxythymidine	C10 H14 N2 O5	242.09003	4.881	241.08287	38463903.13	66426002.8	68348231.93	67995954.88	81457883.75	228051425.6	7904951.191	24347609.6	77184783.74	133034871.7	6621790.12	82199934.31	73648322.62	64144986.03	56825511.77
Com_1455_neg	D-Raffinose	--	C18 H32 O16	504.16882	1.375	503.16129	16973561.72	9933194.025	12995917.03	8645411.829	18484153.04	18698352.18	23116852.91	56313075.75	38653372.68	13723070.72	48974222.68	28298673.25	19257705.24	24103446.42	20639875.45
Com_3256_neg	Xanthurenic acid	Xanthurenic acid; Xanthurenate	C10 H7 N O4	205.03721	5.257	204.02985	8632252.231	11318261.36	9140031.315	8368804.414	7958777.923	7401304.317	23549016.05	5512485.614	10752396.7	4991434.313	17393648.77	6753398.933	7879637.696	8925077.909	9131785.372
Com_6151_neg	Glycyl-L-leucine	Glycyl-leucine; Glycyl-L-leucine	C8 H16 N2 O3	188.11582	1.361	187.10846	6059512.021	2675763.587	5712399.707	5703261.177	3927232.721	10088520.21	2347897.686	7640846.342	3354109.465	1887227.109	2735305.518	4734345.93	3472858.544	4362991.178	4578260.032
Com_340_pos	Nicotinic acid	Nicotinate; Nicotinic acid; Niacin; 3-Pyridinecarboxylic acid	C6 H5 N O2	123.03212	1.756	124.03946	420991598.8	201081039.3	307360622.6	316080660.9	359242489.5	365936665.9	121313461	162671442.3	209630870.9	356941875.7	136148036.1	192245886.5	211297689.4	216900621.2	206845527.9
Com_441_pos	Pipecolic acid	L-Pipecolate; Pipecolinic acid; Pipecolic acid; 2-Piperidinecarboxylic acid; (S)-Piperidine-2-carboxylic	C6 H11 N O2	129.07892	1.354	130.08614	18596249.19	19311298.93	36059433.1	23050157.56	41946957.12	38957291.19	35514911.96	23443507.04	51103038.25	34316985.71	288034777.4	30698567.43	53983648.42	41596403.33	43674856.99
Com_760_pos	Choline	Choline; Bilineurine	C5 H13 N O	103.09992	1.165	104.1072	97647637	63649246	65209427.81	36125721.27	179191781	109006009.9	96075988.45	126654358.8	173444175.3	137740058.7	169580546.2	138072521.1	132560168.2	108687238.7	104418450.8
Com_984_pos	Cytosine	Cytosine	C4 H5 N3 O	111.04342	1.349	112.05066	9324334.185	14708485.32	42014587.16	28904412.05	49970982.51	53473174.43	13381030.41	61506925.09	109862440.2	29078625.22	17522482.4	132285594.5	34699586.45	32215040.79	29502231.9
Com_1230_pos	2'-Deoxyadenosine	Deoxyadenosine; 2'-Deoxyadenosine	C10 H13 N5 O3	251.10159	2.758	252.10899	154491321.8	78379886.14	93595465.75	58495118.65	23243994.05	30400614.13	1291843.376	12426749.84	7360265.472	497946.228	562421.4382	20149002.08	20912395.52	25361073.96	18723230.3
Com_1407_pos	cis-Resveratrol	--	C14 H12 O3	228.07857	5.629	229.08582	31319262.93	62856435.19	42780329.74	28234676	34367782.49	47494821.85	87785261.31	55839007.73	67831311.4	73443443.11	16692877.03	41959592.45	29232090.36	25468777.14	24071044.87
Com_1724_pos	Thymine	Thymine; 5-Methyluracil	C5 H6 N2 O2	126.04303	4.818	127.05032	26124596.58	39810214.06	48001199.55	32479677.1	51193026.94	121890597.8	5148621.054	13947344.98	48940104.59	49221886.35	4752607.353	51492073.31	36183381.12	36695868.45	34487194.77
Com_2541_pos	4-Methoxybenzaldehyde	--	C8 H8 O2	136.05233	5.705	137.05962	53556267.46	53351744.19	42592616.22	57436036.32	47935948.57	44320870.67	30355829.7	27445498.53	36874357.75	32616303.64	27409086.29	34184809.91	40611289.73	35565387.07	32234379.83
Com_3273_pos	Urocanic acid	Urocanate; Urocanic acid	C6 H6 N2 O2	138.04279	1.356	139.05008	26848787.99	27253123.22	23742409.33	26937992.6	48177694	34651461.01	6083967.452	15919058.91	17676807.3	31560374.08	6875505.914	10412985.49	23657975.73	21885886.79	20218167.37
Com_3383_pos	2-Amino-1,3-octadecanediol	--	C18 H39 N O2	301.29768	6.696	302.30487	2521048.246	1418682.043	1323136.794	1235545.933	2886425.743	1604337.279	13423448.61	26235795.92	4956751.359	4990363.547	8258552.586	15235740.93	5961748.533	5457111.106	5780053.916
Com_3656_pos	DL-Homoserine	--	C4 H9 N O3	119.05837	1.26	120.06565	5504996.061	6656914.248	11560506.1	16694033.48	18303598.89	15144905.2	7055273.038	13543565.01	8453311.369	27459581.42	2567754.672	11123761.39	14541039.9	11832196.83	11375861.73
Com_3834_pos	D-(+)-Proline	--	C5 H9 N O2	115.06351	4.948	116.07079	10215197.17	9909492.392	7648823.047	10581399.96	13022704.29	11300060.44	4029397.123	4514190.264	5730822.486	25478033.97	3284971.963	4490329.609	9847638.185	7345178.315	7070803.065
Com_3836_pos	Progesterone	Progesterone; 4-Pregnene-3,20-dione	C21 H30 O2	314.22412	7.491	315.23129	22228963.16	13216211.41	17609586.9	33991981.92	19826824.12	16110057.03	12038636.69	8011140.778	9500103.739	17556440.97	17871409.33	7984440.591	15106651.36	14451919.76	18151943.8
Com_4042_pos	Trifolin	Kaempferol-3-O-galactoside; Kaempferol 3-O-beta-D-galactoside; Trifolin	C21 H20 O11	448.09979	5.694	449.10703	2864033.686	4623729.195	4095617.682	6775767.839	3940136.497	3580033.602	9889655.281	18619034.7	26633172.28	9338663.897	11323553.37	23582663.84	8880289.509	8602507.068	8040659.314
Com_4492_pos	Kaempferol	Kaempferol; 3,4',5,7-Tetrahydroxyflavone; 3,5,7-Trihydroxy-2-(4-hydroxyphenyl)-4H-1-benzopyran-4-one; 5,7,4'-Trihydroxyflavonol; C.I. 75640; Indigo yellow; Kaempherol; Kempferol; Nimbecetin; Pelargidenolon; Populnetin; Rhamnolutein; Rhamnolutin; Robigenin; Swartziol; Trifolitin	C15 H10 O6	286.04744	5.905	287.05472	1055370.202	1277404.599	836803.6037	731806.9977	1069418.932	1251865.945	9643384.496	17821817.2	19335724.82	3622060.507	9888210.751	20772472.59	4990157.644	4315600.886	4532655.922
Com_5363_pos	(R)-Equol	--	C15 H14 O3	242.09414	6.224	243.10155	19272264.99	18976047.51	16876997.35	17189082.64	12121889.99	15962892.97	3562206.111	3658507.791	7392969.433	8410484.871	16384564.47	4398514.03	9495009.964	9155486.702	8645874.381
Com_5483_pos	N6-Me-Adenosine	--	C11 H15 N5 O4	281.11242	4.783	282.11957	19028820.18	16662544.81	22561410.67	20525544.26	16457063.21	14469253.13	3942011.145	5933367.425	8284324.111	5660588.887	2015377.297	5131225.546	11221297.59	9725922.782	11332278.25
Com_6374_pos	Riboflavin	Riboflavin; Lactoflavin; 7,8-Dimethyl-10-ribitylisoalloxazine; Vitamin	C17 H20 N4 O6	376.13822	5.357	377.14554	4192821.602	8307176.294	7908697.326	9141666.516	8631103.687	3592204.007	2871574.657	5332449.419	9684833.08	8005128.792	12426091.3	4927037.332	6574756.63	7017060.217	6628544.173
Com_8033_pos	Thiamine	Thiamine; Thiamin; Vitamin B1; Aneurin; Antiberiberi factor	C12 H16 N4 O S	264.10406	1.172	265.11136	2138447.219	3020703.517	1616594.625	4253928.167	2905581.86	2734535.648	1124903.947	497900.018	4953816.428	8103895.426	195737.8802	857254.9595	3196846.279	2699110.137	1915296.941
Com_8238_pos	N,N-Bis(2-hydroxyethyl)formamide	--	C5 H11 N O3	133.07375	1.278	134.08101	2219876.388	2724209.967	4722928.591	4627361.525	4838768.366	3202522.757	2398204.686	6728232.755	2775998.105	6878927.863	2921616.455	7859026.497	4131304.314	4672673.565	3913077.584
Com_259_pos	Adenosine	Adenosine	C10 H13 N5 O4	267.09643	2.552	268.1037	733161253.5	399210756.5	696093597.9	172286946.1	204037508	251674948.1	19506975.48	54119750.71	22838107.82	3487480.486	6575718.471	46990178.88	108048288.5	101693697.8	101135037.9
Com_360_pos	Hypoxanthine	Hypoxanthine; Purine-6-ol	C5 H4 N4 O	136.03845	3.193	137.04573	93001364.49	157819993.1	96982400.23	54158656.78	544888420.9	568069865.6	29210592.77	78096498.99	111585847.9	17766436.9	38423904.03	78325881.14	127266911.3	117121262.8	113884259.5
Com_465_pos	Eicosapentaenoic acid	(5Z,8Z,11Z,14Z,17Z)-Icosapentaenoic acid; (5Z,8Z,11Z,14Z,17Z)-Eicosapentaenoic acid; Eicosapentaenoic acid; 5,8,11,14,17-Icosapentaenoic acid; (5Z,8Z,11Z,14Z,17Z)-Eicosapentaenoate	C20 H30 O2	302.22419	6.671	303.23148	330513782.1	333471657	401822003.7	339886103.2	284656968.6	339027088.2	23879280.72	14887166.09	21106567.56	39577172.59	21088453.51	18423151.24	157172650.1	156604776.8	160808041.3
Com_602_pos	L-Tryptophan	L-Tryptophan; Tryptophan; (S)-alpha-Amino-beta-(3-indolyl)-propionic acid	C11 H12 N2 O2	204.09	5.068	205.09726	36126649.37	26868132.49	34643311.85	46421643.19	45812608.4	42888465.58	11121298.07	57374693.58	48844733.05	208348447.7	27651274.61	41079614.17	50221190.68	45295997.34	45755291.33
Com_1163_pos	Pantothenic acid	Pantothenate; Pantothenic acid; (R)-Pantothenate	C9 H17 N O5	219.11075	5.02	220.11784	81555078.71	79769672.95	61374413.66	53124552.07	84047972.66	77262102.49	26396860.35	58999725.84	80801741.77	103294620.5	48664481.47	66225148.14	60210697.77	61052544.62	56622421.59
Com_3786_pos	Dehydrocholic acid	Dehydrocholic acid; 3,7,12-Triketo-5beta-cholanoic acid	C24 H34 O5	402.23997	6.272	403.24704	38861331.05	39649069.43	33976331.88	34727682.68	29914026.62	32693719.42	17744620.21	17430256.39	12266874.44	14774854.78	23939958.47	14175582.46	21605806.23	22217360.77	18856933.05
Com_4064_pos	Phenylacetylglycine	Phenylacetylglycine	C10 H11 N O3	193.07381	1.911	194.08102	1510096.081	1838684.001	1374149.118	1345329.659	1589783.008	1650106.456	6083967.541	20392446.93	9351689.694	13433853.07	8373726.092	23161583.35	4600930.596	4376389.245	4010469.639
Com_7590_pos	L-Saccharopine	N6-(L-1,3-Dicarboxypropyl)-L-lysine; Saccharopine; L-Saccharopine; N-[(S)-5-Amino-5-carboxypentyl]-L-glutamic acid	C11 H20 N2 O6	276.13156	1.253	277.1387	3441112.001	4700439.192	6425706.053	7505584.233	14454947.36	9323037.441	1091537.975	1166302.216	795894.4802	555436.4704	669822.8091	1356001.849	6118520.055	6158341.159	5728828.537

Com_8_neg	Phenylpyruvic acid	Phenylpyruvate; Phenylpyruvic acid; alpha-Ketohydrocinnamic acid; keto-Phenylpyruvate; 3-Phenyl-2-oxopropanoate; 2-	C9 H8 O3	164.04714	5.657	163.0401	6795970203	6468418822	6342998441	6874292960	7030067673	7329959950	2729911776	2791219653	2698263448	2563535589	2216150065	3220975334	5029348339	5129112524	4783112801
Com_14_neg	Glycodeoxycholic acid	Glycodeoxycholate; Glycodeoxycholic acid; 3alpha,12alpha-Dihydroxy-5beta-cholan-24-oylglycine	C26 H43 N O5	449.3136	7.915	448.30637	113777871.7	94898007.85	284378415.8	224571516.1	96713681.16	122343555.5	2877109611	2718484246	607871183	228766714.6	4893011013	2687367197	1360200024	1360404110	1262716122
Com_17_neg	Deoxycholic acid	Deoxycholic acid; Deoxycholate; 3alpha,12alpha-Dihydroxy-5beta-cholanate; 3alpha,12alpha-Dihydroxy-	C24 H40 O4	784.58517	8.353	783.578	562287957.7	998239485.7	2374264550	3437310675	378126632.6	533967826.2	576022002.1	344561853.6	413318795.7	3349097260	503656998.3	835947154.9	1593206379	1449744015	1390720565
Com_20_neg	6-(4-methoxyphenyl)pyrimidine-	--	C11 H12 N4 O	216.09945	5.527	215.09216	68304287.74	66974962.37	56564496.5	52686091.28	44903983.69	44845813.07	1552383117	2710504640	3006123061	2974271747	1001218197	2938859135	1132323399	1117091050	1110118920
Com_38_neg	Prostaglandin H2	Prostaglandin H2; (5Z,13E)-(15S)-9alpha,11alpha-Epidioxy-15-hydroxyprosta-5,13-dienoate; (5Z,13E,15S)-9alpha,11alpha-Epidioxy-15-hydroxyprosta-5,13-dienoate; PGH2; (5Z,9alpha,11alpha,13E,15S)-9,11-Epidioxy-15-hydroxy-prosta-5,13-dienoate	C20 H32 O5	334.21403	7.716	333.20667	2291031202	2365813438	2703674357	2150915992	1804741100	1977751687	90439446.47	108111113.1	140505231.5	146379332.8	47375237.32	109478344.9	992196820	1100560816	1084903927
Com_48_neg	Elaidic acid	(9E)-Octadecenoic acid; 9-Octadecenoic acid, (E)-; (E)-Oleic acid; D9-trans-Octadecenoic acid; 9-trans-Octadecenoic acid; trans-D9-Octadecenoic acid; trans-9-Octadecenoic acid; trans-Elaidic acid; trans-Oleic acid; Elaidic acid; Acide elaidique; Elaidinsaure	C18 H34 O2	282.25544	10.578	281.24814	1236657449	1512994802	1527690484	843758772.6	1385835026	1276404705	1431578624	1850219054	1054585257	630479425.1	927227893.5	1419855677	1199177953	1277930063	1161451169
Com_76_neg	Glycochenodeoxycholic acid	Glycochenodeoxycholate; Glycochenodeoxycholic acid;	C26 H43 N O5	449.31351	7.745	448.30615	41580149.31	37836552.88	110678906.9	84940354.91	62940593.36	70582879.44	1523521007	907008538.4	873079366.7	288614726	915265880.6	908110531.5	510428113	499901082.3	493046442
Com_117_neg	gamma-Nonanolactone	--	C9 H16 O2	156.11475	6.216	155.10768	1067612604	989605712.1	507465631.9	504170372	797742580.5	713332507.6	545589630.1	367243966	501372986.6	475276806.3	513698701.3	326373431.2	593321625	577161524.7	604384626
Com_137_neg	2-Ketohexanoic acid	--	C6 H10 O3	130.0628	6.381	129.05556	76859583.35	49900980	54441355.57	112426609.9	95195389.94	69937299.54	13758698.33	188560908.9	136354438.7	527139717.1	237342594.7	77987148.9	118122078.9	116691063.1	120707035.8
Com_159_neg	β-Muricholic acid	beta-Muricholate; beta-Muricholic acid; 3alpha,6beta,7beta-Trihydroxy-5beta-cholan-24-oic acid; 5beta-Cholanic acid-3alpha,6beta,7beta-triol	C24 H40 O5	816.57505	7.586	797.55725	5567093.727	5149430.814	20788410.54	38187024.59	8426103.248	12465597.1	71646537.3	151065896.2	70616946.79	476310079.3	160386803.1	126858731	80986432.33	64446629.72	69732864.92
Com_160_neg	1-acetyl-3-(2-furylmethylene)tetrahydropyrazine-2,5-dione	--	C11 H10 N2 O4	234.06766	7.856	233.06026	6835989.185	6558128.162	6940872.489	5768413.566	6820620.937	6874248.535	239073902	420947440	557237714.4	472185277.2	115446398.8	400642785.9	211027740.7	220100593.9	184520605.5
Com_169_neg	Ethyl chrysanthemumate	--	C12 H20 O2	196.14614	5.823	241.14435	140976954.1	209890456.4	110542181.1	47322457.37	41181799.65	127791457.8	329845385.8	480080538.7	261794560.2	449372899.9	325706948.4	448445898.1	97702708.59	182867257.7	184578556.6
Com_182_neg	15(R),19(R)-Hydroxy prostaglandin F1α	--	C20 H36 O6	354.24035	7.414	353.23309	8550108.675	7652818.172	7986856.305	8689373.455	8382052.036	7418700.914	110145938.4	96485965.43	25553412.13	25150883.32	483098700.1	66075132.42	95160253.11	82831268.55	88835334.29
Com_291_neg	cis-5,8,11,14,17-Eicosapentaenoic acid	--	C20 H30 O2	302.22415	10.021	301.21686	33207741.45	33263707.86	60749704.53	63677707.1	26353565.24	33120204.08	29535034.13	153664323.5	218157558	264556973	37431090.04	145649228.5	106695263.7	103144449.2	108843623.1
Com_297_neg	11(Z),14(Z)-Eicosadienoic	--	C20 H36 O2	308.27114	10.769	307.26367	65891722.11	90419631.88	62047320.52	26782817.13	48798733.15	34169331.2	122688364.5	141972587.5	170434425.6	256127088.3	163530077.2	133427219.2	124765697.7	129474860.8	127775963.7
Com_311_neg	trans-2-Methyl-2-pentenoic	--	C6 H10 O2	114.06777	5.524	113.06062	242929750.4	224259452.7	189923330.8	204118699.3	238623515.4	233091363	187183905.9	129760805.8	207442877.7	182790721.7	295955163.3	142262973.6	178670204.1	197929433.1	184293784.9
Com_320_neg	11-dehydro Thromboxane B2	--	C20 H32 O6	350.20908	6.964	349.20169	361442864.8	352243856.5	353718756.2	309556738.9	260730360.3	258788775.4	115240038.1	19907514.17	20028842.35	21002055.39	21832470.36	19237744	171627483.6	177546245	148255108.7
Com_322_neg	(+/-)5-iPF2α-VI	--	C20 H34 O5	354.24024	7.057	353.23297	115697225.3	95284430.32	57671575.63	111656086.1	82468464.21	117741856.8	296746340.4	97543561.18	226643712.6	231389143.8	193874972	77427734.56	129945167.1	91523975.91	124263748.4
Com_330_neg	Cyclohexaneacetic acid	--	C8 H14 O2	142.09912	5.308	187.09734	19298101.02	15603950.95	13017930.53	12000041.48	15147666.06	13216737.93	41880328.81	46466350.57	51655255.05	52606862.46	277384438.5	79509690.32	35190735.2	37395692.81	34561346.37
Com_336_neg	(+/-)-CP 47,497-C7-Hydroxy metabolite	--	C21 H34 O3	334.25043	9.159	333.24301	349096167.5	313180607.6	73821832.97	66707896.4	83123884.24	72295915.46	483873.0817	518112.0754	969417.15	712217.8148	786738.6098	675825.5214	65032239.02	69343641.32	58694590.46
Com_338_neg	Azelaic acid	Azelaic acid; Nonanedioic acid; 1,7-Heptanedicarboxylic acid	C9 H16 O4	188.10452	5.291	187.0972	16704120.82	10125785.82	8012778.162	7419030.953	9832188.7	8989258.559	36410605.88	37886244.1	42349944.56	50254590.82	272020168.8	35012068.82	27884193.17	30654780.45	29968855.71
Com_341_neg	L-Tyrosinemethylester	L-Tyrosine methyl ester	C10 H13 N O3	195.08923	5.598	194.08191	309430217.3	345967318.9	331292192.4	287375080.7	336575777.3	332444757.5	4865324.66	2721784.538	1363858.026	2725422	2983414.752	4557936.229	181825460.4	179610936.1	149053102.1
Com_343_neg	Hexadecanedioic acid	--	C16 H30 O4	286.21405	7.857	285.20682	7307117.779	7785118.739	4713914.86	4916697.435	4666447.082	5013130.108	43102019.21	5139554.155	7080664.138	9001308.616	267127872.2	5985471.378	40761196.58	36332333.37	42752014.03
Com_345_neg	(±)9(10)-DiHOME	--	C18 H34 O4	314.24525	7.271	313.23795	9316406.047	8992702.989	9376271.015	13219483.23	9841517.991	8880578.959	141634177.7	167899013	248296751.2	217702562.1	129910361.3	141809918.9	95581280.88	90714148.15	89996919.37
Com_361_neg	4-Methoxycinnamic Acid	--	C10 H10 O3	178.06273	6.159	177.05537	326813465.1	16380303.98	42674604.59	23354938.14	13050400.04	112886255.3	2366312.943	1492251.206	1446601.953	926090.0273	1347426.719	1782388.114	50339042.45	49941475.37	43779110.89
Com_378_neg	MGMG (18:2)	--	C27 H48 O9	516.32881	7.114	515.32202	5897131.134	4370102.235	2825436.065	3526431.197	5090370.792	3797110.119	135030847.3	139498883.4	164912978.5	194572306.6	150119898	105710143.2	61189334.15	46810844.61	59706498.05
Com_424_neg	L-Glutamic acid monosodium salt	--	C5 H8 N Na O4	169.03463	1.341	214.03276	24987097.47	42726464.34	76856691.12	92683185.62	151239461.7	67255275.36	14252045.96	5402211.466	56195059.41	172172096.5	1274994.421	27658022.32	61424820.16	99414811.06	73637005.17
Com_452_neg	10-Hydroxydecanoic acid	10-Hydroxydecanoic acid; 10-Hydroxydecanoate	C10 H20 O3	188.14105	6.294	187.13368	149859689.4	114631363.7	96550352.51	151259334	188976099.4	124345746	229140676.2	158910475.9	179839441.4	46040072.61	129976798.3	136266039.9	91186133.93	188484548.9	151855997.9
Com_476_neg	1-(2,4-dihydroxyphenyl)-2-(3,5-dimethoxyphenyl)propan-	--	C17 H18 O5	302.11498	5.673	301.10773	217479658.1	207121750.7	231007611.6	198163820	226545735.6	223018294.9	132239917.1	120307292.8	102945631.6	99451606.36	78537531.7	82703672.67	159584582.4	178179170.4	194493327
Com_491_neg	16-Hydroxyhexadecanoic acid	--	C16 H32 O3	254.22426	9.825	253.21703	180976123	150334620.9	222214067.3	126265039.7	171590828.4	169369064.6	18288402.37	40590335.93	40732927.92	33457623.98	47320557.99	26078611.36	63091579.01	59459314.56	60689532.93
Com_498_neg	Valproic acid	Valproic acid	C8 H16 O2	144.11484	5.999	143.10738	226518618.8	204477519.5	89461060.29	99208087.57	167476298.7	141465727.1	28494557.51	30870535.49	36172640.84	36288677.3	35491520.64	30394458.21	94130295.24	94512145.74	85385468.98
Com_510_neg	(±)9-HpODE	--	C18 H32 O4	312.22963	7.531	311.22232	31912194.54	26537299.94	28279470.26	25221419.03	41791078.25	34895948.24	115048302.7	134844670.9	146374545.1	142286820.7	151983537.3	146494522.9	95022191.86	93860659.18	106968537.6
Com_517_neg	Corchorifatty acid F	--	C18 H32 O5	328.22466	6.572	327.21735	82579396.08	70800177.19	62171805.22	73070275.74	77070308.07	69906273.15	107966262.4	81941494.89	105869260.8	140524117.9	91266707.59	76714929.78	76944211.98	78870100.47	75960066.59
Com_522_neg	Butylparaben	--	C11 H14 O3	194.09398	6.096	193.08659	35807163.3	35852435.53	14501248.92	12288721.73	26718039.96	24855948.81	56022005.84	60244884.49	134719361.2	138185982.4	142688109.7	60802506.64	51667099.39	51912883.98	48895985.68
Com_567_neg	Chenodeoxycholic Acid	Chenodeoxycholate; Chenodeoxycholic acid; 3alpha,7alpha-Dihydroxy-5beta-cholanic acid;	C24 H40 O4	438.29748	7.517	437.29007	50530425.84	65305194.34	123364853.7	165832519	48563467.8	64433030.16	62901008.41	37433667.19	162613851.9	125927790.6	49596891.46	58269783.71	98501571.94	85491705.68	79673193.95
Com_601_neg	Benzyl cinnamate	--	C16 H14 O2	238.09908	5.64	237.09174	176307149.4	189516217.5	178823851.6	143192050.7	178245139.5	185163151.6	60037840.25	54228640.61	46752927.57	55758981.78	33478978.51	49647633.84	118610973.8	124690466.3	153283637
Com_615_neg	Phenylglyoxylic acid	alpha-Oxo-benzeneacetic acid; Benzoylformate; Benzoylformic acid; Phenylglyoxylic acid; Phenylglyoxylate; 2-Oxo-2-phenylacetate	C8 H6 O3	150.03127	5.378	149.02405	5443503.153	20679912.54	19888811.69	17123476.05	15781420.75	13945597.65	22320901.96	46902159.38	26611467.95	28825154.27	140491128.6	43612595.23	38677686.67	29180844.33	30773860.3
Com_675_neg	Prostaglandin F2α	Prostaglandin F2alpha; (5Z,13E)-(15S)-9alpha,11alpha,15-Trihydroxyprosta-5,13-dienoate; (+)-Prostaglandin F2a; 7-[3,5-Dihydroxy-2-(3-hydroxy-1-octenyl)cyclopentyl]-5-heptenoic acid; 9a,11a-PGF2; Amoglandin; Cyclosin; Dinoprost; Enzaprost; Enzaprost F; Panacelan; PGF2a; 9,11,15-Trihydroxy-(5Z,9a,11a,13E,15S)-prosta-5,13-dien-1-oic acid; Prostaglandin F2a; Prostin F 2 alpha; Protamodin; U 14583; (5Z,9alpha,11alpha,13E,15S)-	C20 H34 O5	354.24027	6.535	353.23282	64714328.88	61323942.58	44897827.22	40759409.4	55424124.46	58815492.93	151284301.1	15281872.9	83237051.22	105155600.9	24197318.82	11765573.89	43018624.47	46793265.53	43180834.21
Com_714_neg	1-(1,8-dihydroxy-3,6-dimethyl-2-naphthyl)ethan-1-one	--	C14 H14 O3	230.09395	5.572	229.08662	115261409	150612812.1	145349999.2	101459406.6	131364588.9	151471531.8	41014860.97	28785253.47	41238092.24	9813469.454	10556095.21	30496443.63	101411506.6	108163115	103121796.7
Com_738_neg	FAHFA (2:0/17:0)	--	C19 H36 O4	328.26093	10.172	327.25366	145503658	164556776.7	121046188.7	90683901.29	26190350.34	99795144.49	55564717.74	39681573.95	70158683.87	71234060.09	50776393.86	37994714.73	77193976.15	70043906.19	70048369.99
Com_760_neg	2-{2-oxo-2-[(2-oxo-3-azepanyl)amino]ethoxy}acetic acid	--	C10 H16 N2 O5	244.10955	10.233	243.10217	2416287.265	1647078.479	1872213.059	1873101.514	1117900.231	1187935.634	3354481.929	24906595.46	52365301.09	90618088.01	18703454.72	27652708.97	23691522.19	17075939.72	23700424.76
Com_768_neg	Ethylmalonic acid	--	C5 H8 O4	132.04223	3.993	131.03496	12089870.09	8182184.889	50323567.12	119552378.6	42929230.82	33935776.45	36333666.68	12145757.9	15366719.45	16580397.53	12792745.33	15167421.51	24776841.76	23916945.81	22227302.39
Com_788_neg	Perillic acid	Perillic acid	C10 H14 O2	166.09913	5.447	165.09196	137157128.1	130373735	108175055	104953887.8	98727035.51	93794868.26	8196135.266	7012654.768	5031841.954	6650062.204	10984807.38	12999134.25	68350246.17	63110963.28	58006223.49
Com_792_neg	Hydrocinnamic acid	--	C9 H10 O2	150.06788	6.299	299.12857	104790534	118152026.6	97454238.86	116545648	99391143.17	92774549.4	29566673.79	29688282.11	45848609.11	49609002.44	29636517.46	28695504.64	68611475.26	83251761.78	75092633.28
Com_798_neg	Spiculisporic Acid	--	C17 H28 O6	328.18844	6.132	327.18137	130312100.9	146688638.9	114601534.7	113068403.8	135631482.6	104268746.2	51956881.66	53465060.18	36911023.43	61587026.72	71610862.12	45109496.47	71592326.87	67743084.59	100271928.3
Com_821_neg	19(R)-Hydroxy-prostaglandin	--	C20 H32 O6	350.20918	6.667	349.2019	131439731.1	130361029.4	91351434.56	72517879.19	106216445.6	95881004.34	111831430.7	23724686.05	27146493.5	24945777.58	21035721	21674092.74	78356732.24	73414986.65	64176015.96
Com_850_neg	Epinephrine	--	C9 H13 N O3	183.08922	5.012	182.08191	66123261.92	91480477.98	90439960.4	76835564.11	108657420.2	83141118.6	79747597.53	3488293.403	3075182.862	3745316.695	3633811.885	3116766.773	41116145.03	70325556.51	43228276.69
Com_872_neg	trans-10-Heptadecenoic Acid	--	C17 H32 O2	268.2399	10.209	267.23251	94645267.49	105625392.6	120351911.6	69687819.61	106848056.2	93166561.96	19606761.84	30702782.35	22244609.12	19064897.6	50826723.27	21560900.1	52967390.13	49173846.68	54027047.16
Com_946_neg	19(R)-Hydroxy prostaglandin F1α	--	C20 H36 O6	372.25084	6.526	371.24356	12722566.5	11821223.54	9963477.983	9874442.066	11993656.05	11238061.35	24483921.62	14068636.43	87936212.65	11397763.06	20598675.09	18875685.45	19151481.09	[illegible]	[illegible]
Com_949_neg	1a,1b-Dihomo prostaglandin	--	C22 H38 O5	364.26101	8.025	363.25369	7756036.246	6949136.943	21848683.43	29174926.71	11697824.26	11469741.13	37869287.53	52481648.39	62760329.81	71693790.14	43561082.49	35286578.37	34431689.14	32425747.26	31636634.74
Com_979_neg	Cinnamyl alcohol	Cinnamyl alcohol; 3-Phenyl-2-propen-1-ol; Styrylcarbinol	C9 H10 O	134.07294	5.445	133.06583	43420096.37	43903228.04	43550461.77	47673776.35	42943659.95	42211115.98	48250439.06	77962772.07	64830058.99	60891680.58	58971839.47	88626861.84	51286121.12	51290543.06	49015925.32
Com_987_neg	FAHFA (24:4/6:0)	--	C30 H50 O4	474.37039	9.734	473.36301	3174110.87	2699196.058	2664742.763	2261109.352	2750933.907	2289118.064	17395877.65	22487124.69	49283087.65	69059936.39	9970891.808	24391682.21	17542827.65	16859353.05	18925256.89
Com_989_neg	L-(+)-Arabinose	--	C5 H10 O5	150.05265	1.387	149.04558	77274551.2	41749072.41	30009120.17	30864040.94	45445807.5	96431651.08	26350573.84	52744614.41	27985547.26	68924018.79	79900313.86	38034271.23	61996835.6	64890866.38	32112791.33
Com_996_neg	8,15-Dihete	--	C20 H32 O4	336.2299	7.788	335.22263	9862077.342	9387743.939	9763997.815	10041975.11	11294376.37	10702432.27	42323636.43	30538318.6	43138282.38	58053088.15	83500290.43	25555889.49	26086653.17	27048644.55	25190416.69
Com_1007_neg	Prostaglandin D1	Prostaglandin D1	C20 H34 O5	336.2299	6.692	335.22269	84121340.15	72233090.16	94449745.42	90061220.2	73294008.7	86040186.79	23436810.79	58179333.99	22504901.29	35263943.89	27997906.28	28748650.44	69287878.02	58533819.43	63818833.63
Com_1019_neg	2-(2-carboxy-2-methylpropyl)-4,6-dimethylbenzoic acid	--	C14 H18 O4	250.12023	5.662	249.11302	104138808	35189032.63	100626469.1	24803792.62	88715158.76	27989226.86	6923208.115	8104360.923	41353773.52	33147913.5	47021592.26	8036007.55	17884482.14	18007637.59	22325807.65
Com_1035_neg	N-Glycolylneuraminic acid	--	C11 H19 N O10	325.10055	1.52	324.09329	84779308.09	75761541.57	30883068.53	15273934.71	50315479.6	110546362.3	7986418.105	9397454.432	9220817.097	36026525.01	20795774.36	7686825.642	43578861.91	54253676.34	51580757.14
Com_1044_neg	Ethyl-β-D-glucuronide	--	C8 H14 O7	222.07441	1.407	267.07297	31094635.87	31822697.96	47821388.37	32283505.17	100815807.2	85298524.15	35093615.24	68984146.38	82816138.18	18096971.87	69001664.28	45848276.76	45201285.13	66451023.65	52016245.54
Com_1048_neg	Phloretin	Phloretin	C15 H14 O5	274.08404	5.572	273.07675	52962879.19	99208843.16	90439960.4	87587901.51	100434045.6	104338042.3	40859508	14027104.05	37069436.83	13316781.55	35391156.45	30324474.73	55838086.53	50878789.52	62865252.35
Com_1053_neg	Prostaglandin H1	--	C20 H34 O5	336.22988	6.908	335.22269	92305735.25	73349458.27	69846359.49	85371613.64	58721982.46	95758557.95	41219937	54812387.8	37586703.43	45514086.24	40932091.97	41966483.14	51083038.78	50090087.66	48030258.97
Com_1087_neg	Nonanoic acid	Nonanoic acid; Nonanoate; Pelargonic acid	C9 H18 O2	158.13049	6.231	157.12328	96271437.79	87457463.03	49402155.27	47503172.88	69264666.17	60750613.87	38355314.91	27579739.63	38371030.83	38461456.35	38436532.54	27108425.22	52181119.14	47978657.61	49985698.16
Com_1156_neg	o-Toluic Acid	o-Toluate; o-Methylbenzoate; o-Toluic Acid; 2-Methylbenzoic acid	C8 H8 O2	136.05225	6.069	135.04494	61015039.99	62846218.48	66263805.87	73586679.7	90674719.76	77922586.8	69036186.23	30329118.8	50317280.09	48661634.96	36736039.41	22900638.89	59435900.87	60900904.57	54054734.2
Com_1171_neg	Prostaglandin E1	--	C22 H39 N O5	433.26697	6.013	432.2597	89714705.95	990529.3618	3981190.614	2715840.923	1154338.853	1094529.198	619547.9603	398285.5685	524602.9984	790784.5903	673666.7503	707438.791	9231143.935	6439105.424	7524060.627
Com_1189_neg	N-(1,2,3,4-tetrahydro-1-naphthalenyl)benzenesulfonamide	--	C16 H17 N O2 S	287.10019	5.182	286.09302	39137648.14	57689827.66	65543272.23	75188131.55	54768934.3	70115174.23	6028056.715	9833802.85	5240149.256	8203550.835	4926706.963	10285191.61	38057290.1	40560108.58	42354021.31
Com_1211_neg	Propylparaben	--	C10 H12 O3	180.07838	5.425	179.07106	49480174.79	45744946.81	23085171.68	49101828.25	15836267.8	47732686.67	38782748.13	60233323.09	52034278	49311109.02	46171167.46	70800649.49	46134611.68	52679055.56	42393865.65
Com_1214_neg	2-Hydroxymyristic acid	--	C14 H28 O3	198.198	9.003	197.19084	86510948.93	96201987.83	48729859.69	39542583.87	74718400.82	63182463.53	31474321.17	53516666.91	36810060.94	23028669.29	32566630.18	48421989.9	51754147.89	48139239.87	48242176.26
Com_1270_neg	Vanillyl alcohol	Vanillyl alcohol; 4-Hydroxy-3-methoxy-benzenemethanol; 4-Hydroxy-3-methoxybenzyl alcohol; 4-Hydroxy-3-	C8 H10 O3	154.06272	5.32	153.0554	26468184.24	37408986.54	27565904.72	23490416.63	20648745.73	30467546.85	49660267.67	67144312.44	44148595.67	23550460.35	42426605.68	64363174.86	40785275.17	43381138.96	44224004.23
Com_1292_neg	FAHFA (2:0/23:0)	--	C25 H48 O4	412.35495	10.407	411.34781	63865891.5	81521210.61	75972743.37	69322064.93	78754375.98	49271570.72	1312097.61	893511.4822	532434.6493	734420.1626	968821.3078	704035.2412	32116426.52	31058131.85	27551842.18
Com_1325_neg	Sepiapterin	Sepiapterin	C9 H11 N5 O3	237.08511	1.425	236.07729	47932144.84	40970194.65	26407268.27	24546270.64	58517467.76	68394976.26	84271871.65	63600279.71	37303359.62	18253218.69	22395418.41	39880548.69	26065582.09	50759758.52	41853236.04
Com_1331_neg	Tretinoin	--	C20 H28 O2	300.2086	9.208	299.20145	1741977.107	1234406.12	875025.5204	828975.3378	1065627.78	1717539.035	12565043.55	6397682.17	8188026.245	8988912.106	60997776.46	10602358.62	11215555.02	9466451.105	9751087.722
Com_1338_neg	Erythronolactone	--	C4 H6 O4	118.02663	2.683	117.01931	54227310.08	48797127.54	46402664.39	46317706.47	77591584.93	56645717.99	11964409.75	17026233.91	14061407.72	31529608.23	12949734.77	15999493.15	31385054.61	34872924.36	28440639.22
Com_1340_neg	FAHFA (18:0/7:0)	--	C25 H48 O4	412.35499	8.906	411.34781	20697528.7	16723987.73	34889252.55	66225742.03	25134796.3	20287462.73	1994838.148	386381.8429	1035767.675	1393421.364	2091371.612	405805.1188	14871416.08	19780795.84	17301606.39
Com_1344_neg	2-(1-benzothiophen-3-ylmethylene)-3,4-dihydronaphthalen-1(2H)-one	--	C19 H14 O S	145.03817	1.378	289.06918	9306527.484	5384388.458	7873142.965	12690755.26	13590175	5841666.802	18890394.58	23134302.09	56094346.08	49283796.16	36236251.4	35551678.4	23722016.11	31696704.82	26833382.08
Com_1356_neg	Δ17-6-keto prostaglandin F1α	--	C20 H32 O6	368.2198	6.726	367.21252	14587340.89	13208378.19	12182469.63	10394229.03	11824168.65	12209230.47	14535199.2	8902728.741	9375458.347	10875039.07	59740040.62	11855165.25	16105538.04	14577525.33	12784007.72
Com_1435_neg	5,6-dihydroxyindole	--	C8 H7 N O2	149.04745	5.203	148.04005	38534244.47	34853448.68	45044065.82	47133816.89	33998525.5	43060461.96	34859112.38	41281716.97	48060191.56	44972817.6	39083927.14	34585558.56	43487200.7	36911374.42	33873146.57
Com_1440_neg	2,3-Dinor-TXB2	--	C18 H30 O6	342.20381	5.98	341.19653	46552188.89	41296140.2	34833213.26	37832335.73	41136133.3	41503726.35	32432723.97	30781315.55	32459980.91	44717480.75	28185104.16	26789037.18	34524648.72	37181515.9	29834257.44
Com_1446_neg	5-hex-1-ynyl-2-furoic acid	--	C11 H12 O3	192.07841	5.671	191.07112	7774091.01	11070621.75	12647258.62	10166686.37	5274854.55	9480049.74	27184192.12	56630451.12	33896361.59	19257120.49	37852387.93	41050477	16740815.36	16500672.98	14286695.46
Com_1457_neg	3-Methyl-2-oxobutanoic acid	3-Methyl-2-oxobutanoic acid; 3-Methyl-2-oxobutyric acid; 3-Methyl-2-oxobutanoate; 2-Oxo-3-methylbutanoate; 2-Oxoisovalerate; 2-Oxoisopentanoate; alpha-Ketovaline; 2-Ketovaline; 2-	C5 H8 O3	116.04717	4.896	115.0399	9520124.815	8384996.552	6649699.171	8367726.499	9013083.112	9398870.285	14653422.72	19152268.36	19529038.37	29592575.55	54210421.85	24552813.5	14074067.4	20729062.94	13556994.22
Com_1490_neg	Pyridoxine	Pyridoxine; Pyridoxol	C8 H11 N O3	169.07374	4.988	168.0665	60607538.88	54651643.23	49462784.04	47845297.55	67639133.21	38893107.49	8491892.497	7223325.59	5045883.19	8892622.908	5700063.872	12485930.08	24015392.51	26795964.58	21809889.34
Com_1517_neg	20-Hydroxy-(5Z,8Z,11Z,14Z)-eicosatetraenoic acid	--	C20 H32 O3	320.23471	8.318	319.22729	5706118.056	4445001.722	5495159.682	5972854.683	7436947.197	4505830.988	24625090.54	53621021.07	23295736.27	38168794.16	20624350.93	32842315.32	18865750.97	20222594.3	19754139.76
Com_1542_neg	Calcitriol	Calcitriol; 1alpha,25-Dihydroxyvitamin D3; (1S,3R,5Z,7E)-9,10-Seco-5,7,10(19)-cholestatriene-1,3,25-triol	C27 H44 O3	416.32877	10.256	415.3215	31785369.89	29466268.27	62909397.08	45972719.92	42587051.95	37205511.85	32526727.04	26324305.27	29112815.5	35174543.18	32066237.61	17354957.97	37104635.94	33756588.52	36328698.02
Com_1545_neg	Prostaglandin A1 ethyl ester	--	C22 H36 O4	346.25047	8.524	345.24319	14053556.86	8154434.528	9576998.546	10869570.44	12537832.68	8665324.187	10788102.72	7269007.721	13050473.26	41418893.23	11116561.5	6521285.108	14444777.76	14789222.77	14321804.56
Com_1590_neg	DL-3,4-Dihydroxyphenyl	--	C8 H10 O4	170.05774	5.168	169.05057	31786841.13	32900025.81	35011445.79	32910292.53	32020510.15	26453911.33	17809578.57	20129557.92	19622585.66	23224160.49	49233282.55	16990968.31	24134942.2	25058933.18	24335369.21
Com_1603_neg	2-Hydroxyisocaproic Acid	--	C6 H12 O3	132.07834	5.801	131.07106	4775180.869	4036001.052	17481235.21	10110056.04	6608740.299	3488967.429	4723461.258	28164837.09	4921378.018	5258516.236	48496354.19	4363318.036	9923887.937	8363261.251	11844356.58
Com_1636_neg	1-(4-Methoxyphenyl)-2-propanone	--	C10 H12 O2	164.08358	5.498	163.07623	36919117.57	41478003.11	58998189.97	50119149.82	54262336.93	38581531.72	13758264.09	5991547.447	4628029.738	6651218.01	11665575.89	5904908.425	23057723.42	27840703.84	20090142.35
Com_1643_neg	8-iso-15-keto Prostaglandin E2	--	C20 H30 O5	332.1986	7.872	331.19109	5046604.868	3944805.003	3932470.915	3290977.883	5047868.362	4675345.765	35052428.46	49206050.58	42106446.67	28140004.58	27996104.01	39557829.37	22956825.12	23369073.64	19729546.73
Com_1649_neg	Thromboxane B3	--	C20 H32 O6	322.21397	7.449	321.20679	5007058.692	4936754.59	4486216.637	4454700.416	6764374.706	5361892.14	23010627.75	16198779.19	17356475.73	16233463.12	47241924.24	17012637.1	15176204.62	14561774.43	13203352.04
Com_1690_neg	Xanthine	Xanthine	C5 H4 N4 O2	152.03314	1.425	151.02586	13475805.06	11369485.27	8422748.127	7282493.539	12070862.83	20710016.49	6330838.11	47935539.92	23731880.55	26767519.18	68909006.34	17348070.86	93756679.27	13724718.19	11116554.54
Com_1699_neg	Prostaglandin F3α	Prostaglandin F3alpha	C20 H32 O5	398.23018	6.212	397.2229	56278974.06	54168992.86	46599170.62	45094580.7	34997056	44358607.56	34011494.03	25383666.83	26838960.9	31291676.62	28759633.6	25313847.82	49608199.64	37968699.65	36919854.88
Com_1701_neg	D-3-Phenyllactic acid	--	C9 H10 O3	166.06347	5.817	331.11966	1214898.702	1995783.857	1522459.039	1200528.171	1483351.388	2262225.49	5374807.67	29032601.08	34695486.22	25715995.34	1212823.225	37897064.62	982436.4134	1051689.561	1447093.969
Com_1724_neg	4,6-dimethyl-2-(methylamino)-5-nitronicotinonitrile	--	C9 H10 N4 O2	206.07882	1.425	205.07155	13482498.54	15280533.56	14801925.42	11291428.51	19139766.79	24072292.98	11015899.25	39353241.98	18991413.82	13385758.2	17036549.46	47054813	15653025.8	17638126.52	13683831.88
Com_1740_neg	4-Methylphenol	4-Cresol; p-Cresol; 4-Hydroxytoluene; 4-Methylphenol	C7 H8 O	108.05744	5.344	107.0502	2713326.043	3552170.022	2562867.298	2457988.061	2352238.263	2493295.382	7286375.27	12470096.62	7718286.707	7678595.348	44513226.44	13347802.82	6413850.692	6568100.948	6924838.793

Com_1754_neg	Homogentisic Acid	Homogentisate; Homogentisic acid; 2,5-Dihydroxyphenylacetic acid; 2,5-Dihydroxyphenylacetate	C8 H8 O4	168.04209	5.588	167.03479	31393090.27	32699879.86	33438947.58	48217959.08	20965718.35	21701856.98	7733737.8	10541544.44	7381492.128	7328993.986	8791069.896	9151217.723	17444161.74	19383745.48	18551823.06
Com_1795_neg	11(E)-Eicosenoic Acid	--	C20 H38 O2	310.28706	11.333	309.27975	15644049.22	14644906.2	14700608.78	6784350.446	21190653.52	7596943.701	38047983.91	44576509	23658556.76	10684935.13	26270524.4	43993845.15	25662837.08	28594481.1	27862321.18
Com_1853_neg	1-Caffeoylquinic Acid	--	C16 H18 O9	354.09476	5.649	353.08792	38727186.02	46861924.82	50957391.57	38583624.06	39182353.42	15519087.88	19292924.65	5293455.227	11026253.59	13123820.08	12630707.1	18535643.97	31066668.32	36514845.52	32250694.05
Com_1988_neg	12-Hydroxydodecanoic acid	12-Hydroxydodecanoic acid; omega-Hydroxydodecanoic acid	C12 H24 O3	170.16675	8.13	169.15926	39440990.32	41438961.27	24965915.65	23934514.41	38165761.54	32272180.9	15895873.58	39363512.54	26181692.62	22955790.24	19575234.16	35354199.61	29982660.88	30369877.18	30805416.97
Com_2038_neg	Hippuric acid	Hippurate; Hippuric acid; N-Benzoylglycine; Benzoylaminoacetic acid	C9 H9 N O3	179.05797	5.251	178.0507	31424302.97	47603078.25	40829716.5	34386942.29	44280400.22	38055059.14	35157214.1	6220685.903	23408191.81	11159410.73	29018815.76	7212801.288	39949233.9	30191067.35	27596431.01
Com_2054_neg	LPC 18:2	--	C26 H50 N O7 P	565.33742	9.191	564.3302	5646176.358	6678750.6	6621640.364	2453467.461	4449850.27	3837267.748	6245552.413	37798915.05	4540807.979	1348810.952	3510784.175	3118220.229	7813618.282	6178654.577	7479456.743
Com_2065_neg	Prostaglandin A3	--	C20 H28 O4	664.39785	7.877	663.39087	233188.1745	270345.0324	273084.454	250697.8139	251934.4139	256533.3882	18310216.22	37642105.55	27623875.78	14349111.73	20042751.36	27772306.92	14005705.85	12417716.65	11604427.18
Com_2133_neg	3,4-Dihydroxyphenylpropionic acid	3,4-Dihydroxyphenylpropanoate; Dihydrocaffeic acid; 3,4-Dihydroxyphenylpropionic acid; 3-(3,4-Dihydroxyphenyl)propanoat	C9 H10 O4	200.06826	5.43	199.06087	22421110.86	26581486.34	24026619.81	37846815.28	16554452.17	28670607.19	30172954.56	18048241.65	26483964.06	15063582.81	19316591.29	20418949.17	22747411.49	15876621.7	17414320.74
Com_2171_neg	Lauric acid ethyl ester	--	C14 H28 O2	228.20863	9.605	227.20137	35658010.46	39666141.48	31788390.6	36996010.3	29166720.56	37569279.18	16497118.64	26778605.47	23715300.8	17543132.15	18563156.09	29622559.42	24333843.12	25630389.28	23905326.74
Com_2178_neg	Prolylleucine	--	C11 H20 N2 O3	228.1472	5.021	227.13995	5198534.209	5131292.661	3116208.794	4108077.879	13069909.99	12160298.23	1240170.39	3993393.988	4853784.671	27623258.6	1415324.684	2931639.362	7666362.015	8420574.785	7254139.227
Com_2188_neg	2,3-dinor Prostaglandin E1	--	C18 H30 O5	308.19843	6.776	307.19104	42927075.82	35855455.63	20845394.2	18094460.31	24644515.76	24141620.08	9480750.179	13526296.46	10852324.26	19257146.14	28121993.86	11328669.3	22370420.44	23891396.92	21439312.55
Com_2205_neg	3,8,9-trihydroxy-10-propyl-3,4,5,8,9,10-hexahydro-2H-oxecin-2-one	--	C12 H20 O5	244.13072	6.171	243.12358	35482839.15	37747172.15	41110397.05	31255437.11	36182284.6	34572295.52	20077943.7	14794719.45	18374729.92	20723640.95	15602657.62	16170420.12	26511636.45	28364873.53	25555509.35
Com_2270_neg	Epinephrine bitartrate	--	C13 H19 N O9	333.10571	5.279	314.08792	28937608.32	40477802.5	39621461.78	24620862.92	30952574.49	35877314.89	8258522.908	5526863.112	5108722.974	6182306.189	7261151.059	6862765.24	19349701.17	19203571.81	20492877.89
Com_2314_neg	Genipin	Genipin	C11 H14 O5	208.07328	5.254	207.06593	27618891.23	28761667.38	38645777.75	32496209.64	17378711.19	33913373.83	23315902.19	15687474.51	14885653.38	7944707.922	11626731.11	24828222.36	28524863.56	21805684.11	20481389.21
Com_2365_neg	N1-[4-(cyanomethyl)phenyl]-4-chlorobenzamide	--	C15 H11 Cl N2 O	270.05259	8.115	269.04535	1027261.416	1102938.51	1772143.54	1416222.884	1119710.41	1311803.029	1530608.899	3502251.952	4773007.249	2459176.387	30284532.41	3977396.471	4858465.238	5081058.386	4243170.285
Com_2481_neg	(±)8(9)-DiHET	--	C20 H34 O4	338.2455	10.506	337.23822	9200956.74	4594900.67	5158202.923	5167213.24	4769593.12	2033693.653	20080548.75	13059032.52	12274281.99	23491138.16	9397407.745	20725122.61	15708797.19	9909513.323	10318231.49
Com_2504_neg	DGMG (18:2)	--	C33 H58 O14	678.38273	8.92	677.3739	756039.9639	1600810.724	1447197.794	1481496.042	1273339.851	1026313.266	24641174.59	22081025.13	30417976.38	632127.7695	13521038.98	19435030.27	12490506.9	9648453.962	9943794.7
Com_2507_neg	D-(+)-Maltose	--	C12 H22 O11	388.12138	1.299	387.11411	1117539.592	144722.3842	2493123.018	329764.6094	1201162.568	1628601.009	301076.5542	955622.1606	30345344.43	2846846.431	3978315.004	18657362.58	7416501.011	7474597.441	7420907.614
Com_2537_neg	D-Threose	D-Threose; D-threo-Tetrose	C4 H8 O4	120.04205	1.354	119.03466	21173600.99	9461779.349	14729872.77	12812557.44	12376831.04	16206797.38	15494675.41	28872584.36	13086279.13	15306934.22	21423744.48	16254581.24	15279965.81	15667061.31	15550565.07
Com_2543_neg	5-[4-(2-furylcarbonyl)piperazino]-5-oxo-3-phenylpentanoic acid	--	C20 H22 N2 O5	370.14723	1.447	369.14008	798248.0503	780534.2029	1557441.567	1245324.212	1076055.751	1000717.907	5063375.591	5831034.795	7199627.128	2420608.621	27701323.93	3719778.105	1164670.907	1137982.708	1093896.309
Com_2581_neg	Protectin D1	--	C22 H32 O4	360.22723	8.09	359.21982	703651.6953	1051990.977	985670.3074	799480.9057	827343.4028	827348.8242	31931974.28	27899227.98	24444237.61	18849595.78	14107584.04	23998252.55	15701064.7	16577211.56	15648778.12
Com_2592_neg	Pyrogallol	1,2,3-Trihydroxybenzene; Pyrogallol; Pyrogallic acid; 1,2,3-Benzenetriol	C6 H6 O3	126.03144	5.153	125.02403	33918968.94	39582365.54	16596028.93	10302757.02	11884122.86	15408761.13	7988759.842	8803104.773	7034827.044	7259748.085	5925072.103	10164343.66	11724306.16	19145720.97	11725983.71
Com_2650_neg	FAHFA (19:1/6:0)	--	C25 H46 O4	410.3392	9.635	409.33194	22743289.68	21171335.85	21078995.98	28524701.67	18431231.72	14147472.25	2734656.562	4150650.71	1574483.463	1712477.505	2475523.703	3240515.05	11800534.79	10194868.48	9560821.58
Com_2653_neg	N1-(6-methyl-4-oxo-3,4-dihydroquinazolin-2-yl)-4-nitrobenzamide	--	C16 H12 N4 O4	324.08429	5.976	323.07697	14661728.2	20125708.43	17171307.95	19046378.77	9164549.084	16395935.92	13953233.38	14602889.26	14331713.97	21325652.39	10696351.36	14051505.01	17201274.21	17671557.82	13891130.68
Com_2666_neg	7-Hydroxy-3,4-dihydrocarbostyril	--	C9 H9 N O2	163.06313	5.395	162.056	29074148.73	38065616.74	16837052.63	14980519.48	22610316.87	28216014.92	12826607.38	9566127.133	14563859.3	12085347.62	7825098.593	19074077.44	17752075.73	18643039.98	15170241.63
Com_2767_neg	N-Tigloylglycine	--	C7 H11 N O3	157.07368	4.867	156.06653	31839832.39	20563247.02	17598231.16	25150011.84	15931985.41	14554748.78	14612315.17	2730112.335	1555118.238	1679039.4	5440619.058	2402886.225	11609024.84	10315814.39	9290683.717
Com_2912_neg	Lactobionic acid	--	C12 H22 O12	358.11119	1.472	357.10379	6211302.944	6172685.516	5858531.247	4433464.322	12034797.72	15844987.76	4203682.008	4431549.426	4308672.053	19180176.11	12692997.66	3752706.506	6839570.188	7785793.362	8463457.425
Com_2940_neg	(±)9(10)-EpOME	--	C18 H32 O3	342.24009	7.091	341.23288	29772553.99	9324592.942	5992753.361	15006111.63	7271911.002	6957547.142	7652390.853	7178882.841	9199980.335	9425254.908	12212347.31	5182440.437	11270019.94	9891009.648	12192412.9
Com_2952_neg	4,5-diphenyl-2,3-dihydro-1H-pyrazolo[3,4-c]pyridazin-3-one	--	C17 H12 N4 O	288.09943	5.611	287.09213	20401909.57	19847734.62	22932333.82	17874032.31	15237393.11	17057540.73	20874449.08	24013549.37	21874139.99	15683095.32	17365071.32	21397559.08	16654048.41	19767200.27	16825541.67
Com_2990_neg	L-Ascorbate	Ascorbate; Ascorbic acid; L-Ascorbate; L-Ascorbic acid; Vitamin C	C6 H8 O6	176.03192	1.478	351.05661	3327634.754	3214883.204	8395641.423	5249017.65	6064874.812	4562325.588	6460173.193	23601306.62	6809713.995	1517715.203	4306222.233	13627087.18	5941186.069	5009624.972	5082319.884
Com_3003_neg	Allantoin	Allantoin; 5-Ureidohydantoin;	C4 H6 N4 O3	158.04373	1.366	157.03629	1003617.295	1288232.471	2406687.692	1742859.163	2075553.838	1881955.895	2424282.85	23452647	6536085.197	9391754.549	8718320.672	21814107.93	6547204.06	6013887.764	4365161.713
Com_3012_neg	O-Phospho-L-serine	O-Phospho-L-serine; L-O-Phosphoserine; 3-Phosphoserine;	C3 H8 N O6 P	185.00872	1.288	184.00128	1335005.068	1415414.215	2321968.818	2145995.856	2620705.131	4257365.874	7814137.787	21111479.95	10452101.2	18357913.34	4495412.063	20654328.56	9459930.304	10690093.99	10485026.66
Com_3015_neg	Dl-3,4-Dihydroxymandelic	--	C8 H8 O5	184.03676	5.014	183.02942	27585261.56	32851498.24	18483203.55	11721749.14	22926283.43	6778170.783	8894827.824	8950609.1	8072143.296	6942491.947	6178741.656	9617896.749	17310973.66	15759223.93	16794350.37
Com_3019_neg	4-morpholino-3-nitrobenzene-1-sulfonamide	--	C10 H13 N3 O5 S	288.06068	1.657	287.0535	799963.8237	710835.1509	875043.6825	1165180.134	721851.6493	892276.6153	21340840.94	16338434.97	7489232.741	1268549.426	4912957.573	23404210.03	3319963.744	3381925.334	3384506.526
Com_3025_neg	8-iso Prostaglandin A2	--	C20 H30 O4	316.20346	8.032	315.19614	3422143.741	3243412.045	8682879.6	9054494.613	4428466.928	4107427.381	9493692.325	11857060.44	17284811.28	18274616.58	6283948.591	8519052.359	8317899.797	9516342.546	9050092.714
Com_3055_neg	Corey Lactone Diol	--	C8 H12 O4	172.07334	5.292	171.06604	18382554.21	32163641.62	26563039.49	21877732.46	24915892.84	23990577.53	15450537.84	14444428.08	10758026.96	16183546.32	14952071.75	14926515.2	16658373.04	20413178.95	20048574.29
Com_3119_neg	Ascorbic acid	Ascorbate; Ascorbic acid; L-Ascorbate; L-Ascorbic acid; Vitamin C	C6 H8 O6	176.03186	1.979	175.02452	23995849.09	25300205.66	24844475.21	23392845.87	26464906.89	15678814.57	1639453.823	1092485.271	1198616.579	1179196.149	1312385.516	1585593.201	10119002.09	9086827.813	10084844.78
Com_3149_neg	2-[(carboxymethyl)(methyl)amino]-5-methoxybenzoic acid	--	C11 H13 N O5	193.07365	5.248	192.06645	27049623.26	27034757.16	18732795.44	22525638.21	24991728.39	25312934.99	5276817.808	5693642.868	8133678.966	5541170.765	14948823.39	8525224.543	16330723.4	17873459.3	14526485.5
Com_3188_neg	tetranor-PGDM	--	C16 H24 O7	310.14454	7.064	309.13733	21315442.71	14664403.55	24870186.21	22634993.31	12099989.51	14838126.87	5280439.56	3187392.814	3484213.508	5588822.566	2934377.66	2891867.708	9764787.021	10276295.31	12139808.39
Com_3207_neg	LPG 17:0	--	C23 H47 O9 P	498.29813	7.916	497.29083	2098051.808	2841229.298	2779623.185	3514052.976	2729769.607	3667895.396	4866996.052	3902755.086	15417254.84	2843288.002	20563434.48	5972918.328	6179916.967	5128636.901	6455255.987
Com_3236_neg	4-Ethylphenol	4-Ethylphenol; p-	C8 H10 O	122.07285	5.85	121.06555	2405025.096	2669435.447	1803447.111	2891589.728	1575971.248	2006269.84	23795221.22	5515560.217	8158196.656	9256114.3	2132845.689	4709932.354	4094580.881	3643561.051	4125957.929
Com_3338_neg	Pyridoxine O-Glucoside	--	C14 H21 N O8	331.12594	4.954	330.11813	13688495.3	28336419.34	19163036.81	11866827.61	7886825.706	10271311.62	5119073.932	1996673.299	2041003.21	1300764.54	915445.8467	2925208.429	12534127.02	8521751.436	12718536.35
Com_3344_neg	Decanoic acid	Decanoic acid; Decanoate; Decylic acid; n-Capric acid	C10 H20 O2	172.14625	6.759	171.13899	1515564.078	1646426.274	1298498.112	1303317.325	1364591.329	1255644.899	3164155.533	981186.4066	655562.9925	855025.1464	19290406.11	965957.4372	2023817.362	1993746.543	1781893.411
Com_3397_neg	2-Hydroxy-2-methyl-3-buten-1-yl beta-D-glucopyranoside	--	C11 H20 O7	218.11504	4.994	217.10782	7994361.046	8851506.582	7984614.108	6685282.991	7376094.813	7080139.65	12101688.4	14902878.15	14045083.71	15471535.58	11927810.41	19318558.43	10561827.77	11539899.01	9717692.413
Com_3446_neg	Isorhamnetin	Isorhamnetin; 3-Methylquercetin; Quercetin 3'-methyl ether	C16 H12 O7	316.05814	6.119	315.0509	3972192.711	6731878.895	5874671.251	4224639.648	3476250.355	4803497.143	13781854.09	19282641.1	16005037.66	9259286.223	7953340.194	18793533.09	5390785.305	5850615.251	5504069.187
Com_3483_neg	Benzyl 6-O-beta-D-glucopyranosyl-beta-D-glucopyranoside	--	C19 H28 O11	432.16282	5.586	431.15543	1141986.631	4708603.246	1191912.416	1239027.612	1293031.226	1366026.461	10543975.57	19025029.15	12981012.77	3536280.928	10527671.29	15756044.14	4611261.536	4271776.843	2998684.135
Com_3526_neg	(2S)-4-Oxo-2-phenyl-3,4-dihydro-2H-chromen-7-yl beta-D-glucopyranoside	--	C21 H22 O8	402.1341	6.684	401.12677	3059846.667	2464162.116	1604394.765	1360269.77	2110084.308	2254429.56	6949784.22	6185218.142	10699461.75	14701931.91	11467445.65	7307048.558	5011477.441	4837284.567	5925509.726
Com_3552_neg	5-Hydroxytryptophol	--	C10 H11 N O2	177.07873	5.131	176.07141	14194383.07	12367213.04	10415056.46	16448969.06	10286809.68	13357047.53	7591417.632	15130106.93	14318597.57	14578038.32	17017089.9	13108852.36	11603932.41	9828610.743	11203994.2
Com_3555_neg	Maltotetraose	Maltotetraose	C24 H42 O21	666.22194	1.379	665.21454	5171715.766	2547010.957	5123208.27	3854905.511	7215426.116	6341981.159	6078753.353	18474162.72	11427713.94	4182607.164	17597760.06	8606563.714	5740478.842	7602303.272	5548893.74
Com_3586_neg	5-Dehydroquinic acid	3-Dehydroquinate; 3-Dehydroquinic acid; 5-Dehydroquinate; 5-Dehydroquinic acid	C7 H10 O6	190.0475	4.754	189.04022	20154667.07	21299621.03	20955396.21	14026098.1	19773747.82	18102900.76	6443381.954	6234149.469	3213449.282	14370667.59	2388627.272	13882192.38	11041411.28	11183187.21	9023643.886
Com_3639_neg	FAHFA (2:0/24:4)	--	C26 H42 O4	418.31035	10.156	417.30307	451871.3756	17283849.8	13048292.88	12535221.05	395696.5323	23848637.06	4258610.151	4509244.359	1619423.815	558213.3725	37776.46873	4878191.905	5552101.713	8420118.024	8963847.21
Com_3673_neg	Geranylgeranyl pyrophosphate	Geranylgeranyl diphosphate; Geranylgeranyl pyrophosphate; all-trans-Geranylgeranyl diphosphate; all-trans-Geranylgeranyl pyrophosphate; (2E,6E,10E)-3,7,11,15-Tetramethylhexadeca-2,6,10,14-tetraen-1-yl	C20 H36 O7 P2	450.18837	5.603	449.18109	17576185.84	24588656.37	16804955.09	11054156.08	21699245.22	21652808.89	4192639.789	2727898.838	1960955.997	1730535.129	2352177.104	2758047.438	11493426.97	8332063.528	10610290.93
Com_3698_neg	ethyl 3-hydroxy-4,6-dimethoxy-2-oxoindoline-3-	--	C13 H15 N O6	281.08962	5.2	280.08237	13575119.95	18697024.03	20838204.74	14408336.93	21343384.31	11991312.96	2635738.939	2160603.807	2380527.868	1946755.27	2213727.523	2314386.7	12094303.1	10797368.77	14410694.62
Com_3707_neg	gamma-Glutamylleucine	--	C11 H20 N2 O5	260.13663	5.334	259.12933	3304559.528	1208144.16	5925202.002	3497727.346	5747350.168	23227710.22	525018.8139	1218988.837	737667.2782	2813356.619	1080742.827	1346578.398	6594734.84	4996118.85	4379148.32
Com_3712_neg	LPC 20:0	--	C28 H58 N O7 P	597.40059	10.421	596.39355	234101.8541	297390.6231	250937.5511	208449.8137	247905.9967	239570.355	11055072.77	10324117.89	2232921.414	3890946.338	16741709.41	12177845.18	5110844.522	5299456.025	5222700.632
Com_3854_neg	Gly-Tyr	--	C11 H14 N2 O4	238.09517	5.214	237.08781	7836870.508	6395573.655	9884311.751	17331410.3	7829309.65	8500194.979	381070.0977	509524.3826	422353.5027	469281.787	365358.0484	499310.1656	3942089.433	4427764.587	4504245.128
Com_3898_neg	(+/-)19(20)-DiHDPA	--	C22 H34 O4	362.24531	7.815	361.23807	9738538.026	8079485.889	11989861.42	15698908.92	9944077.751	9539752.381	7198244.193	4085081.35	9763561.57	12752915.63	5691524.021	4025388.659	8475861.864	9027626.554	8501362.861
Com_3904_neg	19(R)-hydroxy Prostaglandin	--	C20 H30 O5	332.19858	7.096	331.19135	12244238.93	13402416.31	15021196.78	16111443.5	19891801.88	17392593.42	6090494.391	7170806.492	7981207.45	8565190.89	7160909.441	6803758.925	10947864.95	11495863.86	11303281.68
Com_3909_neg	2-(1H-1,2,3-benzotriazol-1-yl)-N-(2,3-dihydro-1H-inden-2-yl)acetamide	--	C17 H16 N4 O	292.13085	6.652	291.1236	5956901.443	6478348.889	15114929.82	16974205.02	5310960.847	6466423.74	888717.1961	1049876.297	933922.2606	754609.2937	906561.664	1267902.14	7252784.649	7108373.131	7087178.278
Com_3922_neg	Methyl-beta-galactopyranoside	--	C7 H14 O6	194.07872	1.415	175.0609	12574527.38	13335387.11	14782723.51	15370926.85	14910026.62	15075193.46	11595494.09	16044625.68	12944680.69	12280084.89	14512333.1	11939256.59	9529609.796	11015900.01	11563547.14
Com_3971_neg	10-Undecenoic acid	--	C11 H20 O2	184.14609	6.623	183.13879	12997058.16	12908181.16	13027642.58	10114760.24	19439667.48	16945185.64	9711995.704	11349799.71	10013369.05	9693100.286	8561634.267	11170616.95	13503006.7	12712892.85	13505381.86
Com_4020_neg	(±)7(8)-DiHDPA	--	C22 H34 O4	362.24533	7.996	361.23801	7533797.485	5766227.232	4265014.469	4689113.497	6147884.118	4908479.463	11139334.07	5251347.017	11358146.08	12210887.36	8364077.337	5099397.158	9178398.521	8456181.739	7923480.194
Com_4050_neg	Hydroquinone	Hydroquinone; p-Benzenediol; 1,4-Benzenediol; 1,4-Dihydroxybenzene; Benzene-1,4-diol; Quinol; 4-	C6 H6 O2	110.03654	7.543	109.02931	2989020.963	2090622.931	2427797.387	2528471.09	1831853.337	2045685.874	4603170.505	10389048.28	7017000.979	11855740.23	10118948.03	15434439.8	7291768.092	5451642.826	6655255.656
Com_4122_neg	N-gamma-Acetyl-N-2-Formyl-5-Methoxykynurenamine	--	C13 H16 N2 O4	264.11086	5.258	263.10364	8077455.096	19103983.79	14945020.21	15666508.54	7754822.096	16715043.13	7174880.546	6848895.223	5476636.726	6496387.437	8808891.356	7284312.419	11522188.41	11997398.57	11114629.1
Com_4185_neg	LPC 18:1	--	C26 H52 N O7 P	567.35327	9.695	566.34613	5754634.485	13206098.41	17381091.13	3866690.956	10233996.67	6313232.544	1534930.235	1578333.203	727295.1836	755311.0894	1125004.335	895349.6562	3017077.961	3161386.499	3544855.684
Com_4248_neg	Geniposidic acid	Geniposidic acid	C16 H22 O10	374.12089	5.054	373.11362	2607108.615	3221797.598	2754962.145	3224469.175	2711039.914	2091247.803	1523811.55	14161326.46	1190803.711	1561775.82	825334.1704	7346319.671	2643720.663	2717936.623	3177889.177
Com_4274_neg	N'-(4-chlorophenyl)-4-ethylbenzohydrazide	--	C15 H15 Cl N2 O	274.08391	6.577	273.07657	17258814.58	15941737.66	11387604.74	6957750.235	15551503.47	14212559.38	4061182.282	3366596.054	2125156.641	1141213.537	5424200.676	3667736.733	7032282.639	7035430.32	8114202.964
Com_4275_neg	Lactitol	--	C12 H24 O11	344.13138	1.355	343.12399	481122.9383	473303.8867	937500.5406	601149.7047	602750.864	489282.8469	1828378.365	4703995.392	1254221.542	565674.4947	13493951.96	1866488.571	436383.0624	629813.0284	541495.646
Com_4280_neg	Nonadecanoic acid	Nonadecanoic acid; Nonadecylic acid	C19 H38 O2	298.28675	10.996	297.27939	5707934.873	9546491.079	6855146.218	4725355.431	7064560.23	5114776.932	10231495.79	9932794.676	7678146.042	11010166.31	7520173.263	8886370.872	8378645.476	8802595.695	7495354.928
Com_4360_neg	7-Hydroxy-4-chromone	--	C9 H6 O3	162.0314	5.379	161.02429	12338213.29	9832464.463	12534552.44	12145868.94	5174388.75	14660286.76	6433111.355	5727268.825	8274149.699	10675920.13	7588587.192	8537013.554	9192607.444	12727518.59	11125317.78
Com_4362_neg	N6-Succinyl Adenosine	--	C14 H17 N5 O8	383.10716	5.06	382.09988	10231255.22	6361293.079	6313715.71	4849468.107	16670957.18	10306271.25	1031869.184	1505375.846	1249652.14	1436100.107	980101.7483	2097007.593	5899750.837	5557173.198	6080132.589
Com_4416_neg	Hydroxyglutaric acid	--	C5 H8 O5	148.03694	1.791	147.02966	5248830.772	4780611.541	9871011.563	10430353.93	16334966.94	8571784.903	4970481.505	6059151.955	3492478.061	6719944.494	4063733.386	3821984.517	10787297.13	7436336.126	7216319.768
Com_4455_neg	16-(Hexopyranosyloxy)-7-hydroxy-8,9-epoxypimaran-18-oic acid	--	C26 H42 O10	514.27571	7.865	513.26843	377009.2441	398182.4279	394537.8835	362194.8574	394640.5097	370625.7847	6230752.497	793843.2681	576135.7617	539623.8868	12646152.05	1439742.532	1208821.274	1211350.275	1279813.255
Com_4472_neg	(+/-)9(10)-EpOME	--	C18 H32 O3	342.23995	7.889	341.23315	2327248.414	2353798.981	1391495.184	1307517.902	1386015.011	1930858.928	13118613.66	13025385.69	9109014.747	6534414.932	4069941.926	8835902.916	7276078.588	6159020.743	5647038.922
Com_4544_neg	L-Fucose	6-Deoxy-L-galactose; L-Fucose	C6 H12 O5	164.06828	1.397	163.06126	8733876.722	4286449.595	3502423.286	5084692.754	3151290.276	4790233.307	3854539.445	8858259.988	2860910.781	2460886.991	12199353.05	4415627.407	5445864.658	4337651.771	5173242.405
Com_4558_neg	Chrysin	Chrysin; 5,7-	C15 H10 O4	254.05754	6.908	253.05028	715358.6034	642209.2128	696886.253	704459.9873	619637.1594	1273354.037	14236792.45	2962835.109	3769400.289	3775974.066	763978.1541	3307614.916	1863866.495	2555399.249	2110502.758
Com_4570_neg	5-Aminoimidazole-4-carboxamide-1-beta-d-ribofuranosyl 5-	--	C9 H15 N4 O8 P	338.06326	5.998	337.05591	10861941.11	12891063.78	14554404.44	13202583.15	12294601.27	13658651.07	6819386.019	4108302.959	5099820.612	3564594.147	4779761.027	5141816.996	8912583.247	6632162.961	7660658.621
Com_4593_neg	LysoPE 18:2	--	C23 H44 N O7 P	477.28609	8.974	476.27783	5353590.115	9901388.268	9882560.465	13061669.76	3386066.867	2615588.372	400820.2328	1392692.078	1017271.109	391868.9704	345364.7508	453163.4135	2857260.488	2232665.17	3199057.238
Com_4633_neg	6β-Prostaglandin I1	--	C20 H34 O5	376.22215	7.425	375.21487	428353.5158	696070.2326	546355.3447	1383988.733	443868.2722	503856.2375	1369301.811	2090650.804	927384.5408	492260.4034	11793892.73	1749282.664	2074671.022	2158722.402	2578295.209
Com_4660_neg	D-2-Aminoadipic acid	--	C6 H11 N O4	161.06851	1.38	160.06119	12726803.62	10290873.08	12963086.47	11825060.33	14726750.16	12180840.48	8647117.996	9922985.454	7526055.287	7260701.909	11683874.37	7846221.458	6749270.18	12390522.27	10126053.51
Com_4680_neg	Hydrocortisone	Cortisol; Hydrocortisone; 11beta,17alpha,21-Trihydroxy-4-pregnene-3,20-dione; Kendall's compound F; Reichstein's substance M	C21 H30 O5	362.21071	6.525	361.20343	13482400.71	13399428.66	10412587.28	8737851.347	14824833.8	15121922.72	6175206.817	5426781.506	8528503.18	6883651.034	8814901.9	6022780.822	9493963.6	8372297.47	6441029.936
Com_4725_neg	4-Oxoproline	4-Oxoproline; 4-Oxo-L-	C5 H7 N O3	129.04239	1.462	128.03511	14586443.21	9928279.407	8427360.172	6134332.548	2514832.204	8214780.807	2558277.739	7946776.644	3389555.88	4747942.104	2941017.528	3872411.778	6600606.278	6223615.877	6286858.345
Com_4785_neg	7-Methylxanthine	7-Methylxanthine	C6 H6 N4 O2	166.0488	3.085	165.04152	9539913.743	9638017.446	13227167.97	12158579.93	12327364.91	9579811.241	3489758.695	3087734.019	4374411.308	5367843.843	5258988.532	3921266.641	6265912.161	6481422.239	6224912.219
Com_4801_neg	Anacardic acid	6-Pentadecylsalicylic acid; Anacardic acid	C22 H36 O3	348.26595	10.089	347.2587	7630121.382	10594639.36	11330363.96	12102927.26	7839183.906	7645612.724	466167.192	436936.7089	327497.203	234247.0268	245029.9166	334272.7788	4287944.561	3525313.274	3912019.221
Com_4812_neg	N7-Methylguanosine	N7-Methylguanosine	C11 H17 N5 O5	299.12343	11.47	298.11603	406811.6046	336637.8656	242383.9498	392771.4405	838085.6625	357187.8682	12949466.07	6359374.573	508547.02	759113.7781	2497436.304	5028853.892	3422737.303	3569753.342	2969602.711
Com_4835_neg	N-Acetyl-L-histidine	N-Acetyl-L-histidine	C8 H11 N3 O3	197.08089	1.387	242.0793	2388123.847	2069077.222	2201886.375	1930959.105	2460693.131	3093626.403	4666703.102	11337870.19	4229867.379	1303663.68	3087476.613	7604480.778	2229587.449	2877274.91	3208133.39
Com_4987_neg	2-Furoylglycine	--	C7 H7 N O4	169.03708	6.066	168.0298	13358302.64	5892968.636	11952143.28	10419881.62	11284621.85	8767681.244	1267258.556	1690643.198	1210017.095	2121665.704	1452290.964	1083659.995	7052219.703	5539338.062	5648127.409
Com_4988_neg	Lipoxin B4	Lipoxin B4; LXB4; 5S,14R,15S-Trihydroxy-6E,8Z,10E,12E-eicosatetraenoic acid; (6E,8Z,10E,12E)-(5S,14R,15S)-Trihydroxyicosa-6,8,10,12-	C20 H32 O5	334.21439	8.652	333.20746	2451174.638	2089412.907	2278207.999	2328178.32	1780656.968	2168808.19	1590990.373	1125724.78	2151100.011	1971606.002	10440263.43	1007944.153	3159378.448	2293199.372	2522665.752
Com_5003_neg	2-Naphthol	2-Naphthol; beta-Naphthol	C10 H8 O	144.05713	5.657	143.04982	10240382.91	10397518.33	8110590.595	9795008.415	11585441.29	10336346.89	4175396.09	5537625.787	9132907.046	8501811.899	7330856.002	6130052.812	5595046.314	9666745.541	7998350.402
Com_5018_neg	5-Phenyl-N-(4-(trifluoromethyl)phenyl)oxazol-2-amine	--	C16 H11 F3 N2 O	304.07895	1.424	303.07175	6917602.57	7188988.594	10103431.28	11304785.15	8245010.041	7945509.691	2575743.238	3958856.241	2020802.639	2026954.41	1025886.178	3230707.411	4964286.596	6654864.438	5849320.398
Com_5019_neg	15-Deoxy-Δ12,14-prostaglandin D2	--	C20 H30 O4	316.20381	7.605	315.19653	1589010.927	1123894.159	1624417.421	1551073.908	1454206.088	1004853.424	7890797.797	3711884.157	5670254.173	5840055.064	10354282.05	5934364.651	4222483.266	4246753.951	3594953.173
Com_5094_neg	Dl-3-Hydroxy-kynurenine	--	C10 H12 N2 O4	224.07935	5.076	223.07179	10519854.37	9874886.755	9138407.728	10729176.18	11490891.18	14002498.34	4974573.504	3301673.527	670814.3351	1864945.839	5476794.981	3478825.428	9444263.71	6401017.623	7544480.034
Com_5131_neg	(3R)-4,4-Dimethyl-2-oxotetrahydro-3-furanyl beta-D-glucopyranoside	--	C12 H20 O8	338.11483	7.285	337.10742	657467.4773	607647.9756	738318.7415	764337.3878	666517.6409	671906.1766	4676082.48	6030607.422	7731348.282	8143070.62	5854166.798	3862767.988	3454058.388	2808945.249	2316314.632
Com_5190_neg	Bilirubin	Bilirubin	C33 H36 N4 O6	584.26466	7.571	583.25739	508168.7127	484159.3628	597439.421	715171.1509	920250.7463	536326.6727	6711459.851	6437135.694	7784269.038	7983891.413	4968221.958	5092341.524	2072678.163	2637440.54	2225783.016
Com_5199_neg	Catechol	Catechol; 1,2-Benzenediol; o-Benzenediol; 1,2-Dihydroxybenzene; Brenzcatechin; Pyrocatechol	C6 H6 O2	110.03666	5.907	109.0292	12009674.3	10975309.52	11515189.01	10638724.56	9839408.303	9807450.473	8871203.336	9004857.67	7453449.105	6186371.077	7346315.317	7585946.011	9067736.872	8263261.546	9026331.403
Com_5213_neg	4-oxo-4,5,6,7-tetrahydrobenzo[b]furan-3-carboxylic acid	--	C9 H8 O4	180.04205	5.461	179.03474	8870506.513	8409043.167	10942330.01	8143188.208	7895732.365	9370618.056	9226935.277	5148567.829	8611938.004	5233045.859	9702647.824	6224636.74	9512304.215	10690467.78	8985887.708
Com_5263_neg	Hydroxykynurenine	--	C10 H12 N2 O4	224.08331	5.723	223.07588	4142173.505	1500623.995	1241895.053	1533571.22	989262.2676	1023801.793	7693960.596	5513901.188	7513476.098	7832556.692	4238946.312	6935308.532	2090122.052	2641643.55	2740065.343
Com_5269_neg	FAHFA (18:1/20:3)	--	C38 H66 O4	586.49361	10.577	585.48651	7297773.03	8572286.647	8180856.274	3860275.292	6665805.799	6948500.08	8996454.69	9933104.46	7690743.376	4049456.24	4651083.556	7331123.973	7259718.554	7791356.355	5956260.824
Com_5319_neg	FAHFA (20:0/22:2)	--	C42 H78 O4	646.58744	11.351	645.58014	197873.4911	329412.9179	752963.9655	100579.3036	700815.8312	143529.7859	7870728.705	5693188.063	3363983.675	7701389.755	5803758.915	5303605.828	3395882.184	3311862.081	2971769.884
Com_5380_neg	Capric acid	--	C10 H20 O2	172.14626	6.496	171.1391	11057581.33	10383775.06	9153267.578	7982908.708	7481831.657	7434870.118	1427583.413	9632549.887	5855397.407	4708663.145	3611474.683	9678682.034	4993139.578	6282951.903	6913553.406
Com_5390_neg	5-Hydroxyindole-3-acetic acid	--	C10 H9 N O3	191.05792	5.47	190.05081	7016871.343	13524407.79	7908404.044	8116131.875	7395155.392	4752279.621	6708064.825	3591378.576	6903020.374	3280276.094	6090810.616	5475583.672	7077713.385	4848741.295	5521387.403
Com_5417_neg	3-Methoxytyramine	3-Methoxytyramine	C9 H13 N O2	167.09452	5.204	166.0871	10691908.06	12228665.53	8766869.592	10030610.61	8908043.883	8147287.998	8685647.421	3422695.289	2434590.339	3106939.07	2995415.399	2545908.339	6322348.723	6999065.881	8835708.086
Com_5468_neg	Calcium D-Panthotenate	--	C18 H32 Ca N2 O10	476.16849	5.965	475.16101	2941943.713	7561263.561	11211908.62	7972154.653	5254956.215	4143276.331	4630313.934	2048151.267	1464373.955	1114199.793	422029.0013	3012300.757	5251245.89	7109289.651	4841761.555
Com_5524_neg	17(S)-HpDHA	--	C22 H32 O4	360.22727	7.898	359.21985	1441201.982	1505668.347	1029229.037	1281138.251	1079932.788	1217315.61	9638044.134	9228803.505	9037990.029	6644072.6	1457240.588	7683731.168	5384383.213	4933276.072	2975890.277
Com_5530_neg	Lauric acid	Dodecanoic acid; Dodecanoate; Dodecylcarboxylate; Lauric acid	C12 H24 O2	200.17736	8.786	199.17009	3024308.574	3209540.724	3369378.416	4253417.091	4786971.192	3911097.152	5224390.157	7009453.792	5934796.293	7254790.809	6498105.265	5793287.548	5928453.361	6205102.205	5990636.016
Com_5536_neg	5-Hydroxyindole	--	C8 H7 N O	133.05279	5.239	132.04553	11335421.27	12664208.82	9721822.338	7564964.328	10484321.24	9882517.768	2927561.703	3257589.024	4288063.823	4343180.528	3637524.306	4447698.76	7426897.216	6936698.74	6685280.292
Com_5585_neg	1,2-Dihydro-1,2-	--	C10 H10 O2	162.06787	6.314	161.06067	6450882.887	7796584.195	9556308.562	9557766.585	8559497.623	9279496.108	5788092.186	5665227.881	5681104.826	6181958.191	8091866.334	5089083.836	8117623.432	7258402.418	6890873.028

Com_5665_neg	Inositol	myo-Inositol; D-myo-Inositol; 1D-myo-Inositol; L-myo-Inositol; 1L-myo-Inositol; meso-Inositol; Inositol; Dambose; Cyclohexitol; Meat sugar;	C6 H12 O6	180.06328	1.509	179.056	2156446.169	2753569.346	3592768.231	1174468.53	4378006.127	4961966.396	3764382.867	6469573.187	5621245.131	5693159.704	8509777.436	4097465.872	3348533.664	3400046.503	3022554.415
Com_5669_neg	12-epi Leukotriene B4	--	C20 H32 O4	318.21898	8.244	317.2117	2894679.733	2537467.102	5336782.137	4419695.2	3252095.031	3442943.967	2826919.531	4396283.193	5010382.466	6943099.358	2938079.85	3284615.888	3888628.929	4224930.545	3868665.423
Com_5679_neg	N4-Acetylcytidine	--	C11 H15 N3 O6	285.0974	2.027	284.09012	796593.3287	678672.9174	492987.6644	441212.0282	773771.0013	798244.4022	393308.7408	930349.3445	889343.9511	6919762.437	641395.5256	757478.6945	881212.8833	965943.4189	925401.3138
Com_5701_neg	D-Xylitol	--	C5 H12 O5	152.0683	1.29	151.06105	256319.4305	119267.93	749340.9638	629508.8218	1377718.964	579648.0179	978626.6949	379574.5178	1285836.277	6869422.896	744215.872	135900.2014	4881015.02	5383464.938	5224617.093
Com_5706_neg	N-Acetyl-Asp-Glu	--	C11 H16 N2 O8	304.0906	1.375	303.08337	7144532.829	7238213.903	8663003.907	9170254.146	10384751.91	8363419.803	2523488.072	4195126.737	4004016.604	5749393.432	3382799.863	3852947.93	4904026.944	6127102.292	5262001.814
Com_5719_neg	Citrulline	L-Citrulline; 2-Amino-5-ureidovaleric acid; Citrulline	C6 H13 N3 O3	175.09542	1.503	174.0882	3931457	10176008	10375777.71	8532728.745	9625526.154	9861155.226	1229833.656	7982532.463	2541530.015	5887308.124	1611348.308	6869868.052	7260335.98	8716121.507	8294007.931
Com_5749_neg	5-Hydroxyindole-2-carboxylic acid	--	C9 H7 N O3	177.04227	4.799	176.03499	4882964.293	12134739.7	4452999.399	2798079.792	3673903.903	4334535.263	1434368.75	820442.9548	1052467.558	810898.2508	847230.3638	911186.3948	2611183.879	2718582.507	2407889.788
Com_5835_neg	3-Hydroxydecanoic acid	--	C10 H20 O3	188.14096	6.988	187.13364	2670661.749	2205128.944	2312968.315	1966809.247	2591803.638	3030530.559	2757937.129	6380044.921	4127932.947	6592000.556	6209566.673	5066077.099	3716389.01	3816525.476	4016721.433
Com_5893_neg	5-Methoxyindole-3-Carbaldehyde	--	C10 H9 N O2	175.06309	5.228	174.05574	10097447.37	3849429.186	3411985.751	2816974.984	2558788.834	1882645.121	1268942.063	909173.741	1095508.48	1048831.732	1845284.048	1386512.212	2461137.298	2423736.461	2248461.026
Com_5956_neg	acetoacetate	Acetoacetate; 3-Oxobutanoic acid; beta-Ketobutyric acid; Acetoacetic acid	C4 H6 O3	102.03152	1.335	101.02426	991596.1579	728800.1162	896250.6636	753977.4419	732766.3006	934296.5139	1814849.263	8088714.548	3024396.247	2966343.21	4422486.223	3092649.671	1733402.573	2092618.782	1871165.167
Com_6029_neg	Tridecylic acid	--	C13 H26 O2	214.19304	9.2	213.18575	3223402.307	4271731.667	3897138.073	3968909.739	3771322.86	3825594.005	2948483.745	7925134.929	7644299.561	6207080.819	4601268.001	6513898.467	5384418.69	4674402.855	5259769.907
Com_6056_neg	Glycochenodeoxycholic acid sodium salt	--	C26 H43 N O5 Na	472.30244	6.926	471.29584	7843408.434	6292427.623	5700109.04	6459438.683	6438437.723	6269641.217	6145945.518	2581635.763	5612152.367	5598611.645	7583203.262	1560700.625	5307509.926	5255598.426	5449133.788
Com_6147_neg	Lignoceric acid	Tetracosanoic acid; Lignoceric acid	C24 H48 O2	414.37083	10.16	413.36346	2863354.498	4886497.387	9133036.359	6633784.675	7130639.973	4347601.033	374040.2826	339782.8265	306813.8642	219452.9749	234330.8544	264425.8296	3607783.25	2563097.128	2996383.1
Com_6176_neg	4-Butylresorcinol	--	C10 H14 O2	166.0991	5.864	165.09201	9142902.476	8515603.437	8650981.518	7400066.008	8685975.517	7256539.478	1885946.428	3044619.805	3334828.466	3499312.947	3317991.301	3162049.27	5957635.8	5719825.264	7955127.476
Com_6227_neg	2-Hydroxy-2-methylbutanoic acid	--	C5 H10 O3	118.06289	5.22	117.05548	3688391.893	3691586.305	3260760.255	3412708.038	6226467.472	6284228.063	7045084.572	2537410.557	5217751.915	5891447.98	2691228.795	4188123.603	4203177.627	3785582.743	3897125.765
Com_6287_neg	D-Mannose 6-phosphate	D-Mannose 6-phosphate	C6 H13 O9 P	260.0319	6.849	259.02463	932571.2729	1459237.989	1205745.991	1334698.827	1222291.194	1485846.047	2195267.535	1747920.707	1746670.279	1078505.407	7089594.114	1412955.721	1359488.847	881639.4701	1302649.707
Com_6321_neg	Thymidine 3',5'-cyclic monophosphate	--	C10 H13 N2 O7 P	258.03719	1.404	257.03012	4355424.656	4044977.422	8694252.06	5518214.106	6249298.172	4193843.458	303211.5823	549605.9497	495869.6212	406343.4032	428427.1107	459977.6131	1848467.229	3052484.402	1991272.095
Com_6350_neg	Glucuronic acid-3,6-lactone	--	C6 H8 O6	176.03314	4.845	175.02586	1205038.892	1849628.98	4898398.722	5476148.112	8260977.462	9651695.852	751291.6757	2392233.09	1814425.143	4895823.827	767627.3206	1784237.541	3800288.106	3314878.003	3400794.018
Com_6359_neg	Oxaceprol	--	C7 H11 N O4	173.06859	1.426	172.06129	5836493.364	5432841.564	7175337.503	6869689.567	8563906.326	7021861.751	2265501.878	2380819.687	2105478.577	2489207.718	6944929.856	2396738.666	4430009.46	4432633.382	4399104.76
Com_6424_neg	2-Phenylglycine	--	C8 H9 N O2	151.06323	5.266	150.05579	7779964.251	8503231.198	7452692.937	7445573.006	7542571.497	6971474.497	2931102.73	2778399.541	2744603.182	2962957.682	2261351.924	2028290.511	5371427.637	5837376.24	5247763.954
Com_6502_neg	11(Z)-Eicosenoic acid	--	C20 H38 O2	356.29216	9.895	355.28497	7531967.809	6722009.346	7077702.637	7250904.822	5730264.045	4928727.86	6963930.184	6113330.417	3795285.145	2625755.267	6258300.707	6529282.457	6558817.102	7018409.349	[illegible]
Com_6569_neg	Sorbitan monopalmitate	--	C22 H42 O6	402.29769	8.109	401.29031	8418327.201	7602260.886	6347204.952	6458261.685	4826220.863	4857126.077	1864746.816	1477262.561	2217249.4	2691699.545	1796173.058	1718258.884	4351412.646	5277718.919	4516724.957
Com_6719_neg	LPE 14:0	--	C19 H40 N O7 P	387.29802	8.559	386.29074	8064123.44	5998173.535	3554055.868	2975878.681	4327531.198	3077543.477	6285150.984	3049826.27	3278460.734	2449751.814	2736118.709	2726561.404	3171584.903	3890537.654	3626866.795
Com_6846_neg	SM (d14:3/20:0)	--	C39 H75 N2 O6 P	744.55415	11.03	743.54688	230614.7988	258377.6285	233826.3805	215105.3885	332515.1302	298434.4972	7150347.253	1556023.452	259559.057	173533.9448	731038.9663	727123.2826	1082085.806	739027.1972	794944.0689
Com_6899_neg	Cer-NP (t18:1/14:0)	--	C32 H63 N O4	571.48107	11.811	570.47382	145959.3951	156855.8777	166334.6687	137423.1896	181416.8389	175637.2114	2253901.939	6249900.911	125483.3643	177658.09	611558.4976	1781717.169	813867.9814	906583.0979	743022.5189
Com_6969_neg	4-Methylvaleric Acid	--	C6 H12 O2	116.08387	5.524	115.07659	4058432.239	4129920.679	3243983.21	4413438.011	4073184.881	5011298.473	3764068.387	2581096.155	4648921.435	4113304.117	5930501.156	3399327.335	3693461.853	3504248.884	3096752.555
Com_7019_neg	Ornithine	Ornithine; 2,5-Diaminovaleric acid; 2,5-Diaminopentanoic acid; 2,5-Diaminopentanoate	C5 H12 N2 O2	132.08963	1.3	131.08218	2164439.372	5881641.093	6764828.819	5188921.285	5784684.301	6221059.47	1056203.485	6066221.67	2150235.014	3986502.009	1116508.398	4592995.099	5829577.022	5853303.382	5559838.449
Com_7048_neg	LPE 20:4	--	C25 H44 N O7 P	501.28583	9.084	500.27844	5176255.313	8495243.97	4413574.413	2399729.97	6484683.021	958793.4637	270333.8536	253403.652	357014.9926	393296.1927	223419.3487	246521.8754	3099856.598	2175838.45	2525600.24
Com_7109_neg	L-Serine	L-Serine; L-2-Amino-3-hydroxypropionic acid; L-3-Hydroxy-alanine; Serine	C3 H7 N O3	105.04236	1.296	104.03508	164544.6403	160870.1041	489092.4647	603833.7869	532464.8024	311880.3491	477100.523	2852099.266	1461276.584	4671039.402	579264.1773	1770798.88	1292095.49	1413611.213	1104685.857
Com_7163_neg	2,5-di[4-(trifluoromethyl)benzylidene]cyclopentan-1-one	--	C21 H14 F6 O	396.08726	12.014	395.07999	342851.3691	875797.5811	464769.7736	507459.9816	452450.5933	702265.9859	294348.2648	228042.7594	230187.9441	171642.4708	5628809.126	646908.6016	908662.7991	770559.0453	950688.4856
Com_7165_neg	11-deoxy Corticosterone	--	C21 H30 O3	376.2224	7.057	375.21512	752827.4875	1602884.849	472565.2671	1011980.001	882864.58	1137527.629	6601450.247	1555550.46	3727620.647	3689899.781	1360425.088	920111.6169	1979650.082	1532239.426	1553358.736
Com_7267_neg	3-(1,2,3,3,3-hexafluoropropyl)adamantane-1-carboxylic acid	--	C14 H16 F6 O2	330.11061	5.199	329.10333	2524727.803	2382360.454	2732577.438	1859331.616	7020485.942	3243231.852	1091624.61	754276.155	684788.1263	762339.2114	620323.7289	773593.6009	2329029.954	1729979.586	1353528.951
Com_7313_neg	Glycylproline	--	C7 H12 N2 O3	172.08454	1.363	171.07748	3337520.033	5585737.841	5646384.737	5305241.674	4601233.818	6308399.25	1910439.152	2027071.651	1291700.784	1551713.008	5446011.644	2176247.412	3062755.204	4791710.726	4870214.18
Com_7420_neg	FAHFA (4:0/16:0)	--	C20 H38 O4	342.27674	11.817	341.26944	217068.3566	206624.9523	321844.8042	240189.1173	316236.2962	173220.3558	1002752.873	1765831.439	1415877.539	503645.9577	5316471.281	3827137.618	1596723.8	1570631.905	1286012.807
Com_7455_neg	trans-2-Butene-1,4-dicarboxylic Acid	--	C6 H8 O4	144.04204	1.312	143.03474	941523.1772	1013341.473	1417296.605	831129.9075	1078918.495	739217.5537	1192229.827	5478440.672	3600163.639	2674407.138	3368077.288	1522168.578	2150770.32	1685239.407	1336640.594
Com_7513_neg	(+/-)7(8)-DiHDPA	--	C22 H34 O4	362.24531	7.071	361.23804	5166942.299	5145676.62	5702208.728	5163507.976	6665082.381	6965636.372	1468044.208	960941.6642	1629707.687	1656576.557	1091244.607	1091539.389	3128848.573	3308490.674	3466205.488
Com_7854_neg	2,3-Dinor-8-epi-prostaglandin F2α	--	C18 H30 O5	652.41847	7.08	651.41119	6124992.639	5235425.427	3611834.554	4313880.551	4741342.117	4097904.82	2175198.097	2325267.076	1581800.734	3348387.641	1759481.223	1375607.923	3057611.222	2812804.208	3773000.09
Com_7867_neg	3-Hydroxyanthranilic Acid	--	C7 H7 N O3	153.04252	5.002	152.03525	4998161.959	5200674.647	4326937.739	4534895.587	4375348.709	4323863.752	3529093.925	4496765.406	4254293.021	3902480.648	3328516.535	3715586.506	3897247.308	4201345.516	3857914.566
Com_7992_neg	3-Phenoxybenzoic acid	--	C13 H10 O3	214.06036	1.982	213.05309	613530.4418	507588.0482	339468.9571	332362.0288	607393.2316	591044.9713	405733.7417	4826309.903	495422.6839	443344.5612	439439.9735	4744742.433	759611.7411	646282.646	726392.9022
Com_8235_neg	Prolylglycine	--	C7 H12 N2 O3	190.0951	1.358	189.08798	882446.0532	609152.1165	1019330.119	1008491.526	760617.2184	849760.2021	1171249.26	1287369.966	825795.8514	771570.7131	4374606.877	1255415.298	932473.5134	1522068.891	1024594.671
Com_8268_neg	2,3,4-Trihydroxybenzoic acid	--	C7 H6 O5	170.02108	5.815	169.01401	4345212.028	3743281.297	3710379.001	3546780.992	3097821.264	3613606.675	3991919.064	2324314.33	3317838.378	3548760.924	4193959.555	2621931.311	3499552.592	3298384.043	3699023.917
Com_8281_neg	Estradiol	Estradiol-17beta; Estradiol; beta-Estradiol	C18 H24 O2	272.17724	8.91	271.16983	560630.5235	707060.5581	5364177.803	4600532.283	672568.9592	726713.8414	511739.6983	464500.9761	518123.3487	492699.1401	468481.3232	505563.4795	1076665.522	1025532.312	810087.4041
Com_8488_neg	2-Methoxyestradiol (2-	--	C19 H26 O3	302.1881	7.914	347.18628	1535387.092	1362964.911	2251543.247	1741443.004	2125460.478	2093112.563	2845095.062	2852912.414	2997912.792	3355101.797	1767297.088	2248817.299	1897435.094	2438163.853	2005787.925
Com_8507_neg	DL-o-Tyrosine	--	C9 H11 N O3	181.07377	6.273	180.0666	1487261.036	1347405.584	1589592.009	1485666.903	1375322.788	1390274.744	3975844.473	2578267.389	3540274.215	2158182.834	4089183.781	3404551.981	2273056.832	1852262.69	2266874.192
Com_8557_neg	2-(Formylamino)Benzoic Acid	--	C8 H7 N O3	165.04239	5.128	164.03487	5117467.419	3503867.715	3288075.495	3401699.013	4118367.37	3351809.379	3318597.028	2088472.851	3046316.85	2762886.166	4041032.323	2506351.162	3226121.977	3276776.558	2883171.478
Com_8722_neg	N-Acetylglycine	--	C4 H7 N O3	117.0425	1.502	116.03509	3851074.451	3548124.938	2772165.49	2260548.31	2034697.471	5400957.702	1606364.092	2463472.654	1840245.268	2618210.83	2001150.779	2061360.363	2651863.096	3030284.559	2693405.519
Com_8790_neg	(3-Methoxy-4-hydroxyphenyl)ethylene glycol sulfate	--	C9 H12 O7 S	264.02976	5.087	263.02222	3079644.291	3744911.897	3917772.411	4189813.374	3454941.387	3540734.957	1435501.315	2371232.412	1843762.332	2090154.074	1020629.577	2588260.86	2440233.21	2813990.915	2450636.487
Com_8879_neg	N-Methylthreonine	--	C5 H11 N O3	133.0736	1.339	132.06625	240539.3558	324833.3686	292194.9623	235361.0397	373413.0713	437656.011	916366.3431	3890018.345	1379739.859	1677949.882	2163134.487	3096205.207	295348.6666	330682.2153	197288.5048
Com_8933_neg	Soyasaponin I	Soyasaponin I	C48 H78 O18	942.51895	7.781	941.51233	362113.2781	525189.4527	490851.2119	507451.7767	588948.9169	654678.3955	1454950.255	3114201.635	382665.0222	1632730.444	1252273.32	3852925.258	1694444.039	1408479.826	1414572.322
Com_9092_neg	(+/-)9,10-dihydroxy-12Z-octadecenoic acid	--	C18 H34 O4	628.49147	9.761	627.48438	232059.642	253546.2194	239896.8283	201042.8835	238697.2886	242372.0836	626160.1755	185803.7707	1456388.752	2914653.492	744780.2239	185069.0789	379866.3441	420145.9574	299784.0242
Com_9189_neg	4,7-dimethylpyrazolo[5,1-c][1,2,4]triazine-3-carbonitrile	--	C8 H7 N5	173.06851	1.838	172.06123	1447169.697	1724932.848	1914025.021	2254098.818	2167835.046	2056926.538	1368229.813	939321.9114	3719510.904	2146048.912	2772392.836	1144911.455	2025550.302	1582237.59	1335638.998
Com_9192_neg	Sorbic acid	--	C6 H8 O2	112.05233	4.864	111.04488	3539537.182	3923755.847	4307307.102	3445316.111	3882175.681	4541514.101	1042472.677	878850.9724	871735.4798	999948.1785	1011593.026	958973.1547	3027451.313	3203394.188	2994471.034
Com_9193_neg	LPE 12:0	--	C17 H36 N O7 P	397.22243	7.894	396.21515	4445301.211	1347620.81	1804404.909	974949.854	2169458.634	1915438.526	1212759.997	645417.6793	514353.9677	627441.1288	730267.3368	736734.7169	1464140.719	1630010.075	1943070.718
Com_9198_neg	(S)-beta-Aminoisobutyric Acid	--	C4 H9 N O2	103.0632	1.325	102.05579	711710.9999	955310.3924	1027647.842	1047652.106	1427091.566	1567397.86	518224.2558	2524450.972	921323.5237	2838454.14	457416.5984	1832282.292	1428734.953	1411525.438	1224742.153
Com_9219_neg	17α-Hydroxyprogesterone	17alpha-Hydroxyprogesterone; 17alpha-Hydroxy-4-pregnene-3,20-dione; Pregn-4-ene-3,20-dione-17-ol;	C21 H30 O3	330.21921	11.552	329.21179	117837.4311	129396.5803	105431.2645	106224.2644	121648.2068	125698.0557	2184288.265	2496113.885	2130378.952	2822426.63	2112751.966	2913017.338	1289020.991	1531557.958	1754088.846
Com_9304_neg	5(S),15(S)-DiHETE	--	C20 H32 O4	318.21882	7.145	317.21155	456787.7776	585346.3943	500158.2034	704120.0971	451928.6584	422618.8284	3515723.052	1048859.672	3612042.08	1968876.201	860991.6961	2151417.086	880918.3033	1015584.934	883936.0212
Com_9381_neg	Paracetamol	Acetaminophen; Paracetamol	C8 H9 N O2	151.06299	4.731	150.05571	4116022.628	4857778.517	3969349.883	2984987.341	4037633.657	3150846.181	1549736.482	757733.2029	865129.8944	880361.1167	657245.9358	762941.2918	2280550.417	2650390.581	2426955.707
Com_9487_neg	N-Propionylglycine	--	C5 H9 N O3	131.05795	2.041	130.05067	3297761.601	2215464.281	2627947.135	3008448.284	3291895.309	2441956.366	2632740.524	2317226.979	2049906.593	2652602.502	3208871.323	2239449.738	2531618.525	2098747.439	2237435.116
Com_9489_neg	7Z, 10Z, 13Z, 16Z, 19Z-docosapentaenoic acid	--	C22 H34 O2	330.25737	9.661	329.25003	1073849.228	940629.5843	607503.6906	408749.4664	865609.0079	1002220.099	772961.2408	708185.2801	886377.2442	2651162.844	1335380.445	525627.1691	721271.3615	1208279.757	854781.9538
Com_9561_neg	5-Methyluridine	--	C10 H14 N2 O6	258.08505	1.486	257.07776	1343403.044	1622338.621	2222334.408	1429416.709	2485408.187	1226815.689	505075.7682	1658392.942	1288845.721	1316912.927	973668.8682	3331404.422	1231787.156	1314213.986	1116857.206
Com_9566_neg	(±)10(11)-EpDPA	--	C22 H32 O3	344.23772	9.282	343.23068	4078209.231	3200780.905	2217201.343	1111422.361	2498704.217	2684248.659	305690.122	272279.1866	277464.9565	498733.4092	277515.3122	394433.3857	1470409.228	837944.4518	945116.416
Com_9852_neg	Methyl 3-indolylacetate	--	C11 H11 N O2	189.07865	5.582	188.07156	2335201.104	1694252.765	1400420.614	3269541.548	1489353.332	993883.5864	653275.7388	601238.3927	735214.805	731501.881	525134.6386	888527.0355	1076924.707	930449.6506	1042253.693
Com_9898_neg	N-acetyl-L-ornithine	--	C7 H14 N2 O3	174.10003	1.349	173.0927	3001237.603	1498760.873	2781298.388	2108931.93	1745312.38	3680858.49	1288217.072	2724411.709	2182866.444	1796526.46	2966542.589	1623513.462	2000421.923	2285416.055	1959501.804
Com_9920_neg	D-Phenylalanine	D-Phenylalanine; D-alpha-Amino-beta-phenylpropionic acid	C9 H11 N O2	165.07874	1.36	164.07158	2005546.41	3416822.771	3654159.754	2705018.396	2940771.777	2922548.133	1012677.82	2680125.704	1609862.466	1751588.57	1364675.383	1387342.242	2510734.935	3067328.652	2637239.045
Com_9957_neg	4-Guanidinobutanoic acid	--	C5 H11 N3 O2	145.0849	1.377	144.07764	312109.4122	298542.3957	462849.4973	493872.1955	520843.1868	520544.4371	454817.106	828292.1986	1013497.225	2389980.075	602396.5653	612619.7954	669240.6858	989192.2971	670310.1093
Com_9972_neg	Heneicosanoic acid	--	C21 H42 O2	326.31821	11.727	325.311	2581941.02	2456313.809	2548847.922	1359832.598	3726163.998	849587.4116	268285.5595	162905.2254	79431.4616	51858.20078	79805.525	178048.1937	1571086.807	1717462.726	1420412.128
Com_10105_neg	20-Carboxy-Leukotriene B4	--	C20 H30 O6	366.20422	5.604	365.19592	2494189.451	1726364.116	1603593.692	1566598.524	3343163.069	3404631.051	1945869.654	1408959.697	1910200.593	2310448.067	1932519.435	1286761.738	1488540.653	2243417.269	1299419.709
Com_10189_neg	L-Carnitine	L-Carnitine; L-gamma-Trimethyl-beta-hydroxybutyrobetaine; Vitamin BT; 3-Carboxy-2-hydroxy-N,N,N-trimethyl-1-propanaminium hydroxide, inner salt; Levocarnitine; (R)-Carnitine	C7 H15 N O3	161.10517	1.387	160.09795	805024.0455	759602.2018	761245.3187	637392.7863	1003020.314	898866.2458	1356073.266	814021.8281	984127.6973	513877.2143	2765821.884	704707.8897	709292.9218	584163.2231	759107.5678
Com_10230_neg	5-Phenylvaleric Acid	--	C11 H14 O2	178.09918	6.034	177.0919	2840966.384	2989324.537	3390775.78	2538551.782	2545527.936	3178826.001	1260805.345	1285341.742	1203094.945	1755869.456	1430193.853	1458979.508	2119763.357	2201949.346	1615402.248
Com_10367_neg	L-Adrenaline	L-Adrenaline; (R)-(-)-Adrenaline; (R)-(-)-Epinephrine; (R)-(-)-Epirenamine; (R)-(-)-Adnephrine; 4-[(1R)-1-Hydroxy-2-(methylamino)ethyl]-1,2-benzenediol	C9 H13 N O3	183.08937	6.113	228.08751	3396822.775	3744228.603	2535024.268	2647580.16	1209977.057	2805927.313	1412761.501	1522440.715	1196535.391	1841891.547	2011582.177	1312608.669	2390709.208	2283341.515	1804594.967
Com_10458_neg	Ureidoisobutyric Acid	--	C5 H10 N2 O3	146.06894	1.295	145.06166	620980.3355	307568.1254	1291465.115	842301.0154	932733.261	2095304.632	385952.1842	1584836.558	2206292.807	953311.6523	2602818.842	1514499.335	1653154.779	1696843.152	1424931.342
Com_10685_neg	LPG 16:0	--	C22 H45 O9 P	484.28245	7.844	483.27435	748832.4262	1386479.19	2457560.309	1468650.7	156025.217	158213.3663	1714255.86	1204085.012	820357.2165	157885.0297	2475631.61	1217855.765	859441.8749	1716682.9	1261613.713
Com_11107_neg	(1E)-1,7-bis(4-hydroxyphenyl)hept-1-en-3-	--	C19 H20 O3	296.14107	7.146	295.13379	268353.2822	236978.4148	247367.7162	251246.6547	239724.6349	225922.5179	2616556.754	224274.4309	206784.4606	339802.0741	301797.5477	185022.7784	292097.7001	222538.4888	225309.5229
Com_11175_neg	8-iso Prostaglandin F2α Ethanolamide	--	C22 H39 N O5	397.28212	10.596	396.27484	971629.5452	986503.402	817521.977	308771.6095	1306530.598	730520.5692	2580180.629	1024596.864	208303.1271	139241.7813	526235.6161	516718.167	565775.8647	548992.0594	424466.7718
Com_11209_neg	S-(5-Adenosy)-L-	--	C14 H20 N6 O5 S	384.12251	6.872	383.11462	1590527.037	1681113.118	1327974.018	1421360.23	2520472.129	3029516.724	947090.3605	1536712.351	1054462.71	1303061.916	960825.0808	1029644.061	1587358.902	1835756.783	1595753.529
Com_11274_neg	2-(3,4-dimethoxyphenyl)ethanamine	--	C10 H15 N O2	181.11029	5.494	180.10309	2122548.969	2408327.572	2662034.241	1811680.206	1627957.97	2091599.064	824215.9834	878079.5502	952583.4424	917578.4954	637908.0817	896582.0593	1125914.235	1516640.04	1209542.464
Com_11682_neg	alpha-D-Glucopyranosyl 2-O-(2-methylbutanoyl)-alpha-D-glucopyranoside	--	C17 H30 O12	426.16856	7.166	425.16129	2483808.404	2028146.18	1530109.975	1498442.409	1604247.702	1128893.609	351266.1508	226243.0743	195866.3759	217311.8889	339582.0984	267784.6938	1126045.831	1060553.726	968594.4151
Com_12006_neg	5-Hydroxymethyl-2-furancarboxylic acid	--	C6 H6 O4	142.02643	5.496	141.01923	1311227.694	1280605.603	1198194.997	1070220.407	1018654.357	1002515.624	1697522.431	903539.4729	1389280.133	1461634.087	1081393.304	1138119.183	1033345.732	1158404.028	1069321.271
Com_13152_neg	S-Adenosyl-L-methionine	S-Adenosyl-L-methionine; S-Adenosylmethionine; AdoMet; SAM	C15 H22 N6 O5 S	398.13893	6.254	397.13165	1700853.918	643185.0533	989971.0437	935957.539	489113.339	441839.386	207775.7246	336956.9446	425076.5249	408058.7555	347931.423	303378.0532	251454.6405	330876.4998	354933.478
Com_13490_neg	Docosanoic Acid	Docosanoic acid; Docosanoate; Behenic acid	C22 H44 O2	340.33369	11.509	339.32642	85435.99196	93816.7532	76441.11535	83239.77612	88198.92899	100032.0323	78603.77395	74691.57169	60757.37291	48494.18568	1216484.728	80588.54963	163704.332	132556.3836	137053.4412
Com_13506_neg	2-Hydroxy-6-Aminopurine	--	C5 H5 N5 O	151.04904	5.499	150.04187	1550742.127	750313.8189	923895.4192	1119483.481	1333288.976	924490.4215	384892.792	390358.3319	384290.0549	441001.5744	268287.2111	404922.2019	500137.1091	472184.3775	551852.6428
Com_14861_neg	Noradrenaline	L-Noradrenaline; Noradrenaline; Norepinephrine; Arterenol; 4-[(1R)-2-Amino-1-hydroxyethyl]-1,2-	C8 H11 N O3	169.07423	6.444	168.0665	322530.7203	575725.6674	394248.679	824619.0879	302560.3982	259491.9056	269438.6715	215696.8181	264275.2898	251840.9378	306140.0639	204894.9189	253216.4762	273675.0167	245888.2129
Com_15194_neg	Undecanoic acid	Undecanoic acid; Hendecanoic acid	C11 H22 O2	186.16185	11.668	185.15457	88628.70583	100264.059	85048.1696	82500.17996	82414.2356	110622.4268	126107.5825	606499.2209	179428.0705	57390.00959	92196.80552	668065.2015	132009.0613	201639.4459	146763.9038
Com_15443_neg	Palmitelaidic acid	--	C16 H30 O2	254.22402	9.241	253.2169	296265.3916	341708.7752	209246.3697	222680.5643	324018.0833	335805.8237	232124.071	305370.3917	242056.9786	443143.2445	270503.7832	282550.2099	283752.7651	266309.8745	288169.9362
Com_15789_neg	Terephthalic Acid	Terephthalate; Terephthalic acid; 1,4-Benzenedicarboxylic acid	C8 H6 O4	166.02651	6.937	377.05099	212135.1179	274473.9624	373820.4959	299919.5872	199832.5568	212711.1838	195875.2707	225418.5881	155185.7563	237510.0802	252973.2768	215697.5552	220186.2233	389260.8587	352617.0584
Com_2_neg	Glycocholic acid	Glycocholate; Glycocholic acid; 3alpha,7alpha,12alpha-Trihydroxy-5beta-cholan-24-oylglycine	C26 H43 N O6	465.30846	7.234	464.30081	145851483.5	129508510.6	287047360.8	266775004.3	205424286	154465731.6	12414064207	8968421448	3426768760	1382146179	13860952526	8847532838	4343294550	4214829846	4290709734
Com_2864_neg	Glycoursodeoxycholic acid	--	C26 H43 N O5	449.31378	7.14	448.30688	2538886.168	1380054.756	2936107.505	3693231.641	1315408.007	1090408.926	25547677.61	24567765.35	8943939.917	5354561.469	20003244.75	24931945.11	8864224.14	9485199.925	7864948.156
Com_53_neg	Ursolic acid	Ursolic acid	C30 H48 O3	456.35975	9.556	455.35233	38221718.12	56472320.92	8314532.416	7110941.074	58166662.72	72788510.01	1966309124	1400478689	1200063406	919421454.5	925619154.1	1068205229	925080741.1	845867410.5	881788286
Com_59_neg	18-β-Glycyrrhetinic acid	--	C30 H46 O4	470.33903	8.215	469.33167	8797463.576	6557869.029	3511923.809	3977134.902	12727368.64	13628343.05	757993834.1	1479299349	1028487345	1284941948	1184547205	755127988.1	730874567.8	727966158.2	683780671.9
Com_1288_neg	Pentadecanoic acid	Pentadecanoic acid; Pentadecylic acid; n-Pentadecanoic acid	C15 H30 O2	242.22427	10.003	241.217	64266578.32	69347827.92	58851539.39	48499202.34	64962093.54	71412008.17	38751879.25	66233404.28	54518433.99	42425528.17	41774905.09	59006830.32	54673604.2	53430001.76	51540913.33
Com_82_neg	Tetradecanedioic acid	--	C14 H26 O4	258.18276	7.261	257.17532	1360898780	1320756263	1383375227	1261440517	1333696485	1407578919	877208089	258631041.1	676027161.2	967282979.5	383874741	303976654.9	947396817.2	922699372.6	922235875.6
Com_289_neg	2-Hydroxyphenylalanine	--	C9 H11 N O3	181.07363	5.001	180.06635	312859755.4	360638308.5	375401074	363006772.5	409635420.2	393482502.1	3192247.086	7305509.37	3320225.032	3995151.507	8595097.596	11536191.62	201115611.3	203399608.3	177334030.2
Com_194_neg	N-acetyl-D-glucosamine	--	C8 H15 N O6	221.08975	1.371	220.08234	400701193.2	332164159.3	199747772.4	140019727.1	218034323	471049112.2	50847943.3	121814922.4	112806637.9	378425966.5	59047489.45	137152478.6	199302758.6	256318755.9	234012762
Com_764_neg	12-oxo Phytodienoic Acid	--	C18 H28 O3	292.20358	8.094	291.1962	34704581.43	43042411.32	39378061.77	65543055.66	6526121.757	4429640.178	3009521.351	114186340.4	14546830.36	25730307.64	7619972.078	103156084.7	38727145.76	38220278.77	40941590.42
Com_475_neg	7-Ketodeoxycholic acid	--	C24 H38 O5	406.27161	6.885	405.26437	29694849.8	27906190.74	39519211.61	49101505.21	31261357.28	45946737.43	77496043.26	66197545.41	64733166.75	152450998.1	127599601.5	65786627.18	84581994.85	73599021.46	65418092.42
Com_144_neg	13(S)-HOTrE	13(S)-HOT; (9Z,11E,15Z)-(13S)-Hydroxyoctadeca-9,11,15-trienoate; (9Z,11E,15Z)-(13S)-13-Hydroxyoctadeca-9,11,15-trienoic acid; (9Z,11E,13S,15Z)-13-Hydroxyoctadeca-9,11,15-trienoic acid; 13(S)-HOTrE	C18 H30 O3	294.21919	7.891	293.21191	47744788.39	44423526.65	30445586.58	42825433.57	61457156.12	41489573.91	347235763.1	338852731.2	520561820.2	505348926.9	346927623.7	264869123.4	231996800.6	242094528.8	233082735.4
Com_667_neg	6-Ketoprostaglandin F1α	--	C20 H34 O6	370.23506	6.21	369.22778	82396010.23	67801219.36	66856052.36	64254049.22	61407653.66	59949371.38	88679278.13	50552157.39	70627083.69	106642618.2	79753729.28	50898969.07	61497159.89	60099650.5	60203625.53
Com_5066_neg	13,14-Dihydro prostaglandin	--	C20 H36 O5	356.25604	8.7	355.24881	6922813.15	7659244.73	5280159.481	6774030.455	4977681.292	4761139.074	4629927.788	5344450.828	3251960.793	5144266.126	10213534.08	5721602.531	5698168.222	5718173.444	5541440.953
Com_95_neg	Arachidic acid	Icosanoic acid; Eicosanoic acid; Arachidic acid	C20 H40 O2	312.30239	11.348	311.29507	128101181.8	193808478	220513706	113713282.7	339351854.7	139451536.8	1014846015	767111324.1	603774126	846825042.5	844290639.8	909243224.6	595811370.9	642543007.7	606880890.7
Com_246_neg	Arachidonic acid	Arachidonate; Arachidonic acid; (5Z,8Z,11Z,14Z)-Icosatetraenoic acid; cis-5,8,11,14-Eicosatetraenoic	C20 H32 O2	304.23985	9.986	303.23248	421725299.5	460362563	472568828.5	346690689.3	387063235.1	440852923.3	96238393.83	89276026.06	128532180.3	155964208.1	81086342.93	89189731.78	193977314.4	193584828	191061079.3
Com_2838_neg	Uracil	Uracil	C4 H4 N2 O2	112.02717	1.477	111.01978	11118493.66	10154613	10570108.82	9837948.004	16029585.63	13436786.51	6427249.625	25014153.67	15515704.49	15500259.66	13815927.11	18141112.51	12021173.74	9732433.902	13149982
Com_1089_neg	Glycolithocholic acid	Glycolithocholate; Glycolithocholic acid	C26 H43 N O4	433.31842	8.492	432.31116	16891681.2	20833719.71	20534094.12	24010881.21	15294735.2	30812590.99	88229654.05	62108648.21	52393074.96	11574933.95	61667240.22	58592352.43	44322194.26	38682445.02	33700096.9
Com_1551_neg	Docosahexaenoic Acid	(4Z,7Z,10Z,13Z,16Z,19Z)-Docosahexaenoic acid; 4,7,10,13,16,19-Docosahexaenoic acid; Docosahexaenoic acid; Docosahexaenoate	C22 H32 O2	328.23977	9.912	327.23254	50533783.99	57736176.16	62552090.85	47344267.39	51649954.19	59013492.53	13607322.24	11843550.96	16697797.32	20516410.69	8550383.319	10624012.9	25382836.48	25404893.58	23799665.01
Com_969_neg	2,3-Dinor-11β-prostaglandin	--	C18 H30 O5	362.19358	6.054	361.18649	95946563.71	96318825.62	82239852.61	93403024.3	83684171.66	76380389.61	43426747.58	35228895.09	28586831.66	40433346.31	27215634.83	41153661.11	46342293.1	66844723.96	60535845.08
Com_810_neg	MAG (18:4)	--	C21 H34 O4	350.24524	8.087	349.23816	1256342.418	1566450.683	1293413.748	1706985.567	1938050.566	2130146.223	70499840.74	46569134.31	31560113.14	39225814.18	104316221	31770922.1	24645394.69	24550608.6	24205111.47
Com_877_neg	methyl 2-[(2-acetyl-3-oxo-1-butenyl)amino]acetate	--	C9 H13 N O4	199.08422	5.006	198.0769	123757817.2	107264448.6	103004605.3	83858298.69	86892658.74	84751768.01	14142526.38	13760627.97	12517161.13	12410050.01	10194996.44	15624159.56	54054432.38	50866772.84	66930125.05
Com_206_neg	8Z,11Z,14Z-Eicosatrienoic acid	--	C20 H34 O2	306.25541	10.247	305.24805	106018847.1	108813098.3	85846568.94	70099043.62	84787771.05	70689077.46	284412669.2	253436569.3	314409086.3	363200085.5	86703734.77	232158899.6	173024159.6	157003415.7	169145639.1
Com_227_neg	Bisphenol A	Bisphenol A; 2,2-Bis(4-Hydroxyphenyl)propane	C15 H16 O2	228.1148	5.69	227.10739	427115576.3	398021898.4	413570589.2	442830459.3	400255607.9	417123330.7	184245277.1	153320714.4	171918561.5	148634153.7	123025775.8	177554577.1	309717085.5	324314558.2	321336208.9
Com_356_neg	Inosine	Inosine	C10 H12 N4 O5	268.08043	3.382	267.07315	36082622.02	57532750.6	40909954.24	17968211.44	330040264.4	228365695.2	14182293.92	43789565.65	53421915.99	4198033.431	22059243.35	35706775.07	54398168.41	55581114.91	52106990.93
Com_1381_neg	Reserpine	Reserpine	C33 H40 N2 O9	608.2633	9.708	607.25635	1481013.023	1680884.218	1668340.024	1376235.315	1321932.585	1458557.842	38364289.52	20501062.87	54253832.32	1943151.738	58142298.26	17857113.54	23191291.32	19307504.84	17153506.42

Com_1716_neg	12,13-EODE	--	C18 H32 O3	296.23451	7.937	295.22708	5358291.651	3986764.792	3417472.041	4312013.645	5358747.024	3895976.799	6948920.802	15404467.17	17426862.73	37023287.08	9357282.469	10809491.62	14352624.45	11622037.9	13916917.49
Com_1046_neg	23-Nordeoxycholic acid	--	C23 H38 O4	378.27667	8.244	377.26938	83974998	86681217.71	86235210.89	85945394.88	43320144.22	46400339.33	35716216.41	36922832.42	40336685.38	54374689.07	39048179.13	28466183.2	33874326.24	35064234.84	33084708.13
Com_1743_neg	5α-Dihydrotestosterone	--	C19 H30 O2	290.22414	7.293	289.21689	534015.7273	481777.8944	547919.2044	630079.2295	988797.6097	703266.2499	40473464.25	43553309.37	47424172.32	30233047.96	1069178.429	42235418.45	15538515.59	15702070.91	16228135.22
Com_1459_neg	3-[4-methyl-1-(2-methylpropanoyl)-3-oxocyclohexyl]butanoic acid	--	C15 H24 O4	268.16717	6.496	267.15982	59589201.99	70727383.9	58045122.3	59168503.53	44193552.38	55964864.14	27609651.62	10497041.31	22821368.72	25236557.15	14076727.19	9627850.903	37599550.75	42683880.77	41057994.76
Com_1424_neg	3-(3,4-Dihydroxyphenyl)-2-Methylalanine	--	C10 H13 N O4	211.08425	5.135	210.0769	56049987.03	45774118.29	69346755.85	57522533.98	63745928.77	50310347.31	10368840.68	6805516.119	7547412.402	9046245.418	12310574.67	8489443.5	50954087.07	53227674.74	36746157.32
Com_441_neg	O-Desmethylnaproxen	--	C13 H12 O3	216.07864	5.356	215.07118	159975307.6	190549039.5	219848753.6	216576207.3	163921198.7	176291809.1	3076621.803	882157.4646	849009.1092	2282011.236	866261.8252	932815.0732	109117121.4	111526341.6	108475673.5
Com_2842_neg	6-Methoxy-2-naphthoic acid	--	C12 H10 O3	202.06259	5.351	201.0556	19869245.64	26584180.13	29740511.98	26290393.54	16585201.72	26368399.71	3804211.167	3346960.844	2740128.233	1838668.275	2858852.853	2662237.203	15751803.35	15791338.4	15882384.54
Com_6688_neg	Biotin	Biotin; D-Biotin; Vitamin H; Coenzyme R	C10 H16 N2 O3 S	244.08787	5.452	243.08051	7055624.156	7973552.508	6051178.732	6942249.272	6444106.618	6836369.603	1545329.809	1459523.727	1658857.19	783562.8327	1106659.418	1575455.507	4116234.799	4958732.613	4541942.854
Com_3030_neg	NG,NG-Dimethyl-L-arginine	NG,NG-Dimethyl-L-arginine; N,N-Dimethylarginine; Nomega,Nomega-Dimethyl-L-arginine; ADMA	C8 H20 Cl2 N4 O2	274.09531	5.561	319.09348	16463371.29	15810906.32	14586628.34	12895388.6	15119415.51	13166046.84	14802492.92	14505443.34	20502109.99	18212240.71	18452327.39	19774713.35	12029251.52	16020896.67	14203356.16
Com_1247_neg	(R)-3-Hydroxy myristic acid	--	C14 H28 O3	244.2034	9.01	243.19629	84854889.24	94296725.85	48008840.05	38956754.28	73750052.84	62470632.96	31059513.79	52862605.64	36235862.48	22591166.68	31979977.49	47673645.65	51024928.43	47440645.36	47481652.04
Com_1117_neg	Dimetghyl 4-Hydroxyisophthalate	--	C10 H10 O5	210.05261	5.411	209.04533	51527970.72	58851967.38	59598296.97	75155762.2	53161250.6	61904303.49	64986034.03	59525363.37	61520344.75	59805180.19	37207199.39	73821050.97	51197655.05	55494727.09	51736889.18
Com_5067_neg	O-Acetyl-L-carnitine	O-Acetylcarnitine; O-Acetyl-L-carnitine	C9 H17 N O4	203.11567	4.883	202.10838	6198337.996	3463784.621	2333015.869	1485915.895	2224100.661	2177307.164	1457750.563	1823073.339	2886653.596	6733456.572	10208556.53	1973940.082	3259282.277	3521239.91	3595107.314
Com_967_neg	Bicyclo Prostaglandin E2	--	C20 H30 O4	370.19911	6.203	369.19183	73437329.32	65863258.68	56323864.4	56621888.97	63109113.13	65467435.64	50443220.15	49904122.17	42780283.24	30960232.77	85685137.94	49096899.13	51010787.8	54286884.86	55080693.04
Com_86_neg	Delta-Tridecalactone	--	C13 H24 O2	212.1774	7.258	211.17023	1321805727	1279062273	1342101393	1222895735	1293189537	1365731083	853716674.1	251047026.2	651940284.3	930448769.7	375744049.5	292566715.9	921381586.2	895173653.5	897567698
Com_4639_neg	LPE 13:0	--	C18 H38 N O7 P	411.23827	8.175	410.2309	15065303.34	5849266.243	4120005.003	2644259.024	4114682.808	3205960.239	830841.842	370549.0195	473105.0943	404815.9995	341962.0992	358738.8616	3615269.725	3207272.567	3586851.47
Com_2738_neg	23-Norcholic acid	--	C23 H38 O5	394.2719	7.062	393.26465	32226079.56	16828315.86	10827880.02	10104013.45	13436566.97	14336134.37	3904488.946	2254696.639	1591073.321	6048218.143	3927199.341	3748542.66	8771520.626	8436722.676	9402018.031
Com_2455_neg	Nicotinate ribonucleoside	--	C11 H13 N O6	255.07411	6.53	254.06683	34010787.74	18520464.41	35981268.25	23393295.76	19933271.01	17678869.74	1235171.761	1370141.747	1031980.266	1137063.955	564172.0075	1344087.084	7819124.5	9898407.385	10553774.26
Com_608_neg	Levodopa	3,4-Dihydroxy-L-phenylalanine; L-Dopa; 3-Hydroxy-L-tyrosine; L-beta-(3,4-Dihydroxyphenyl)alanine; Levodopa; Dihydroxy-L-	C9 H11 N O4	197.06861	5.072	196.06134	182517186.4	152719665.1	156283481.9	144781120.6	127726247.9	123557803.1	14082142.08	8818280.562	8620750.587	9253131.628	8865702.327	8449825.098	91540902.21	83778837.51	82503748.49
Com_3661_neg	Asp-Phe methyl ester	--	C14 H18 N2 O5	294.12162	5.249	293.11407	21804036.49	13737341.35	9794702.828	13012202.04	13986892.48	17166692.9	11849695.35	9184854.061	9816816.736	9846237.673	9229178.731	10555739.2	10123276.54	10275298.68	8004106.295
Com_3623_neg	LysoPC 12:1	--	C20 H36 N O7 P	433.22499	7.208	432.21771	8360379.116	7814427.766	12347096.38	18885074.58	9428411.404	7938212.571	1490082.988	1069075.502	709049.1953	3259825.613	1401365.922	1069260.349	5744823.312	6906554.867	5580746.284
Com_2494_neg	(+/-)-Equol	--	C15 H14 O3	242.09433	5.86	241.08728	4880718.848	8027433.719	6701423.924	6835300.427	6415133.814	6651557.284	19133023.86	20878671.35	23143044.46	23336953.72	20633039.72	21068066.7	8541186.589	9773543.033	11779458.76
Com_3901_neg	Docosatrienoic Acid	--	C22 H38 O2	334.28689	11.072	333.27969	15668148.41	22816185.39	13389432.1	6268117.058	18038342.03	8826367.39	2199067.695	4324831.253	1664687.013	736266.4041	1413574.006	2972185.821	7980765.697	8584583.103	7523255.152
Com_5231_neg	Catechin	Catechin; (+/-)-Catechin	C15 H14 O6	290.07894	5.181	289.07144	8104345.291	2560898.598	1740240.676	1682047.315	2585158.716	2552400.053	5291861.968	5790374.522	10300315.14	1379572.818	2855704.716	4846634.328	2016683.191	1776254.46	1780309.514
Com_3878_neg	N-Oleoyl Glycine	--	C20 H37 N O3	339.27705	10.231	338.26978	6317515.065	11787022.85	19449411.83	6608827.525	13770803.23	6211768.746	1838880.646	2787428.306	1496664.903	1987797.972	984200.983	1514953.114	5423886.984	7631038.198	5641424.667
Com_448_neg	α-Lactose	--	C12 H22 O11	388.12144	1.449	387.11395	7630999.908	1687910.439	7681192.878	4105156.301	11144073.62	12590945.55	19394784.74	86119740.42	30638159.83	3800403.317	196439015.5	18364554.52	10810135.96	8536455.472	10588307.21
Com_627_neg	Docosapentaenoic acid	(7Z,10Z,13Z,16Z,19Z)-Docosa-7,10,13,16,19-pentaenoic acid; Docosapentaenoic acid	C22 H34 O2	330.25548	10.174	329.24814	168105835.3	202273079.7	147464320.7	97553496.01	107763551.5	135673250.4	74950347.33	59302938.92	92094829.02	90891900.42	64482743.1	54393577.85	97787665.28	91533970.85	91664923.2
Com_1522_neg	Syringic acid	Syringic acid; Syringate; 4-Hydroxy-3,5-dimethoxybenzoate; Gallic acid 3,5-dimethyl ether	C9 H10 O5	198.05251	5.352	197.04507	54444551.45	64747694.72	63825851.18	50939300.4	46030397.35	44928480.63	30169521.22	16000013.41	24083571.96	15101460.72	14954429.88	19220979.97	35825026.97	42566219.28	31985819.7
Com_963_neg	Adrenic acid	7,10,13,16-Docosatetraenoic acid; (7Z,10Z,13Z,16Z)-Docosa-7,10,13,16-tetraenoic acid; Adrenic acid	C22 H36 O2	332.27112	10.589	331.26379	89456817.46	126478742.9	69591003.71	45040448.29	73428560.55	62275751.17	7649238.815	8858617.822	10406386.84	10033604.43	7745250.872	6579375.923	37025296.24	39279707.7	33300158.1
Com_3599_neg	13,14-dihydro-15-keto Prostaglandin A2	--	C20 H30 O4	668.4286	7.702	667.42133	14441196.69	15702730.65	21649256.94	14804796.53	4797400.352	12279515.78	71189.72833	52242.60454	57066.61345	54981.73663	121343.2917	58592.09336	3117258.295	3182150.177	2835092.532
Com_4226_neg	Undecanedioic acid	--	C11 H20 O4	216.13575	5.673	215.12845	17641299.07	15407892.12	11324254.89	14330493.1	13641036.43	12157114.19	11135188.65	7930108.548	9523360.179	8761399.454	10294210.89	7238121.228	8964958.92	13810757.08	11227231.92
Com_78_neg	Hesperetin	Hesperetin; 3',5,7-Trihydroxy-4'-	C16 H14 O6	302.07864	5.874	301.0712	200515141.7	180682047.2	179965076.7	189885324.3	213653950.9	203212772.5	762108691.8	845975762.5	839075167.4	1021172278	677429445.3	756705347.9	427111790.9	403946716.2	441157060.2
Com_1208_neg	all-cis-4,7,10,13,16-Docosapentaenoic acid	--	C22 H34 O2	330.25545	10.33	329.24817	51517417.2	74058366.72	84204392.16	50629171.01	41977672.26	38469704.98	6073553.563	4395436.593	6876211.457	6815995.107	5762701.917	4350583.971	25088875.29	29126405.36	23278441.68
Com_992_neg	Monobutyl phthalate	--	C12 H14 O4	222.08888	5.335	221.08154	18469716.83	19984220.81	13776079.56	18664746.29	9764832.416	17705870.07	25323160.37	44804780.44	68237392.35	68643568.47	7261339.395	60862547.96	33503952.43	29164419.77	18091447.28
Com_5718_neg	N-Oleoyl dopamine	N-Oleoyl dopamine	C26 H43 N O3	463.33629	7.973	462.32901	6802231.389	6257166.32	8601743.641	9146983.634	1502652.948	4690389.192	4799137.184	4225994.137	5217477.835	5711579.152	1764157.742	5696804.505	5977320.994	5805954.053	4854875.988
Com_2559_neg	Palmitic Acid	Hexadecanoic acid; Hexadecanoate; Hexadecylic acid; Palmitic acid; Palmitate; Cetylic acid	C16 H32 O2	302.24518	7.994	301.23792	4318100.907	4542279.041	4283652.279	7429639.519	6660817.629	3660317.14	22098225.78	12352964.22	16910816.72	22501417.47	6978661.868	9830954.109	8527635.108	9216590.861	11353717.92
Com_8851_neg	Lysopc 16:1	--	C24 H48 N O7 P	493.3183	8.403	492.31091	3264064.804	5170895.79	4678078.384	3373163.523	3927277.597	4123310.015	381630.7554	241985.2977	550160.9194	578146.7102	306719.6658	423820.0597	1664786.88	2274609.318	2323880.997
Com_3813_neg	4-Pregnen-17alpha,20alpha-Diol-3-One	--	C21 H32 O3	332.23488	8.88	331.22748	20654613.34	19717821.99	9321182.308	8279097.995	8878701.148	5271038.858	363761.0947	330570.6058	407019.4556	350638.3444	692310.9552	395642.4719	5821087.698	5354655.289	5644998.489
Com_2062_neg	Estrone	Estrone; 3-Hydroxy-1,3,5(10)-estratrien-17-one	C18 H22 O2	540.32899	6.637	539.32172	46416951.81	37727790.22	33351217.18	36008432.64	37122773.86	31520057.29	11736400.46	23848297.2	17583078.12	29309327.43	18148299.26	28307083.2	31368820.87	27896741.54	34049996.54
Com_3718_neg	Thr-Leu	--	C10 H20 N2 O4	232.14211	4.867	213.12439	5221940.936	2759984.56	3244315.725	3710370.873	21321528.39	14204894.15	446554.2474	2794622.845	980908.5957	3398346.438	600809.3831	1705940.095	4315461.516	3505660.61	4057851.272
Com_9051_neg	1-O-(3,4,5-Trimethoxybenzoyl)-beta-L-	--	C16 H22 O10	420.12687	5.239	419.12042	1818991.203	2421787.371	3288525.453	1749323.742	1504029.304	1865407.473	3758613.484	2709620.778	3847493.338	1979119.423	311933.6436	3270484.522	1365646.095	1538893.387	1978058.149
Com_4484_neg	3b,7b-Dihydroxy-5-androsten-17-one	--	C19 H28 O3	304.2035	8.227	303.19629	2931300.262	3412235.233	12201218.85	13608104.43	8891352.594	6038070.83	668013.2901	358975.8154	650935.2999	811549.9808	345316.4864	737299.3492	4654237.286	5479015.538	4926271.058
Com_5290_neg	Vitamin B2	Riboflavin; Lactoflavin; 7,8-Dimethyl-10-ribitylisoalloxazine; Vitamin	C17 H20 N4 O6	376.13695	5.657	375.12967	12188628.35	12505438.14	4035509.057	4095446.61	2901940.605	6353624.626	1655705.893	1420834.654	930145.7301	1409797.793	1075398.302	1297719.107	4327300.35	3901070.498	3759240.185
Com_3745_neg	dopaquinone	Dopaquinone	C9 H9 N O4	195.05297	5.594	194.04558	20872534.89	24175315.34	18326787.12	14829467.77	12067728.65	15689679.16	3280615.812	3383661.881	3067316.251	2404114.028	3167094.833	2305960.425	12123178.07	11004212.27	7686154.618
Com_4867_neg	Epoxomicin	--	C28 H50 N4 O7	554.36665	8.665	553.35889	834950.9957	878045.7761	681714.1002	1108670.645	956660.9528	1183610.695	7087879.14	4089304.334	7945503.188	8852801.963	8739859.393	5116759.921	5098618.246	4322227.677	4090595.636
Com_5396_neg	Adrenosterone	Adrenosterone	C19 H24 O3	300.17216	6.583	299.16492	1024596.859	1488390.63	942261.8064	888480.132	1003670.649	1045057.649	1490535.9	1508840.529	9855527.964	2162027.616	1014746.914	6387243.247	1405648.53	1049782.156	955615.4322
Com_6572_neg	Deoxycorticosterone	11-Deoxycorticosterone; Deoxycorticosterone; Cortexone; 21-Hydroxy-4-pregnene-3,20-dione; Desoxycortone; DOC	C21 H30 O3	330.21909	9.134	329.21185	339309.8327	323841.2717	344410.0692	406988.2053	343749.7917	369175.1882	5829098.698	1094006.501	5313636.224	5378101.105	786041.1914	859553.2113	1877902.646	2149215.885	2221111.966
Com_367_neg	9-HpOTrE	--	C18 H30 O4	310.21405	7.318	309.20688	29859130.8	25572642.14	20151355.5	25912885.73	29160890	24273374.64	60184952.74	62627724.58	168679464.7	202768295.7	137535901.1	73701285.98	72867002.33	98327334.02	97235065.18
Com_1_pos	Oleamide	Oleamide; Oleyl amide; (Z)-9-Octadecenamide	C18 H35 N O	281.27132	9.842	282.2785	39940395847	28297798937	32319034280	14739095911	34185107675	23646454396	50202031491	52273160911	41983017788	28133263556	34654427135	53381069076	40488242716	42158255271	41827004130
Com_11_pos	Oleoyl ethylamide	--	C20 H39 N O	309.30274	10.566	310.31	1039517582	222105641.3	745075009.4	282649734.5	1339094252	242410726.5	4697313388	6165832227	1446650262	890550260.6	2058401316	4712738868	3060506452	3122555934	2921872052
Com_18_pos	5,6-dimethoxy-2-(2-methoxyphenyl)-4H-chromen-4-one	--	C18 H16 O5	136.0534	5.703	295.09604	9126167976	9042092836	7995601295	8460435909	7888814471	8630387928	3088142360	2365688245	2842399572	2879739020	2408997297	3061543732	5132313935	5243463208	5240992078
Com_23_pos	Ursodeoxycholic acid	Ursodiol; Ursodeoxycholic acid; Ursodeoxycholate; 3alpha,7beta-Dihydroxy-5beta-cholan-24-oic acid	C24 H40 O4	392.29213	8.271	393.29907	1292262409	2166645790	4056140991	5523360398	599450000.4	1520008863	833081816	552986519.9	3791527428	4254235330	955039402.6	1244692815	2070428963	2165825754	2122765571
Com_64_pos	Monoolein	--	C21 H40 O4	356.29212	10.022	357.29929	82482977.76	221669183.3	111452960.1	52302426.32	177880682.2	89285063.42	1147542684	1505689775	339302017.6	82010991.79	194406367.5	1421521655	626927117.5	652014045.3	634006647.3
Com_135_pos	5-[(10Z)-14-(3,5-dihydroxyphenyl)tetradec-10-en-1-yl]benzene-1,3-diol	--	C26 H36 O4	412.25843	7.439	413.26569	593516238.3	735501700.6	921440697	1020854633	602321395	1111502876	503908237.7	7606536.76	856616161.6	648615784	790343142.2	517490568.3	731607191	587331536.4	595312289
Com_184_pos	3-Amino-4-methylpentanoic	--	C6 H13 N O2	131.09462	2.405	132.1019	209139855	258028742.9	162926601.4	307838262.3	253942745	240582030.4	14250861.96	88638396.47	95569933.34	621808483.1	8515369.152	79035024.36	218529942	185726161.7	171672060.6
Com_224_pos	octadec-9-ynoic acid	--	C18 H32 O2	262.22915	7.998	263.23663	268007944.6	276783486.2	207973002.8	166885948.4	396682262.5	306622435	471540691.6	312115851.2	376541346.5	529419819	266952658.3	361669374.7	357152407.8	350542568.2	342215345.9
Com_233_pos	(+/-)11(12)-EET	--	C20 H32 O3	302.22429	7.633	303.23157	19258421.81	17610783.89	26932285.05	25945021.68	16205643.01	19728280.79	103653138.6	41121720.85	63218349.27	129729170.5	522226941.5	53404024.01	96673644.71	93035698.71	107640980.6
Com_235_pos	N-Tetradecanamide	--	C14 H29 N O	227.22479	8.831	228.23222	547999532.8	467398362.8	426102988.1	271425885.3	424138907.6	455751090.3	497315300.8	385983033.7	438235521.3	281304312.5	312492247.4	536862094.3	411718862.3	406033599.2	399272637.7
Com_258_pos	Beta-Muricholic acid	--	C24 H40 O5	408.28731	7.155	409.29459	9528948.825	8291752.185	20404475.48	18922847.62	14948196.61	10274935.46	387945182.8	275934289.1	143460579.4	52161851.28	483696740.4	292869460	207846514.4	214821970	205959482
Com_270_pos	Diosgenin	Diosgenin; (3beta,25R)-Spirost-5-en-3-ol	C27 H42 O3	414.31312	10.272	415.32056	6776008.682	8867419.088	8705847.119	6591870.592	9048762.089	7175923.842	198229126.5	387096297.7	156656429.7	121752332	30711773.05	339554141.7	174535669.3	121149897.4	184795153.7
Com_276_pos	Normorphine	Normorphine	C16 H17 N O3	271.12068	4.884	272.12802	1909974.774	2166041.662	1641576.668	1409346.566	1726825.625	1324958.355	83597758.1	374000900.1	350165911	51136454.67	4306824.256	305415552	76699288.65	68450343.91	66593191.13
Com_293_pos	2-[2-(2-pyridyloxy)ethoxy]pyridine	--	C12 H12 N2 O2	198.07916	5.481	199.08638	629728265.4	657805344.1	472794715.2	508235091.9	661231365.6	670992044.2	2352694.79	871888.7348	713335.6989	817739.8957	3952801.907	1536285.695	246822214.6	237388993.8	224549557.6
Com_295_pos	N-Stearoyl taurine	--	C20 H41 N O4 S	408.30261	8.979	409.31	18747643.14	19068183.17	12315659.68	15835265.53	244878287.5	212304992.1	72801039.27	55507311.76	59667629.13	75787536.53	422181778.8	48076545.7	97981573.54	98331981.65	97508190.32
Com_324_pos	Morphine	Morphine	C17 H19 N O3	285.13629	4.884	286.14346	166101.5274	317152.8388	313998.9109	318781.7537	367630.4407	316477.6766	54014785.52	329353343.7	271167571	64827290.4	2054170.002	252857557.9	52464578.91	48714453.68	47369963.04
Com_362_pos	Octadeca-11E,13E,15Z-trienoic acid	--	C18 H30 O2	278.22436	9.565	279.23175	163655323.5	175606605.4	197230293.3	117574263.3	242352430	250931388.6	193568254.6	240879654	377240496	219121614	230694173.9	197333226.2	226221179.3	231038837.2	211864005.3
Com_365_pos	Prostaglandin B1	Prostaglandin B1; PGB1	C20 H32 O4	336.22971	6.816	337.23703	323498995	241992296.1	320831470.7	444173667	161212646.9	188526798.9	30616887.57	19418648.92	22231611.85	22690241.28	289469031	43406070.87	159334772.7	141905712.3	158559248.3
Com_379_pos	DL-Norvaline	--	C5 H11 N O2	117.07904	1.298	118.08627	228032342.9	62926487.32	176022541	345344573.5	216039624.8	334629614.5	50196732.64	279629239.7	120497911.8	174930244.3	85754142.52	195816838.2	193584357	182613761.6	178938674.1
Com_392_pos	(+/-)5(6)-EET	--	C20 H32 O3	320.23483	6.684	321.24225	395013055.6	390621384.5	466194081.5	411425965.6	358229741.4	416665348.4	64059254.2	46827358.18	46489224.42	75196460.64	53477496.48	32864753.77	198046252.8	199441421.2	203615857.6
Com_410_pos	Harmine	Harmine	C13 H12 N2 O	212.09478	5.484	213.10207	411393905.1	471095262.1	372189801.3	335511495.1	471342603.5	540134014.6	2017341.969	1129112.357	1085167.959	1173582.931	4080231.272	1392929.291	210209604.1	214588389.1	148111008.6
Com_466_pos	Leucylproline	--	C11 H20 N2 O3	228.14747	4.976	229.15482	108955722	121526000.9	65465349.6	87135578.4	259043026.7	261484973.3	12906444.12	44767594.95	52135308.54	268727151.6	18345220.85	46400764.97	112706429.9	99123975.85	101024674.9
Com_486_pos	(11E,15Z)-9,10,13-trihydroxyoctadeca-11,15-dienoic acid	--	C18 H32 O5	350.20681	7.143	351.21402	57663347.63	63397526.52	52078542.23	54850923.63	49265992.66	52023114.62	120526297.9	103960401.5	290702494.3	241281799.2	130933691.1	123666828.5	115100070.6	89380950.04	100334761
Com_499_pos	2,4-diaziran-1-yl-6-(1-phenyl-1H-pyrrol-2-yl)-1,3,5-triazine	--	C17 H16 N6	304.14421	4.97	305.15146	3923436.196	1787736.791	1699564.026	1739977.01	1642749.845	1581334.941	257001193.6	1327707.602	33103810.82	21558948.34	69005629.03	1501088.749	28227098.02	27537779.45	25027266.68
Com_500_pos	(+/-)5(6)-EET Ethanolamide	--	C22 H37 N O3	345.26665	5.175	346.2739	196565.7109	205199.2977	186055.1179	170488.9355	185749.4097	198697.2502	37465744.06	22396689.23	10562708.82	13534395.47	255597800.1	24412388.05	28112757.35	26161984.55	26920336.7
Com_516_pos	Thromoboxane B1	--	C20 H36 O6	394.23289	6.902	395.24017	14111219.09	13203988.84	10552097.71	11143698.21	12730004.89	14399152.6	35382990.37	16747524.89	14741624.99	15433822.1	247369896.9	21232110.15	36618869.37	31442447.09	36796285.96
Com_521_pos	1-Methyladenosine	1-Methyladenosine	C11 H15 N5 O4	281.11312	6.196	282.12039	30729604.06	21782766.14	18991502.23	20781576.52	31384296.49	33435039.12	100377516.9	138401551.4	132607493.8	238960376.1	11810363.64	119202130.9	80835754.69	65470874.49	58288706
Com_523_pos	13-HPODE	--	C18 H32 O4	312.2293	7.291	313.23685	137980774	197379538.2	100829365.1	193657643.9	236334755.6	196066922.7	130671901.9	187619094.6	266567111.9	115620321.2	214640159.4	168892733.1	128666103.9	125994369.4	112466403.5
Com_540_pos	(2E,4E)-N-(2-methylpropyl)dodeca-2,4-dienamide	--	C16 H29 N O	251.22474	8.67	252.23209	361979930.3	264529671.1	161171062.7	144704217.3	183025682.3	253757747	130354618.6	113554700.7	174324031.7	91998541.2	128445847.9	202701986.7	157978402.7	159507224.9	156707870.7
Com_541_pos	2-(2,6-dimethoxyphenyl)-5,6-dimethoxy-4H-chromen-4-one	--	C19 H18 O6	324.0994	5.594	325.10663	307343609.9	343321124.3	305457580.7	307601706.8	329301093.7	361249591.4	73295827.54	88036077.43	85939556.01	92471313.39	66600459.66	94916548.09	188519377.5	179673135.4	185580542.7
Com_574_pos	Avocadyne 1-acetate	--	C19 H34 O4	308.23478	7.95	309.24207	13842507.38	10810959.8	11930078.31	19268137.68	9876114.515	9828967.277	75143036.86	190043224.4	52949047.13	122383351.6	45780194.74	97431423.28	59232786.22	56047591.03	59086415.23
Com_596_pos	RKK	--	C18 H38 N8 O4	430.30535	11.35	431.31262	10568995	13255531.75	17161203.07	11054703.97	24764993.57	9445963.073	141231357.4	63719976.6	154647579.4	16349827.53	132734942.2	224509852.3	64355603.57	73617675.08	72562674.35
Com_620_pos	5-phenyl-3-(phenylmethylene)furan-2(3H)-one	--	C17 H12 O2	248.08356	5.658	249.09094	142282916.7	147962092.9	126141057.2	148143011.8	139459143.3	139547696.3	179880326.6	157984215.2	179541946.4	202127346.5	104283413.1	174970797.7	127398112.5	124706990.5	123999289.7
Com_622_pos	1,2-dihydroxyheptadec-16-yn-4-yl acetate	--	C19 H34 O4	348.22959	6.821	349.23672	39965318.06	48788065.64	34199638.47	62564412.84	37690592.95	25754235.03	20125236.11	175576676.1	15974037.99	20418178.66	113412921.7	86889908.82	30828067.82	30859762.52	30636707.59
Com_655_pos	(2R)-2-[(2R,5S)-5-[(2S)-2-hydroxybutyl]oxolan-2-yl]propanoic acid	--	C11 H20 O4	216.13605	5.943	217.14319	293828349.9	277206274.1	127514305.5	128090148.5	223061630	212514369	29733142.31	32540364.86	40520040.59	49684460.02	37469845.97	34480615.21	118445249.8	106611872.6	106296493.1
Com_688_pos	PC (16:2e/2:0)	--	C26 H50 N O7 P	519.33202	9.12	520.33936	32826963.23	42101805.71	40722790.52	14014661.6	41938407.76	33980378.29	23936773.3	155900380.6	21095342.7	6553989.587	16852190.96	11813086.41	33048616.99	29456528.89	32166068.31
Com_701_pos	Styrene	Styrene; Phenylethylene	C8 H8	104.06294	4.795	105.07028	98659672.65	174008441.1	109782866.3	74168681.02	281481126.6	320068618.8	11732206.15	29527551.91	24733481.47	31508874.97	9790732.626	52564968.41	105411461.3	91182374.6	87049429.29
Com_711_pos	7-(2-hydroxypropan-2-yl)-1,4a-dimethyl-decahydronaphthalen-1-ol	--	C15 H28 O2	222.19822	7.734	223.20547	5078660.743	6339568.197	3901979.117	8110218.142	5522597.531	3935568.104	82785862.1	60015717.33	83041714.53	175462148.9	27493974.24	70357670.13	49532532.29	46813316.25	54373385.12
Com_731_pos	Hydroxyprogesterone caproate	Hydroxyprogesterone caproate; Primolut depot; 17alpha-Caproyloxypregn-4-ene-3,20-dione	C27 H40 O4	428.2923	6.618	429.29962	13566809.35	12356177.25	10224929.38	13195868.28	14856715.98	15361111.2	17532712.46	9334044.233	12004848.87	19558212.8	175334061.8	8973179.928	25064833.99	27157208.65	25745775.67
Com_751_pos	13-OxoODE	13-OxoODE; 13-KODE; (9Z,11E)-13-Oxooctadeca-9,11-dienoic acid	C18 H30 O3	316.20122	7.821	317.20853	31321866.25	28982125.56	27110809.22	33382459.53	40368827.93	34297151	76876367.28	98867205.11	160535895	166072312.3	76035329.65	73825174.02	78830728.97	78174124.44	61901457.74
Com_779_pos	Thromboxane B2	Thromboxane B2; TXB2	C20 H34 O6	392.21741	6.156	393.22461	174325727.6	177626320.8	155020359	141588402.9	122768522.3	153156960.8	104108836.4	91183495.3	101343658.5	98079977.39	166188760.6	84642688.77	114907753.4	108789595.7	113773660.5
Com_790_pos	α-Aspartylphenylalanine	--	C13 H16 N2 O5	280.10963	5.652	281.11682	222692950.2	222536288.1	139275355	143065734.9	236305880.9	238961162.6	57694315.9	74084502.63	73807379.37	114819317.5	61961451.46	75811906.68	148744003.7	148129674.6	143975671.9
Com_825_pos	2-(14,15-Epoxyeicosatrienoyl) glycerol	--	C23 H38 O5	394.26937	8.409	395.27664	2467810.559	2249172.99	1612728.42	2215749.992	2094513.609	2988589.284	45368400.44	129797403.5	82179409.92	27077036.29	11304182.51	21614099.88	33797991.99	40771351.65	43419044.06
Com_849_pos	2-Oxindole	--	C8 H7 N O	133.0527	5.69	134.06006	91811953.85	125270928.7	151391155.2	158945471.1	184884072.1	260298809.2	30011103.3	47831460.46	130778624.5	95667079.47	148715097	66792907.35	114653360.4	116959256.4	116119885.6
Com_877_pos	L-Alanyl-L-Lysine	--	C9 H19 N3 O3	217.14239	1.352	218.14946	8915802.076	9367311.468	6609930.277	6790953.792	11666958.04	9733182.711	5485664.147	9911800.321	11836130.36	15815748.6	143873146.9	13701031.31	21962174.29	19103510.1	19428108.5
Com_900_pos	11β-Hydroxyandrosterone	3alpha,11beta-Dihydroxy-5alpha-androstane-17-one; 11beta-Hydroxyandrosterone	C19 H30 O3	306.21914	7.66	307.22641	19515174.51	17662768.04	16737628.15	16954473.33	15731379.22	17095847.51	73050189.91	117837765.5	42148030.75	60995339.35	59618583.12	83515142.13	48314374.11	46505674.55	47507844.62
Com_986_pos	16,16-Dimethyl prostaglandin	--	C22 H34 O4	384.2296	6.623	385.23685	55417681.04	36842113.69	27216158.89	79208568.06	33949276.88	41251339.55	10691620.79	108101888.9	9356010.265	21419091.82	42309271.01	50441945.16	39433051.21	39072383.78	38231887.4
Com_990_pos	α-Linolenoyl ethanolamide	--	C20 H35 N O2	321.26639	8.795	322.27374	22011219.56	66081486.28	27305372.64	7839656.348	17159279.33	11508368.05	78271983.95	107518141.8	126905155.6	89401502.96	115429875.1	84461786.26	61569005.42	57486348.3	61572984.76
Com_1025_pos	Lysopa 18:0	--	C21 H43 O7 P	438.27477	8.218	439.2818	2028947.396	2418706.993	3414416.479	2964418.729	3136693.465	3304038.941	33780512.38	104141444.6	15142384.73	42930317.33	100157629.5	69544873.29	58699090.12	66436847.91	54421430.05
Com_1029_pos	2,6-Di-tert-butyl-1,4-benzoquinone	--	C14 H20 O2	238.15689	5.991	239.16409	186922602.2	172364770.3	130792333.6	116195986.7	96577965.72	94909591.92	30014631.1	23246374.38	35356422.62	42628332.21	27013598.64	28014884.7	87161728.46	117653852.9	81637290.61
Com_1030_pos	Alloxan	--	C4 H4 N2 O5	160.0127	1.031	161.02008	186761958.6	196551444.8	121244784.8	98665152.21	144883336.2	111639098.7	39525825.61	32919558.16	54574093.78	51739047.1	36085904.39	21823768.84	95516531.54	76665145.95	67465856.45
Com_1055_pos	D-Proline	D-Proline	C5 H9 N O2	115.06343	1.338	116.07079	49738305.02	41655782.33	39882239.87	62271834.37	83724815.97	70707542.57	25394887.16	79183752.28	50638313.37	115855357.5	78159850.4	70506105.41	68770886.66	55305004.05	55984797.95
Com_1057_pos	11-Deoxy prostaglandin F1α	--	C20 H36 O4	362.24265	8.292	363.24997	3977249.252	2484257.202	2104769.197	2628849.667	3925572.23	2135893.601	60853086.33	100278789	42343469.83	30229400.48	117501236	88010212.1	45603666.45	43828396.33	45539892.69
Com_1062_pos	Testosterone undecanoate	--	C30 H48 O3	456.36018	8.992	457.36752	58731630.56	33480983.34	46206832.05	48883372	83770543.92	74778192.43	93149293.59	99700374.77	94557526.17	107561571.7	63805878.57	99899314.83	81171442.46	85820711.3	75626874.17
Com_1079_pos	2-Amino-1,3,4-octadecanetriol	--	C18 H39 N O3	317.29259	6.037	318.2999	58132416.49	54085666.81	46946980.59	38793636.05	45020199.77	61101846.41	101835862.6	57379774.83	75409509.69	66105545.18	37454255.36	118976059.9	58067438.55	57098676.11	56598864.03
Com_1082_pos	3-hydroxy-2-octylpentanedioic acid	--	C13 H24 O5	242.15178	6.095	243.15891	54252724.97	58500918.29	35808688.08	37253707.48	42119147.65	42385975.5	109188740.9	79352034.03	125585469.1	67553652.82	78099128.78	97059457.62	47232410.08	53693057.2	76713676.31
Com_1105_pos	Ciprostene	--	C22 H36 O4	346.25039	8.194	347.2576	134221310.3	68370369.61	106188002.2	145381221.4	86494868.32	88231264.94	16052872.89	15004174.31	14419128.24	27078506.09	14557203.61	15990897.79	44838078.85	48095118.14	53994618.68
Com_1132_pos	Maslinic acid	--	C30 H48 O4	494.33681	9.045	495.34409	16829327.3	14794731.55	14152235.9	15015427.26	25948916.36	21382964.52	64549309.32	53072032.27	77217463.31	106449416	22942307.68	51398957.77	47811532.52	47006420.75	46111899.08
Com_1145_pos	Enalaprilat	Enalaprilat; Enalaprilate	C18 H24 N2 O5	348.16976	5.115	349.17703	986442.2073	1145380.418	926166.5406	774191.9028	1004744.675	1213198.86	108344424.6	3425315.65	36173861.78	28425080.7	12012749.23	1959848.226	22073069.51	25950897.49	25553085.11
Com_1177_pos	PC (14:0e/2:0)	--	C24 H50 N O7 P	495.33419	9.661	496.34164	57297499.03	64968515.12	105047995.3	136936022.8	93171119.42	97564242.28	13620347.37	15489569.59	27781639.87	44169108.71	14410928.47	17582834.04	47353433.27	26300623.75	46549178.61
Com_1201_pos	Prostaglandin F2α-1-glyceryl ester	--	C23 H40 O7	450.25895	7.393	451.26624	2464735.967	2249950.432	2810944.41	2516931.371	2352761.043	3648619.725	1023768.084	1158872.533	48562235.41	100372167.2	1093407.908	909615.7198	11169308.35	14633119.05	11670753.06
Com_1217_pos	Prostaglandin K2	--	C20 H30 O5	332.19599	7.069	333.20328	100550614	32091701.16	92795999.64	24879948.65	86209698.52	95041082.99	42926344.28	59274490.96	88933848.97	99225020.7	44985111.56	56844906.11	79938181.22	77302203.44	73366455.86
Com_1220_pos	4-methoxy-6-(prop-2-en-1-yl)-2H-1,3-benzodioxole	--	C11 H12 O3	192.07863	5.846	193.08591	138342323.1	121432533.7	93235790.31	132342017.8	107507849.1	119126385.9	54632211.29	47521394.64	60789947.76	51183629.63	52794222.61	52235981.36	80825973.77	64777305.5	49172904.24
Com_1223_pos	Prostaglandin I2	Prostaglandin I2; (5Z,13E)-(15S)-6,9alpha-Epoxy-11alpha,15-dihydroxyprosta-5,13-dienoate; Prostacyclin; PGI2; Epoprostenol	C20 H32 O5	352.2226	7.049	353.2305	31736770.51	48577411.62	40011468.2	39338742.66	73538286.1	42338365.02	56259435.33	60833437.78	111359327.9	94326660.78	96558447.34	57186202.89	53001123.99	56859930.06	53306838.64
Com_1225_pos	3-(propan-2-yl)-octahydropyrrolo[1,2-a]pyrazine-1,4-dione	--	C10 H16 N2 O2	196.12128	4.935	197.12856	22657789.04	19463665.09	3342815.27	5458032.774	158760824.2	143157554.7	596387.5266	17031124.57	635831.1161	1665572.916	656245.5386	24724277.92	25392768.57	22033442.81	24895173.42
Com_1255_pos	Palmitoleic Acid	(9Z)-Hexadecenoic acid; Palmitoleic acid; cis-9-Hexadecenoic acid; (9Z)-Hexadec-9-enoate	C16 H30 O2	276.2087	7.046	277.21597	79606438.95	63734324.89	60605246.94	66635497.27	52172730.37	47887830.17	55256371.67	56343984.94	79859021.04	93438854.43	99136787.58	54501042.74	58430845.98	61419877.74	57424719.64
Com_1269_pos	(3beta,9xi)-3-(beta-D-Glucopyranosyloxy)-14-hydroxycard-20(22)-enolide	--	C29 H44 O9	536.29563	8.688	537.3028	956627.202	4242613.181	1343897.398	952155.674	1768178.772	1083029.757	97937163.86	74617991.74	33473712.43	3228453.054	36129115.89	72503512.77	27680498.87	30261936.04	27553237.2

Com_1299_pos	3-methoxy-2-phenyl-4H-furo[2,3-h]chromen-4-one	--	C18 H12 O4	292.07326	5.592	293.08057	135111534.2	134051267.8	126378841	123796183.5	141476481.5	150014766.7	33396737.42	36444784.45	40887032.7	39835743.21	29611083.11	45996563.94	81357287.55	80906356.19	79327289.21
Com_1301_pos	Sphingosine (d18:1)	--	C18 H37 N O2	299.28216	9.547	300.28952	60370761.02	83085440.75	68770042.74	32603171.2	63697006.86	56528771.5	40736815.62	65241440.1	80616834.22	72886372.64	45086149.25	70375965.97	52712520.63	57048181.31	56501862.19
Com_1305_pos	Sparteine	(-)-Sparteine; Sparteine	C15 H26 N2	117.10471	3.998	235.21642	183297.8668	204697.8826	157109.6844	144113.9775	170339.6243	189602.8343	12167607.8	94147.77615	5119601.611	13729168.97	94825139.24	132108.4725	26137564.5	23745136.55	27204877.35
Com_1341_pos	3-(3,4,5-trimethoxyphenyl)propanoic acid	--	C12 H16 O5	262.08178	5.5	263.08908	4964656.8	5168055.273	2355669.092	1305707.07	4656410.038	4852081.759	16061411.61	6046907.917	9955249.623	8180822.254	91856157.1	6269551.154	10687984.29	7840476.081	8215087.955
Com_1360_pos	4-methoxy-9-(2-methylbut-3-en-2-yl)-7H-furo[3,2-g]chromen-7-one	--	C17 H16 O4	284.10452	6.052	285.11179	78453016.35	66651131.43	87010736.15	117990238.6	57323114.56	82084716.4	12822145.27	10985107.87	13179050.48	13980831.59	6967486.391	12145953.28	47840643.02	44196460.37	42801974.98
Com_1372_pos	LNH	--	C16 H26 N6 O5	382.19649	5.967	383.20367	123369937.9	115718260.4	130415813.1	96514112.17	102127931.5	107942888.1	42183796.08	48510080.76	49593660.18	61106603.84	43860663.03	52860543.74	51261801.1	49915378.79	76483173.55
Com_1389_pos	8-Isoprostaglandin F1α	--	C20 H36 O5	338.26391	9.32	339.25018	85449477.92	152975068.4	39843189.46	34763140.18	107515097.2	96371563.98	20614703.1	24965297.15	17339727.35	14502392.35	20990932.36	20822914.04	39114741.39	39069910.14	33796087.25
Com_1393_pos	Doxycycline	Doxycycline	C22 H24 N2 O8	444.15672	5.519	445.16394	48871385.88	45570688.2	83475380.37	115271294.7	61198935.36	66639408.82	45090782.32	15417092.4	6802604.429	10576762.47	11316777.476	14778108.65	38874203.52	41817242.65	42138157.92
Com_1397_pos	5-[(E)-2-(3,5-dihydroxyphenyl)ethenyl]-2-methoxybenzene-1,3-diol	--	C15 H14 O5	274.08385	5.519	275.09116	82559203.1	145664169.8	128522457	110999525	101384411.6	137401182.3	49516040.04	43014097.77	67729450.19	53685412.95	45553862.6	53226275.89	61434655.54	58097488.29	59934997.6
Com_1400_pos	Andrographolide	Andrographolide	C20 H30 O5	350.20762	6.247	351.21494	78582709.51	82952485.97	99328366.59	66939670.84	65367300.39	69733842.9	52183843.46	10606580.56	67345067.63	85874797.45	44475572.32	40612610.57	53197843.5	49047693.5	51483324.45
Com_1414_pos	Nootkatone	Nootkatone	C15 H22 O	218.16692	8.835	219.17424	13627743.24	12824990.32	8784717.247	10052942.13	22037163.39	21481727.77	49240910.37	51562349.28	52018136.11	84743900.08	26810610.32	54231418.07	34267734.65	35037259.39	33299977.47
Com_1419_pos	5-(2,5-dihydroxyhexyl)oxolan-2-one	--	C10 H18 O4	224.10469	5.58	225.11192	84463247.91	68463338.46	90101677.21	52559993.61	19859603.3	107662986.7	86973192.02	41518831.45	46322632.83	53316032.69	64687599.58	50710970.49	62457615.54	71631457.29	60199787.52
Com_1427_pos	dihydrotachysterol	Dihydrotachysterol	C28 H46 O	398.35433	10.234	399.3616	85783689.05	124729149.1	125457702.4	6180681.931	78999429.42	50343404.89	6341944.868	9080922.019	5290688.086	4308590.332	4546146.012	4879021.305	42211837.46	44329056.49	40665372.87
Com_1487_pos	Allolithocholic acid	--	C24 H40 O3	376.29709	9.204	377.30469	4487284.758	4359540.772	5177357.184	5390411.837	4073230.526	7455876.094	44987435.76	45203106.42	58512139.4	25551539.18	82731883.78	2971176.218	23064699.1	30503528.67	28494406.32
Com_1506_pos	NSI-189	--	C22 H30 N4 O	366.24022	7.656	367.2475	5532053.964	4583713.913	4419993.198	5455718.495	3637817.326	3952159.372	7437081.842	68533464	6017303.814	69793695.457	49878835.8	21809775.35	18196878.51	19114261.84	19239028.71
Com_1528_pos	3-methyl-5-oxo-5-(4-toluidino)pentanoic acid	--	C13 H17 N O3	257.10232	1.275	258.10962	14665792.72	10135492.97	8767046.899	11462573.56	27409235.12	19369663.73	43411615.62	67093354.59	19899425.46	23659328.27	10286028.28	81878818.56	27984609.42	29442458.6	29267312.04
Com_1542_pos	Estriol	Estriol; 1,3,5(10)-Estratriene-3,16-alpha,17beta-triol	C18 H24 O3	288.17232	6.761	289.17957	17112022.95	20458341.51	13592905.65	14428665.08	15029895.85	16201047.4	47512707.28	16786854.82	38591457.94	16150201.99	41718646.35	81047993.48	24308766.93	20700829.26	22751460.81
Com_1544_pos	15-OxoEDE	--	C20 H34 O3	304.2398	6.552	305.24701	31588485.99	32817186.61	36635267.81	38638278.66	42691748.21	51838023.21	60197541.65	23147606.84	46748676.72	76171641.5	29562689.44	23704365.07	38784087.42	34343343.1	34859790.21
Com_1566_pos	2-Thio-acetyl MAGE	--	C21 H42 O3 S	396.26583	7.612	397.25018	4109170.722	3990727.513	3252394.572	4119111.178	4216936.127	3688860.413	18590063.57	11680562.67	12038916.18	12275261.01	76882831.86	13635721.14	13999988.84	13481770.14	14851579.32
Com_1574_pos	17beta-Trenbolone	--	C18 H22 O2	270.16165	7.111	271.16885	8251525.379	9122424.055	5414577.387	5788103.982	8189761.213	9171097.965	44083615.92	64746709.11	56228243.47	54292455.47	26332070.65	35214626.31	22733310.97	27494911.13	25698072.43
Com_1577_pos	N1-(4-cyclohexylphenyl)-2-[(4-	--	C21 H25 N O S	339.16543	5.691	340.17245	18702741.51	15862459.21	12409718.35	9859125.962	15054190.08	28394378.68	9169902.312	64565068.61	39985511.94	39167468.46	2875926.082	55760874.28	25568843.15	16903378.85	18907333.19
Com_1617_pos	Diosmetin	Diosmetin; Luteolin 4'-methyl ether	C16 H12 O6	300.06317	6.264	301.07056	1989249.094	1550166.728	1836266.96	1407054.963	1388713.434	1303822.678	46390090.53	21861874.12	16279848.33	13256127.87	74322355.79	14739427.16	11561395.04	13129158.78	15344031.61
Com_1623_pos	Sebacic acid	Sebacic acid; Decanedioic acid	C10 H18 O4	202.12042	5.942	203.12756	112645281.6	102347489.6	48252455.48	49696137.28	85486865.47	84206401.4	9886769.611	10845597.91	13899844.03	17896948.25	12966058.73	12998906.65	43279243.63	41107134.82	44305985.07
Com_1637_pos	Deoxyguanosine	Deoxyguanosine; 2'-Deoxyguanosine	C10 H13 N5 O4	267.09739	5.548	268.10461	13924015.32	65488733.48	64497066.1	95292107.19	16935993.38	79883516.48	26384086.63	29588644.31	24422184.8	27424390.64	61144057.14	29179131.23	42502044.24	44888820.88	53609111.76
Com_1652_pos	2-{[methyl(2,3,4,5,6-pentahydroxyhexyl)amino]methylidene}malononitrile	--	C11 H17 N3 O5	271.1163	1.305	272.12344	6747240.353	8112942.29	19221868.74	6299876.666	25323483.44	16039435.12	1105602.809	61654026.59	11640988.87	1165223.77	2280119.08	34247036.54	8147077.019	8073270.009	9105497.545
Com_1730_pos	5-oxoproline	--	C5 H7 N O3	129.04252	1.264	130.04987	33011132.73	29107679.81	56572096.55	67862895.51	99608228.71	87671148.68	13672643.92	44316916.18	27082248.8	66745071.36	12995955.22	44215811.75	56285018.52	49327396.77	46877771.83
Com_1735_pos	trans,trans-2,4-Heptadienal	--	C7 H10 O	110.07316	5.704	111.08067	77225800.79	56373104.45	48645189.95	65532271.92	68257981.8	57390798.95	25802286.89	22154677.97	46904013.16	66488645.23	26099542.7	24612396.26	47601390	48560250.24	31015042.63
Com_1756_pos	Anandamide (AEA)	--	C22 H37 N O2	353.29261	10.299	354.29987	4333498.087	4263190.253	15429121.94	10961872.79	8346858.311	6769626.91	13981190.58	47106131.39	31094965.08	65479046.52	10474220.64	48014258.83	19622765.45	25860717.03	22972425.47
Com_1816_pos	tetranor-12(R)-HETE	--	C16 H26 O3	248.17744	7.28	249.18474	10722175.46	11921285.97	12726411.35	14090996.67	12815850.95	12145559.66	46148041.59	49353301.85	49407807.63	26271508	64970081.34	35214362.11	28368601.21	31068338.81	31237690.87
Com_1831_pos	N-Acetyl-D-tryptophan	N-Acetyl-D-tryptophan	C13 H14 N2 O3	246.10039	5.267	247.10765	98559140.05	104115451.1	79437810.78	73804105.35	92527437.92	94099424.94	11427797.14	4388597.24	6052517.122	7199172.323	2960533.064	5679912.064	38776819.78	37197426.02	31903139.32
Com_1836_pos	Stiripentol	--	C14 H18 O3	216.1148	5.692	217.12206	98073704.93	84264610.59	80277332.66	80182821.46	73617488.13	79540368.53	22745696.53	20535999.62	23721381.44	28300571.72	26941072.39	19613601.53	51302721.45	45952519.93	54599694.71
Com_1842_pos	(9cis)-Retinal	--	C20 H28 O	284.21362	7.224	285.22083	5004002.077	3779306.456	6400743.399	5376151.496	4639750.333	4852124.502	9400852.839	19697218.77	31554216.61	61979670.03	7513738.535	17639393.88	16335022.53	17102237.53	16778243.74
Com_1843_pos	5-(6-hydroxy-6-methyloctyl)-2,5-dihydrofuran-2-one	--	C13 H22 O3	208.14615	6.117	209.15347	20873749.7	19548083.74	16479483.6	18574148.92	17863749.79	17939784.1	37872154.68	25527605.52	40178908.8	34829276.72	37409583.3	65874234.86	25313333.93	22202113.3	22747367.2
Com_1845_pos	(+)-ar-Turmerone	--	C15 H20 O	216.1512	7.409	217.15852	4038907.089	3723232.676	3431854.799	4142712.588	3959705.111	3988704.477	7732131.791	13440414.5	40604169.84	61921587.63	38288660.28	9500392.968	18940985.86	19492153.93	18115865.63
Com_1861_pos	7α-Hydroxytestosterone	7alpha-Hydroxytestosterone	C19 H28 O3	304.20353	6.722	305.21078	73112083.95	85076338.63	73656140.29	82154116.95	63077613.96	76078136.6	28138758.51	18356302.29	21735228.63	26146301.43	38384720.5	24058339.37	47735296.28	42697561.49	44000298.39
Com_1874_pos	3-(2-methylpropyl)-octahydropyrrolo[1,2-a]pyrazine-1,4-dione	--	C11 H18 N2 O2	210.1369	5.025	211.14415	21337426.96	22398130.13	2302143.188	2607345.138	98242789.22	41213352.58	263363.8383	12077981.79	382788.9813	971422.638	274145.9884	14243640.99	22336990.18	20875052.36	19845659.76
Com_1909_pos	(12Z)-9,10,11-trihydroxyoctadec-12-enoic acid	--	C18 H34 O5	352.22304	6.64	353.23038	43040888.55	36147337.53	30741627.3	33039213.22	32366859.71	35560891.53	31743394.17	48592143.53	61293359.44	59978876.6	58137990.42	36900035.61	37702354.49	33553232.51	32350996.94
Com_1929_pos	PC (14:1e/4:0)	--	C26 H52 N O7 P	521.3477	9.629	522.3548	31017445.38	67632103.6	88980225.99	25394396.53	64069949.7	39129746.58	15562784.15	7706973.148	6037896.684	13523992.74	8140735.542	5568465.319	23310280.62	18294330.63	23114680.79
Com_1933_pos	Testosterone	Testosterone; 17beta-Hydroxy-4-androsten-3-one	C19 H28 O2	288.20859	7.669	289.21579	16537740.79	13938103.05	14524681.94	14415245.91	10273078.97	14439132.45	35510069.93	51643066.15	17039657.96	28193836.75	30823698.26	36204769.49	23317828.75	21595657.35	23014029.22
Com_1944_pos	(1E,4E)-1,5-bis(4-methoxyphenyl)penta-1,4-dien-3-one	--	C19 H18 O3	316.10563	5.494	317.11292	26918872.27	25273821.77	20547940.04	21629560.32	22479167.62	27021163.89	50187699.54	35895620.13	63080043.27	39948669.14	43654472.56	62659795.02	33338833.47	31854989.06	27419751.23
Com_1945_pos	cis-7-Hexadecenoic Acid	--	C16 H30 O2	254.22434	8.157	255.23157	4350420.024	3929872.458	3652678.636	4012444.118	5126039.12	4444989.151	60683260.86	25000715.11	35337231.24	28458190.11	29411116.25	27413975.03	21454703.16	20889690.72	21362765.39
Com_1960_pos	N-[5-[(dimethylamino)sulfonyl]-2-methylphenyl]cyclohexanecarboxamide	--	C16 H24 N2 O3 S	324.15515	5.89	325.16226	31895315.95	64328914.95	40652583.38	49805276.87	77132887.96	66663069.55	28140661.34	28756491.8	43545905.88	43745537.89	36282383.06	33748238.88	55024088.49	35954603	42115912.33
Com_1961_pos	Stearoyl ethanolamide	--	C20 H41 N O2	327.31352	10.311	328.32086	26221433.37	22918228.24	16366714.45	13198519.55	51882904.42	13750372.34	23362019.34	50756946.37	23609972.36	28613624.97	17394086.59	36037534.58	28391285.69	29584387.85	27759594.65
Com_1964_pos	(2E,4E)-N-(2-methylpropyl)deca-2,4-dienamide	--	C14 H25 N O	223.19336	9.39	224.20068	54628799.08	49419199.03	42276953.8	26087676.12	43596154.05	42644150.09	52558302.94	49996685.82	46035767.98	28684190.85	36881112.84	62022171.07	43865123.91	47420709.35	43155574.53
Com_1975_pos	D-δ-Tocopherol	--	C27 H46 O2	402.34724	10.151	403.3544	60742509.58	94170309.41	86260554.08	49296833.91	57536114.95	25421934.51	950129.8537	775901.9271	668485.034	367940.001	597074.3682	411363.3978	29252247.74	25479540.54	30818161.85
Com_1976_pos	(1E)-5-hydroxy-1,7-diphenylhept-1-en-3-one	--	C19 H20 O2	302.12634	6.181	303.1337	59216148.59	58211367.88	50880051.26	57386233.6	55488253.87	58032392.07	34096664.79	33461880.43	41668581.47	57724742.85	28816101.3	33710662.77	42759661.78	45189810.4	49835756.37
Com_2024_pos	1,4-dihydroxy-1,4-dimethyl-7-(propan-2-ylidene)-decahydroazulen-6-one	--	C15 H24 O3	252.17232	5.974	253.17963	41425575.08	38696955.87	29692559.24	30832978.42	22644715.88	34985220.47	29868725.92	30141686.9	46087286.68	55856318.49	28785142.57	25450022.23	32524157.93	31945337.91	31727352.78
Com_2049_pos	All-Trans-13,14-Dihydroretinol	--	C20 H32 O	288.245	7.357	289.25232	20671991.5	17361856.85	58428435.73	73421084.67	19052793.64	19251895.75	5309579.17	4388575.863	7082732.177	15594196.95	4406209.429	3865448.424	16970864.44	16011157.58	16406884.01
Com_2058_pos	6-Acetylmorphine	6-Acetylmorphine; 6-O-Monoacetylmorphine	C19 H21 N O4	654.29361	5.03	328.15408	9066890.827	3655530.131	5620351.38	4351331.745	4533309.022	5978256.224	56418686.4	10619378.84	22262278.42	22501494.14	12933708.6	11590213.24	11451526.61	10948815.63	10454471.01
Com_2074_pos	3-Methoxy prostaglandin F1α	--	C21 H38 O6	368.25595	8.386	369.26285	2224806.548	2006462.728	2828081.181	3494025.465	2326836.312	1912817.26	50801878.83	38991866.79	23873586.87	7076428.151	31516901.37	57821307.92	21054399.54	36011338.46	21613235.34
Com_2101_pos	methyl 2,5-dimethyl-1H-pyrrole-3-carboxylate	--	C8 H11 N O2	175.0582	5.798	176.06544	84031892.88	80106222.97	54165062.01	41990207.01	66577601.94	65098347.11	24647771.01	3337689.811	3133137.052	3502847.03	11560874.73	3314629.262	31687514.34	33864150.77	31716014.37
Com_2127_pos	LSD-d3	--	C20 H22 [2]H3 N3 O	304.24038	9.933	305.24805	77152453.81	82228069.43	65500012.5	70317852.12	42805638.85	87626693.39	12524885.55	4850764.996	23236984.07	25354646.42	8366359.172	9433484.29	25626840.2	26127722.81	14833271.23
Com_2137_pos	Luteolin	Luteolin; 3',4',5,7-Tetrahydroxyflavone; 5,7,3',4'-Tetrahydroxyflavone	C15 H10 O6	286.04732	6.048	287.0545	2206485.223	2080173.749	2753168.731	1882680.066	1545082.156	1878511.277	34112018.16	45546789.77	37364658.2	27293782.67	15644870	31926485.42	10324180.3	13675945.95	14293907.07
Com_2169_pos	N-Acetyl-D-lactosamine	--	C14 H25 N O11	383.14198	1.346	384.14944	50621599.17	36452332.4	32326422.73	23843592.39	63058142.96	93285991.04	8988289.792	26998351.81	9372462.034	34722575.87	14382883.36	16320147.23	35063708.03	35014267.19	30756967.79
Com_2177_pos	p-Mentha-1,3,8-triene	--	C10 H14	134.10949	5.68	135.11678	78036149.6	63012405.84	63123734.55	49715601.56	67354422.1	59084276.24	37079771.11	23846093.26	23457499.14	51292941.98	30569315.99	22617592.81	36896790.51	33099872.97	31702741.94
Com_2215_pos	Cer-NDS (d18:0/16:0)	--	C34 H69 N O3	539.5274	10.677	540.53467	1033799.652	636276.5201	458063.6283	12195935.14	1342695.48	6895639.645	7770908.942	4331191.324	30075755.68	50253887.7	1618222.443	976630.5016	10964173.32	13331656.63	7424430.763
Com_2231_pos	Epitestosterone	--	C19 H28 O2	288.20868	6.519	289.216	78202605.1	70135793.36	31679635.83	29997922.45	40859185.88	40944729.6	9170322.247	6685152.126	[illegible]	8704435.221	24004544.06	10028197.29	21789999.58	21699384.74	20805681.26
Com_2242_pos	Indole	Indole; 2,3-Benzopyrrole	C8 H7 N	117.05812	5.067	118.0654	4686900.923	3419561.728	4406513.609	6494394.499	6169794.219	6949653.51	3605408.82	12251332.02	11404111.33	49647308.12	6802084.564	8937893.182	7496400.396	6379468.507	6585945.411
Com_2255_pos	N4-Acetylsulfamethoxazole	--	C12 H13 N3 O4 S	295.05809	1.242	296.06531	10395687.19	6096514.622	3729870.678	4120440.596	13627476.05	8403697.536	30317904.42	37782346.19	11397903.92	2957817.865	1881719.379	52574342.59	14601189.11	15858662.08	14927791.02
Com_2281_pos	2,4-dihydroxyheptadec-16-en-1-yl acetate	--	C19 H36 O4	350.24537	7.493	351.25275	9253275.19	9808288.996	5939303.265	8763388.34	7944328.879	8162295.228	27958400.6	42517413.81	22059352.34	15383156.27	36819370.65	30101472.57	20740505.47	22913730.48	14736826.93
Com_2282_pos	(2E,4E)-N-[2-(4-hydroxyphenyl)ethyl]dodeca-2,4-dienamide	--	C20 H29 N O2	315.21971	5.357	316.22693	4428042.406	1249127.847	3394063.513	65250069.54	1433444.812	2031769.087	5271513.911	109302.3524	4328816.166	2663593.352	11499490.61	117656.3435	7681947.724	7642878.695	7241800.386
Com_2307_pos	6-methoxy-2-phenyl-3,4-dihydro-2H-1-benzopyran-4-	--	C16 H14 O3	254.09398	5.641	255.10136	50521945.52	58585992.34	62043667.26	64490635.13	32306435.27	40679744.7	2840675.759	2535587.443	2601252.694	1026493.136	2364014.068	2762474.927	29284901.49	26912286.83	29706792.47
Com_2314_pos	4-[4-phenyl-3,6-dihydro-1(2H)-pyridinyl]butanoic acid hydrochloride	--	C15 H19 N O2	245.13722	1.333	246.14452	11819303.01	64173809.04	63860455.5	64275329.45	74219724.68	19362975.13	7824602.533	13471819.1	8092282.878	11507847.34	30253523.34	11314111.97	38722508.74	32823402.27	32121867.89
Com_2334_pos	(-)-Caryophyllene oxide	--	C15 H24 O	220.18258	9.382	221.18985	41708645.57	38595087.18	36289967.36	19777965.79	40033377.95	34474050.44	33409425.2	35237529.05	40511211.5	28008280.63	30760607.24	50668475.65	35104757.2	38584410.99	31804337.8
Com_2368_pos	1,7-bis(4-hydroxyphenyl)heptan-3-one	--	C19 H22 O3	298.1566	6.526	299.16397	1744290.354	2006469.678	1388710.928	1212790.525	1155855.292	1430649.926	25990615.36	17375956.33	22751943.55	46658224.2	2742304.856	10999800.47	11180945.88	12058650.42	10768809.15
Com_2381_pos	Isorhapontigenin	--	C15 H14 O4	258.08911	5.833	259.09647	34191142.7	40703328.04	31954048.58	32261060.1	34997660.45	37776701.8	24129234.51	15459972.35	25039308.69	23891418.97	47597891.81	21332455.54	27380233.06	25806934.21	24743803.16
Com_2382_pos	6-Hydroxyflavone	6-Hydroxyflavone; 6-Hydroxy-2-phenyl-4-	C15 H10 O3	238.06285	5.818	239.07016	9765703.996	11262886.05	9906217.434	9348151.616	11369125.13	12131088.16	33747690.02	29370912.31	36070242.84	46277871.05	34486623.22	32135086.51	19538998.34	19061351.38	20508546.32
Com_2385_pos	alpha-Farnesene	alpha-Farnesene; (3E,6E)-alpha-Farnesene	C15 H24	204.18765	7.732	205.19493	1804679.188	1914513.246	1387378.558	2013379.694	1789749.776	1829293.313	19519429.78	11906853.61	22129798.64	46207270.18	6752285.241	20711242.15	14251763.89	12415615.25	14143982.06
Com_2397_pos	N-{4-[(2R,3R)-3-(Hydroxymethyl)-5-oxo-2-morpholinyl]phenyl}acetamide	--	C13 H16 N2 O4	246.1004	5.002	247.10765	40224384.4	81250062.48	49536661.11	29147918.66	49158714.23	80569142.6	1389376.7	1171720.935	1470629.988	1728324.266	1548360.648	1463651.399	32312734.33	16995100.53	29692887.36
Com_2401_pos	3-hydroxy-3-(2-pyridylmethyl)indolin-2-one	--	C14 H12 N2 O2	240.08998	5.191	241.09726	2993138.949	2601830.593	2664491.25	3143422.521	3265054.932	3002162.461	440189.1599	504782.5959	355507.2634	445642.5709	47195126.25	656270.2241	4374802.432	3581448.105	3820133.357
Com_2403_pos	Cynaropicrin	Cynaropicrin	C19 H22 O6	368.12562	7.53	369.133	551432.8918	603082.4397	537815.6238	686738.3949	725230.2944	507851.0163	12159994.14	39848494.7	24640793.72	34679932.14	5799748.37	31072625.49	15120706.37	14113459.74	15319729.53
Com_2417_pos	Capryloylglycine	--	C10 H19 N O3	201.13622	1.362	202.14351	4746664.619	6175951.328	6109700.526	5982561.51	8963698.477	6859732.136	2263566.946	1951834.112	2461367.073	2504357.955	46657265.85	3333196.933	8598625.538	8690558.246	7848293.501
Com_2418_pos	trans-Cinnamaldehyde	--	C9 H8 O	132.05747	6.02	133.06471	11369776.9	20142934.97	9643002.37	9040029.075	18879632.7	17250044.3	20877450.85	24692298.93	27885591.39	45345058.43	40172462.07	23012215.09	21781138.62	22028669.5	21319076.81
Com_2461_pos	15-Deoxy-Δ12,14-prostaglandin J2-2-glycerol	--	C23 H34 O5	390.23784	8.385	391.24518	652748.0324	725950.7319	543460.9513	705232.8518	697358.323	835811.4843	38153479.02	32447570.25	20503264.09	2119035.112	27251908.95	47535238.41	15982533	15635212.72	16874873.4
Com_2476_pos	2-(3,5-dimethyl-1H-pyrazol-4-yl)-5-methoxybenzoic acid	--	C13 H14 N2 O3	246.10038	5.118	247.10765	52970843.99	54916206.98	34496729.21	40912668.58	71108588.58	62454464.54	11427797.14	4388597.24	1763756.555	7199172.323	1808749.828	5679912.064	22571207.19	22426760.88	21827841.78
Com_2504_pos	Betulin	Betulin	C30 H50 O2	442.38113	10.672	443.38794	1928068.837	2232438.004	2383912.331	2244335.492	3965322.374	1544355.132	44875993.73	32381173.65	14782469.49	15577130.61	18746131.39	25943962.51	10602134.48	11252084.38	10759399.6
Com_2521_pos	3-amino-2-phenyl-2H-pyrazolo[4,3-c]pyridine-4,6-	--	C12 H10 N4 O2	242.08031	5.916	243.08751	4653082.365	3615357.313	4058604.378	8724185.089	5436678.123	6494619.767	44646849.78	3561309.774	3088824.673	10166601.08	23312574.06	6890412.422	11715861.31	7640134.959	9425857.016
Com_2528_pos	Ergosterol peroxide	--	C28 H44 O3	428.32903	10.719	429.33655	10745258.81	7255812.438	7561803.707	5014460.729	12927582.97	4545199.212	27627313.68	20452122.79	23701169.02	12273198.04	44424627.62	35366413.65	31989703.62	23167866.05	20434963.13
Com_2530_pos	3-(1,3-benzoxazol-2-ylthio)-5,5-dimethylcyclohex-2-en-1-	--	C15 H15 N O2 S	273.08451	1.492	274.09186	11613064.88	12042865.88	34064034.18	16788138.6	37049580.07	48617631.57	2361022.954	11807618.86	4339304.691	43190896.47	19867917.02	4744069.429	22284617.04	16268498.1	18938342.94
Com_2571_pos	3,4,5-trihydroxy-6-methyloxan-2-yl 2-	--	C14 H19 N O6	319.1049	5.734	320.11221	62895131.28	55592000.8	52682008.14	56008156.39	68071910.93	67717338.45	9988600.495	4430815.307	6602234.666	4180532.475	11161051.58	7587901.268	29244451.6	30190176.4	30337270.52
Com_2581_pos	2'-Deoxycytidine	Deoxycytidine; 2'-Deoxycytidine	C9 H13 N3 O4	227.09015	1.348	228.09749	6836294.081	9904458.544	13617542.82	7052457.427	10384628.4	20461739.2	6958628.106	11261605.17	40796923.27	12275106.49	4227759.089	44978382.95	11238853.02	9108760.548	10040842.95
Com_2591_pos	Heptadecanoic Acid	--	C17 H34 O2	292.23989	7.821	293.2471	4945236.088	4326665.67	4587844.378	6251632.109	4599280.456	4506421.234	43330344.13	36311455.74	27886117.09	36761240.27	15379310.57	23369592.14	25195715.83	22119217.13	23479045.24
Com_2596_pos	Fmoc-L-Isoleucine	--	C21 H23 N O4	353.1626	5.37	354.16983	1989356.321	1874976.389	1968752.782	1803417.425	1740870.617	1920641.843	43148062.71	11508901.53	36426230.36	23126530.11	11833248.67	12023985.96	11585130.2	10939467.48	10997241.39
Com_2636_pos	2-hydroxy-6-[(8Z,11Z)-pentadeca-8,11,14-trien-1-yl]benzoic acid	--	C22 H30 O3	359.24585	5.041	360.25323	203660.4059	214632.0245	183002.6166	188533.1308	218553.1681	230797.4209	15756597.78	763786.8755	12445822.77	12180708.78	42174531.54	840445.8167	4776777.773	5056541.373	5765574.884
Com_2637_pos	1-[2-(2,5-dimethyl-1H-pyrrol-1-yl)-4-nitrophenyl]-1H-	--	C15 H14 N4 O2	282.10799	5.502	283.11542	50354959.3	55087103.74	33797386.92	49606828.59	49883852.6	54981140.21	42233673.96	32001027.31	37177543.84	35279145.47	37996192.3	33798399.22	34358831.08	38426961.1	36732062.77
Com_2643_pos	4-oxododecanedioic acid	--	C12 H20 O5	266.11296	5.914	267.12009	25487166.47	30728833.51	42994888.99	39790712.59	40228809.58	47197693.11	37864834.05	30176373.69	34049578.09	26090784.65	30592301.92	43547076.38	31514655.41	31468293.52	35433045.9
Com_2654_pos	15-Deoxy-Δ12,14-prostaglandin A1	--	C20 H30 O3	300.2087	8.257	301.21597	12031982.3	15050784.02	19500766.76	20234037.34	11300102.56	12165649.41	33022459.6	25674578.96	31859006.13	40723466.95	26288102.92	25314322.92	20865328.9	21453925.27	20637692.71
Com_2667_pos	TMK	--	C15 H30 N4 O5 S	189.09717	1.356	190.10451	63586210.91	21551867.47	40074358.49	34704383.15	29105142.99	53515557.84	8581317.741	7150518.257	9440189.023	12946323.35	15630201.32	12297807.79	24963935.14	23267544.99	23284649.49
Com_2680_pos	L-Tyrosine	L-Tyrosine; (S)-3-(p-Hydroxyphenyl)alanine; (S)-2-Amino-3-(p-hydroxyphenyl)propionic acid; Tyrosine	C9 H11 N O3	181.07125	5.926	182.07855	38327353.54	58312345.23	59992397.56	38674622.26	56950792.79	65504844.42	31380397.13	13723054.94	19396423.42	16739003	13261560.13	20834696.91	36290943.25	32736186.98	39575093.74
Com_2682_pos	2-Phenylethylamine	Phenethylamine; 2-Phenylethylamine; beta-Phenylethylamine; Phenylethylamine	C8 H11 N	121.08906	4.789	122.09634	22830310.76	42940936.1	26411917.3	17624466.83	63296998.95	72691572.97	3322111.674	8763630.222	7064299.188	7530932.715	2336962.262	12091643.84	25396098.73	21340981.93	20621995.93
Com_2685_pos	N-Acetylsphingosine	--	C20 H39 N O3	363.2742	8.48	364.2814	2012356.336	2143289.846	1621070.722	1745296.013	4395081.71	2127433.261	41311007.84	29792834.4	21795110.85	2210120.221	21816939.24	14814283.84	7303069.334	8311618.219	8557640.302
Com_2687_pos	methyl 3,3,3-trifluoro-2-hydroxy-2-[(2-phenoxyacetyl)amino]propanoate	--	C12 H12 F3 N O5	307.06919	5.122	308.07654	55263968.11	62994560.77	59877026.54	27227915.73	51085484.49	51240253.54	1293091.752	1078125.494	846288.4347	1149927.485	1783290.594	1476775.188	26113331.71	24945559.11	23650937.97
Com_2735_pos	3,5,7-trihydroxy-2-phenyl-4H-chromen-4-one	--	C15 H10 O5	270.0526	6.275	271.05978	1164958.054	1375418.096	1365922.147	1075885.479	1522424.83	993213.1599	15751617.58	15935794.21	24446532.9	39275466.28	30745974.56	15561482.55	10509662.94	10957084.79	10657017.45
Com_2775_pos	Boldione	--	C19 H24 O2	284.17732	6.346	285.1846	8117473.498	7838453.093	6470408.838	6320555.156	6934529.569	8265356.755	24875364.4	3006523.099	11790222.48	18125758.27	39791124.41	3719039.886	10337244.85	11076368.07	10262498.51
Com_2784_pos	Etiocholanolone	Etiocholanolone; 3alpha-Hydroxy-5beta-androstan-17-one; Etiocholan-3alpha-ol-17-one; 3alpha-Hydroxyetiocholan-17-one	C19 H30 O2	272.21367	8.899	273.22089	5107614.425	5652741.445	5292863.161	5718066.825	6160751.616	5261304.468	14365967.9	13599469.65	24873245.1	38507147.31	9319350.001	16638728.52	13186808.98	12663320.4	12392875.23
Com_2795_pos	3-Acetyl-11-keto-β-boswellic acid	--	C32 H48 O5	512.34755	8.593	513.35486	18946751.43	20240875.45	10594316.52	11905770.93	29498207.76	36382027.19	39431115.75	17227593.49	21902255.17	34450359.21	13541762.08	14056691.53	17903856.28	19192617.44	17919563.33
Com_2801_pos	2-(piperidinomethylidene)malononitrile	--	C9 H11 N3	161.09571	1.199	162.10304	25591731.5	6727673.949	16030380.46	50899818.83	15449033.03	18243202.79	2909795.794	2855193.229	2854572.812	6450003.144	5681125.713	3359663.502	13049939.15	10190796.47	9925570.695
Com_2875_pos	Valylproline	--	C10 H18 N2 O3	214.13159	1.372	215.13869	13413978.77	15424456.1	11037380.5	15769429.36	17240075.16	15752830.84	5853095.707	8658665.299	13095211.01	16282166.34	37917487.28	11276245.23	13864849.91	12986240.95	14088186.08
Com_2891_pos	Tetranor-12R-HETE	--	C16 H26 O3	248.17735	9.728	249.18463	2478085.196	2332522.442	1559456.717	1343907.072	3481146.016	3739978.769	37772262.27	22487338.88	13191774.5	12299031.68	9835762.911	17271508.22	13662675.66	14579973.76	15083402.97
Com_2899_pos	Orlistat	--	C29 H53 N O5	533.343	9.886	534.35028	1439316.944	1196499.63	1170728.975	770978.5999	1258650.371	1022873.705	4385112.597	7413703.122	10596242.37	36563570.88	13642407.6	5384310.828	6043407.392	3912045.359	5014276.091
Com_2985_pos	Lagochilin	--	C20 H36 O5	356.25594	6.456	357.26312	55227983.47	14339085.89	30464490.34	14913910.93	16118155.16	13224641.13	9684739.011	6788388.935	11612640.71	18770600.85	8973488.722	7463176.563	12480452.4	14069765.22	13662853.23
Com_3003_pos	2-[(4-nitrobenzyl)thio]pyridine	--	C12 H10 N2 O2 S	246.05043	5.374	247.05775	4848692.312	3414483.739	4342203.319	5639532.934	5231928.363	4615198.437	17965506.91	30061986.62	30185394.06	22533051.99	26078929.81	37175265.54	10079482.76	10065618.44	8279054.058
Com_3006_pos	1-(7-methoxy-2-oxo-2H-chromen-8-yl)-3-methyl-2-oxobutyl acetate	--	C17 H18 O6	340.09436	5.317	341.10175	54773426.85	30364531.49	31704465.1	21845102.39	2909681.397	32505786.58	4710863.653	2759669.087	3018605.861	2343432.219	2024901.933	2777613.984	17980297.76	17800597.95	22920359.11
Com_3047_pos	4-Ethylbenzaldehyde	--	C9 H10 O	134.0732	5.686	135.08037	34995460	39615603.73	36475037.58	33850671.98	35702289.41	34861545.53	23273770.32	25747558.74	28572865.07	34282902.44	27734676.53	20476420.58	30906903.09	15677682.22	26348441.36
Com_3061_pos	7-methylpyrimido[4,5-d]pyrimidin-4-amine	--	C7 H7 N5	183.04909	1.277	184.05582	9258584.273	12507930.87	17587188.84	20432545.64	23486921.88	21412370.53	1474097.777	18324981.48	8293120.218	34106908.01	2908556.447	12620191.68	14354443.54	13506871.39	14257485.89
Com_3071_pos	Amitriptyline-d3	--	C20 H20 [2]H3 N	280.20136	6.412	281.20862	467100.4995	458508.3501	432647.4191	402075.8911	415562.0115	447386.5457	282051.0676	5802317.686	11066913.33	10986392.81	34948353.97	7385755.289	5458019.533	4975334.526	5022831.588
Com_3078_pos	Lysine Butyrate	--	C10 H22 N2 O4	234.15735	1.267	235.16382	11077321.62	376509.0563	12307990.83	32417435.01	11046866.71	29093515.97	3343754.003	29523894.57	4288144.335	1402275.607	4355767.651	14561881.44	13242479.98	12425645.41	14497688.17
Com_3090_pos	HKK	--	C18 H33 N7 O4	393.25136	4.873	394.25864	163548.7494	173658.1919	154954.3067	148415.6389	154592.4984	150927.7802	1994163.581	1411797.803	8928528.044	16407027.9	34808143.81	1354901.784	6027644.599	5822501.494	6062208.946
Com_3097_pos	N-(9-oxodecyl)acetamide	--	C12 H23 N O2	213.17286	5.268	214.18015	6698903.902	7371500.405	6828618.833	6791802.979	7547981.767	6830369.276	5099787.203	721646.2749	1304935.52	1141703.896	34657903.79	977122.4056	6723475.853	6109825.776	6547384.685
Com_3111_pos	Corticosterone	Corticosterone; 11beta,21-Dihydroxy-4-pregnene-3,20-dione; Kendall's compound B; Reichstein's substance H	C21 H30 O4	346.21216	7.415	347.21906	8390376.486	9237412.028	10097964.17	7090665.539	8658866.204	7744223.765	8654326.272	29212283.39	15207772.93	13034302.82	7749289.863	10580764.25	8807490.241	11348243.27	9473486.827
Com_3113_pos	11-Deoxy prostaglandin F1β	--	C20 H36 O4	322.25063	7.384	323.2579	19705048.26	16796072.27	16153503.47	17183883.71	14128831.29	15273851.07	15415681.78	17595780.49	22778760.62	33557083.62	14221481.71	19955835.51	16374943.32	16573269.41	17018282.36
Com_3171_pos	ethyl 5-hydroxy-4-oxo-4H-chromene-2-carboxylate	--	C12 H10 O5	234.05264	6.391	235.06001	1054571.548	1015028.21	757322.5492	784002.3242	967468.3816	983896.7821	4901133.787	17148112.08	20767083.75	17343079.55	4115869.302	34913909.31	7113642.632	7234360.239	7500868.516
Com_3178_pos	N6,N6,N6-Trimethyl-L-lysine	N6,N6,N6-Trimethyl-L-lysine	C9 H20 N2 O2	188.15349	8.749	189.16077	12754896.48	14510043.83	12549044.12	11737358.63	16008342.22	17160994.44	28794742.43	28480700.81	30321777.1	29351776.76	19406822.36	30608244.91	23041304.83	25142551.72	23117027.39
Com_3206_pos	Isoquinoline	Isoquinoline; 2-Benzazine; Benzo[c]pyridine	C9 H7 N	129.05794	4.782	130.06526	2466279.672	3031857.406	2386613.488	2198147.238	2463170.889	2799360.68	2214396.134	4063477.164	22267425.69	32323401.59	6347819.073	6044085.849	6384943.041	5257567.24	5432472.628
Com_3225_pos	Flunitrazepam-d3	--	C16 H9 [2]H3 F N3 O3	316.10563	6.675	317.11285	10109330.38	11858223.06	10552278.84	12426562.67	10663234.61	9708368.507	17327587.45	20832083.21	25200895.09	32122240.56	31979966.63	23733241.22	17764736.16	17662646.52	15155606.87
Com_3360_pos	N-[1-(4-methoxy-2-oxo-2H-pyran-6-yl)-2-methylbutyl]acetamide	--	C13 H19 N O4	270.1579	4.917	271.16501	46293223.95	47010358.34	39218013.1	40735985.31	35560092.22	36653476.86	1675205.36	2332507.374	1130283.371	2023130.316	715933.793	2891440.497	19981832.2	17725813.28	15442272.71
Com_3368_pos	1,4-dihydroxyheptadec-16-en-2-yl acetate	--	C19 H36 O4	310.24849	9.575	311.25577	10773515.47	12275694.51	9850316.882	5668296.684	10864622.37	10107977.61	17505229.61	22449866.79	23454122.46	27938467.65	31233741.22	16937830.42	15837974.16	16220894.93	17243158.22

Com_3369_pos	Homo-Gamma-Linolenic Acid (C20:3)	--	C20 H34 O2	306.25537	7.973	307.26266	10629926.45	12163330.26	12323476.33	15027107.3	8363323.639	8764859.872	20098050.19	26406794.69	18785779.33	24355722.34	25185020.19	25028467.72	15081224.67	14851154.15	16514246.25
Com_3407_pos	KKK	--	C18 H36 N6 O4	362.30274	6.043	363.31012	2628329.843	2121142.772	1858333.365	2112151.78	1915847.282	2015263.681	2398653.295	5795817.906	17313375.99	29985651.04	4798257.398	3897995.435	3765227.88	4411813.414	3653770.356
Com_3419_pos	Acetyl-L-carnitine	--	C9 H17 N O4	203.11549	1.348	204.12274	17103825.97	17414977.98	18396282.21	15559678.94	19961864.86	17256607.42	8415418.482	16065578.07	21566538.66	29825542.9	1167331.277	24163414.95	15036813.32	16171630.92	14455797.12
Com_3458_pos	2-(3,4-dihydroxyphenyl)acetamide	--	C8 H9 N O3	189.03993	5.079	190.04723	3109039.869	4365985.314	4063584.715	3820419.128	4142861.359	4802422.807	2240355.772	2594500.88	26030753.32	4829736.672	2230217.473	28888011.88	7681397.887	8005676.239	5832945.615
Com_3478_pos	(2E)-6-hydroxy-2-methyl-6-(4-methylphenyl)hept-2-enoic	--	C15 H20 O3	230.1263	1.355	231.13358	4668796.388	5853687.81	4294716.581	4974367.586	7268380.211	5993893.414	2981160.629	3314035.869	5453266.365	5758480.159	30040003.36	4962138.133	7273143.819	7690544.004	7701737.212
Com_3525_pos	5-trans prostaglandin F2β	--	C20 H34 O5	376.2232	6.311	377.23071	40226298.56	39403805.08	34815013.55	38442549.01	30059360.27	37807768.88	20273861.7	11002320.04	15847260.17	23849516.87	11648991.15	10054610.13	24370594.97	24597980.42	25188682.88
Com_3535_pos	3-Acetoxyurs-12-en-23-oic	--	C32 H50 O4	498.36839	9.521	499.37567	8135130.023	9037791.471	6348404.073	8543522.807	15255514.75	10209172.39	11476782.29	9547541.081	15962338.05	28673781.36	10509756.87	9755191.639	10653266.57	10935406.27	10721553.04
Com_3546_pos	L-Glutathione (reduced)	--	C10 H17 N3 O6 S	307.08413	6.399	308.09143	766152.4707	379039.5183	575551.8129	382847.4995	659183.4791	998310.5043	7183042.28	18142022.16	21844803.94	28612354.62	6113866.0196	18970064.05	8387712.279	8648284.844	8232330.854
Com_3554_pos	(+/-)11(12)-DiHET	--	C20 H34 O4	360.22745	7.176	361.23456	9996365.552	9706206.115	27305945.72	6540385.953	8139314.797	7502707.878	22903750.65	9016102.926	20471896.72	28548935.8	6606002.072	9748594.331	9196441.436	10069991.87	8808406.613
Com_3572_pos	Ocfentanil-d5	--	C22 H22 [2]H5 F N2 O2	375.24076	4.818	376.24808	141031.2599	136969.0304	119808.5739	114820.2366	143848.1945	148660.9286	1318258.377	1131237.656	328704.1279	453509.804	29104128.32	1202625.897	5017457.001	4304848.025	3934801.092
Com_3578_pos	N6-Acetyl-L-lysine	N6-Acetyl-L-lysine	C8 H16 N2 O3	188.11725	7.88	189.12447	20056609.67	21546797.12	32242809.58	37693742.19	24246002.75	17415696.85	7819776.413	6170192.979	9561400.037	12670391.43	8937965.323	6729916.774	13424972.27	14756878.28	17715399.18
Com_3597_pos	S-Adenosylhomocysteine	S-Adenosyl-L-homocysteine; S-Adenosylhomocysteine	C14 H20 N6 O5 S	384.11928	5.778	385.12656	30224646.19	9158913.147	33426787.22	37438397.98	31539218.38	21461962.41	3252897.339	2527607.588	2443451.605	2765240.479	2074402.599	2991505.07	5998468.528	4474315.004	5262938.312
Com_3617_pos	4-(4-hydroxy-3,5-dimethylphenyl)-2-methylphthalazin-1(2H)-one	--	C17 H16 N2 O2	280.12114	5.174	281.12842	7167861.504	7847960.366	5995603.127	6490316.826	5566695.96	6538690.872	4836592.494	1875438.712	2108471.762	2929356.878	28688173.67	2791945.809	5493689.139	4619997.809	4888297.465
Com_3630_pos	2-Arachidonoyl glycerol	--	C23 H38 O4	378.27441	7.063	379.28165	3430577.956	3847948.869	4771210.699	4927468.182	2823640.673	2546809.588	2934187.77	2858755.054	10465363.17	27809216.29	3704997.778	2707318.727	5919871.324	4944470.404	4871799.088
Com_3636_pos	5α-Pregnan-3,20-dione	--	C21 H32 O2	316.23994	9.134	317.24724	42428064.09	33211460.11	9322448.694	9618679.519	9659541.251	10104406.08	5353490.995	10406930.79	13305726.86	27756594.24	2768733.709	12496025.02	10875130.03	11435622.3	10865622.08
Com_3673_pos	Linoleladic Acid (C18:2N6T)	--	C18 H32 O2	280.24158	9.516	281.2482	8966712.887	6989578.093	8611618.137	7208887.701	5145203.092	6228908.852	12483853.97	23218129.49	20424380.48	27296620.31	1710177.96	22264590.81	18939525.23	14209257.24	16018647.72
Com_3694_pos	Tetranor-12(S)-HETE	--	C16 H26 O3	288.1684	4.785	289.17578	42460317.62	36946353.51	10970114.86	15513644.94	30396313.43	22050071.32	2855477.178	2260369.193	2318297.85	4913599.994	2089768.764	3253453.811	10742018.44	8494661.78	9081185.426
Com_3716_pos	Coenzyme Q2	--	C19 H26 O4	318.18679	10.023	319.194	18385434.41	21213920.51	15743825.12	10279764.07	18725721.46	16449376.16	25899523.07	23276629.95	26006691.87	18883404.11	18304856.18	21813343.57	21821055.12	20952613.71	22003850.45
Com_3719_pos	1-(3,4-dimethoxyphenyl)ethan-1-one	--	C10 H13 N O3	195.08927	2.18	196.09653	19345402.34	25339739.73	18164139.85	19205323.43	42937924.88	30904426.26	2709676.753	1355577.649	1607933.012	1940877.479	1204657.931	1682177.215	6961088.778	6012847.299	5724634.331
Com_3796_pos	3,5-di(2-furylmethylidene)tetrahydro-2H-pyran-4-one	--	C15 H12 O4	256.07323	5.534	257.08051	28683751.98	39196821.46	36471670.23	34614077.19	36543501.01	43983451.75	9614422.982	5931377.123	6771947.635	6803495.007	3907903.326	6377588.218	12691714.76	20911324.82	18774295.89
Com_3876_pos	4-(benzoylamino)-3-hydroxybutanoic acid	--	C11 H13 N O4	223.08445	5.249	224.09184	14765547.43	13643655.08	20512750.92	25993231.9	8544961.92	12734626.47	16567722.18	21889029.14	24802699.18	16368607.68	19942392.37	21881932.32	14477665.99	16217617.61	18796226.39
Com_3900_pos	16α-Hydroxydehydroepiandrosterone	16alpha-Hydroxydehydroepiandrosterone; 5-Androstene-3beta,16alpha-diol-17-one	C19 H28 O3	321.23586	8.542	322.24313	38545083.86	38517650.46	30652982.4	29710264.78	40117611.12	40151517.18	13109583.51	7498538.727	15695956.37	11450127.21	15041006.99	8837057.144	18277991.04	19505727.87	18139313.24
Com_3902_pos	N1-(2-amino-2-oxoethyl)-2-(isopropylthio)acetamide	--	C7 H14 N2 O2 S	212.05596	5.784	213.06329	1194115.9	1028394.551	790170.0537	652417.2907	1175134.774	998933.9048	13430583.47	17785473.3	28084862.58	21746928.2	21334451.03	21372624.42	10725530.74	10637379.39	10786012.34
Com_3924_pos	3-(dimethylamino)-1-[4-(phenylsulfonyl)phenyl]prop-2-en-1-one	--	C17 H17 N O3 S	315.09264	2.482	316.09988	12196645.17	14593678.08	1119223.477	5022474.817	22017887.18	10808714.6	6571276.746	19610852.03	8654975.73	24809324.72	2962107.769	23216513.31	10862496.66	11513719.18	10364484.83
Com_3938_pos	1-(4-hydroxyphenyl)propane-1,2-diol	--	C9 H12 O3	190.06016	5.665	191.06741	38009349.44	38692516.97	32078528.39	33006047.85	23378667.96	34811036.08	9458682.584	8149049.39	9799918.79	9872547.343	7477113.739	9554631.059	15961626.22	18303896.32	19318547.35
Com_4046_pos	5-Henicosyl-1,3-benzenediol	--	C27 H48 O2	404.36434	11.337	405.37195	5689696.649	6203024.209	5654134.048	3596910.617	9772508.857	3224129.604	24327564.18	12142957.59	9376761.364	7548084.203	4789861.715	20116101.46	12570896.22	12304270.37	13219868.57
Com_4077_pos	6-amino-4-(3,4-dimethoxyphenyl)-2-thioxo-2H-thiine-5-carbonitrile	--	C14 H12 N2 O2 S2	168.0326	5.185	169.03976	3003694.645	2421761.27	3780625.035	4246669.405	3268930.458	3689635.161	24031797.27	19926388.26	20804981.88	14203623.83	23768544.02	20988074.68	6216216.022	6213013.36	6055960.321
Com_4193_pos	N'-hydroxy-4-(trifluoromethoxy)benzenecarboximidamide	--	C8 H7 F3 N2 O2	220.04837	5.297	221.05559	35192646.32	35678674	27908056.42	28035563.94	28356836.14	31918517.53	10953958.78	6786910.088	9611911.258	9125650.036	10146798.83	9231621.295	18735578.46	17611985.07	16899159.69
Com_4228_pos	ethyl 1-methylpyrrolo[1,2-a]pyrazine-7-carboxylate	--	C11 H12 N2 O2	204.08994	4.884	205.09721	1299196.974	1487300.161	1199742.209	1208892.19	1339783.169	1466468.534	1116468.354	389959.1808	1098799.806	1027535.609	22881532.86	464189.2272	2249728.7	2450175.903	2403278.383
Com_4248_pos	Erucic acid	Erucic acid; (13Z)-Docosenoic acid; cis-13-	C22 H42 O2	338.31796	10.402	339.32492	6508631.973	2883469.893	5872437.089	5083451.507	5693963.671	2698136.339	20587000.61	19230466.18	6881584.359	3290319.813	11589408.31	16014532.41	11619227.27	11390265.69	11219085.61
Com_4256_pos	RNK	--	C16 H32 N8 O5	208.1249	7.167	209.13223	1481828.088	1536461.414	1411152.066	2535102.64	1483108.424	1358184.245	12512928.39	10913570.43	6405228.709	4628699.173	22712529.15	10270784.12	7455986.619	7600017.844	6809853.282
Com_4262_pos	2-[[(4,5-dimethoxy-2-nitrophenethyl)imino]methyl]phenol	--	C17 H18 N2 O5	330.12118	5.753	331.12851	6958941.254	7556769.693	7614837.507	6059972.454	7930708.052	7221727.436	11109265.88	12899660.41	17181535.32	22050730.78	14710483.55	14479410.47	10003826.73	11581858.06	9319982.326
Com_4281_pos	L-Dopa	3,4-Dihydroxy-L-phenylalanine; L-Dopa; 3-Hydroxy-L-tyrosine; L-beta-(3,4-Dihydroxyphenyl)alanine; Levodopa; Dihydroxy-L-	C9 H11 N O4	197.0689	5.162	198.07617	25006614.49	27871120.99	32689900.71	28865984.15	11801188.21	13168935.07	5475404.06	5230485.726	4917028.422	6136217.529	4461368.433	6409693.049	12637431.95	11107551.12	10974557.91
Com_4320_pos	LDGTS 15:0	--	C25 H49 N O6	459.35582	9.001	460.3631	10123963.5	11022442.95	7987278.285	9209517.842	18018494.32	15624332.86	5248340.813	18787602.04	7260361.034	12046193.62	3270953.914	16718810.92	8974262.647	9456475.229	8558080.732
Com_4364_pos	Artemisinin	Qing Hau Sau; Artemisinin	C15 H22 O5	586.2781	6.154	587.28577	8901464.55	13108739.82	25605332.62	28491550.03	18278679.67	21661325.79	1713777.699	1491904.2	453545.7975	3547141.705	1234774.359	844689.3049	8734174.838	9061806.299	8447341.122
Com_4368_pos	8-(1,2-dihydroxy-3-methylbut-3-en-1-yl)-7-methoxy-2H-chromen-2-one	--	C15 H16 O5	293.12616	4.753	294.13345	3689185.089	3988001.189	3501367.416	3891633.244	3479753.72	3124968.475	8329337.711	15972180.41	8979323.489	19197731.56	1451258.089	22655989.38	12239618.36	9948405.574	8522186.024
Com_4443_pos	(6E,10E)-3,7,11,15-tetramethylhexadeca-1,6,10,14-tetraene-3,5,9-triol	--	C20 H34 O3	339.27685	6.153	340.28412	919241.3028	963321.0231	808116.0694	769485.4843	725174.8827	953949.4996	9326300.837	18066032.35	14598204.91	4908771.748	12814267.16	16370332.94	6270193.274	5944292.07	6269520.06
Com_4455_pos	Kahweol	--	C20 H26 O3	314.18789	6.613	315.19513	32479163.03	28603190.56	25073104.79	19337560.05	28509135.35	30741348.29	11619521.16	6877083.668	10297739.12	10675270.22	10745836.7	9788021.747	15503747.87	15077581.42	17279638.58
Com_4464_pos	Sinomenine	Sinomenine	C19 H23 N O4	329.15837	4.802	330.16565	6275328.966	5474394.147	4147524.163	4723086.572	6070067.28	5448884.982	668296.3767	1983168.818	1459030.782	20669459.16	754707.3856	1518954.933	4404794.993	4645951.303	4059199.063
Com_4483_pos	methyl 2-mercapto-1-methyl-1H-imidazole-5-carboxylate	--	C6 H8 N2 O2 S	172.0296	1.306	173.03699	6795992.03	8915421.675	2267890.225	4803123.927	4744324.009	5978519.523	2349739.38	9392918.187	10030884.23	20584552.68	3247506.079	10845554.81	6202415.335	5362522.878	7464807.325
Com_4487_pos	3-(2-chlorophenyl)-3-(2,4-difluoroanilino)acrylonitrile	--	C15 H9 Cl F2 N2	290.04117	1.462	291.04849	1320964.008	1684357.837	1063078.272	841774.6231	2750450.731	1710238.943	1568802.948	11848460.54	7419061.995	672028.2501	1872321.773	21825663.07	4482226.676	3856981.929	3259432.162
Com_4523_pos	N-Acetyldopamine	--	C10 H13 N O3	195.08943	4.799	196.097	26045841.74	35938421.55	24772410.76	21474526.95	31389554.28	26136486.25	1523794.224	1527782.359	1607144.287	2670297.511	1694769.862	1676318.04	10902431.75	10495451.79	9799384.134
Com_4583_pos	Sodium cholate	--	C24 H39 Na O5	430.26959	7.685	431.27704	23943542.44	24325536.99	24006863.03	21244431.95	14943087.89	20401123.63	5802775.871	10374681.26	14246485.54	19878728.12	14861354.14	8405975.637	16207157.98	15157133.76	15276140.54
Com_4638_pos	Carbaprostacyclin	--	C21 H34 O4	332.23475	8.799	333.24197	22807958.9	23119106.09	21453280.82	26234114.82	17105918.99	20021637.25	10805645.22	8716528.842	9153143.463	5892758.773	8065452.57	10137263.06	13146989.18	13165703.62	13765846.28
Com_4639_pos	α-Lapachone	--	C15 H14 O3	264.07849	5.334	265.08575	5359364.441	20456901.82	29346833.43	18093062.53	21909729.76	19950806.06	4062723.399	2263465.185	2731174.695	2134500.924	1721168.419	2664065.34	14985381.12	9804651.842	13280396.14
Com_4678_pos	Quinoline	Quinoline; Benzo[b]pyridine	C9 H7 N	129.05789	5.353	130.06519	30433600.17	27614815	18544363.11	13431375.66	22251471.49	7362771.399	553414.7895	702492.5626	771804.5418	1254362.373	654931.6272	583114.1139	8096376.622	6302356.84	6555934.261
Com_4714_pos	Tolterodine	Tolterodine	C22 H31 N O	325.24035	5.588	326.24762	17533065.57	20104852.56	14508530.41	14985866.45	15305841.41	17520075.28	4230000.644	1486503.298	4853864.601	4907957.643	19744919.25	1697496.675	11117429.7	11035642.73	11117369.8
Com_4740_pos	N-[4-(diethylamino)phenyl]-N'-phenylurea	--	C17 H21 N3 O	321.12401	1.366	322.1312	5776037.597	7688075.765	8260568.48	8569696.564	8807666.537	8447221.698	9180551.767	11560554.46	12192734.61	11324749.99	2890517.837	20215377.5	6827791.993	8289128.727	9930790.352
Com_4751_pos	DL-Tryptophan	--	C11 H12 N2 O2	226.07426	5.241	227.08154	25882684.28	20790229.52	18553490.72	25261666.37	25090900.66	25598880.26	1945794.9	1406800.882	1623271.162	1765937.186	1358603.853	1686582.926	13103104.56	11934711.92	9777457.432
Com_4811_pos	T-2 Triol	--	C20 H30 O7	364.18641	5.8	365.19373	27087771.12	19598512.22	20524859.7	22980076.55	21527810.86	25422967.97	15565023.37	12093334.87	16178818.36	18663605.06	12835480.49	11898995.92	16936014.65	15484448.65	15681342.8
Com_4821_pos	THC	Dronabinol; Delta9-Tetrahydrocannabinol; Tetrahydrocannabinol; THC	C21 H30 O2	314.22428	10.103	315.23154	789207.198	624072.7588	569580.3253	878439.8702	601100.7582	675931.0812	16378959.35	16188522.04	10602414.98	12529215.42	3755255.53	12444449.65	4937993.451	8124449.742	8244089.839
Com_4859_pos	(2R,3S,4S,5R,6R)-2-(hydroxymethyl)-6-(propan-2-yloxy)oxane-3,4,5-triol	--	C9 H18 O6	260.06793	5.348	261.07434	20627316	24719243.17	27545728.59	19677106.06	22499238.55	26962979.04	3987538.747	3363802.702	4357229.38	3953207.551	3252603.825	4146092.011	14382424.87	12790541.72	11514043.23
Com_4868_pos	6-(3-hydroxybutan-2-yl)-5-(hydroxymethyl)-4-methoxy-2H-pyran-2-one	--	C11 H16 O5	250.08396	6.312	251.09137	2379965.757	2943246.601	2553162.475	2584052.93	2284058.833	2769374.406	6677743.664	12381090.53	14849594.72	17038998.58	4640845.68	19549595.04	7240966.353	8401805	7548759.762
Com_4875_pos	6,6-dimethyl-4-piperidino-5,6-dihydro-2H-thiine-2-thione	--	C12 H19 N S2	241.09459	1.358	242.10179	18499496.34	32454462	23360832.14	23565466.08	27041583.6	23065800.04	12252782.29	4206179.689	4187275.327	3955504.069	4405871.691	5381979.257	13448274.64	15650445.17	12423566.18
Com_4912_pos	2-(tert-butyl)-6,7-dimethoxy-4H-3,1-benzoxazin-4-one	--	C14 H17 N O4	263.11567	5.281	264.12296	12296263.43	32094619.28	15559328.56	11806080.11	12355589.4	15649453	1884163.022	1859033.919	3096324.526	3173448.181	1461857.936	1976780.597	8892414.473	6926554.943	6592198.514
Com_4926_pos	Dehydroepiandrosterone (DHEA)	--	C19 H28 O2	270.19822	7.652	271.2056	15588064.88	13701129.9	13128852.92	14093053.24	10182114.19	10026176.56	9256949.686	15704037.69	6342346.294	11790402.38	6788458.477	10088308.15	11167764.82	10374756.72	10526789.83
Com_4943_pos	L-Tyrosine methyl ester	L-Tyrosine methyl ester	C10 H13 N O3	195.08941	6.065	196.09669	15176613.89	23533452.77	26897434.99	17243339.43	20021858.83	21323114.41	9573501.75	3833115.544	5183063.492	4053982.806	4643134.023	5798427.619	9144571.24	8599470.323	10332521.81
Com_5029_pos	Ethyl oleate	--	C20 H38 O2	310.28687	7.49	311.29419	903164.1413	1123384.784	1775606.231	1423756.84	940465.282	955844.1809	4039588.734	6040851.111	9629331.438	17559831.45	3813154.433	6467912.769	5180348.909	4661310.26	4827664.155
Com_5071_pos	Norbutorphanol	--	C16 H21 N O2	259.15742	4.819	260.16479	106444.5969	103378.5931	90175.53596	86661.59409	108570.7034	112203.1572	6965060.074	342949.8232	7416961.545	17355213.13	243049.3918	499449.9065	1871382.851	878209.4824	1688723.614
Com_5079_pos	N-[(dimethylamino)methylene]-	--	C10 H14 N2 O2 S	226.08204	5.344	227.08969	8211807.407	10057085	8567202.168	12415121.37	14872341.08	11425053.56	7415570.972	5547726.432	8653043.518	8068724.286	17825618.49	8631037.622	13052497.77	7660683.158	12893940.93
Com_5134_pos	LPE 16:0	--	C21 H44 N O7 P	453.2846	9.364	454.29193	11410483.37	28025320.3	18279564.27	7780228.313	27290274.51	10258100.59	3208625.523	990673.8343	837050.5343	2219043.696	1408896.45	733459.6481	6475506.837	7760867.661	8008492.57
Com_5150_pos	11(Z),14(Z),17(Z)-Eicosatrienoic acid	--	C20 H34 O2	306.25584	10.518	307.26297	4333699.582	4775338.954	4049629.765	2851147.233	7604706.698	5644763.584	7412318.243	14697892.58	3674045.594	2201210.362	5504711.188	6461060.621	9232551.633	5919684.165	6279893.585
Com_5160_pos	gamma-Tocopherol	gamma-Tocopherol	C28 H48 O2	416.36505	9.878	417.37234	3040320.434	3147021.29	3317093.183	13726944.64	3333183.849	3754756.016	3041238.9	2613691.593	6046387.821	16845361.64	2422103.326	2967019.505	3338000.997	3781510.735	3551140.088
Com_5172_pos	17alpha-Ethinyl estradiol	Ethynyl estradiol; 17alpha-Ethinyl estradiol; Ethinyl estradiol; Ethinylestradiol	C20 H24 O2	296.17706	7.427	297.18442	1980684.553	2391565.2	2348839.031	2575669.693	1667113.251	2404397.713	11934824.58	9011476.916	12267274.64	16790133.63	10944247.11	8497260.858	5304724.501	6583035.839	7162232.509
Com_5194_pos	3-benzyl-4-hydroxy-5-(4-hydroxyphenyl)-2,5-dihydrofuran-2-one	--	C17 H14 O4	282.08901	5.296	283.09616	8028279.829	10981240.6	24988382.44	9885881.209	12189910.33	10519206.53	2281681.74	2759474.542	3082514.078	1911160.923	1204411.705	2569608.84	5555939.726	5388619.12	4590424.301
Com_5204_pos	Methylimidazoleacetic acid	Methylimidazoleacetic acid; Tele-methylimidazoleacetic acid; 1-Methyl-4-imidazoleacetic acid; 1-Methylimidazole-4-acetate; Methylimidazoleacetate	C6 H8 N2 O2	140.05843	1.278	141.06563	21609698.05	29453275.67	20502470.35	21211685.38	14807839.79	14748141.6	1516079.513	2829376.887	1387400.295	2907684.129	1837621.446	2315038.405	8515427.113	8091371.738	7602839.482
Com_5206_pos	N-allyl-2-(2-methyl-1-benzofuran-3-yl)acetamide	--	C14 H15 N O2	251.09471	5.001	252.10199	634302.6653	581229.7003	398979.1077	688626.7236	550256.5788	505590.7615	4569346.17	3419358.622	6654540.851	16647216.45	6185021.16	5156493.482	3190051.997	2700750.304	2695627.35
Com_5279_pos	3-hydroxy-1,5-diphenylpentan-1-one	--	C17 H18 O2	276.11253	4.766	277.11981	2416759.138	2340415.831	1647168.719	881784.2637	946904.1919	2028667.93	7067089.103	239095.3059	10963928.38	16293150.23	981581.3401	269263.4756	5242300.141	3203088.369	3223037.882
Com_5294_pos	(5E)-7-methylidene-10-oxo-4-(propan-2-yl)undec-5-enoic	--	C15 H24 O3	274.15441	6.763	275.16168	453682.4951	713161.7488	408579.8763	485344.85	374235.8788	476110.7239	2921536.905	6021245.446	11579080.03	16244184.25	13845675.06	7084414.399	4900334.108	5248808.703	5438081.147
Com_5453_pos	Furanyl fentanyl 3-furancarboxamide isomer-d5	--	C24 H21 [2]H5 N2 O2	379.23557	5.027	380.2428	2332444.729	2349936.446	3269405.538	3215785.56	3100177.736	2973714.208	2809795.314	205564.3555	12190515.45	15478538.86	4352455.913	345262.0293	4485067.816	4330133.355	3719452.374
Com_5468_pos	α-Hydroxymidazolam	--	C18 H13 Cl F N3 O	341.07603	5.415	342.08282	4454043.02	5904231.784	6078379.751	6370449.071	5467226.718	4988548.048	15882438.13	9721364.344	9715805.895	4699393.181	7389896.676	11766313.05	7563773.596	6716088.883	7110516.222
Com_5520_pos	Propionylcarnitine	--	C10 H19 N O4	217.13125	1.889	218.13855	798000.3463	944335.0541	1392400.567	1533791.26	1459130.483	1139547.52	2074598.084	276838.833	248861.7261	235167.0885	15646135	274658.74	908484.605	712002.8936	815489.7249
Com_5534_pos	D-(-)-Aspartic acid	--	C4 H7 N O4	133.03741	1.26	134.04463	2906257.7	4763775.7	8795928.629	7935231.917	7980503.082	7689934.751	1860715.88	13196213.69	6525365.364	7630692.934	1759094.997	11182433.4	7327333.519	6049311.88	5728893.282
Com_5537_pos	2,4-Dimethylbenzaldehyde	--	C9 H10 O	134.0731	5.473	135.08041	8156108.11	7461350.418	6127949.421	6694126.907	6116507.747	5954071.086	8777325.797	8368331.369	13033184.62	15158647.98	8816293.087	13316628.99	7957438.43	9017958.202	7660356.524
Com_5577_pos	Isobutyryl carnitine	--	C11 H21 N O4	231.14712	5.131	232.1544	23539196.39	18424956.29	14760781.23	14682291.75	14948634.25	14373472.41	1397138.56	1340972.703	1230139.457	1617782.695	1433801.115	1589339.741	7259317.97	7137117.963	7614547.997
Com_5590_pos	Sedanolide	--	C12 H18 O2	194.13054	6.71	195.13779	23466626.98	20892256.47	17740307.99	17169859.39	19152223.8	17436275.48	5478360.927	5150225.037	5424999.781	7522630.868	4580448.498	5082351.488	10509152.64	10364051.08	10339514.95
Com_5620_pos	4,7,8-trimethoxyfuro[2,3-b]quinoline	--	C14 H13 N O4	259.08394	5.48	260.09131	17298963.15	18155128.54	17828612.58	19779942.67	11546715.49	12511778.28	263637.1226	381369.5559	151208.9899	189369.2695	495494.9933	277800.4544	7091565.505	6377165.472	6747729.508
Com_5677_pos	Amlexanox	--	C16 H14 N2 O4	298.09518	6.301	299.10251	17469019.94	16164356.9	19377076.22	19459692.54	22340001.79	21044150.51	425259.5968	204442.3196	438768.475	282069.1075	459293.7911	226554.5415	7379466.548	8112065.259	8408602.074
Com_5685_pos	2,3-dihydroxypropyl 12-methyltridecanoate	--	C17 H34 O4	284.23479	8.61	285.24203	5609376.637	5295468.177	4007464.743	3754985.733	5868364.792	4566320.982	13757031.42	10287413.22	13656079.18	3901040.479	13507612.45	15476820.2	8490080.752	9022035.714	9159323.88
Com_5699_pos	IMH	--	C17 H29 N5 O4 S	399.20053	5.059	400.20798	14106816.72	18011856.82	17679713.42	19346401.61	14244647.6	14354286.25	698734.4675	443964.6912	155473.0679	225411.284	160209.1733	698718.3464	5978213.929	5562233.943	5417637.586
Com_5702_pos	2-morpholino-1-phenyl-1-	--	C12 H17 N O2	189.11592	6.269	190.12314	567909.4963	779897.6745	568518.666	568816.0216	697392.2077	466158.7606	3364600.439	12589233.03	1073484.859	2973948.688	1297790.765	3712726.687	2386253.908	2483670.527	2224986.277
Com_5733_pos	Saxitoxin	Saxitoxin	C10 H17 N7 O4	281.12579	5.673	282.13336	13635451.08	12682723.78	4613617.889	18318863.14	9950074.968	11787944.59	9812052.778	4619886.349	9944360.965	8860658.169	14768464.4	5415490.588	9499100.026	8032476.898	8083902.43
Com_5747_pos	16-Heptadecyne-1,2,4-triol	--	C17 H32 O3	306.21711	8.188	307.22427	13554197.09	13070262.83	11211512.88	10337930.31	22962988.82	22478347.13	13507445.63	12197818.73	12090566.99	8399331.29	11611703.84	13715946.55	10815147.23	10898558.93	8653263.334
Com_5753_pos	4-morpholinobenzoic acid	--	C11 H13 N O3	207.0893	1.352	208.09657	1077450.313	1931931.067	1120084.108	1023037.713	1448634.881	1655169.031	13964913.36	9920364.371	6871242.135	9277083.536	4079765.568	15165980.09	4665972.935	4501273.776	4144000.317
Com_5756_pos	Mag (18:1)	--	C21 H40 O4	356.2914	9.294	357.298	7899896.756	3871009.138	7080058.818	9513899.401	22887794.78	23571211.45	3574686.321	2942889.918	3680102.864	2080496.941	2359643.499	2495468.678	5648927.663	5852589.928	5081718.759
Com_5769_pos	1-(3-methoxy-2-nitrostyryl)pyrrolidine	--	C13 H16 N2 O3	248.11602	4.873	249.12306	13998143.66	21106708.38	21167082.56	18567262.45	10934268.38	7281870.771	3259907.335	2397397.617	1531478.167	2188378.015	9174315.315	2221739.328	5510730.905	7215527.116	5341319.264
Com_5811_pos	5-Methylcytosine	5-Methylcytosine	C5 H7 N3 O	125.05911	1.348	273.10782	6033294.201	6692204.496	7007330.695	7313284.677	6101222.289	7974114.017	9710082.653	10175088.57	11746381.33	11372905.63	5577674.386	14914864.16	5437622.344	5600221.292	5245055.251
Com_5834_pos	1-hydroxy-1-(4-methoxyphenyl)propan-2-yl 4-methoxybenzoate	--	C18 H20 O5	338.11485	7.202	339.12213	892340.8176	730481.5248	1070929.008	1349629.342	1333638.311	1039643.621	10784405.94	7805754.942	11097284.72	13910909.38	9541828.328	6409947.314	5070008.642	6110504.009	6958184.312
Com_5876_pos	Cholest-4-en-3-one	Cholest-4-en-3-one; Cholestenone; 4-Cholesten-3-one	C27 H44 O	384.33906	8.376	385.34622	13389709.14	15158323.17	18345560.17	18339376.7	20786756.86	18943508.89	6062603.7	5245382.516	7877534.336	8920950.867	2520417.588	4730394.137	8332663.523	10517621.27	9711493.427
Com_5886_pos	4-methyl-5-oxo-2-pentyl-2,5-dihydrofuran-3-carboxylic acid	--	C11 H16 O4	212.10305	5.211	213.11018	10714403.99	12145610.46	12727826.57	12046028.91	13075324.49	14712768	10924054.1	11916021.88	3944047.973	8493447.268	10049895.77	10037080.92	10315015.06	8976120.328	8915109.197
Com_5927_pos	N1-(2,3-dihydro-1,4-benzodioxin-2-ylmethyl)-2,2-dimethylpropanamide	--	C14 H19 N O3	249.13605	1.363	250.14322	4924693.926	7902601.167	7322259.299	6758860.279	11137026.78	7715086.718	6687089.36	10139832.92	7828750.745	9852641.778	3838687.311	14415565.37	6581356.165	6941995.959	7948101.84
Com_5930_pos	ILK	--	C18 H36 N4 O4	186.13805	11.035	187.14536	3047769.513	4860407.65	8660723.747	5122951.037	21751244.99	9071811.958	6484497.015	4044865.919	6015873.502	5632779.59	3733464.436	3623052.193	5627557.02	6167792.254	6266662.552
Com_5937_pos	ethyl 1-(4-acetyl-2-aminophenyl)piperidine-4-carboxylate	--	C16 H22 N2 O3	290.16296	5.038	291.1702	1378223.239	1492165.578	1269106.124	1051143.842	1958216.746	1393758.279	2903493.444	5946070.453	15228117.51	11807304.67	629907.7839	6974144.626	3536522.231	3530223.95	3355995.937
Com_5950_pos	4-(2,3-dihydro-1,4-benzodioxin-6-yl)butanoic acid	--	C12 H14 O4	222.08655	6.149	223.09386	2262990.962	2985305.207	3430695.643	2822811.79	2715976.942	2609715.493	7354657.417	2283912.34	15166599.36	10001223.24	2230675.187	3089542.598	4526865.545	3304012.415	3316627.557
Com_5957_pos	Cholecalciferol	Vitamin D3; Cholecalciferol; Calciol	C27 H44 O	384.33874	8.598	385.34583	7921114.36	9720643.873	17217212.99	17945007.39	17372896.94	15927146.34	3743764.657	3513658.157	3640656.486	5905322.291	1134950.656	2821374.833	7910593.258	7561299.004	7552367.84
Com_6005_pos	LDGTS 16:0	--	C26 H51 N O6	473.37181	9.369	474.37897	2664101.582	2668860.262	3203452.716	2695700.883	8582877.412	4163449.134	3506786.262	11562083.64	3428356.654	5357029.987	2563591.975	7940011.647	5769706.426	5861480.306	4862693.561
Com_6071_pos	Taurochenodeoxycholic Acid (sodium salt)	--	C26 H45 N O6 S	481.27801	5.446	482.28552	14675374.05	23044475.4	18521467.25	16996903.71	17766120.83	20833091.28	412363.1354	462397.8231	430992.4054	539761.0091	450649.5688	439649.0545	7319071.844	7325609.328	6212212.219
Com_6129_pos	Cafestol	Cafestol	C20 H28 O3	316.20262	6.775	317.2099	5805465.359	5372647.653	4356783.078	4723052.161	5265976.585	5197596.521	10621321.25	7366241.385	11424337.13	10297221.94	13254958.97	11964100.97	6865881.723	6623481.388	6603879.925
Com_6157_pos	Enrofloxacin	--	C19 H22 F N3 O3	381.14648	6.576	382.15384	8817936.14	4036141.444	5340934.169	5164662.956	6252141.158	9642420.836	1637747.361	7841791.732	14397226.29	531695.3725	4609810.955	10585098.26	6149374.387	5657695.74	5665790.557
Com_6242_pos	7-{[(2E)-3,7-dimethylocta-2,6-dien-1-yl]oxy}-2H-chromen-2-one	--	C19 H22 O3	320.13703	4.726	321.14435	11804378.99	9782421.484	9168158.874	10248705.84	8926915.533	11287183.29	1661024.142	1294543.707	1285031.158	12532757.99	797724.478	1196058.441	4616892.211	4448330.999	4359222.061
Com_6245_pos	7-(2-aminophenyl)heptanoic	--	C13 H19 N O2	243.12156	1.361	244.12875	9721296.628	12646135.63	10262648.24	10130269.77	13737373.97	11495135.22	3741045.302	7096087.744	4436912.717	8828942.374	12874589.57	8310884.652	7784842.025	8099356.616	7198403.558
Com_6260_pos	3'-Hydroxystanozolol	--	C21 H32 N2 O2	362.2605	10.781	363.26758	304214.1218	239066.3346	223407.5143	228790.6662	280929.6898	211619.7516	11706700.93	10834325.18	371219.2858	659977.0516	2721815.607	5069658.505	2914169.262	2919015.724	2836419.86
Com_6264_pos	2-oxo-2H-chromene-3-carboxylic acid	--	C10 H6 O4	190.02952	1.023	191.03674	13918148.6	15955311.9	11551637.91	11676648.37	13431991.89	16057984.92	4934270.439	4543355.853	7048894.035	6194337.022	4855876.697	3461781.331	11735262.44	10126310.9	8525020.483
Com_6296_pos	3-(4-hydroxy-3-methoxyphenyl)propanoic	--	C10 H12 O4	218.05769	5.299	219.06494	16941645.15	21577115.56	16673729.65	16470099.88	18023924.29	16888797.07	5533547.873	5088100.475	5366972.066	4084979.236	3569239.555	5066476.052	10954860.48	12084647.28	9537998.642
Com_6470_pos	5,7-dihydroxy-2-phenyl-4H-chromen-4-one	--	C15 H10 O4	254.05763	6.836	255.06493	529975.1922	826709.8343	645754.7615	852520.3678	1083765.14	1717653.769	12165640.38	2767581.28	3544923.286	3700900.826	1665220.948	3159072.267	2707072.525	2301543.788	2398170.386
Com_6632_pos	1-[(3,5-dimethylisoxazol-4-yl)sulfonyl]piperidine	--	C10 H16 N2 O3 S	244.08495	5.044	245.09218	17745493.36	13734268.62	7363582.4	7185641.902	17135795.33	14733854.44	638019.5144	291902.5726	698463.5134	891464.4274	1043618.521	694449.6991	6582389.48	5501581.646	5737986.711
Com_6667_pos	1,7,8-trihydroxy-3-methyl-1,2,3,4,7,12-hexahydrotetraphen-12-one	--	C19 H18 O4	310.12053	5.696	311.12784	2157690.013	1313661.48	1815962.387	1518384.997	1397341.554	2163061.093	11540962.9	3525864.415	7025387.437	3198655.651	2064610.806	868288.2834	2899375.031	2871045.925	3986507.774
Com_6710_pos	Eriodictyol	Eriodictyol	C15 H12 O6	288.06316	7.853	289.07043	629545.0475	665163.6979	942167.9577	894808.4201	683084.0959	662463.8794	3269297.899	9612885.009	2992399.535	2146071.098	821050.6786	7309479.248	2055074.867	2197497.595	1920932.36
Com_6758_pos	Guanidinosuccinic acid	N-Amidino-L-aspartate; N-Amidino-L-aspartic acid; Guanidinosuccinic acid	C5 H9 N3 O4	175.05827	5.034	176.06551	7876159.708	6492038.406	14140094.88	14598050.75	8969849.676	8893876.961	1203061.111	5835203.047	6027250.866	1412854.707	3519553.68	7587778.846	4458902.739	3666084.965	6752343.635
Com_6760_pos	Metanephrine	--	C10 H15 N O3	197.10513	5.769	198.11241	17148000.42	8001400.968	5525378.51	5385846.044	8417262.169	14949613.08	5645626.363	3465121.271	3749487.352	5408041.491	3744128.216	5001430.598	5708382.591	5498169.338	6168159.37
Com_6766_pos	FQH	--	C20 H26 N6 O5	860.40109	8.249	861.40894	665386.2384	899678.0075	1109193.065	1301444.551	569310.7851	686514.2777	10596734.71	9486973.536	5785570.039	1015099.874	2006068.807	8718087.901	3245230.934	3033905.28	3121001.208
Com_6775_pos	2-Methylbutyroylcarnitine	--	C12 H23 N O4	245.16264	5.283	246.16994	17091153.3	17594472.31	14909970.07	13398748.22	11558604.21	13416771.76	1097163.335	1124650.32	862660.3549	1130693.563	1291248.055	1309171.468	8601360.372	6401842.724	6141823.644
Com_6833_pos	N'-{6-[(5-chloro-3-pyridyl)oxy]-3-pyridyl}-N,N-dimethyliminoformamide	--	C13 H13 Cl N4 O	276.07781	5.696	277.08502	14689306.89	16148792.17	14136213.5	13806120.09	17289058.28	13782470.91	6941765.466	2607030.79	6134715.147	5373859.832	4085601.782	5386134.985	9334008.123	10335656.1	8855063.944

Com_6856_pos	Egconine methyl ester	Egconine methyl ester; Methyl ecgonine	C10 H17 N O3	199.12058	1.37	200.12784	12404143.58	13873539.83	15604761.51	14279509.52	13012902.82	10637658.45	4221773.141	4627177.65	4454869.156	5276505.285	5285755.265	4016044.458	8417691.27	9609234.742	10274372.52
Com_6910_pos	4-acetyl-4-(ethoxycarbonyl)heptanedioic acid	--	C12 H18 O7	296.08873	5.233	297.09564	16606561.32	16882872.35	8143247.032	8132335.889	9086910.726	7819895.07	4517614.713	3553782.995	4552891.614	4444690.036	6292848.796	4352806.16	4513358.789	5019078.049	5047744.182
Com_6948_pos	Palmitoylethanolamide	Palmitoylethanolamide; Palmidrol	C18 H37 N O2	299.28214	9.277	300.2894	7612318.244	9016401.109	3444844.317	3380843.19	12805108.13	10610063.86	1149939.112	9132916.718	2938399.223	7077834.198	1798249.037	7991211.261	6824050.471	5777573.345	4807420.931
Com_6949_pos	4-(methylthio)-6-phenyl-2-(3-pyridyl)pyrimidine-5-	--	C17 H12 N4 S	304.07634	1.321	305.08365	1272075.734	1902430.687	1050222.983	1485705.312	1307805.206	2230937.405	5868705.979	9128725.737	5219222.255	7363617.089	3654191.947	8439098.198	4272252.725	4419945.955	4105775.596
Com_6974_pos	2-deoxyglucose-6-phosphate	--	C6 H13 O8 P	244.03475	5.461	245.04189	705545.1446	726901.6974	707473.7811	650850.6902	1038453.35	854963.8443	4734422.199	8422301.245	7130843.034	6049816.4	4053630.452	11101993.78	1520767.057	1532914.852	1731378.841
Com_6983_pos	Deoxyinosine	Deoxyinosine	C10 H12 N4 O4	252.08553	3.842	253.0925	1744224.225	1756083.461	1612073.099	1486541.216	5792159.832	18857522.92	1112145.761	6873994.949	5520956.174	2998369.163	1034917.246	8071113.897	3690428.66	3969441.223	3083309.284
Com_7054_pos	Lysope 14:0	--	C19 H40 N O7 P	425.25352	8.591	426.26123	16110562.58	12760083.87	6906108.204	5128626.44	9484165.215	7639003.211	1356228.884	375380.2525	454884.4427	484526.9576	309336.5652	568989.3846	3898191.94	5781990.907	4594945.134
Com_7100_pos	(2,6-dimethylpiperidino)(3,4,5-trimethoxyphenyl)methanone	--	C17 H25 N O4	307.17834	5.032	308.18558	15515799.96	17979511.85	14608064.25	13289773.17	10920184.23	11392076.76	1442665.743	2558154.098	2516203.78	2433409.971	1153104.364	1495082.24	7467418.685	5796524.452	6290070.338
Com_7105_pos	Azetidine-2-carboxylic acid	--	C4 H7 N O2	101.04784	1.265	102.05515	2933272.958	4343426.71	6195674.253	7813865.525	10104998.9	7380323.761	1517678.166	5804506.038	7948900.272	10151170.68	5941776.28	9466422.073	5850155.374	5800307.47	5331728.649
Com_7115_pos	Eicosapentaenoic acid ethyl ester	--	C22 H34 O2	347.27966	8.513	348.28683	8734093.303	6580059.237	5106362.69	4561355.741	9962303.313	7235319.239	8720264.849	8006986.413	9128199.611	8179292.276	6322784.848	5990248.045	9546513.363	8868674.496	9691810.511
Com_7128_pos	Cortodoxone	11-Deoxycortisol;	C21 H30 O4	346.214	5.72	347.22156	15807980.85	13005812.56	12298553.62	11596395.39	14037181.14	16839343.9	6939558.97	4206054.778	4436613.893	6866712.628	5440116.663	5153483.917	8532179.131	7206251.631	8859072.385
Com_7227_pos	4-oxo-5-phenylpentanoic acid	--	C11 H12 O3	209.10516	4.654	210.11246	9259317.58	11751914.12	7737532.506	8088710.197	15734171.31	14975703.76	815192.3747	876880.9042	696150.1361	1283462.596	542262.756	863162.7497	4476249.616	4438208.728	4318374.647
Com_7238_pos	D-Erythrose 4-phosphate	D-Erythrose 4-phosphate	C4 H9 O7 P	200.00838	1.149	201.01564	9064557.191	9429501.269	5179891.658	4077631.051	7923578.143	7970003.763	9904325.946	7666592.774	8177258.765	7102590.188	4191456.062	5875709.099	9213881.003	5734850.115	4749415.696
Com_7255_pos	Methylhippuric acid	--	C10 H11 N O3	193.07375	1.357	194.08102	2196273.17	2832431.494	2139454.348	1998127.637	2361740.835	2269604.308	3579996.127	8472515.752	5738108.989	1968974.84	2740076.065	9134657.859	1902100.528	1838325.819	1926157.849
Com_7299_pos	Milbemycin A3 oxime	--	C31 H43 N O7	541.30413	5.312	542.31152	358641.7501	367067.8814	334392.1682	311814.3993	301532.6079	329075.1926	1613132.132	147616.3196	6556089.036	9649247.914	5304672.754	149727.9859	1385033.466	1652589.356	2011628.169
Com_7301_pos	Bz-RS-ISer(3-Ph)-Ome	--	C17 H17 N O4	299.1155	4.833	300.12277	1515462.94	1499820.804	1643707.578	681110.9635	1416406.514	1354385.376	3337847.546	8385663.408	8089798.85	2561661.553	209651.9046	7847840.19	2960378.708	3164711.331	3551782.15
Com_7307_pos	O-7460	--	C25 H48 F O5 P	500.3134	5.971	501.32068	5971521.751	5414203.363	5245109.538	3989174.191	5533872.821	7749973.944	6390355.918	5425905.266	8911707.969	9629485.964	7316735.507	2702576.563	4549234.273	4424359.44	6921516.652
Com_7322_pos	2-piperidinobenzoic acid	--	C12 H15 N O2	223.12084	4.78	206.11763	7296246.627	6906405.608	4511899.066	5596899.224	12649233.27	11089334.38	9860456.074	4287937.385	6023519.949	8420636.114	2184265.376	4572285.974	6111032.735	3636775.365	5539867.569
Com_7499_pos	Gemifloxacin	--	C18 H20 F N5 O4	389.15029	4.872	390.15765	2789746.312	1888120.001	3638204.112	2921235.38	3792758.205	3215897.987	8098446.466	6805783.632	5911214.856	9178284.703	1440327.235	7162110.895	3388557.969	3105635.873	3315868.632
Com_7530_pos	(6E)-7-(2H-1,3-benzodioxol-5-yl)-1-(piperidin-1-yl)hept-6-en-1-one	--	C19 H25 N O3	315.17874	1.357	316.186	5933498.87	8140741.272	7403769.039	7398683.824	11399561.45	9510896.256	2279402.845	3638338.096	5498300.807	9101664.297	4418587.703	4640536.327	5298321.812	5125105.551	5494705.765
Com_7669_pos	Biochanin A	Biochanin A	C16 H12 O5	284.0683	6.45	285.07538	1155648.435	1160695.776	1410761.963	991633.8188	1307596.518	1402349.581	9147167.918	2768155.933	3727626.67	4728041.471	6179518.7	2922610.593	2135010.961	2288280.294	2687742.38
Com_7706_pos	Narasin	--	C43 H72 O11	746.50859	9.333	747.51587	395361.6125	455592.3631	368424.0448	227462.1799	1044156.285	345953.9043	4763876.868	1422535.535	3201504.141	1614717.369	9049301.773	5431963.534	2117045.915	2018406.134	1891893.441
Com_7726_pos	delta-Tocopherol	delta-Tocopherol	C27 H46 O2	402.34987	10.674	403.35715	344509.6599	2012386.937	1374866.341	942887.9725	361610.5639	4122860.013	2506592.885	4017495.672	1468227.306	8757994.022	2723745.565	2770141.829	4702995.904	5305515.197	6219130.457
Com_7761_pos	(3R)-8-hydroxy-3-(4-methoxyphenyl)-3,4-dihydro-1H-2-benzopyran-1-one	--	C16 H14 O4	252.07846	6.319	253.08574	8330789.728	10671952.36	12416664.9	11591230.66	9359715.465	11134265.6	2146730.534	1635813.639	2066479.639	1508499.749	706439.5544	1691494.225	6352472.973	5792017.782	6635849.113
Com_7773_pos	MMH	--	C16 H27 N5 O4 S2	417.14882	1.281	418.15607	249167.7433	274043.2162	8718402.171	11551408.27	1070474.301	686056.285	379330.2035	1789915.476	169526.1262	2339207.187	353308.624	519838.3806	2057227.353	1616588.452	2353241.376
Com_7793_pos	N~5~-(pyridin-2-ylmethyl)-1H-1,2,4-triazole-3,5-diamine	--	C8 H10 N6	190.09659	11.195	191.10387	1203329.163	1290989.96	2903804.345	1025737.696	1172596.725	928935.8004	4096517.557	7485903.995	8184513.585	899078.8832	1145680.213	3826539.496	3685345.497	2825274.218	3647870.007
Com_7802_pos	Egonine	Egconine	C9 H15 N O3	185.1024	4.936	186.10959	11767487.62	8704407.407	9610256.419	11465714.28	11134271.14	9438653.433	2228988.151	1879290.954	2339189.289	3095405.902	6858732.785	2073437.046	6409418.429	5750643.667	5553698.64
Com_7827_pos	RMK	--	C17 H35 N7 O4 S	433.24623	4.946	434.25351	208673.4651	210096.6895	187494.4839	188013.9639	219626.6242	216594.5357	8789537.992	1707647.375	2788048.818	4067415.456	5827490.287	1973329.038	1590838.948	1465959.712	1411376.175
Com_7871_pos	6β-Oxycodol N-oxide	--	C18 H23 N O5	333.15746	5.21	334.16473	1292830.485	1027345.662	1288964.257	827797.3664	1013680.781	1035254.6	8626824.034	6848671.496	6449550.972	8431006.056	834307.2977	6907953.319	3359517.341	2877751.758	3143256.763
Com_7887_pos	13,14-dihydro-15-keto-tetranor Prostaglandin E2	--	C16 H26 O5	320.16015	6.72	321.16718	6313456.13	6705932.51	6045902.833	5247298.635	5553092.146	15227000.54	2968257.553	2583484.087	2927420.729	3349213.349	2796183.655	2033801.594	4381532.603	3873814.914	3777715.659
Com_7903_pos	Meperidine-d5	--	C15 H16 [2]H5 N O2	252.18763	10.211	253.19489	555716.5802	499452.2854	644712.1841	384262.0084	1003780.981	470388.0714	6137055.958	7290401.531	1751760.292	5751467.903	1540103.221	6386775.589	2777242.697	3245054.662	2888166.205
Com_7942_pos	5-hydroxy-6,7-dimethoxy-2-phenyl-4H-chromen-4-one	--	C17 H14 O5	280.07145	5.853	281.07861	12995002.47	2507406.59	10138939.27	10717486.53	8167863.225	12191693.58	5486965.767	2306584.952	3955802.78	2743778.843	3058758.795	2553593.91	7759359.143	7026812.454	7038328.79
Com_8022_pos	Isoproterenol	Isoproterenol; Isoprenaline	C11 H17 N O3	211.12044	1.353	212.12758	11904556.84	13577820.77	9956475.142	9957494.536	12958572.42	11531578.54	2291746.312	3147855.492	3438638.37	5138500.269	3598689.031	3761279.162	7663661.168	6525664.451	6546480.31
Com_8026_pos	1-(4-methyl-2-morpholino-1,3-thiazol-5-yl)ethan-1-one	--	C10 H14 N2 O2 S	226.08168	5.3	227.08896	548712.145	4940356.572	844857.1719	623127.0359	5547288.68	4531310.461	7415570.972	5547726.432	8653043.518	6013960.614	5606366.572	8631037.622	5550282.405	4820478.139	4870254.965
Com_8164_pos	1-oxo-2,3-dihydro-1H-inden-4-yl benzoate	--	C16 H12 O3	252.07856	5.314	253.08586	5725501.624	7446619.353	6778791.09	10505712.17	6128289.232	3504423.257	723917.7745	430063.159	644715.091	662444.9909	475180.0806	529269.5973	3139852.257	2671906.845	2669570.428
Com_8170_pos	5-Methyl-2'-deoxycytidine	5-Methyl-2'-deoxycytidine	C10 H15 N3 O4	241.10591	1.226	242.1133	4294045.515	8503262.292	11725109.79	9924650.266	4539395.277	3699727.09	79153.25719	194366.5805	111868.3019	969878.3895	527641.1431	151637.061	3886004.441	2173810.335	2126377.811
Com_8291_pos	Guggulsterone	--	C21 H28 O2	312.20882	8.397	313.21609	1638324.208	1788137.208	1265011.401	1836747.135	1807720.372	1797011.337	7887398.624	2720784.049	2949356.134	2084942.368	2925769.841	2632545.068	1442822.804	2918580.692	2846686.709
Com_8308_pos	2,6-di(2-furyl)-3-propyltetrahydropyridin-4(1H)-one hydrochloride	--	C16 H19 N O3	273.13199	1.345	274.13916	6628444.65	9301280.528	9639794.309	5904084.154	12256918.01	6799700.627	1813697.002	2641940.02	3239223.047	3742284.313	4315071.919	3001433.795	4728279.302	4751101.759	5079937.923
Com_8330_pos	Lysopa 16:0	--	C19 H39 O7 P	410.2443	9.216	411.25229	3616156.923	1766820.675	3531910.064	4839249.359	5205599.958	5150164.413	1036466.696	3971599.405	2053123.719	2023215.456	7820869.182	3733456.72	3165370.511	3148034.272	4036530.451
Com_8413_pos	4-{3-[(3,4-dihydroxyphenyl)methyl]-2-methylbutyl}benzene-1,2-diol	--	C18 H22 O4	324.13263	4.928	325.13959	2947362.72	3323414.872	1796520.892	2847609.75	1628155.213	3046889.416	7710734.06	2218426.325	2909447.253	2879933.911	6408801.462	2381698.36	2780227.888	2512474.672	1322829.802
Com_8426_pos	16,16-Dimethyl prostaglandin	--	C22 H36 O4	386.24524	9.224	387.25253	3477103.785	7515081.954	7591761.824	5701573.736	3728499.017	4461246.436	2442652.306	6492066.479	7187891.556	3065953.046	4670764.116	4706943.36	5251800.604	7490199.624	5983381.322
Com_8556_pos	Celastrol	--	C29 H38 O4	450.27458	6.045	451.28186	9079254.382	8973607.618	4697974.038	9678145.876	9176130.082	6669393.129	2549914.739	1756387.561	2331546.479	4317419.763	3004325.858	1778978.331	4290105.777	4303413.813	5307223.452
Com_8564_pos	Pro-Leu	--	C11 H20 N2 O3	228.14707	1.357	229.15436	10330682.12	12028904.32	8585905.324	8518283.317	11241297.79	9751671.314	1682321.098	2689883.391	2936276.455	4616207.78	4343621.181	3121089.178	6819841.5	5680437.021	5678470.09
Com_8603_pos	(2E)-1-(2-hydroxy-3,4,5,6-tetramethoxyphenyl)-3-phenylprop-2-en-1-one	--	C19 H20 O6	366.11002	7.684	367.11731	209601.8229	224162.4297	221280.0625	318530.8244	306617.2437	258716.1158	699144.7956	1499219.523	6098472.168	7177532.099	427054.4213	928417.2428	1626031.966	1790499.011	1269634.012
Com_8621_pos	Methyl indole-3-acetate	--	C11 H11 N O2	189.07616	6.013	190.08348	9474854.946	8324103.985	8164721.229	7076428.226	10604679.59	12954958.26	1308976.494	1276475.861	1237114.478	1243456.037	1837503.98	1073298.72	4924297.779	4276810.079	4079108.858
Com_8655_pos	Riboflavin-5-phosphate	FMN; Riboflavin-5-phosphate; Flavin	C17 H21 N4 O9 P	456.10493	6.053	457.112	938233.6912	854559.0687	764169.6055	675650.9703	698914.1108	828701.2875	1842328.85	6173796.479	3985685.852	7023025.825	995680.5092	5129808.284	1553781.58	2174190.848	1521066.48
Com_8734_pos	PC (18:4e/2:0)	--	C28 H50 N O7 P	543.33203	9.053	544.33911	9526145.258	12381423.28	7601648.24	5269314.683	8543047.085	7786123.1	644348.1397	369428.8795	389119.0944	424415.7374	596648.771	348456.588	3319056.147	3475652.725	2526227.461
Com_8762_pos	D-Glucarate	D-Glucarate; D-Glucaric acid; L-Gularic acid; D-Saccharic acid; D-Glucosaccharic acid; Glucaric	C6 H10 O8	210.03743	0.993	211.04483	8311262.132	10461331.58	7623820.702	9292292.775	7638616.976	10516083.71	3832651.241	2184540.422	4416231.358	4637000.19	2915065.961	2382029.42	6409725.634	5147046.543	4735861.521
Com_8778_pos	(±)-Abscisic acid	--	C15 H20 O4	286.11631	3.894	287.1235	7728626.681	7765659.864	7803338.387	9261759.072	8978434.884	7330596.974	336171.0582	224630.4082	395906.9155	2253152.756	534715.3734	254787.7668	2616581.947	2225274.982	2255580.026
Com_8803_pos	KPH	--	C17 H28 N6 O4	362.20763	7.341	363.2149	6709179.751	6937674.3	5144621.187	6250150.456	6663697.254	7018203.056	5281303.776	4993002.731	7328409.153	5532791.843	4504636.182	7333502.248	5576098.497	5289720.476	5492896.288
Com_8893_pos	4-(1H-pyrazol-1-yl)-N,N-bis(2-pyridinylmethyl)benzenesulfonamide	--	C21 H19 N5 O2 S	405.12409	1.318	406.13129	7975770.479	6062415.226	3171059.074	1800475.826	10862916.92	6681145.04	575515.466	3350831.576	880587.7159	2725133.183	1228972.115	1090918.261	4028750.154	4319006.8	2986194.345
Com_8951_pos	Bisphenol G	--	C21 H28 O2	329.23529	5.268	330.24271	264597.3797	276782.982	248073.0146	249164.152	252912.0621	299536.56	5370797.548	705743.4684	1320518.309	3034142.235	6878233.203	98681.71814	146240.2422	170757.5289	166244.5336
Com_8960_pos	methyl 7-hydroxy-4-oxo-8-propyl-4H-1-benzothiine-2-carboxylate	--	C14 H14 O4 S	278.05769	5.303	279.06491	9249462.047	10188404.78	9971521.117	7848639.733	6812677.4	8376686.458	587912.5225	549772.1246	377822.0025	348131.3229	481010.1174	549197.5786	5681349.812	4368464.297	5238512.731
Com_8981_pos	2-(3,4-dihydroxyphenyl)-3,5,7-trihydroxy-6-methyl-4H-chromen-4-one	--	C16 H12 O7	316.05803	5.75	317.06525	4046882.344	5966334.047	5153822.317	4650357.934	4821819.775	4757051.096	6829655.62	2953152.809	6274181.746	2963244.391	2258322.89	3966389.336	2988782.696	3464848.702	2737985.899
Com_9003_pos	LPE 18:3	--	C23 H42 N O7 P	475.26946	8.638	476.27658	6057842.541	11668424.36	7576899.267	4054921.543	6823804.041	4830421.393	994349.529	448425.6704	339318.6618	305651.7408	208168.1053	301452.449	2634070.849	3224607.612	2632720.039
Com_9004_pos	2-morpholinophenyl 2-thiophenecarboxylate	--	C15 H15 N O3 S	289.07839	3.709	290.08566	1725835.851	2404697.168	2367693.128	1582645.816	2309424.142	5188050.409	406786.9273	4692622.68	3070230.556	2969114.175	562675.2189	7014426.045	2208597.484	2407194.39	2527322.903
Com_9167_pos	N1-(2,3-dihydro-1,4-benzodioxin-6-yl)acetamide	--	C10 H11 N O3	193.07132	4.68	194.07863	459863.7025	9915137.634	9484757.294	5960838.109	5852944.409	11397703.29	85049.17259	73581.04486	935469.8935	835252.2702	769847.5184	794363.2224	2841182.913	2676476.649	4321483.368
Com_9193_pos	EKK	--	C17 H33 N5 O6	421.25479	6.601	422.26083	7843358.218	7897379.375	3817545.205	8432919.62	4252554.462	5047677.932	695607.8462	1145874.084	1232846.918	1614291.992	1145597.065	1370428.362	3181717.331	2717661.313	2840890.231
Com_9279_pos	N-[2-(4-methoxyphenoxy)ethyl]-2-	--	C14 H15 N O4	261.10325	2.596	262.11057	772851.7769	2095024.177	5561444.72	8325169.366	4479566.617	4870004.264	824623.1578	682160.7364	801412.0187	827165.1117	612003.544	730319.0942	1230424.99	1534416.269	1404017.732
Com_9331_pos	6-(Methylthio)purine	--	C6 H6 N4 S	166.03071	5.14	167.03786	1130735.572	877855.9679	1767258.774	888831.5129	1190909.307	975897.3336	6355719.726	2967330.83	3127253.412	3072841.991	1229030.336	3531226.992	1517120.009	1709863.401	1878097.989
Com_9344_pos	L-Homocystine	L-Homocystine;	C8 H16 N2 O4 S2	268.05592	5.198	269.06311	7679853.778	10440938.51	9178518.753	3541984.07	5900615.94	10835775.13	2627540.226	1565280.097	1962065.741	2030615.91	1844745.29	1935891.403	3680095.963	5665771.809	5794919.713
Com_9443_pos	LysoPE 18:0	--	C23 H48 N O7 P	481.31575	8.999	482.32346	5465860.68	6214976.958	8980288.338	3515701.506	7650367.506	5687465.343	362089.6767	319467.2946	444046.9857	472005.8804	309659.639	347067.6036	1954111.8	2515351.468	2394595.793
Com_9471_pos	5-benzhydryl-1-(phenylsulfonyl)-1H-pyrazole	--	C22 H18 N2 O2 S	374.11113	5.075	375.11844	2046258.999	2823704.556	2523064.426	2363343.23	2110016.525	6576635.457	1465160.362	1490361.071	2811399.46	5971539.766	1819484.294	904602.0026	2102631.89	1978819.09	1722462.366
Com_9533_pos	methyl 3-(1H-indol-3-yl)-2-[(phenylmethylene)amino]propanoate	--	C19 H18 N2 O2	306.14219	1.339	307.14954	6260680.385	8455596.385	6648783.976	6081635.772	9442300.676	6187213.183	2168871.298	3933568.505	2142486.168	2769200.23	1960050.329	3802122.662	4712062.458	4458286.271	4407458.785
Com_9607_pos	dimethyl 2-(3-nitro-2-pyridyl)malonate	--	C10 H10 N2 O6	216.1009	5.315	255.06406	489685.9618	1001569.815	661004.3736	974417.5442	584892.7391	434452.9422	4375362.427	923746.8345	1558470.372	4467436.849	4504643.363	6158180.661	1158935.061	1206636.977	1256741.235
Com_9610_pos	Palmitoyl sphingomyelin	--	C39 H79 N2 O6 P	702.56536	6.325	703.57239	5252273.334	4924832.891	4992978.217	5800147.151	9294579.628	6877038.143	222564.4854	178307.547	200912.1202	246010.9566	1205282.498	175481.6472	3021199.551	2511958.73	2058383.495
Com_9640_pos	(2S)-2-(2-hydroxypropan-2-yl)-2H,3H,7H-furo[3,2-g]chromen-7-one	--	C14 H14 O4	246.08468	1.375	247.09196	6255927.891	7014782.18	7241589.225	7675234.948	8445345.055	6401810.782	682282.9924	1201669.063	1295912.557	894262.0166	1657752.656	1680243.918	3447759.789	3500706.992	4372505.872
Com_9702_pos	Nervonic acid	(15Z)-Tetracosenoic acid; Nervonic acid; (Z)-15-Tetracosenoic acid	C24 H46 O2	366.34941	8.845	367.35681	8900861.389	8574719.386	7104573.662	7328185.189	4373530.419	3899027.862	348719.3267	310423.5425	528507.3161	496770.5941	326618.9968	350306.0676	2465311.872	2763988.387	2728669.491
Com_9754_pos	Oxymorphone	Oxymorphone	C17 H19 N O4	301.13112	4.998	302.1384	1461351.919	1412338.671	1321331.582	1089493.002	983176.0578	1124533.084	5785965.015	4398621.775	4290471.478	2610106.764	1368483.53	5477266.739	1784616.023	1831637.557	1575898.211
Com_9772_pos	(+/-)-Cannabichromeorcin	--	C17 H22 O2	258.16572	6.821	259.17322	184223.7071	191875.2741	192018.1632	270608.526	1517646.644	310978.526	131784.701	404193.0819	1611336.953	5599936.072	1281377.032	2190896.15	217639.9938	235704.7472	222248.0845
Com_9787_pos	2-[(3S)-1-(4-Chlorobenzyl)-3-pyrrolidinyl]-1-methyl-1H-benzimidazole	--	C19 H20 Cl N3	325.13132	5.187	326.13855	235892.4483	241771.6285	218128.8305	190612.298	205162.6255	219463.6827	997755.1113	4850752.827	5845292.385	3153119.762	173701.4663	5272331.267	2119281.762	1734392.886	1977866.823
Com_9797_pos	6-methyl-4-(morpholinomethyl)-2H-	--	C15 H17 N O3	259.12067	6.054	260.12799	2751774.546	3756542.131	3747163.876	1820262.704	8708471.765	10089162.57	649947.3504	540717.6436	887787.7115	981354.4061	827743.1189	685951.3109	2321222.443	2706309.941	2390385.469
Com_9808_pos	5,7-dihydroxy-2-(2,3,4-trihydroxyphenyl)-4H-chromen-4-one	--	C15 H10 O7	302.04225	5.576	303.04953	999719.296	990932.2495	1156606.478	1025973.163	1077175.348	1002118.141	3399195.424	3795620.563	2588823.174	5561025.572	2381810.426	3025811.962	1459017.508	1742183.562	1385753.9
Com_9822_pos	Ergocalciferol	Vitamin D2; Ergocalciferol	C28 H44 O	378.32777	10.359	379.33548	2036734.556	2359926.447	2034176.674	1172399.409	2262822.171	1648014.994	3701514.246	4814849.414	3435051.127	4278756.607	3196377.294	3391053.724	2378937.758	3194802.749	2956191.51
Com_9867_pos	L-Kynurenine	L-Kynurenine; 3-Anthraniloyl-L-alanine	C10 H12 N2 O3	208.08492	3.754	209.09219	8635128.011	7290280.651	5439969.421	5753023.15	4780731.891	4706471.546	487234.6811	303598.8875	532351.3852	599102.4352	467364.9984	368156.2755	1626469.98	2073930.243	1810048.195
Com_9872_pos	Celestolide	--	C17 H24 O	266.1624	1.357	267.16974	1158492.483	1895673.522	1365965.072	1142331.2	1711743.672	1352620.946	1506356.618	361402.7267	390562.7793	356015.6003	5654403.514	451842.8696	1172048.004	837936.4432	774665.3815
Com_9910_pos	5-acetyl-2,6-dimethyl-1,2,3,4-tetrahydropyridin-4-one	--	C9 H13 N O2	189.07631	4.758	190.08359	555532.8198	514374.6366	503319.3487	498169.4367	433980.6341	504701.2227	5626805.862	684783.9436	1137497.093	1139239.657	1193641.766	849724.0735	1177220.363	1001156.821	1034755.542
Com_9966_pos	PC (14:0e/3:0)	--	C25 H52 N O7 P	509.34764	9.713	510.35489	1550043.526	4018552.266	8049619.208	1831920.695	2785981.164	1515460.164	1450553.108	1288538.105	557738.7985	5277768.159	552845.2697	899708.0449	2955828.922	2375959.118	2145356.185
Com_9975_pos	4-Aminobiphenyl	4-Aminobiphenyl	C12 H11 N	169.08896	2.938	170.09624	8438632.316	5204195.46	2544821.962	1697365.886	4139310.133	3450014.381	174876.9673	153643.3907	182962.6594	195985.1455	145090.5729	160987.6889	1334179.016	1234235.027	1205843.004
Com_10119_pos	(2R,3S,4S,5R,6R)-2-(hydroxymethyl)-6-(2-phenylethoxy)oxane-3,4,5-triol	--	C14 H20 O6	306.10754	7.735	307.11465	8206710.572	5351898.843	6312695.268	4869289.76	6137360.725	8159505.962	718623.5301	480012.0162	594365.3266	690827.1884	743107.1299	561796.2263	2010169.749	1964552.518	2288798.346
Com_10146_pos	APH	--	C14 H21 N5 O4	305.14669	1.36	306.15405	5460657.468	5965909.579	5267643.992	5285495.185	8330691.214	6403446.675	1306010.916	915781.9328	1495323.571	1228882.297	1551767.592	1366896.834	2576140.584	2572935.329	2545704.551
Com_10228_pos	YLK	--	C21 H34 N4 O5	444.22775	6.815	445.23486	2667515.631	3607691.505	3318313.007	2688255.424	2502502.163	2712497.714	1894848.062	2070076.953	1592215.35	1305870.976	2575529.191	5406425.69	2184949.317	2580949.188	2016809.3
Com_10280_pos	Cer-NDS (d18:0/18:0)	--	C36 H73 N O3	567.55829	10.637	568.56531	7911250.322	392550.3019	375612.3726	287097.7423	1116140.933	351083.7708	1130668.78	1148814.972	1027986.1	2399666.069	649431.465	951692.6177	607926.1294	653733.1379	634476.3714
Com_10356_pos	TKK	--	C16 H33 N5 O5	375.25127	6.504	376.25854	3013919.306	2987222.199	869300.0232	1735218.013	2424093.036	2446038.725	5106589.213	1701618.736	1324604.053	3040775.358	819826.8061	1053266.704	1751924.288	1324334.203	1327497.906
Com_10409_pos	5,8-dihydroxy-10-methyl-5,8,9,10-tetrahydro-2H-oxecin-2-one	--	C10 H14 O4	220.07088	6.061	221.07816	1073700.695	1664709.724	1160392.101	1476399.288	1136197.293	1319845.571	2959273.344	3591958.195	4746251.203	4907755	630193.0346	4820404.704	1049774.197	957543.0923	1086622.203
Com_10451_pos	2-Deoxyuridine	Deoxyuridine; 2-Deoxyuridine; 2'-	C9 H12 N2 O5	228.07427	1.352	229.08159	4972664.181	7109865.512	6595386.122	6503582.863	7324708.928	7543271.876	1545497.199	1669988.325	1733181.685	1500880.273	3586347.917	2404387.995	4017877.376	3022332.218	3857284.418
Com_10466_pos	8(9)-EET Ethanolamide	--	C22 H37 N O3	385.26155	5.414	386.26883	571859.1109	635835.7912	640719.9929	561298.9188	512109.3459	628712.5797	691099.1962	182636.4632	401862.9306	633245.8344	4989651.1	174867.3398	701690.7918	755020.6021	623060.6685
Com_10488_pos	Azilsartan	--	C25 H20 N4 O5	478.12567	5.813	479.1329	1470081.533	2713390.603	3300414.121	2893965.831	2583028.505	2083308.607	4978128.894	3948502.772	3719800.641	4603541.526	892613.9931	4347227.628	2506208.467	2710916.887	2904910.022
Com_10514_pos	Clinafloxacin	--	C17 H17 Cl F N3 O3	347.08235	1.269	348.08963	3578529.829	4608860.699	1964715.841	1892844.747	4743280.322	3885727.633	114807.9732	616970.142	559156.1115	4810086.95	243203.3338	652619.1734	2062421.352	2326043.482	2064039.136
Com_10548_pos	SPK	--	C14 H26 N4 O5	330.19008	4.992	331.19736	2298200.187	1383946.81	1206518.077	1156412.105	1733680.847	1285319.027	515196.5669	1162000.958	2025630.463	4772480.334	675586.2351	983007.9908	1313924.282	1299011.049	1091694.771
Com_10555_pos	3-ethyl-4-hydroxy-1-methyl-1,2-dihydroquinolin-2-one	--	C12 H13 N O2	203.09466	4.852	204.10188	2207471.871	1461000.307	1294404.58	2088885.459	4908017.093	1661192.421	4911156.528	533394.344	1081485.337	884515.8042	321607.3446	506047.7856	1710463.853	1819796.27	1456413.754
Com_10557_pos	3-pentadecyl-4,5,6,7-tetrahydrobenzo[d]isoxazol-4-one oxime	--	C22 H38 N2 O2	362.29656	7.936	363.30383	6180140.429	5749254.552	5757109.631	5417526.437	7408173.829	8634486.219	802953.3162	602287.8329	733915.9026	1974358.839	632484.6362	586379.156	2760166.043	3104146.917	1947241.835
Com_10576_pos	D-Glucuronic acid	D-Glucuronic acid	C6 H10 O7	194.04261	0.982	195.05008	6215401.465	6296859.318	3555519.731	4723462.681	5059426.332	6501868.474	2043459.688	1764659.449	2738376.015	2281330.009	1574597.402	1413837.401	4475453.086	3453288.66	2677246.9
Com_10667_pos	3-[4-(4-methoxyphenyl)-1,3-thiazol-2-yl]-5-methylisoxazole	--	C14 H12 N2 O2 S	272.05731	5.314	273.06445	957078.9688	981859.0224	894455.7234	834063.1169	806560.6573	880233.5025	3101138.352	3492921.379	3858113.68	2597007.55	4785127.654	3167826.675	1635526.679	1326154.994	1185791.948
Com_10837_pos	6-methyl-7-nitro-2,3-dihydro-1,4-benzodioxine	--	C9 H9 N O4	195.05324	5.412	196.0605	5387860.197	7955885.94	5902925.113	5737016.819	5868973.242	5211335.471	1060485.938	718809.6617	968879.1308	1006456.662	1415276.239	1031460.092	2796985.726	2922558.516	2824213.898
Com_10838_pos	Mezlocillin	Mezlin; Mezlocillin	C21 H25 N5 O8 S2	556.14441	10.812	557.15155	7056988.513	125408.3399	95986.60495	116977.621	1127462.448	7407990.545	207164.3429	159314.5056	167711.151	3700212.157	120352.2264	126994.0471	857094.8796	891786.0135	849058.8586
Com_10851_pos	5-hydroxy-2-phenyl-4H-chromen-4-one	--	C15 H10 O3	238.06276	6.563	239.07004	578529.6093	715148.8251	853039.7902	626422.2243	714002.8702	721818.3708	4619199.85	1902386.661	952556.9103	1743243.091	669156.0426	1859734.278	935929.772	1041908.061	1260499.527
Com_10863_pos	7-[(3,3-dimethyloxiran-2-yl)methoxy]-6-methoxy-2H-chromen-2-one	--	C15 H16 O5	276.09963	5.358	277.10654	2523039.542	2986656.859	2320457.87	2082206.768	2441418.32	3040234.758	1989415.686	3888388.934	2731709.241	3405523.415	2078334.358	3182918.36	1762052.967	1567071.283	1259268.43
Com_10879_pos	2-(2,4-dihydroxyphenyl)-3,5,7-trihydroxy-4H-chromen-4-one	--	C15 H10 O7	302.04547	5.319	303.05228	460138.6994	472052.3053	430031.0703	400995.8743	387773.4063	423193.396	3655921.722	3869130.056	4246274.462	2975968.952	3409859.062	3650273.909	1314336.168	1373638.518	1125218.993
Com_11044_pos	AL 8810 Methyl ester	--	C25 H33 F O4	451.27412	4.958	452.28015	281621.1756	283541.9283	253038.4827	253739.5615	296403.3212	292311.2804	488034.3224	121574.0756	3732517.237	3271105.459	4421578.742	126680.2455	724004.2344	1005754.651	726875.3551
Com_11072_pos	N-Palmitoyl taurine	--	C18 H37 N O4 S	363.24079	4.881	364.24808	204173.4355	213261.9224	189373.0705	187818.1301	213819.0865	209271.0398	902922.3239	110302.0342	1543596.457	1958294.578	4403783.97	107952.3297	572164.1848	636650.2831	726180.2332
Com_11126_pos	Leukotriene B4 Ethanolamide	--	C22 H37 N O4	401.25629	5.237	402.26358	6648445.483	5493197.807	2628806.585	2700901.924	3496277.181	2859497.619	467433.0535	471881.7206	431263.2337	523040.4054	2034237.447	373790.2831	1923251.191	1764582.701	2135215.209
Com_11138_pos	NNK	4-(N-Nitrosomethylamino)-1-(3-pyridyl)-1-butanone; NNK	C14 H26 N6 O6	396.16838	5.382	397.17548	2026988.712	5135788.904	5715592.806	5648762.914	3667601.603	5514832.265	142378.6166	109728.3962	111927.1837	141082.1101	235558.9406	101158.9319	2274562.503	1939998.703	2419972.972
Com_11185_pos	Methyl N-cyano-N'-[2-(2,2-dichlorocyclopropyl)ethyl]carbamimidothioate	--	C8 H11 Cl2 N3 S	251.00692	1.239	252.01425	2520912.314	6024923.09	1018691.019	1281218.714	1652497.974	4386863.391	644774.1317	2239489.461	916085.3982	4186311.016	241882.72	2942699.719	2722990.788	2654462.021	2892637.801
Com_11209_pos	Glycohyocholic acid Sodium	--	C26 H42 N Na O6	487.29158	9.662	488.29886	457702.2371	358529.6182	472148.038	246780.8711	404560.3746	342184.2463	1779243.502	3423521.661	1334282.396	2160922.946	4278246.132	1692338.461	1351452.277	1267228.992	1172837.452
Com_11241_pos	2-[5-(2-hydroxypropyl)oxolan-2-yl]propanoic acid	--	C10 H18 O4	240.07846	6.494	241.08574	1084773.562	1408722.648	775615.4513	997951.8167	980541.8418	1100101.861	2341132.341	3593451.758	1842581.416	3189465.647	845258.3538	2598738.54	1536343.131	1396379.211	1426898.295
Com_11256_pos	Prostaglandin E2-1-glyceryl ester	--	C23 H38 O7	448.24645	5.822	449.25372	5807684.95	7284794.004	4811993.485	2875226.579	5025942.863	4086070.247	2677107.261	1377702.012	2876747.09	3966880.706	2564919.734	2445153.946	3680801.105	3813313.232	4783376.055
Com_11297_pos	Orotidine	--	C10 H12 N2 O8	288.06038	1.608	289.06766	645796.6205	695262.1703	516929.4554	552349.4109	824283.5899	739536.2801	4207286.813	2412880.753	1446797.004	324784.1971	951510.097	2522527.833	741901.0851	780914.941	808439.4352
Com_11375_pos	Pyrithioxin	--	C16 H20 N2 O4 S2	368.08761	5.351	369.09506	2583334.759	4194061.714	6013360.81	2883094.913	3013256.609	4032007.305	3641460.164	1191117.425	1482813.476	827394.0268	1546909.067	1619203.784	2469436.382	1527569.6	2190365.416
Com_11423_pos	4,5-diphenyl-1,3-oxazole-2-	--	C15 H11 N O S	253.05849	3.863	254.0658	2249613.608	2119450.772	5723364.451	5331839.172	2372907.993	2584183.797	188134.9147	129012.3547	231216.7022	466556.8446	226630.537	204675.8927	1504308.327	1234190.52	1277668.368
Com_11429_pos	Obscurolide A1	--	C15 H17 N O5	313.0982	4.743	314.10541	3941711.642	3329743.481	4978937.907	5326025.53	2873848.292	3219064.163	345628.1307	697924.1191	368203.1319	686441.4258	72360.37674	596910.8579	1527365.535	1479900.711	1563504.807
Com_11446_pos	1-(5,7-dichloro-2,3,4,4a-tetrahydro-1H-xanthen-4-yl)pyrrolidine	--	C17 H19 Cl2 N O	323.0866	4.827	324.09387	5527710.592	4131288.575	3594319.602	3808548.93	6387246.134	4002357.204	109399.2858	99625.40695	113187.2681	152034.749	101958.5561	147147.6873	2144753.378	2245932.333	1851581.562
Com_11461_pos	2-(cyclopropylcarbonyl)-3-(4-fluoroanilino)acrylonitrile	--	C13 H11 F N2 O	268.03812	5.217	269.04547	241829.0366	252809.8306	223618.3705	218093.8245	226105.7508	246282.3215	371634.5559	1578129.157	4460296.959	3780214.15	202887.4496	1476434.929	519225.2583	470014.234	456614.6197
Com_11506_pos	3-hydroxy-N-(1-hydroxy-4-methylpentan-2-yl)-5-oxo-6-phenylhexanamide	--	C18 H27 N O4	303.18234	6.042	304.18994	5729166.781	5063344.186	5864926.72	4330307.174	4492774.349	4859977.771	446717.215	449181.1451	496926.1198	603978.8089	498702.7978	507086.1806	2209453.886	2305839.378	2337550.297
Com_11533_pos	LPH	--	C17 H27 N5 O4	365.20585	5.171	366.2132	5082123.846	5604049.393	5841367.375	3784591.283	5261019.783	6328610.884	201970.2484	182281.5132	285454.6391	326052.4275	204351.62	223263.9077	1678743.046	1746050.408	1489119.707
Com_11654_pos	3-(4-nitrophenyl)[1,2,3]triazolo[1,5-a]quinazolin-5-amine	--	C15 H10 N6 O2	306.085	4.825	307.09222	5895164.286	5411444.441	4266750.89	3260657.613	6130231.278	3835513.732	639185.687	657365.9341	673743.2367	895723.6185	641458.8787	702027.4625	2170291.166	2168741.196	2001352.65
Com_11691_pos	16(R)-HETE	16(R)-HETE; (5Z,8Z,11Z,14Z)-(16R)-16-Hydroxyeicosa-5,8,11,14-tetraenoic acid; (5Z,8Z,11Z,14Z)-(16R)-16-Hydroxyicosa-5,8,11,14-tetraenoic acid	C20 H32 O3	342.21503	5.112	343.22226	3401393.339	3627371.168	3290098.804	5052467.502	3135444.013	5886317.762	163569.6176	94108.84409	109220.0263	132737.17	117505.1383	134003.5268	1357490.074	1541222.127	1424528.947
Com_11707_pos	N1-[2-(2-pyridyl)ethyl]-2-aminobenzamide	--	C14 H15 N3 O	263.1014	2.25	264.10867	4858118.003	5562084.469	3987290.541	4044165.746	5797021.735	6830472.131	707810.7442	721021.8355	914073.5059	1268414.334	871388.5385	806106.7071	2164572.217	1977305.677	2232131.178

Com_11890_pos	Gluconolactone	D-Glucono-1,5-lactone; Gluconic lactone; Gluconic acid lactone; 1,5-Gluconolactone; delta-Gluconolactone; D-Gluconolactone; Gluconolactone	C6 H10 O6	178.04772	5.485	179.05516	2930983.02	2479319.43	2174742.195	2468617.287	2775701.313	3776263.592	607442.0657	2327385.08	645303.3351	3617121.592	639617.3129	3862689.389	1903713.053	1745651.784	1081196.945
Com_12042_pos	10-Nitrolinoleate	--	C18 H31 N O4	307.2206	8.518	308.22787	2835291.864	3841464.211	1652783.028	1454396.448	2688810.256	3652135.969	1436817.216	1661178.507	3991149.845	1677108.017	1062471.668	2545372.05	2590723.897	2468911.695	2312740.477
Com_12057_pos	Lysopg 18:1	--	C24 H47 O9 P	510.29756	7.087	511.30484	1214412.185	1146395.123	1630520.294	1302674.311	1839279.825	2372164.022	2846060.831	3079586.207	2688520.839	3371512.192	3349502.427	2318051.171	2077240.498	1867305.197	1783237.776
Com_12165_pos	Lysope 16:0	--	C21 H44 N O7 P	453.28505	8.936	454.29202	5419892.302	2945923.301	2563280.607	1164150.681	2805579.483	1719478.037	318625.4197	363343.9648	415838.7225	495639.1143	265203.4657	319622.9235	623970.4944	789618.8764	752637.6249
Com_12296_pos	acetyl phosphate	Acetyl phosphate	C2 H5 O5 P	139.98703	1.174	140.99446	3322733.006	5939320.363	1048369.364	1702369.209	832777.4947	1202446.189	229091.8021	151899.0824	176578.8831	310923.9509	89891.10396	262050.6314	779651.1464	608816.4336	856086.3105
Com_12306_pos	5-fluoro AB-PINACA N-(4-hydroxypentyl) metabolite	--	C18 H25 F N4 O3	386.18035	4.947	387.18762	2954621.351	2340169.737	2137458.901	4475451.465	2327647.806	2245511.714	151424.4112	297799.9002	164501.892	2514198.85	321778.7612	135854.8888	1164060.927	1561421.431	1213506.101
Com_12377_pos	3,14-dihydro-15-keto-tetranor Prostaglandin E2	--	C16 H26 O5	320.15947	7.765	321.16675	539307.0739	214754.8656	173159.914	187336.2761	211503.1063	216964.6075	2071606.529	2865755.98	586277.1487	330337.5484	443082.3234	2012750.265	455211.2206	613900.6136	584613.6088
Com_12544_pos	Val-Ser	--	C8 H16 N2 O4	204.11065	1.218	205.1179	3638775.126	2942848.62	3638352.121	2830209.065	5121969.515	3837045.221	496818.74	1082312.501	1781070.894	1325798.569	1479855.489	2223658.636	2472607.12	2280359.073	2350319.199
Com_12568_pos	Uracil 1-beta-D-arabinofuranoside	--	C9 H12 N2 O6	244.06911	1.384	245.07639	2800423.15	3766687.524	4228546.947	3417041.765	5095157.367	4755346.656	677542.3521	2323039.717	3454514.791	1438764.609	1676901.066	2997951.449	2526173.892	3040483.238	2316941.804
Com_12590_pos	Irganox 259	--	C40 H62 O6	660.44491	9.031	661.45294	2846674.11	4033520.987	2361936.33	4219612.37	4500169.155	2613042.874	1063006.736	1380415.733	1398585.699	1630489.36	932888.8087	1866739.527	1488496.461	2473564.44	1899028.318
Com_12608_pos	acridine-9(10H)-thione	--	C13 H9 N S	211.04534	1.893	212.05261	324457.6334	1467610.433	359286.6973	293771.6749	1730430.551	1808138.868	2336836.585	1456736.854	1624413.595	758554.3329	1214156.667	3350482.193	1090819.052	673791.1556	701620.7405
Com_12810_pos	Citrinin	Citrinin	C13 H14 O5	272.06645	5.375	273.07373	371640.7416	1411377.272	325779.2769	300823.7302	306030.2677	420559.4324	1019265.714	2624513.147	3159735.534	2430240.025	973890.6767	2971942.498	932932.0588	1533324.581	1049705.139
Com_12902_pos	LDGTS 20:0	--	C30 H59 N O6	529.43406	10.29	530.44116	127437.2731	142288.4561	124176.7303	112643.6335	139145.8208	132015.5766	938770.7765	758174.3403	416252.8653	914655.0874	3045670.622	956144.7017	404408.6193	526196.3019	398612.0591
Com_12919_pos	Phe-Phe	--	C18 H20 N2 O3	312.14702	5.305	313.1543	3067716.784	2088635.616	2550474.278	3233553.404	3531552.697	2874188.198	997665.5111	2563106.414	1152663.746	725883.5739	201201.5626	1040321.279	1570340.824	1616373.658	1641687.505
Com_13051_pos	Cortisol	Cortisol; Hydrocortisone; 11beta,17alpha,21-Trihydroxy-4-pregnene-3,20-dione; Kendall's compound F; Reichstein's substance M	C21 H30 O5	724.4135	7.269	725.42078	644792.1777	783254.2643	717453.0286	1064546.928	624508.7917	857911.1092	2598177.026	2497857.647	1955053.078	641880.111	2551627.584	2357920.975	886665.968	1414179.811	839238.0728
Com_13077_pos	PC (14:1e/3:0)	--	C25 H50 N O7 P	507.33223	9.23	508.33948	2296414.677	1443557.624	4267781.274	1468069.142	3946252.626	2467985.578	253064.0732	268791.7242	226086.7448	329217.1832	324293.3495	342237.8579	992652.4851	1231909.126	1338247.482
Com_13099_pos	DL-Panthenol	--	C9 H19 N O4	205.13199	5.955	206.13889	2299073.412	5032975.576	1360352.536	1472297.013	2009155.082	2915362.356	393844.5838	459357.662	535753.4119	575600.6527	490629.2809	441844.0124	1458696.499	1222093.997	1081517.11
Com_13125_pos	Choline bitartrate	--	C9 H19 N O7	253.11632	1.352	254.12399	2517255.262	3495869.348	3526481.715	3785230.357	3802660.879	3710784.323	1078829.397	1027547.149	1041031.67	1227546.23	1521968.784	1395344.828	1983509.059	2733836.734	2444011.288
Com_13186_pos	L-(-)-alpha-Amino-epsilon-Caprolactam	--	C6 H12 N2 O	128.09492	1.348	129.10213	3509885.912	4955201.523	1601312.253	1941390.472	3111218.698	2979459.589	698746.9239	868503.8837	861249.5131	719262.3054	1627094.739	1138649.209	1563488.133	1528297.734	1601189.168
Com_13323_pos	P-Coumaroyl Agmatine	--	C14 H20 N4 O2	276.15848	4.961	277.16559	296080.0684	278131.3827	197994.0595	186682.4542	236655.8507	229171.952	1428279.929	949030.0812	721788.1842	905197.2828	2802492.393	840070.7367	472818.5874	653013.6274	513222.3961
Com_13330_pos	GPH	--	C13 H19 N5 O4	347.10382	5.072	348.1109	2078526.764	1301519.842	2049780.23	2329774.003	3031224.218	2528309.357	762901.209	1733081.694	605465.5505	1010634.769	359730.8338	2894536.386	866018.6308	1694779.179	1553968.408
Com_13446_pos	Cholesterol	Cholesterol; Cholest-5-en-3beta-ol	C27 H46 O	386.35445	10.518	387.36172	1093891.351	734322.7107	1246892.912	756984.4034	3093001.554	603074.3608	1466231.522	2313409.98	621799.4794	1033077.379	980045.2119	1404497.16	1338359.832	1272992.419	1495036.775
Com_13530_pos	N1-pyrazin-2-yl-4-chlorobenzamide	--	C11 H8 Cl N3 O	215.0252	1.883	216.03261	101544.0181	129629.3488	72398.25716	156844.2105	126488.0578	98471.98945	695500.6663	1676374.085	2953568.307	2220037.676	773260.921	2010235.183	740136.279	487293.0719	603966.1901
Com_13601_pos	Quinic acid	Quinate; Quinic acid; Kinic acid; Chinic acid; L-Quinic acid; L-Quinate; (-)-Quinic acid	C7 H12 O6	192.06331	1.364	193.07051	2309681.623	2192794.448	1967795.039	3045534.341	3005037.562	2233749.241	2672894.353	1668220.352	1947226.715	2462194.024	1441565.917	1998636.453	2236539.819	1829294.754	1900133.134
Com_13621_pos	N-{[(2R,4S,5R)-5-Ethyl-1-azabicyclo[2.2.2]oct-2-yl]methyl}-2-furamide	--	C15 H22 N2 O2	244.15703	1.866	245.16431	81900.96025	119909.7492	71159.98289	72488.48205	103118.27	102793.0553	437323.4168	49369.01687	65742.07805	59183.8765	2661161.695	59150.01948	292656.4178	137551.0156	219744.2801
Com_13742_pos	Gly-Phe	--	C11 H14 N2 O3	222.10042	4.908	223.10774	3123959.165	3719351.354	2874436.685	3368685.074	4049324.45	3934647.361	355784.3347	1182582.758	568326.4491	1021148.484	518927.8505	366429.2087	1941074.503	1778027.397	1680351.541
Com_13956_pos	N'-[6-(tert-butyl)thieno[3,2-d]pyrimidin-4-yl]-4-methylbenzohydrazide	--	C18 H20 N4 O S	362.11901	4.85	363.12628	108461.3157	118474.1346	104147.5627	91578.33657	108129.8752	107941.9039	67433.44826	61408.85362	74074.24884	2416539.192	359693.5549	63701.61706	174945.669	194419.5795	220800.9697
Com_14038_pos	4-Hexyloxyaniline	--	C12 H19 N O	193.1465	1.428	194.15373	148105.6744	196087.9686	125878.7016	151133.9427	217725.2857	230077.0635	1055652.86	1746215.587	472001.0646	714326.5037	1456995.552	2523461.71	642380.6428	536841.1913	555497.633
Com_14397_pos	2-(2-acetyl-3,5-dihydroxyphenyl)acetic acid	--	C10 H10 O5	232.07303	5.614	233.04408	1403370.218	1602337.414	1804824.839	1024238.148	1558599.522	1584215.744	928547.4155	1910841.044	1928001.046	1832065.504	1489440.639	1916678.491	1018237.145	1109332.368	1366697.764
Com_14461_pos	Triacanthine	Triacanthine	C10 H13 N5	203.11693	5.588	204.12421	1427593.682	994694.5748	1152055.372	1204740.883	1632414.786	1707736.815	213286.4354	1882202.859	770746.9619	1947079.591	499379.2383	1518001.563	1423934.995	940762.7149	881252.125
Com_14482_pos	Ala-Trp	--	C14 H17 N3 O3	275.12703	5.045	276.13434	3095518.515	2491674.307	2040725.601	2017349.733	3279556.193	2619219.716	465384.5798	795775.2944	1347595.794	2149660.776	890537.8271	723013.5625	1807457.432	1050159.03	1375834.728
Com_14522_pos	3-amino-2,6-diphenyl-4,7-dihydro-2H-pyrazolo[3,4-d]pyrimidin-4-one	--	C17 H13 N5 O	303.11394	4.169	304.12122	1792754.222	1549906.749	2455656.753	2844448.98	1379121.976	1691778.235	51900.50645	68468.15226	121519.8526	293304.519	43789.88574	61575.86802	410354.5505	310660.3606	282024.6763
Com_14813_pos	Lysope 18:1	--	C23 H46 N O7 P	479.30129	9.146	480.30856	433634.5204	837138.2011	2969546.257	195550.1595	1055463.927	930069.2602	327723.2399	413378.1985	339424.945	651694.5826	529021.0675	371072.7697	360177.9384	514642.1921	295250.2141
Com_14835_pos	Tomatidine	Tomatidine	C27 H45 N O2	393.36015	9.383	416.34937	1179227.513	2357063.818	2952838.479	1750235.565	1789854.323	2059458.329	1010746.193	963038.361	1018021.843	755958.9608	752352.0293	1201559.239	892010.1547	891843.835	931564.7518
Com_14848_pos	2-[2-(1-isobutylcyclohexyl)-1-methylethylidene]hydrazine-1-carboxamide	--	C14 H27 N3 O	275.19963	4.919	276.20691	202588.4432	206633.2991	186284.4771	184455.4043	214827.2326	195525.4757	612397.9171	103057.0581	146205.8618	167535.4776	2028079.489	100861.6894	286864.3362	246432.303	195160.6898
Com_14939_pos	1-adamantyl(piperidino)methano	--	C16 H25 N O	247.19335	7.875	248.20062	2567462.459	2104001.703	1370918.548	906537.7285	1540666.459	2316402.72	831685.2509	823497.3674	1003633.067	1184281.382	743277.6219	1360173.062	1824855.058	1133229.107	999253.966
Com_15156_pos	Thymidine 3,5-cyclic monophosphate	--	C10 H13 N2 O7 P	304.04543	5.307	305.0528	400175.4281	410536.5047	373991.294	348739.8383	337240.4648	368044.6757	1272257.462	1596266.46	2002639.424	1644099.947	1746877.247	1611010.072	587936.1012	456783.4976	311596.5968
Com_15188_pos	Tyrosylalanine	--	C12 H16 N2 O4	252.10924	4.64	253.1165	2860997.383	1910258.988	684854.5077	1267024.044	748730.0938	538016.728	93797.22628	82960.05045	103912.0225	105825.6047	128607.2027	90109.32037	476320.3209	444622.1297	485306.2184
Com_15208_pos	EQH	--	C16 H24 N6 O7	394.16054	5.625	395.16782	2442539.931	3153532.731	2530255.195	2227808.712	2598661.044	2626075.864	1437632.334	1025341.294	1249100.725	1413590.763	1075743.238	1208623.561	1709998.853	1885289.106	1522642.474
Com_15404_pos	Fenvalerate	Fenvalerate; Pydrin	C25 H22 Cl N O3	436.1483	5.202	437.15558	2556109.263	3054322.092	2276138.663	3729849.393	2277771.441	2143929.478	1003974.61	1040096.546	1237014.449	1738815.254	1192875.323	1302025.75	1600387.831	1564062.331	1713376.489
Com_15407_pos	Melatonin	Melatonin; N-Acetyl-5-methoxytryptamine	C13 H16 N2 O2	232.1203	5.309	233.12758	1522819.186	2021913.507	2596987.734	1669309.576	1683149.451	1911217.4	616588.7324	437268.8175	222121.845	378036.1584	949936.0549	483629.4594	1610033.15	1082172.804	1105283.791
Com_15434_pos	N1-[2-oxo-6-(1H-pyrrol-1-yl)-2H-chromen-3-yl]acetamide	--	C15 H12 N2 O3	268.08458	6.397	269.09189	2104092.026	2818327.772	2462741.484	2295617.954	2779314.575	3011042.195	564225.625	177815.5202	218183.4783	277145.9549	483753.7068	185198.7782	963534.1678	1190746.166	1223406.976
Com_15462_pos	N-{[5-(tert-butyl)-2-thienyl]carbonyl}-N'-(6-methyl-2-pyridyl)urea	--	C16 H19 N3 O2 S	317.12183	1.435	318.12924	1832016.177	1933974.665	1708008.676	2294175.484	2567301.691	2206530.866	103151.7843	102686.5986	103949.7616	684978.3559	254235.1973	147381.4695	631718.4216	823525.5154	974492.9241
Com_15679_pos	Kaempferitrin	Kaempferitrin; Kaempferol-dirhamnoside; Lespenefril	C27 H30 O14	278.09682	5.568	579.18286	1461623.336	1950483.376	2428511.983	1771800.698	1423944.382	2292287.941	508877.9087	470524.6757	649121.3443	530208.3629	321888.2504	559293.7305	1264948.538	1058266.929	1326891.54
Com_16061_pos	N-Acetylglucosamine 1-phosphate	--	C8 H16 N O9 P	301.0563	1.498	302.06354	459336.0579	746766.57	735257.651	487875.4724	1001472.478	1180099.425	170491.3641	331360.2011	134631.7204	1466873.971	462301.4906	262289.4521	532092.3107	648900.553	696770.5709
Com_16197_pos	Pleuromutilin	Pleuromutilin; Pleuromulin	C22 H34 O5	416.19598	5.842	417.20319	1552094.102	564891.0919	721307.0542	1409178.143	2278686.908	1610766.609	214147.2883	259460.7383	277503.2063	245780.2833	439794.7676	142175.6763	416696.167	566396.4567	299855.995
Com_16205_pos	WKK	--	C23 H36 N6 O4	230.13751	2.002	231.14479	2113781.215	1912273.763	780906.9714	1127638.442	1690607.657	2000208.529	90533.90219	205200.4815	761463.9376	1414649.153	201072.1988	270133.6819	753876.3928	651921.6653	888794.8713
Com_16389_pos	4-Hydroxy-3-methylbenzoic	--	C8 H8 O3	152.04661	5.064	322.12704	671557.5293	163017.2144	877926.4656	149583.4928	793166.4414	1202536.096	1382631.406	738562.342	794253.3382	437434.0534	458861.2168	1098015.527	698615.1814	631758.5385	674298.0558
Com_16497_pos	N-(4-butyl-2-methylphenyl)-N'-[4-(4-methylpiperazino)phenyl]urea	--	C23 H32 N4 O	380.25374	7.159	381.26102	817316.1024	1032689.05	758393.0538	879177.7236	1431675.503	884981.2217	829743.3244	990148.6355	1377602.734	1304593.926	977149.3324	984247.3266	1161812.352	1022723.524	861950.8278
Com_16743_pos	2-Aminobenzenesulfonamide	--	C6 H8 N2 O2 S	172.03113	1.299	362.09576	112638.1775	47123.22281	157080.4559	166553.35	198370.8655	557432.019	193579.7571	491124.4118	660067.1093	287832.2576	1247992.088	753022.456	380091.1586	406151.7503	459740.595
Com_17085_pos	VLK	--	C17 H34 N4 O4	358.25774	4.817	359.26474	1380618.046	1171300.85	590449.6216	558620.2771	1740711.519	1474725.268	75294.38205	157186.3711	224364.2624	225459.5257	86306.52953	151922.7005	534147.9203	520773.6278	518907.1191
Com_17254_pos	Cystine	Cystine; Dicysteine; alpha-Diamino-beta-dithiolactic	C6 H12 N2 O4 S2	240.02455	1.319	241.03183	93931.08734	65486.04389	61687.4587	73321.25714	1631051.778	308198.7264	49364.76014	52175.57934	47763.15389	46871.25102	67256.27786	71592.07255	97488.24011	119269.8859	139693.8788
Com_17266_pos	N1-(3-pyridyl)-2,3,4,5,6-pentamethylbenzene-1-sulfonamide	--	C16 H20 N2 O2 S	304.11833	1.226	305.12561	179730.6837	599412.7163	863935.1753	1348908.754	1002201.025	525671.0596	56998.45264	101087.0983	74838.87615	69621.25982	710503.4626	92225.27156	368603.2022	384180.548	392613.3937
Com_17376_pos	(2R,3S,4S,5R,6S)-2-(hydroxymethyl)-6-phenoxyoxane-3,4,5-triol	--	C12 H16 O6	273.12074	6.005	274.12802	1516943.299	1184396.872	1100757.272	1041801.734	965582.927	1069469.824	393258.7246	303935.3564	336241.6677	505645.2194	334725.6861	314879.9658	650803.1626	650577.4333	612547.5159
Com_17786_pos	2-({2-oxo-2-[(2-oxo-3-azepanyl)amino]ethyl}sulfanyl)acetic acid	--	C10 H16 N2 O4 S	260.08718	4.61	261.09445	1234584.439	1061675.765	1037781.634	908084.8099	1248266.246	1131681.042	733820.5204	113823.6332	570422.4993	700100.9589	91957.33229	81454.32769	462395.6001	564442.104	466529.9784
Com_17973_pos	Lysopc 20:4	--	C28 H50 N O7 P	543.33391	8.268	544.34119	121761.067	164634.8358	200674.1352	222651.6333	92920.42283	112952.5653	734024.396	604282.8712	179417.2797	181608.4545	136714.1236	109764.579	120156.972	120034.0968	118138.9035
Com_18102_pos	5'-Deoxy-5'-(Methylthio)Adenosine	--	C11 H15 N5 O3 S	297.08968	5.04	298.09695	803907.3138	909939.7308	973269.9517	717043.3285	665467.3148	888104.074	254615.0608	145885.8548	175575.98	153362.9908	152579.4583	235733.8706	472376.2432	476125.3291	418788.1714
Com_18112_pos	Guanidineacetic acid	--	C3 H7 N3 O2	117.0539	1.345	118.06118	186550.5067	175697.7879	127048.1203	163959.7309	216558.1195	222410.6422	185708.1275	359348.6988	397322.1828	104812.6833	233295.173	689363.3371	166229.7524	188330.2956	163194.6083
Com_5_pos	7-Ketolithocholic acid	--	C24 H38 O4	390.27636	7.451	391.2836	3321101978	4102197863	9859221428	11981816031	4310284006	6130270905	4234241107	2871965890	5803864786	8974682468	8023032376	3430482478	6121583003	5783921475	6018080811
Com_39_pos	Stercobilin	L-Urobilin; L-Stercobilin; Stercobilin	C33 H46 N4 O6	594.34113	5.46	595.34833	2860625310	3298814774	3902689546	3661199921	2752296172	3081085721	87276681.75	172341042.9	141876660.2	429053665.1	161285421.3	138151908.8	1480513820	1463953871	1523326572
Com_102_pos	MAG (18:3)	--	C21 H36 O4	352.26093	9.075	353.26807	66487752.58	167502076.7	66850462.25	40580532.83	86680119.71	96184589.43	567594554.5	858013463.8	406989138.4	97412314.15	318133988.9	627135691.3	357172757.5	336194420.4	314336622.7
Com_132_pos	Phenylpropiolic acid	--	C9 H6 O2	146.03741	1.037	147.04469	939868373.6	1048586998	823691127	751002453.8	903079874.6	717507534.8	199684549.9	179639070.3	273460786.4	396941876.3	228644717.4	186129436.3	761218683.2	706553393.5	452441707.2
Com_211_pos	Ergosta-5,7,9(11),22-Tetraen-3-beta-Ol	--	C28 H42 O	394.32318	9.519	395.33041	137590807.7	158069059.3	50586903.7	60422275.22	893928099.6	681850567.9	117605067.9	87830229.07	76489154.11	110867258.7	444150873.6	94356063.78	176120134.4	146381814.4	183536419.9
Com_223_pos	MAG (18:2)	--	C21 H38 O4	354.27651	8.988	355.28378	313453764.8	2380348.5	580634109.6	707885585.9	559406708.2	401582902.4	170838885.6	167382276	203146533.9	258467646.1	193577714.2	130127372.8	241036315.9	243198165.5	237411700.5
Com_282_pos	(+/-)12(13)-DiHOME	--	C18 H34 O4	296.23486	8.414	297.24216	467003655.8	679636630.2	592259725.4	565285810	660457097.6	763777297.1	382827996.6	231787738.7	322728180.9	240643984.9	332497456.2	310225259.3	385322427.5	388561966.1	354615392.2
Com_349_pos	7-Ketocholesterol	--	C27 H44 O2	400.33372	8.583	401.34103	64418984.64	91233458.65	108819096.1	139254273.6	86735517.04	94130527.74	50376734.5	57300637.76	150052361.9	349576644.3	47593554.9	71122212.4	125610013	127575357.2	103135113.7
Com_393_pos	Prostaglandin F2alpha	Prostaglandin F2alpha; (5Z,13E)-(15S)-9alpha,11alpha,15-Trihydroxyprosta-5,13-dienoate; (+)-Prostaglandin F2a; 7-[3,5-Dihydroxy-2-(3-hydroxy-1-octenyl)cyclopentyl]-5-heptenoic acid; 9a,11a-PGF2; Amoglandin; Cyclosin; Dinoprost; Enzaprost; Enzaprost F; Panacelan; PGF2a; 9,11,15-Trihydroxy-(5Z,9a,11a,13E,15S)-prosta-5,13-dien-1-oic acid; Prostaglandin F2a; Prostin F 2 alpha; Protamodin; U 14583; (5Z,9alpha,11alpha,13E,15S)-9,11,15-Trihydroxyprosta	C20 H34 O5	354.24021	6.9	355.24759	288006612.9	219505035.7	101294147.2	167831328.9	148251528.4	177570390.1	45489266.97	26361533.04	30063890.24	35911385.62	320385246.5	41137890.09	79987227.44	70627469.99	69692828.94
Com_549_pos	Adenine	Adenine; 6-Aminopurine	C5 H5 N5	135.05446	2.573	136.06174	316312209.4	347331065.9	340896344.3	191387892.5	83485671.34	141905693.6	16878652.96	39557040.69	26156685.44	3659213.027	6552435.858	38421472.96	79723845.82	72088741.04	71184765.68
Com_605_pos	Linoleoyl ethanolamide	--	C20 H37 N O2	323.28207	9.254	324.28934	30660451.5	51467600.63	29225602.19	13657373.94	78558996.02	30679124.08	102147430.9	180682288.2	158851269.4	109668015.9	156224829.7	135221005.7	89921711.66	97234775.24	93825828.25
Com_708_pos	Oleanolic acid	Oleanolic acid; 3beta-Hydroxyolean-12-en-28-oic acid; Astrantiagenin C; Caryophyllin; Oleanolate	C30 H48 O3	478.34198	9.035	479.34924	52751266.63	60030169.44	21605379.92	38613772.57	70485440.26	60347493.54	148410400.5	127063846.9	196429840.7	122960112.5	87544207.39	139886887.7	165742949.4	158969015.8	167496200.1
Com_808_pos	Theophylline	Theophylline; 1,3-Dimethylxanthine	C7 H8 N4 O2	180.06342	5.481	181.07068	189047633.6	198195545.5	130686051.1	204241297.9	184188793.3	209872356.6	1086544.199	154544.5249	154825.0084	193897.8546	1729851.91	161730.8631	77566443.2	87488843.07	72360424.36
Com_1152_pos	Kynurenic acid O-hexside	--	C16 H17 N O8	351.0949	5.701	352.10217	128897957.6	132205939.3	109990379.7	110116201.6	123502175	144198765.3	101684559.3	84250969.29	110060747.2	86944743.47	83682699.97	87492676.09	98723406.01	93673033.15	92650274.89
Com_1207_pos	Lithocholic acid	Lithocholic acid; Lithocholate; 3alpha-Hydroxy-5beta-cholanate; 3alpha-Hydroxy-5beta-	C24 H40 O3	358.28697	9.004	359.29446	32860865.81	50012524.92	72245661.26	82326270.86	54116031.37	70315653.1	42066232.44	28667174.52	93812102.51	99860701.79	36317910.76	52777877.93	48226581.81	56961549.03	55360730.3
Com_1324_pos	Isophorone	Isophorone; 1,1,3-Trimethyl-3-cyclohexene-5-one	C9 H14 O	138.10443	6.171	139.11168	141947334.4	127185628.9	81109346.23	86198844.91	103299794.5	96110363.24	69952367.14	42447384.74	61272479	83399927.97	56854496.21	49750684.79	81608461.16	76345598.57	75478733.96
Com_1398_pos	alpha-Benzylsuccinic acid	--	C11 H12 O4	208.07343	5.716	209.08061	127005312.2	113927930.8	128483341.4	101120927.1	115241702	110185603.8	45872851.1	37664236.74	40508246.14	36222372.6	30875920.22	42950025.24	67731254.74	73910101.66	67369253.32
Com_1444_pos	8-Hydroxyguanosine	--	C10 H13 N5 O6	299.08733	5.631	300.0946	98998113.75	106134348.1	102084649.7	110669023.7	99924494.14	109070070.9	16987137.1	12953987.17	13296179.97	15755666.99	9400391.323	13986304.35	58030032.39	59179673.74	57366916.87
Com_1470_pos	Nor-9-carboxy-δ9-THC	--	C21 H28 O4	344.19835	7.806	345.20581	2306765.449	2453162.226	1920238.246	2122038.096	2077770.866	1744872.55	64843158.01	12498049.04	37074842.03	81335267.19	4590773.68	12494957.4	20315843.06	21725790.83	20950151.34
Com_1471_pos	o-Cresol	o-Cresol; 2-Hydroxytoluene; o-Methylphenol	C7 H8 O	108.05747	5.694	109.06506	120901822.6	113657587.7	105248743.2	103199344.4	93450887.51	104935810.7	51211520.29	37891100	50754941.94	55675462.36	42259461.12	40240845.92	76639841.93	65826399.37	62658544.42
Com_1494_pos	Methyl EudesMate	--	C11 H14 O5	226.08388	5.702	227.09134	111065050.9	110935593	119533346.9	93365851.19	95135727.28	105502366.1	37361678.16	30254455.04	30256563.85	29372879.97	27219850.02	41331254.93	64928853.26	62191229.79	56190999.02
Com_1519_pos	Glycerol 1-hexadecanoate	--	C19 H38 O4	330.27669	8.937	331.28372	18710300.98	14844075.85	10053392.16	16707923.81	13698875.18	14626573.65	31912456.32	60513795.45	87828041.51	61729013.77	49822809.35	57985646.08	38678036.32	38356789.93	36278690.99
Com_1956_pos	Traumatic acid	Traumatic acid; (2E)-Dodecenedioic acid; Dodec-2-enedioic acid	C12 H20 O4	228.13608	5.909	229.14333	89295011.83	94719882.69	81854172.45	62504085.23	48602666.76	70512778.81	31134710.22	30528858.57	51621769.43	57787895.23	37157675.26	33752709.54	53853135.69	67445947.85	49206485.85
Com_1999_pos	Formononetin	Formononetin	C16 H12 O4	268.07316	7.158	269.08044	794295.8961	600567.2805	858389.8464	1121581.664	874352.4965	764218.1518	58478071.81	7037750.504	2641199.704	5009598.055	1315274.965	9524926.025	10501793.15	9264796.619	7463624.667
Com_2005_pos	Oleoyl ethanolamide	--	C20 H39 N O2	325.2977	9.761	326.30499	36796094.41	42490665.5	34003615.5	15504543.79	38882765.57	25101011.2	22839977.6	49153855.68	40838709.03	45865332.78	37566701.13	39709027.23	34833070.68	34310019.29	34763694.27
Com_2204_pos	D-Glucosamine 6-phosphate	D-Glucosamine 6-phosphate; D-Glucosamine phosphate	C6 H14 N O8 P	259.04531	1.49	260.05258	62922581.2	42604585.18	10756506.31	8427842.991	21889712.45	58821981.31	8652186.045	19334562.41	11344751.09	50550825.51	4064888.979	16013725.52	21802185.85	22920868.78	17425779.57
Com_2715_pos	cis-gondoic acid	--	C20 H38 O2	310.28684	9.199	311.29413	2290411.626	2786476.405	3792662.208	5332542.127	1144307.921	1257739.95	24591412.16	14497283.01	24883564.44	39623316.77	19149779.16	13128690.2	14865665.84	15508992.17	15214696.7
Com_2789_pos	5beta-Androstane-3,17-dione	5beta-Androstane-3,17-dione	C19 H28 O2	288.20938	9.731	599.40918	1915432.493	3877589.114	3810864.853	1546540.827	3845785.437	2499823.849	16101537.03	33409752.1	5768972.095	10010871.07	4590469.303	11300225.86	6294219.223	8010382.924	6324110.33
Com_2798_pos	LPE 15:0	--	C20 H42 N O7 P	439.26949	8.88	440.27679	59958506.76	50960252.85	25517814.5	13593223.52	30710423.37	21233917.93	2307228.412	1180348.236	1654525.966	1879321.674	651219.4997	1969386.346	16841818.38	14919070.39	15144666.6
Com_3175_pos	L-Alanyl-L-proline	--	C8 H14 N2 O3	186.09755	1.35	187.10474	8141729.573	10282299.41	7337250.204	6539555.194	8571954.433	7600947.092	4325663.316	10206785.05	14693331.89	20571122.68	33704636.97	12296855.49	10911471.13	9533124.804	9500025.309
Com_3209_pos	Asp-Phe	--	C13 H16 N2 O5	280.10577	4.957	281.11337	15456184.81	17696301.63	15218662.83	12530098.18	17987674.71	18257561.8	6064874.81	7218050.678	26427233.06	32293432.06	4268796.592	6033858.454	16034252.27	13058643.29	12875423.25
Com_3336_pos	Porphobilinogen	Porphobilinogen	C10 H14 N2 O4	226.09541	4.933	227.10271	40802510.2	48897667.12	39666899.65	41080364.36	40461757.55	33315399.51	17153675.29	12243329.74	17839049.13	20774985.63	18060777.23	16199627.81	25628330.71	25251482.01	28146106.69
Com_3384_pos	Biliverdin	Biliverdin; Biliverdin IX	C33 H34 N4 O6	582.2472	6.36	583.25433	21858752.75	20733203.49	39537939.9	40273392.22	32708368.75	39521466.85	14197564.94	12738859.67	13289113.42	22775750.51	8819969.958	11364905.57	19822278.6	20679108.5	19823544.91
Com_3402_pos	Methyltestosterone	Methyltestosterone	C20 H30 O2	302.22432	9.498	303.23157	14493581.5	11253815.57	12878657.7	8317626.093	7911963.068	9902555.638	8023254.437	10747826.02	18183770.01	30039924.21	14212124.96	10468169.7	13232341.12	9572689.877	12363436.75
Com_3420_pos	Anthranilic acid	Anthranilate; Anthranilic acid; o-Aminobenzoic acid; Vitamin L1; 2-	C7 H7 N O2	137.04754	1.352	138.05487	26296912.55	22491727.01	17446971.41	15479360.22	29689851.94	35908144.09	10321555.23	16306872.72	11391238	29815405.01	12110596.11	11571981.37	21924136.1	16113654.21	17872339.7
Com_3806_pos	Trehalose	alpha,alpha-Trehalose; alpha,alpha'-Trehalose; Trehalose	C12 H22 O11	342.11776	5.603	685.24292	714566.5607	725743.7151	632672.2355	607296.7144	695549.0878	703121.1107	26569638.75	7417740.432	7432945.934	12596462.63	2045488.403	4740264.888	3190036.605	3293159.818	3953022.209
Com_3898_pos	Tetrahydroaldosterone	--	C21 H32 O5	364.2246	6.101	365.23181	30914821.06	44215379.33	24304807.49	30687211.57	30457646.06	25179971.4	7377505.281	6864508.425	6895764.918	9526667.311	8481395.545	6668755.822	15005769.08	14796388.02	16579452.98
Com_4145_pos	LPE 18:1	--	C23 H46 N O7 P	479.30078	9.562	480.30804	11821501.22	40283107.73	28761120.96	9540083.458	34404863.18	10870851.98	1276167.418	1129565.74	788255.3252	765912.9006	796782.8064	1107239.898	8991027.831	11427843.62	9859852.566
Com_4155_pos	Dithranol	Dithranol; Anthralin	C14 H10 O3	226.06293	5.372	227.07033	21369714.38	38305341.67	24560966.31	25432703.03	36440104.57	34525574.19	2660060.351	2774695.889	2170320.987	5313149.835	1468360.419	3093571.8	15800212.47	16513761.27	17501712.6
Com_4196_pos	beta-Estradiol 17-Acetate	--	C20 H26 O3	314.1876	7.88	315.19485	4557739.686	4216003.797	4357641.403	4438466.49	5122917.102	6147612.397	8257272.7	7531613.382	13515986.42	13261268.5	23068325.81	7914531.147	9121442.285	9285811.208	8896055.945
Com_4207_pos	Feruloyl Putrescine	--	C14 H20 N2 O3	264.14739	4.952	265.15469	21989923.45	10481773.18	9089701.566	15899518.78	35898157.82	11940358.4	2009584.727	1831260.673	2821280.387	3622200.827	1708672.881	2238654.889	10154663.05	9855217.563	8693184.777
Com_4382_pos	Tyramine	Tyramine; 2-(p-Hydroxyphenyl)ethylamine	C8 H11 N O	137.08406	1.352	138.09108	8590225.722	14520504.94	13997625.54	11075249.67	11501087.07	14169612.55	10011843.77	18460965.87	10353334.51	14318548.01	6068414.468	20088848.93	13895847.32	11842250.29	9912792.77
Com_4764_pos	9,10-Dihome	--	C18 H34 O4	314.24527	6.537	315.25232	5533346.871	3101121.776	3881831.19	2765733.084	3394822.575	2907684.53	13011112.56	10439017.11	17305372.19	18826873.25	14601197.2	10860332.08	7904581.554	6895789.12	6328834.842
Com_4785_pos	LPE 18:2	--	C23 H44 N O7 P	477.285	9.077	478.29224	18412238.84	33180863.8	19907366.11	8729340.589	22828706.15	14859673.92	2339162.187	8336303.925	3854536.445	710482.4615	1342439.026	1302795.241	9889694.978	11024562.92	8886676.205
Com_5141_pos	N-Acetyl-L-tyrosine	--	C11 H13 N O4	223.08455	5.43	224.09187	6966536.953	7669011.377	16669096.2	22654366.74	5036141.564	5780677.365	7023912.681	4810146.217	7480727.214	7853111.042	6684854.071	5945469.55	12916694.89	13371399.27	14290187.67
Com_5740_pos	Stearic acid	Octadecanoic acid; Stearate; Stearic acid	C18 H36 O2	284.27113	8.709	285.27835	9337675.415	8345032.645	6047869.437	6048726.091	10092185.36	7989153.235	3915831.882	8081115.105	3974151.337	14330838.61	3051166.151	6569440.852	6836806.527	7101151.524	6997171.114
Com_5783_pos	N-lactoyl-phenylalanine	--	C12 H15 N O4	237.09965	5.308	238.10706	21644292.03	17885596.73	21115395.54	14458276.18	19296452.18	17623361.62	11034696.49	3220747.083	1364132.216	7127127.66	5769443.96	5325456.368	10635867.98	10541500.49	14152131.29
Com_6112_pos	Gamma-Glu-Leu	--	C11 H20 N2 O5	260.13703	4.891	261.14432	17334389.87	18055322.92	14147571.57	17265106.56	15381076.32	10579153.23	1466626.532	3410163.213	2165933.323	3245019.155	2011233.098	1904275.623	6570307.075	6115975.574	6253493.11
Com_6203_pos	Cytidine	Cytidine	C9 H13 N3 O5	486.17049	1.37	487.17776	80210.44464	100765.5895	1864385.992	380621.3306	4567123.646	2229150.067	143818.5835	6715119.355	6896222.44	221010.645	263810.4008	13462076.63	736473.1729	810084.1098	866398.8413
Com_6218_pos	5,6-Dihydroxyindole-2-Carboxylic Acid	--	C9 H7 N O4	193.03755	5.15	194.04478	16169084.03	14827844.72	13097476.48	14565378.46	14433734.42	12734367.77	10535429.19	7902793.408	11507315.42	12602756.28	11018003.76	9229136.851	11420615.67	9459226.901	9655643.69
Com_6309_pos	LPC 18:3	--	C26 H48 N O7 P	517.31645	8.674	518.3241	10513205.19	7549107.619	14311790.66	5691276.488	19747719.15	15268703.67	7947531.088	5755015.127	6334363.78	4576230.066	5783638.034	6023536.503	7065567.141	8710445.074	6423099.238
Com_6988_pos	LPE 16:1	--	C21 H42 N O7 P	413.31319	8.807	414.31912	16307765.3	15808030.76	11713496	7273701.729	10858298.82	9292595.418	1215031.115	933231.5394	989800.0324	1415724.168	1287123.131	1512962.704	4511818.385	3216340.457	3968380.992
Com_7017_pos	2-Arachidonyl Glycerol ether	--	C23 H40 O3	386.27916	10.012	387.28632	14590065.53	13147032.79	15160582.4	13793888.61	14961525.42	15996768.67	2653111.655	2146659.1	1698073.531	2709771.682	1085301.374	1965072.143	7026698.187	6510504.355	7463621.919
Com_7150_pos	Ala-Gln	--	C8 H15 N3 O4	217.10605	1.362	218.11336	10272834.33	15774677.64	13031163.57	13358819	14486085.48	13240586.91	2718350.734	3075377.869	3157924.248	4916260.05	9652784.968	4028203.7	8931199.029	8021834.54	6720846.211
Com_7516_pos	JNJ-1661010	--	C19 H19 N5 O S	365.1316	1.349	366.13892	7823873	6069690	10646087.88	7899474.751	14656860.59	15430172.07	2206865.055	5858352.776	2264673.661	5915059.46	4404797.207	3720356.515	6059174.122	6534093.438	7410099.756
Com_7651_pos	(5-L-Glutamyl)-L-Amino Acid	--	C8 H14 N2 O5	218.08986	1.361	219.097	8162867.685	9688503.771	9370644.858	11894766.77	12473427.77	12303006.92	1967649.087	2592046.169	1790801.797	2924676.281	7256901.262	3630472.327	5835332.902	5161322.222	4474845.217
Com_7853_pos	L-Leucyl-L-alanine Hydrate	--	C9 H18 N2 O3	202.13146	1.359	203.13875	6635618.071	7081549.869	7015685.954	6535230.799	8208218.707	8592561.453	2386921.655	4062399.793	4376790.482	3335263.652	8713292.961	4928789.214	5954621.474	5471486.907	5429033.813
Com_7858_pos	Cinnamoylglycine	--	C11 H11 N O3	205.07368	5.688	206.08076	9987242.177	8546948.764	9351348.746	11294742.78	11545037.25	9267451.107	3552360.061	4072469.847	3286442.091	3630936.183	2436523.15	3274720.472	6468890.852	6067763.332	5476252.255

Com_8553_pos	Boc-beta-cyano-L-alanine	--	C9 H14 N2 O4	214.09501	1.366	215.10236	7222498.359	10518866.43	8466968.495	8118589.05	10095470.24	9572379.037	2213414.8	2081726.755	2101141.135	1987038.197	7459332.902	2848767.881	5453456.161	4893835.815	5540000.985
Com_8920_pos	4-Hydroxyretinoic Acid	--	C20 H28 O3	316.20378	5.581	317.21106	9489138.387	10090062.71	7397634.677	8685379.047	10806370.59	9077663.914	4185006.078	2883856.612	4454318.184	5717075.877	1589590.071	3442859.29	4506912.085	6045678.397	6132542.817
Com_9435_pos	gamma-Glutamylmethionine	--	C10 H18 N2 O5 S	278.09356	4.794	279.10086	8699010.999	1418706.757	5569917.428	3416035.337	2999786.43	10918944.89	76281.59217	75328.52694	107090.8183	135815.9769	79496.44818	88126.64146	1282453.249	1445174.623	1301991.646
Com_9803_pos	N-Acetyl-DL-phenylalanine	--	C11 H13 N O3	207.08933	5.758	208.09654	7242654.114	8518408.726	8109277.165	6041634.665	6544768.318	7792341.44	5227407.227	3409762.152	5057747.218	4511483.41	5291498.334	5464481.42	5244767.196	5351051.758	5300225.166
Com_10261_pos	Propionyl-L-carnitine	--	C10 H19 N O4	217.13117	4.953	218.13844	7435019.463	7006216.198	6693829.813	6211379.515	8114997.652	7011331.915	1018770.77	1178930.392	1075262.497	1923501.539	1241078.619	1295033.481	3487936.621	4205950.965	2974379.111
Com_10421_pos	4-Oxoretinol	4-Oxoretinol	C20 H28 O2	300.20836	9.752	301.2157	2627444.925	4064039.629	3531716.58	3287218.247	4303782.941	4449843.514	3669775.489	2916726.82	3524081.441	4895677.042	3148004.77	2641207.438	3153170.107	2690137.227	3506700.653
Com_10961_pos	1-Methylguanosine	--	C11 H15 N5 O5	297.10742	4.752	298.11465	5899330.293	3478939.995	4331885.664	4528040.629	7015245.443	7625653.222	655650.565	2382048.877	1690848.882	1286077.72	950919.3239	1927701.206	2629423.176	2795672.074	2525296.649
Com_11033_pos	Hydrocortisone acetate	Cortisol 21-acetate; Hydrocortisone acetate; Cortell	C23 H32 O6	404.21838	5.75	405.2258	6558929.418	7554815.316	2582208.514	5754247.208	4554641.477	6334315.484	1816004.904	1640289.48	1726214.256	2538019.102	1799912.91	1552165.282	3404724.666	2964343.057	3230545.076
Com_11288_pos	Pregnenolone	Pregnenolone; 5-Pregnen-3beta-ol-20-one; 3beta-Hydroxypregn-5-en-20-one	C21 H32 O2	316.23992	9.736	317.2471	1292663.364	1643103.486	1650030.582	1426914.313	2050999.412	1709482.658	2799226.072	2688745.377	2953607.304	4094119.294	1777556.474	3246227.435	2455749.397	2238451.369	2243293.6
Com_13720_pos	Menaquinone	Menaquinone; Vitamin K2	C31 H40 O2	444.30459	8.279	445.3121	882260.4885	903196.544	1000338.234	1319872.049	1615511.688	1879091.4	2200445.621	1549836.866	1797806.439	1556623.505	2605198.201	2186422.35	1174045.58	1170057.496	1120248.864
Com_15320_pos	Phe-Pro	--	C14 H18 N2 O3	262.13165	5.721	263.1394	2606403.524	1938615.983	2024361.982	1994475.681	2739814.076	2180003.951	1434483.584	1039083.9	1108792.589	1616468.75	972045.7373	1252171.677	1668406.585	1772018.11	1616829.922

Supplemental Table S9: All differential metabolites between fecal samples of captive and wild *Cervus elaphus kansuensis*

Compound ID	Name	Formula	Molecular weight	RT [min]	m/z	mzCloud Results	mzVault Results	MassList Results	All_C1	All_C2	All_C3	All_C4	All_C5	All_C6	All_W1	All_W2	All_W3	All_W4	All_W5	All_W6	FC	log2FC	*P* value	ROC	VIP	Up/Down
Com_293_pos	2-[2-(2-pyridyloxy)ethoxy]pyridine	C12 H12 N2 O2	198.07916	5.481	199.0864	Invalid mass	No results	No results	629728265.4	657805344.1	472794715.2	508235091.9	661231365.6	670992044.2	2352694.79	871888.7348	713335.6989	817739.8957	3952801.907	1536285.69	351.4764	8.457284	1.72E-06	1	1.310226	up
Com_808_pos	Theophylline	C7 H8 N4 O2	180.06342	5.481	181.0707	Invalid mass	No results	No match	189047633.6	198195545.5	130686051.1	204241297.9	184188793.3	209872356.6	1086544.199	154544.5249	154825.0084	193897.8546	1729851.91	161730.863	320.6278	8.324756	2.51E-05	1	1.291251	up
Com_410_pos	Harmine	C13 H12 N2 O	212.09478	5.484	213.1021	Full match	No results	No results	411393905.1	471095262.1	372189801.3	335511495.1	471342603.5	540134014.6	2017341.969	1129112.357	1085167.959	1173582.931	4080231.272	1392929.29	239.1597	7.901831	1.92E-07	1	1.314394	up
Com_336_neg	(+/-)-CP 47,497-C7-Hydroxy metabolite	C21 H34 O3	334.25043	9.159	333.243	Full match	No results	No results	349096167.5	313180607.6	73821832.97	66707896.4	83123884.24	72295915.46	483873.0817	518112.0754	969417.15	712217.8148	786738.6098	675825.521	231.1104	7.852438	3.93E-06	1	1.297933	up
Com_3599_neg	13,14-dihydro-15-keto Prostaglandin A2	C20 H30 O4	668.4286	7.702	667.4213	Invalid mass	No results	No results	14441196.69	15702730.65	21649256.94	14804796.53	4797400.352	12279515.78	71189.72833	52242.60454	57066.61345	54981.73663	121343.2917	58592.0934	201.4243	7.654094	1.46E-08	1	1.310397	up
Com_441_neg	O-Desmethylnaproxen	C13 H12 O3	216.07864	5.356	215.0712	Full match	No results	Full match	159975307.6	190549039.5	219848753.6	216576207.3	163921198.7	176291809.1	3076621.803	882157.4646	849009.1092	2282011.236	866261.8252	932815.073	126.8059	6.986478	1.83E-06	1	1.30931	up
Com_341_neg	L-Tyrosinemethylester	C10 H13 N O3	195.08923	5.598	194.0819	No results	No results	Full match	309430217.3	345967318.9	331292192.4	287375080.7	336575777.3	332444757.5	4865324.66	2721784.538	1363858.026	2725422	2983414.752	4557936.23	101.1089	6.659767	1.26E-06	1	1.314056	up
Com_1975_pos	D-δ-Tocopherol	C27 H46 O2	402.34724	10.151	403.3544	Invalid mass	No results	No results	60742509.58	94170309.41	86260554.08	49296833.91	57536114.95	25421934.51	950129.8537	775901.9271	668485.034	367940.001	597074.3682	411363.398	99.02909	6.62978	8.43E-09	1	1.306456	up
Com_1292_neg	FAHFA (2:0/23:0)	C25 H48 O4	412.35495	10.407	411.3478	No results	Full match	No results	63865891.5	81521210.61	75972743.37	69322064.93	78754375.98	49271570.72	1312097.61	893511.4822	532434.6493	734420.1626	968821.3078	704035.241	81.37644	6.346539	1.09E-09	1	1.3174	up
Com_9435_pos	gamma-Glutamylmethionine	C10 H18 N2 O5 S	278.09356	4.794	279.1009	No results	Full match	No results	8699010.999	1418706.757	5569917.428	3416035.337	2999786.43	10918944.89	76281.59217	75328.52694	107090.8183	135815.9769	79496.44818	88126.6415	58.74409	5.876372	2.09E-05	1	1.281304	up
Com_289_neg	2-Hydroxyphenylalanine	C9 H11 N O3	181.07363	5.001	180.0664	Full match	No results	Full match	312859755.4	360638308.5	375401074	363006772.5	409635420.2	393482502.1	3192247.086	7305509.37	3320225.032	3995151.507	8595097.596	11536191.6	58.37548	5.867291	5.05E-06	1	1.306034	up
Com_361_neg	4-Methoxycinnamic Acid	C10 H10 O3	178.06273	6.159	177.0554	No results	No results	Full match	326813465.1	16380303.98	42674604.59	23354938.14	13050400.04	112886255.3	2366312.943	1492251.206	1446601.953	926090.0273	1347426.719	1782388.11	57.16867	5.837153	0.000838	1	1.190146	up
Com_5677_pos	Amlexanox	C16 H14 N2 O4	298.09518	6.301	299.1025	No results	Full match	No results	17469019.94	16164356.9	19377076.22	19459692.54	22340001.79	21044150.51	425259.5968	204442.3196	438768.475	282069.1075	459293.7911	226554.542	56.89206	5.830155	1.68E-07	1	1.315303	up
Com_5620_pos	4,7,8-trimethoxyfuro[2,3-b]quinoline	C14 H13 N O4	259.08394	5.48	260.0913	Full match	No results	No results	17298963.15	18155128.54	17828612.58	19779942.67	11546715.49	12511778.28	263637.1226	381369.5559	151208.9899	189369.2695	495494.9933	277800.454	55.21759	5.787056	8.77E-08	1	1.309114	up
Com_5699_pos	IMH	C17 H29 N5 O4 S	399.20053	5.059	400.208	Invalid mass	No results	No results	14106816.72	18011856.82	17679713.42	19346401.61	14244647.6	14354286.25	698734.4675	443964.6912	155473.0679	225411.284	160209.1733	698718.346	41.02551	5.358449	2.36E-05	1	1.289403	up
Com_6071_pos	Taurochenodeoxycholic Acid (sodium salt)	C26 H45 N O6 S	481.27801	5.446	482.2855	Invalid mass	No results	No results	14675374.05	23044475.4	18521467.25	16996903.71	17766120.83	20833091.28	412363.1354	462397.8231	430992.4054	539761.0091	450649.5688	439649.055	40.87905	5.35329	2.43E-11	1	1.321947	up
Com_2687_pos	methyl 3,3,3-trifluoro-2-hydroxy-2-[(2-phenoxyacetyl)amino]propanoate	C12 H12 F3 N O5	307.06919	5.122	308.0765	Invalid mass	No results	No results	55263968.11	62994560.77	59877026.54	27227915.73	51085484.49	51240253.54	1293091.752	1078125.494	846288.4347	1149927.485	1783290.594	1476775.19	40.33946	5.33412	9.70E-10	1	1.311833	up
Com_11446_pos	1-(5,7-dichloro-2,3,4,4a-tetrahydro-1H-xanthen-4-yl)pyrrolidine	C17 H19 Cl2 N O	323.0866	4.827	324.0939	Invalid mass	No results	No results	5527710.592	4131288.575	3594319.602	3808548.93	6387246.134	4002357.204	109399.2858	99625.40695	113187.2681	152034.749	101958.5561	147147.687	37.95031	5.24604	8.52E-11	1	1.317408	up
Com_2397_pos	N-{4-[(2R,3R)-3-(Hydroxymethyl)-5-oxo-2-morpholinyl]phenyl}acetamide	C13 H16 N2 O4	246.1004	5.002	247.1077	Invalid mass	No results	No match	40224384.4	81250062.48	49536661.11	29147918.66	49158714.23	80569142.6	1389376.7	1171720.935	1470629.988	1728324.266	1548360.648	1463651.4	37.60653	5.232911	7.59E-07	1	1.309642	up
Com_11138_pos	NNK	C14 H26 N6 O6	396.16838	5.382	397.1755	Invalid mass	No results	No results	2026988.712	5135788.904	5715592.806	5648762.914	3667601.603	5514832.265	142378.6166	109728.3962	111927.1837	141082.1101	235558.9406	101158.932	32.91571	5.040704	3.18E-08	1	1.301299	up
Com_11691_pos	16(R)-HETE	C20 H32 O3	342.21503	5.112	343.2223	Invalid mass	No results	No results	3401393.339	3627371.168	3290098.804	5052467.502	3135444.013	5886317.762	163569.6176	94108.84409	109220.0263	132737.17	117505.1383	134003.527	32.47457	5.021239	5.63E-10	1	1.315228	up
Com_3813_neg	4-Pregnen-17alpha,20alpha-Diol-3-One	C21 H32 O3	332.23488	8.88	331.2275	No results	No results	Full match	20654613.34	19717821.99	9321182.308	8279097.995	8878701.148	5271038.858	363761.0947	330570.6058	407019.4556	350638.3444	692310.9552	395642.472	28.39531	4.827581	1.96E-06	1	1.288982	up
Com_4801_neg	Anacardic acid	C22 H36 O3	348.26595	10.089	347.2587	Full match	No results	No results	7630121.382	10594639.36	11330363.96	12102927.26	7839183.906	7645612.724	466167.192	436936.7089	327497.203	234247.0268	245029.9166	334272.779	27.95432	4.804999	1.62E-09	1	1.312578	up
Com_4678_pos	Quinoline	C9 H7 N	129.05789	5.353	130.0652	Full match	No results	No results	30433600.17	27614815	18544363.11	13431375.66	22251471.49	7362771.399	553414.7895	702492.5626	771804.5418	1254362.373	654931.6272	583114.114	26.46797	4.726176	1.34E-06	1	1.287326	up
Com_1340_neg	FAHFA (18:0/7:0)	C25 H48 O4	412.35499	8.906	411.3478	No results	Full match	No results	20697528.7	16723987.73	34889252.55	66225742.03	25134796.3	20287423.59	1994838.148	386381.8429	1035767.675	1393421.364	2091371.612	405805.119	25.17367	4.653843	1.12E-05	1	1.24907	up
Com_9975_pos	4-Aminobiphenyl	C12 H11 N	169.08896	2.938	170.0962	Full match	No results	Full match	8438632.316	5204195.46	2544821.962	1697365.886	4139310.133	3450014.381	174876.9673	153643.3907	182962.6594	195985.1455	145090.5729	160987.689	25.13387	4.651561	2.42E-05	1	1.28887	up
Com_850_neg	Epinephrine	C9 H13 N O3	183.08922	5.012	182.0819	Full match	No results	Full match	66123261.92	91480477.98	76835564.11	108657420.2	83141118.6	79747597.53	3488293.403	3075182.862	3745316.695	3633811.885	3116766.773	3493382.6	24.61886	4.621692	7.19E-10	1	1.320944	up
Com_4145_pos	LPE 18:1	C23 H46 N O7 P	479.30078	9.562	480.308	No results	Full match	Full match	11821501.22	40283107.73	28761120.96	9540083.458	34404863.18	10870851.98	1276167.418	1129565.74	788255.3252	765912.9006	796782.8064	1107239.9	23.13835	4.532214	3.23E-05	1	1.270345	up
Com_3360_pos	N-[1-(4-methoxy-2-oxo-2H-pyran-6-yl)-2-methylbutyl]acetamide	C13 H19 N O4	270.1579	4.917	271.165	Invalid mass	No results	No results	46293223.95	47010358.34	39218013.1	40735985.31	35560092.22	36653476.86	1675205.36	2332507.374	1130283.371	2023130.316	715933.793	2891440.5	22.79529	4.510664	1.11E-05	1	1.296163	up
Com_11533_pos	LPH	C17 H27 N5 O4	365.20585	5.171	366.2132	Full match	No results	No results	5082123.846	5604049.393	5841367.375	3784591.283	5261019.783	6328610.884	201970.2484	182281.5132	285454.6391	326052.4275	204351.62	223263.908	22.41277	4.486249	3.07E-10	1	1.315368	up
Com_2455_neg	Nicotinate ribonucleoside	C11 H13 N O6	255.07411	6.53	254.0668	No results	No results	Full match	34010787.74	18520464.41	35981268.25	23393295.76	19933271.01	17678869.74	1235171.761	1370141.747	1031980.266	1137063.955	564172.0075	1344087.08	22.37416	4.483762	1.26E-08	1	1.301796	up
Com_2307_pos	6-methoxy-2-phenyl-3,4-dihydro-2H-1-benzopyran-4-one	C16 H14 O3	254.09398	5.641	255.1014	Full match	No results	No results	50521945.52	58585992.34	62043667.26	64490635.13	32306435.27	40679744.7	2840675.759	2535587.443	2601252.694	1026493.136	2364014.068	2762474.93	21.8413	4.448987	6.43E-08	1	1.3003	up
Com_3854_neg	Gly-Tyr	C11 H14 N2 O4	238.09517	5.214	237.0878	No results	No results	Full match	7836870.508	6395573.655	9884311.751	17331410.3	7829309.65	8500194.979	381070.0977	509524.3826	422353.5027	469281.787	365358.0484	499310.166	21.82845	4.448138	3.75E-07	1	1.308519	up
Com_8170_pos	5-Methyl-2'-deoxycytidine	C10 H15 N3 O4	241.10591	1.226	242.1133	No results	No results	Full match	4294045.515	8503262.292	11725109.79	9924650.266	4539395.277	3699727.09	79153.25719	194366.5805	111868.3019	969878.3895	527641.1431	151637.061	20.98071	4.390992	9.12E-05	1	1.223298	up
Com_2798_neg	LPE 15:0	C20 H42 N O7 P	439.26949	8.88	440.2768	No results	Full match	No results	59958506.76	50960252.85	25517814.5	13593223.52	30710423.37	21233917.93	2307228.412	1180348.226	1654525.966	1879321.674	651219.4997	1969386.35	20.94726	4.38869	1.75E-06	1	1.264847	up
Com_38_neg	Prostaglandin H2	C20 H32 O5	334.21403	7.716	333.2067	Invalid mass	No results	No match	2291031202	2365813438	2703674357	2150915992	1804741100	1977751687	90439446.47	108111113.1	140505231.5	146379332.8	47375237.32	109478345	20.69774	4.371402	1.77E-06	1	1.303253	up
Com_6147_neg	Lignoceric acid	C24 H48 O2	414.37803	10.16	413.3635	Invalid mass	No results	No results	2863354.498	4886497.387	9133036.359	6633784.675	7130639.973	4347601.033	374040.2826	339782.8265	306813.8642	219452.9749	234330.8544	264425.83	20.12536	4.330943	5.99E-07	1	1.29864	up
Com_8734_pos	PC (18:4e/2:0)	C28 H50 N O7 P	543.33203	9.053	544.3391	No results	No results	Full match	9526145.258	12381423.28	7601648.24	5269314.683	8543047.085	7786123.1	644348.1397	369428.8795	389119.0944	424415.7374	596648.771	348456.588	18.43435	4.204324	4.78E-09	1	1.305882	up
Com_6632_pos	1-[(3,5-dimethylisoxazol-4-yl)sulfonyl]piperidine	C10 H16 N2 O3 S	244.08495	5.044	245.0922	Invalid mass	No results	No results	17745493.36	13734268.62	7363582.4	7185641.902	17135795.33	14733854.44	638019.5144	291902.5726	698463.5134	891464.4274	1043618.521	694449.699	18.29501	4.193378	3.47E-07	1	1.279992	up
Com_14522_pos	3-amino-2,6-diphenyl-4,7-dihydro-2H-pyrazolo[3,4-d]pyrimidin-4-one	C17 H13 N5 O	303.11394	4.169	304.1212	Invalid mass	No results	No results	1792754.222	1549906.749	2455656.753	2844448.98	1379121.976	1691778.235	51900.50645	68468.15226	121519.8526	293304.519	43789.88574	61575.868	18.28664	4.192718	3.29E-05	1	1.263786	up
Com_8960_pos	methyl 7-hydroxy-4-oxo-8-propyl-4H-1-benzothiine-2-carboxylate	C14 H14 O4 S	278.05769	5.303	279.0649	Invalid mass	No results	No results	9249462.047	10188404.78	9971521.117	7848639.733	6812677.4	8376686.458	587912.5225	549772.1246	377822.0025	348131.3229	481010.1174	549197.579	18.12377	4.179811	6.92E-10	1	1.315572	up
Com_3119_neg	Ascorbic acid	C6 H8 O6	176.03186	1.979	175.0245	Full match	No results	Full match	23995849.09	25300205.66	24844475.21	23392845.87	26464906.89	15678814.57	1639453.823	1092485.271	1198616.579	1179196.149	1312385.516	1585593.2	17.44278	4.124558	1.28E-10	1	1.31606	up
Com_39_pos	Stercobilin	C33 H46 N4 O6	594.34113	5.46	595.3483	Full match	No results	No results	2860625310	3298814774	3902689546	3661199921	2752296172	3081085721	87276681.75	172341042.9	141876660.2	429053665.1	161285421.3	138151909	17.30705	4.113288	1.60E-05	1	1.289483	up
Com_9702_pos	Nervonic acid	C24 H46 O2	366.34941	8.845	367.3568	Full match	No results	Full match	8900861.389	8574719.386	7104573.662	7328185.189	4373530.419	3899027.862	348719.3267	310423.5425	528507.3161	496770.5941	326618.9968	350306.068	17.0161	4.088829	8.75E-08	1	1.300891	up
Com_9610_pos	Palmitoyl sphingomyelin	C39 H79 N2 O6 P	702.56536	6.325	703.5724	Full match	No results	No results	5252273.334	4924832.891	4992978.217	5800147.151	9294579.628	6877038.143	222564.4854	178307.547	200912.1202	246010.9566	1205282.498	175481.647	16.66631	4.058862	6.15E-05	1	1.259833	up
Com_9443_pos	LysoPE 18:0	C23 H48 N O7 P	481.31575	8.999	482.3235	No results	No results	Full match	5465860.68	6214976.958	8980288.338	3515701.506	7650367.506	5687465.343	362089.6767	319467.2946	444046.9857	472005.8804	309659.639	347067.604	16.64111	4.056679	1.17E-07	1	1.305924	up
Com_9972_neg	Heneicosanoic acid	C21 H42 O2	326.31821	11.727	325.311	No results	Full match	No results	2581941.02	2456313.809	2548847.922	1359832.598	3726163.998	849587.4116	268285.5595	162905.2254	79431.4616	51858.20078	79805.525	178048.194	16.48436	4.043026	7.82E-06	1	1.242439	up
Com_7054_pos	Lysope 14:0	C19 H40 N O7 P	425.25352	8.591	426.2612	No results	No results	Full match	16110562.58	12760083.87	6906108.204	5128626.44	9484165.215	7639003.211	1356228.884	375380.2525	454884.4427	484526.9576	309336.5652	568989.385	16.34908	4.031138	1.40E-06	1	1.26854	up
Com_7048_neg	LPE 20:4	C25 H44 N O7 P	501.28583	9.084	500.2784	No results	Full match	No results	5176255.313	8495243.97	4413574.413	2399729.97	6484683.021	958793.4637	270333.8536	253403.652	357014.9926	393296.1927	223419.3487	246521.875	16.01401	4.001263	0.000305	1	1.224661	up
Com_259_pos	Adenosine	C10 H13 N5 O4	267.09643	2.552	268.1037	Full match	Full match	Full match	733161253.5	399210756.5	696093597.9	172286946.1	204037508	251674948.1	19506975.48	54119750.71	22838107.82	3487480.486	6575718.471	46990178.9	16.00113	4.000102	0.000371	1	1.169124	up
Com_9003_pos	LPE 18:3	C23 H42 N O7 P	475.26946	8.638	476.2766	No results	Full match	No results	6057842.541	11668424.36	7576899.267	4054921.543	6823804.041	4830421.393	994349.529	448425.6704	339318.6618	305651.7408	208168.1053	301452.449	15.78996	3.980936	2.25E-06	1	1.270302	up
Com_4523_pos	N-Acetyldopamine	C10 H13 N O3	195.08943	4.799	196.097	Full match	No results	Full match	26045841.74	35938421.55	24772410.76	21474526.95	31389554.28	26136486.25	1523794.224	1527782.359	1607144.287	2670297.511	1694769.862	1676318.04	15.49118	3.953375	5.13E-10	1	1.313329	up
Com_608_neg	Levodopa	C9 H11 N O4	197.06861	5.072	196.0613	No results	No results	Full match	182517186.4	152719665.1	156283481.9	144781120.6	127726247.9	123557803.1	14082142.08	8818280.562	8620750.587	9253131.628	8865702.327	8449825.1	15.27953	3.933529	3.58E-10	1	1.316139	up
Com_3719_pos	1-(3,4-dimethoxyphenyl)ethan-1-one oxime	C10 H13 N O3	195.08927	2.18	196.0965	Full match	No results	Full match	19345402.34	25339739.73	18164139.85	19205323.43	42937924.88	30904426.26	2709676.753	1355577.649	1607933.012	1940877.479	1204657.931	1682177.21	14.84606	3.892008	5.37E-08	1	1.295498	up
Com_465_pos	Eicosapentaenoic acid	C20 H30 O2	302.22419	6.671	303.2315	Full match	Full match	Full match	330513782.1	333471657	401822003.7	339886103.2	284656968.6	339027088.2	23879280.72	14887166.09	21106567.56	39577172.59	21088453.51	18423151.2	14.60385	3.868277	1.07E-06	1	1.307265	up
Com_4751_pos	DL-Tryptophan	C11 H12 N2 O2	226.07426	5.241	227.0815	Invalid mass	No results	No results	25882684.28	20790229.52	18553490.72	25261666.37	25090900.66	25598880.26	1945794.9	1406800.882	1623271.162	1765937.186	1358603.853	1686582.93	14.42505	3.850505	1.33E-11	1	1.318793	up
Com_1831_pos	N-Acetyl-D-tryptophan	C13 H14 N2 O3	246.10039	5.267	247.1077	No results	No results	Full match	98559140.05	104115451.1	79437810.78	73804105.35	92527437.92	94099424.94	11427797.14	4388597.24	6052517.122	7199172.323	2960533.064	5679912.06	14.38782	3.846776	9.69E-06	1	1.292835	up

Com_11423_pos	4,5-diphenyl-1,3-oxazole-2-thiol	C15 H11 N O S	253.05849	3.863	254.0658	Invalid mass	No results	No results	2249613.608	2119450.772	5723364.451	5331839.172	2372907.993	2584183.797	188134.9147	129012.3547	231216.7022	466556.8446	226630.537	204675.893	14.09278	3.816884	1.10E-06	1	1.267986	up
Com_1427_pos	dihydrotachysterol	C28 H46 O	398.35433	10.234	399.3616	No results	No results	Full match	85783689.05	124729149.1	125457702.4	6180681.931	78999429.42	50343404.89	6341944.868	9080922.019	5290688.086	4308590.332	4546146.012	4879021.31	13.6874	3.774776	0.003411	0.944	1.110609	up
Com_7227_pos	4-oxo-5-phenylpentanoic acid	C11 H12 O3	209.10516	4.654	210.1125	Invalid mass	No results	No results	9259317.58	11751914.12	7737532.506	8088710.197	15734171.31	14975703.76	815192.3747	876880.9042	696150.1361	1283462.596	542262.756	863162.75	13.30429	3.733819	3.55E-08	1	1.296444	up
Com_9867_pos	L-Kynurenine	C10 H12 N2 O3	208.08492	3.754	209.0922	No results	No results	Full match	8635128.011	7290280.651	5439969.421	5753023.15	4780731.891	4706471.546	487234.6811	303598.8875	532351.3852	599102.4352	467364.9984	368156.275	13.27344	3.73047	5.45E-09	1	1.305148	up
Com_15188_pos	Tyrosylalanine	C12 H16 N2 O4	252.10924	4.64	253.1165	Invalid mass	No results	No results	2860997.383	1910258.988	684854.5077	1267024.044	748730.0938	538016.728	93797.22628	82960.05045	103912.0225	105825.6047	128607.2027	90109.3204	13.23485	3.72627	0.000179	1	1.247068	up
Com_788_neg	Perillic acid	C10 H14 O2	166.09913	5.447	165.092	Full match	No results	Full match	137157128.1	130373735	108175055	104953887.8	98727035.51	93794868.26	8196135.266	7012654.768	5031841.954	6650062.204	10984807.38	12999134.2	13.23217	3.725978	7.46E-07	1	1.302156	up
Com_4484_neg	3b,7b-Dihydroxy-5-androsten-17-one	C19 H28 O3	304.2035	8.227	303.1963	No results	No results	Full match	2931300.262	3412235.233	12201218.85	13608104.43	8891352.594	6038070.83	668013.2901	358975.8154	650935.2999	811549.9808	345316.4864	737299.349	13.1806	3.720344	3.95E-05	1	1.234227	up
Com_6775_pos	2-Methylbutyroylcarnitine	C12 H23 N O4	245.16264	5.283	246.1699	No results	No results	Full match	17091153.3	17594472.31	14909970.07	13398748.22	11558604.21	13416771.76	1097163.335	1124650.32	862660.3549	1130693.563	1291248.055	1309171.47	12.90714	3.690097	7.19E-11	1	1.316531	up
Com_4639_neg	LPE 13:0	C18 H38 N O7 P	411.23827	8.175	410.2309	No results	Full match	Full match	15065303.34	5849266.243	4120005.003	2644259.024	4114682.808	3205960.239	830841.842	370549.0195	473105.0943	404815.9995	341962.0992	358738.862	12.58968	3.65417	4.10E-05	1	1.238993	up
Com_6321_neg	Thymidine 3',5'-cyclic monophosphate	C10 H13 N2 O7 P	258.03719	1.404	257.0301	Invalid mass	No results	No results	4355424.656	4044977.422	8694252.06	5518214.106	6249298.172	4193843.458	303211.5823	549605.9497	495869.6212	406343.4032	428427.1107	459977.613	12.50494	3.644427	4.38E-08	1	1.30264	up
Com_4364_pos	Artemisinin	C15 H22 O5	586.2781	6.154	587.2858	Invalid mass	No results	No results	8901464.55	13108739.82	25605332.62	28491550.03	18278679.67	21661325.79	1713777.699	1491904.2	453545.7975	3547141.705	1234774.359	844689.305	12.49722	3.643535	3.47E-05	1	1.229906	up
Com_9167_pos	N1-(2,3-dihydro-1,4-benzodioxin-6-yl)acetamide	C10 H11 N O3	193.07132	4.68	194.0786	Invalid mass	No results	No results	459863.7025	9915137.634	9484757.294	5960838.109	5852944.409	11397703.29	85049.17259	73581.04486	935469.8935	835252.2702	769847.5184	794363.222	12.32874	3.623954	0.004083	0.889	1.007599	up
Com_8778_pos	(±)-Abscisic acid	C15 H20 O4	286.11631	3.894	287.1235	Invalid mass	No results	No results	7728626.681	7765659.864	7803338.387	9261759.072	8978434.884	7330596.974	336171.0582	224630.4082	395906.9155	2253152.756	534715.3734	254787.767	12.21905	3.61106	0.000362	1	1.239445	up
Com_5577_pos	Isobutyryl carnitine	C11 H21 N O4	231.14712	5.131	232.1544	No results	No results	Full match	23539196.39	18424956.29	14760781.23	14682291.75	14948634.25	14373472.41	1397138.56	1340972.703	1230139.457	1617782.695	1433801.115	1589339.74	11.70023	3.548465	8.32E-09	1	1.315614	up
Com_8164_pos	1-oxo-2,3-dihydro-1H-inden-4-yl benzoate	C16 H12 O3	252.07856	5.314	253.0859	Full match	No results	No results	5725501.624	7446619.353	6778791.09	10505712.17	6128289.232	3504423.257	723917.7745	430063.159	644715.091	662444.9909	475180.0806	529269.597	11.56782	3.532045	6.01E-07	1	1.293178	up
Com_12296_pos	acetyl phosphate	C2 H5 O5 P	139.98703	1.174	140.9945	No results	No results	Full match	3322733.006	5939320.363	1048369.364	1702369.209	832777.4947	1202446.189	229091.8021	151899.0824	176578.8831	310923.9509	89891.10396	262050.631	11.51066	3.524898	0.000216	1	1.184866	up
Com_4593_neg	LysoPE 18:2	C23 H44 N O7 P	477.28609	8.974	476.2778	No results	No results	Full match	5353590.115	9901388.268	9882560.465	13061669.76	3386066.867	2615588.372	400820.2328	1392692.078	1017271.109	391868.9704	345364.7508	453163.414	11.04696	3.465577	5.64E-05	1	1.199315	up
Com_5134_pos	LPE 16:0	C21 H44 N O7 P	453.2846	9.364	454.2919	No results	Full match	Full match	11410483.37	28025320.3	18279564.27	7780228.313	27290274.51	10258100.59	3208625.523	990673.8343	837050.5343	2219043.696	1408896.45	733459.648	10.96475	3.454801	2.13E-05	1	1.221059	up
Com_549_pos	Adenine	C5 H5 N5	135.05446	2.573	136.0617	No results	No results	Full match	316312209.4	347331065.9	340896344.3	191387892.5	83485671.34	141905693.6	16878652.96	39557040.69	26156685.44	3659213.027	6552435.858	38421473	10.83112	3.43711	0.000516	1	1.153455	up
Com_3470_neg	Uric acid	C5 H4 N4 O3	168.02809	1.388	167.0207	Full match	Full match	Full match	12481573.71	15155552.56	22836958.26	12897144.11	12391089.92	14579919.06	223431.7398	1747334.927	714698.3933	4830672.676	275397.8976	602050.355	10.76325	3.428041	0.001307	1	1.176092	up
Com_1230_pos	2'-Deoxyadenosine	C10 H13 N5 O3	251.10159	2.758	252.109	Full match	Full match	Full match	154491321.8	78379886.14	93595465.75	58495118.65	23243994.05	30400614.13	1291843.376	12426749.84	7360265.472	497946.228	562421.4382	20149002.1	10.37183	3.374599	0.004449	1	1.057774	up
Com_4155_pos	Dithranol	C14 H10 O3	226.06293	5.372	227.0703	No results	No results	Full match	21369714.38	38305341.67	24560966.31	25432703.03	36440104.57	34525574.19	2660060.351	2774695.889	2170320.987	5313149.835	1468360.419	3093571.8	10.33368	3.369282	2.22E-06	1	1.280758	up
Com_10119_pos	(2R,3S,4S,5R,6R)-2-(hydroxymethyl)-6-(2-phenylethoxy)oxane-3,4,5-triol	C14 H20 O6	306.10754	7.735	307.1147	Invalid mass	No results	No results	8206710.572	5351898.843	6312695.268	4869289.76	6137360.725	8159505.962	718623.5301	480012.0162	594365.3266	690827.1884	743107.1299	561796.226	10.30357	3.365072	3.00E-09	1	1.309678	up
Com_3597_pos	S-Adenosylhomocysteine	C14 H20 N6 O5 S	384.11928	5.778	385.1266	Invalid mass	No results	No results	30224646.19	9158913.147	33426787.22	37438397.98	31539218.38	21461962.41	3252897.333	2527607.588	2443451.605	2765240.479	2074402.599	2991505.07	10.1681	3.345978	6.20E-05	1	1.263553	up
Com_11506_pos	3-hydroxy-N-(1-hydroxy-4-methylpentan-2-yl)-5-oxo-6-phenylhexanamide	C18 H27 N O4	303.18234	6.042	304.1899	Invalid mass	No results	No results	5729166.781	5063344.186	5864926.72	4330307.174	4492774.349	4859977.771	446717.215	449181.1451	496926.1198	603978.8089	498702.7978	507086.181	10.10477	3.336964	1.44E-11	1	1.319005	up
Com_1208_neg	all-cis-4,7,10,13,16-Docosapentaenoic acid	C22 H34 O2	330.25545	10.33	329.2482	Full match	No results	Full match	51517417.2	74058366.72	84204392.16	50629171.01	41977672.26	38469704.98	6073553.563	4395436.593	6876211.457	6815995.107	5762701.917	4350583.97	9.944912	3.313959	1.71E-07	1	1.296022	up
Com_8851_neg	Lysopc 16:1	C24 H48 N O7 P	493.3183	8.403	492.3109	No results	No results	Full match	3264064.804	5170895.79	4678078.384	3373163.523	3927277.597	4123310.015	381630.7554	241985.2977	550160.9194	578146.7102	306719.6658	423820.06	9.884049	3.305102	6.29E-07	1	1.295912	up
Com_3006_pos	1-(7-methoxy-2-oxo-2H-chromen-8-yl)-3-methyl-2-oxobutyl acetate	C17 H18 O6	340.09436	5.317	341.1018	Invalid mass	No results	No results	54773426.85	30364531.49	31704465.1	21845102.39	2909681.397	32505786.58	4710863.653	2759669.087	3018605.861	2343432.219	2024901.933	2777613.98	9.872534	3.30342	0.003773	0.944	1.097271	up
Com_2476_pos	2-(3,5-dimethyl-1H-pyrazol-4-yl)-5-methoxybenzoic acid	C13 H14 N2 O3	246.10038	5.118	247.1077	Full match	No results	Full match	52970843.99	54916206.98	34496729.21	40912668.58	71108588.58	62454464.54	11427797.14	4388597.24	1763756.555	7199172.323	1808749.828	5679912.06	9.819625	3.295668	0.000228	1	1.222344	up
Com_3909_neg	2-(1H-1,2,3-benzotriazol-1-yl)-N-(2,3-dihydro-1H-inden-2-yl)acetamide	C17 H16 N4 O	292.13085	6.652	291.1236	Invalid mass	No results	No results	5956901.443	6478348.889	15114929.82	16974205.02	5310960.847	6466423.74	888717.1961	1049876.297	933922.2606	754609.2937	906561.664	1267902.14	9.704543	3.27866	5.39E-05	1	1.261805	up
Com_6988_pos	LPE 16:1	C21 H42 N O7 P	413.31319	8.807	414.3191	No results	Invalid mass	No results	16307765.3	15808030.76	11713496	7273701.729	10858298.82	9292595.418	1215031.115	933231.5394	989800.0324	1415724.168	1287123.131	1512962.7	9.689301	3.276393	2.38E-07	1	1.29643	up
Com_5204_pos	Methylimidazoleacetic acid	C6 H8 N2 O2	140.05843	1.278	141.0656	Full match	No results	Full match	21609698.05	29453275.67	20502470.35	21211685.38	14807839.79	14748141.6	1516079.513	2829376.887	1387400.295	2907684.129	1837621.446	2315038.41	9.562354	3.257366	1.28E-07	1	1.29009	up
Com_13077_pos	PC (14:1e/3:0)	C25 H50 N O7 P	507.33223	9.23	508.3395	No results	Full match	No results	2296414.677	1443557.624	4267781.274	1468069.142	3946252.626	2467985.578	253064.0732	268791.7242	226086.7448	329217.1832	324293.3495	342237.858	9.112888	3.187908	3.10E-05	1	1.268997	up
Com_963_neg	Adrenic acid	C22 H36 O2	332.27112	10.589	331.2638	Full match	No results	No results	89456817.46	126478742.9	69591003.71	45040448.29	73428560.55	62275751.17	7649238.815	8858617.822	10406386.84	10033604.43	7745250.872	6579375.92	9.093989	3.184913	1.61E-06	1	1.290439	up
Com_15462_pos	N-{[5-(tert-butyl)-2-thienyl]carbonyl}-N'-(6-methyl-2-pyridyl)urea	C16 H19 N3 O2 S	317.12183	1.435	318.1292	Invalid mass	No results	No results	1832016.177	1933974.665	1708008.676	2294175.484	2567301.691	2206530.866	103151.7843	102686.5986	103949.7616	684978.3559	254235.1973	147381.47	8.981781	3.167002	0.000375	1	1.229579	up
Com_3694_pos	Tetranor-12(S)-HETE	C16 H26 O3	288.1684	4.785	289.1758	Invalid mass	No results	No results	42460317.62	36946353.51	10970114.86	15513644.94	30396313.43	22050071.32	2855477.178	2260369.193	2318297.85	4913599.994	2089768.764	3253453.81	8.950151	3.161912	2.34E-05	1	1.242085	up
Com_320_neg	11-dehydro Thromboxane B2	C20 H32 O6	350.20908	6.964	349.2017	Invalid mass	No results	No results	361442864.8	352243856.5	353718756.2	309556738.9	260730360.3	258788775.4	115240038.1	19907514.17	20028842.35	21002055.39	21832470.36	19237744	8.729542	3.125906	0.000274	1	1.236909	up
Com_744_neg	Uridine	C9 H12 N2 O6	244.06927	2.315	243.062	Full match	Full match	Full match	52546933.27	73424952.11	61322555.22	34029189.99	144611655.9	82926610.34	2500887.91	9444245.298	13856710.27	7294881.538	5022335.281	13946500.7	8.62109	3.10787	8.98E-05	1	1.195315	up
Com_4185_neg	LPC 18:1	C26 H52 N O7 P	567.35327	9.695	566.3461	No results	Invalid mass	No results	5754634.485	13206098.41	17381091.13	3866690.956	10233996.67	6313232.544	1534930.235	1578333.203	727295.1836	755311.0894	1125004.335	895349.656	8.578269	3.100686	5.08E-05	1	1.224854	up
Com_11429_pos	Obscurolide A1	C15 H17 N O5	313.0982	4.743	314.1054	Invalid mass	No results	No results	3941711.642	3329743.481	4978937.907	5326025.53	2873848.292	3219064.163	345628.1307	697924.1191	368203.1319	686441.4258	72360.37674	596910.858	8.552703	3.09638	0.000761	1	1.187889	up
Com_2842_neg	6-Methoxy-2-naphthoic acid	C12 H10 O3	202.06259	5.351	201.0556	No results	No results	Full match	19869245.64	26584180.13	29740511.98	26290393.54	16585201.72	26368399.71	3804211.167	3346960.844	2740128.233	1838668.275	2858852.853	2662237.2	8.430667	3.075647	2.50E-08	1	1.299194	up
Com_2571_pos	3,4,5-trihydroxy-6-methyloxan-2-yl 2-(methylamino)benzoate	C14 H19 N O6	319.1049	5.734	320.1122	Invalid mass	No results	No results	62895131.28	55592000.8	52682008.14	56008156.39	68071910.93	67717338.45	9988600.495	4430815.307	6602234.666	4180532.475	11161051.58	7587901.27	8.258411	3.045864	2.12E-05	1	1.285186	up
Com_1189_neg	N-(1,2,3,4-tetrahydro-1-naphthalenyl)benzenesulfonamide	C16 H17 N O2 S	287.10019	5.182	286.093	Invalid mass	No results	No results	39137648.14	57689827.66	65543272.23	75188131.55	54768934.3	70115174.23	6028056.715	9833802.85	5240149.256	8203550.835	4926706.963	10285191.6	8.141592	3.025311	3.25E-07	1	1.287529	up
Com_7590_pos	L-Saccharopine	C11 H20 N2 O6	276.13156	1.253	277.1387	Full match	Full match	Full match	3441112.001	4700439.192	6425706.053	7505584.233	14454947.36	9323037.441	1091537.975	1166302.216	795894.4802	555436.4704	669822.8091	1356001.85	8.136799	3.024461	2.13E-05	1	1.235812	up
Com_15434_pos	N1-[2-oxo-6-(1H-pyrrol-1-yl)-2H-chromen-3-yl]acetamide	C15 H12 N2 O3	268.08458	6.397	269.0919	Full match	No results	No results	2104092.026	2818327.772	2462741.484	2295617.954	2779314.575	3011042.195	564225.625	177815.5202	218183.4783	277145.9549	483753.7068	185198.778	8.115695	3.020715	5.83E-05	1	1.267846	up
Com_2650_neg	FAHFA (19:1/6:0)	C25 H46 O4	410.3392	9.635	409.3319	No results	Full match	No results	22743289.68	21171335.85	21078995.98	28524701.67	18431231.72	14147472.25	2734656.562	4150650.71	1574483.463	1712477.505	2475523.703	3240515.05	7.936467	2.988497	1.64E-06	1	1.279914	up
Com_2101_pos	methyl 2,5-dimethyl-1H-pyrrole-3-carboxylate	C8 H11 N O2	175.0582	5.798	176.0654	Invalid mass	No results	No results	84031892.88	80106222.97	54165062.01	41990207.01	66577601.94	65098347.11	24647771.01	3337689.811	3133137.052	3502847.03	11560874.73	3314629.26	7.919061	2.985329	0.000703	1	1.190507	up
Com_9566_neg	(±)10(11)-EpDPA	C22 H32 O3	344.23772	9.282	343.2307	Invalid mass	No results	No results	4078209.231	3200780.905	2217201.343	1111422.361	2498704.217	2684248.659	305690.122	272279.1866	277464.9565	498733.4092	277515.3122	394433.386	7.793514	2.962274	1.19E-05	1	1.260085	up
Com_392_pos	(+/-)5(6)-EET	C20 H32 O3	320.23483	6.684	321.2423	Full match	No results	No results	395013055.6	390621384.5	461194081.5	411425965.6	358229741.4	416665348.4	64059254.2	46827358.18	46489224.42	75196460.64	53477496.48	32864753.8	7.64515	2.934545	3.24E-06	1	1.301887	up
Com_12165_pos	Lysope 16:0	C21 H44 N O7 P	453.28505	8.936	454.292	No results	Full match	Full match	5419892.302	2945923.301	2563280.607	1164150.681	2805579.483	1719478.037	318625.4197	363343.9648	415838.7225	495639.1143	265203.4657	319622.924	7.629117	2.931516	8.37E-05	1	1.239486	up
Com_1444_pos	8-Hydroxyguanosine	C10 H13 N5 O6	299.08733	5.631	300.0946	No results	No results	Full match	98998113.75	106134348.1	102084649.7	110669023.7	99924494.14	109070070.9	16987137.1	12953987.17	13296179.97	15755666.99	9400391.323	13986304.3	7.609653	2.927831	9.07E-07	1	1.313096	up
Com_17085_pos	VLK	C17 H34 N4 O4	358.25774	4.817	359.2647	Full match	No results	No results	1380618.046	1171300.85	590449.6216	558620.2771	1740711.519	1474725.268	75294.38205	157186.3711	224364.2624	225459.5257	86306.52953	151922.7	7.513495	2.909484	2.62E-05	1	1.215653	up
Com_877_neg	methyl 2-[(2-acetyl-3-oxo-1-butenyl)amino]acetate	C9 H13 N O4	199.08422	5.006	198.0769	Full match	No results	No results	123757817.2	107264448.6	103004605.3	83858298.69	86892658.74	84751768.01	14142526.38	13760627.97	12517161.13	12410050.01	10194996.44	15624159.6	7.495654	2.906054	6.19E-10	1	1.312242	up
Com_4912_pos	2-(tert-butyl)-6,7-dimethoxy-4H-3,1-benzoxazin-4-one	C14 H17 N O4	263.11567	5.281	264.123	Full match	No results	No results	12296263.43	32094619.28	15559328.56	11806080.11	12355589.4	15649453	1884163.022	1859033.919	3096324.526	3173448.181	1461857.936	1976780.6	7.416313	2.890702	2.26E-06	1	1.262273	up

Com_4207_pos	Feruloyl Putrescine	C14 H20 N2 O3	264.14739	4.952	265.1547	No results	No results	Full match	21989923.45	10481773.18	9089701.566	15899518.78	35898157.82	11940358.4	2009584.727	1831260.673	2821280.387	3622200.827	1708672.881	2238654.89	7.398959	2.887322	5.25E-05	1	1.231043	up
Com_4639_pos	α-Lapachone	C15 H14 O3	264.07849	5.334	265.0858	Invalid mass	No results	No results	5359364.441	20456901.82	29346833.43	18093062.53	21909729.76	19950806.06	4062723.399	2263465.185	2731174.695	2134500.924	1721168.419	2664065.34	7.390125	2.885599	0.000156	1	1.21158	up
Com_3698_neg	ethyl 3-hydroxy-4,6-dimethoxy-2-oxoindoline-3-carboxylate	C13 H15 N O6	281.08962	5.2	280.0824	Full match	No results	No results	13575119.95	18697024.03	20838204.74	14408336.93	21343384.31	11991312.96	2635738.939	2160603.807	2380527.868	1946755.27	2213727.523	2314386.7	7.387584	2.885103	5.44E-07	1	1.305714	up
Com_10557_pos	3-pentadecyl-4,5,6,7-tetrahydrobenzo[d]isoxazol-4-one oxime	C22 H38 N2 O2	362.29656	7.936	363.3038	Invalid mass	No results	No results	6180140.429	5749254.552	5757109.631	5417526.437	7408173.829	8634486.219	802953.3162	602287.8329	733915.9026	1974358.839	632484.6362	586379.156	7.341317	2.876039	2.81E-05	1	1.267335	up
Com_3870_neg	Glycerophospho-N-palmitoyl ethanolamine	C21 H44 N O7 P	453.28526	9.433	452.2781	Full match	Full match	Full match	9128801.331	23082305.34	15595314.26	5911059.019	19144643.02	6719129.624	5090780.66	1461694.243	1002048.03	1065935.742	732147.6779	1524217.88	7.316589	2.871171	0.000192	1	1.163111	up
Com_7100_pos	(2,6-dimethylpiperidino)(3,4,5-trimethoxyphenyl)methanone	C17 H25 N O4	307.17834	5.032	308.1856	Full match	No results	No results	15515799.96	17979511.85	14608064.25	13289773.17	10920184.23	11392076.76	1442665.743	2558154.098	2516203.78	2433409.971	1153104.364	1495082.24	7.216842	2.851368	2.21E-06	1	1.283722	up
Com_3673_neg	Geranylgeranyl pyrophosphate	C20 H36 O7 P2	450.18837	5.603	449.1811	Invalid mass	No results	No results	17576185.84	24588656.37	16804955.09	11054156.08	21699245.22	21652808.89	4192639.789	2727898.838	1960955.997	1730535.129	2352177.104	2758047.44	7.21118	2.850235	4.53E-07	1	1.277596	up
Com_3623_neg	LysoPC 12:1	C20 H36 N O7 P	433.22499	7.208	432.2177	No results	No results	Full match	8360379.116	7814427.766	12347096.38	18885074.58	9428411.404	7938212.571	1490082.988	1069075.502	709049.1953	3259825.613	1401365.922	1069260.35	7.198139	2.847624	2.36E-05	1	1.234064	up
Com_7017_pos	2-Arachidonyl Glycerol ether	C23 H40 O3	386.27916	10.012	387.2863	Invalid mass	No results	No results	14590065.53	13147032.79	15160582.4	13793888.61	14961525.42	15996768.67	2653111.655	2146659.1	1698073.531	2709771.682	1085301.374	1965072.14	7.150427	2.838029	1.68E-05	1	1.293011	up
Com_8621_pos	Methyl indole-3-acetate	C11 H11 N O2	189.07616	6.013	190.0835	Invalid mass	Invalid mass	No results	9474854.946	8324103.985	8164721.229	7076428.226	10604679.59	12954958.26	1308976.494	1276475.861	1237114.478	1243456.037	1837503.98	1073298.72	7.095523	2.826909	1.47E-08	1	1.302543	up
Com_4987_neg	2-Furoylglycine	C7 H7 N O4	169.03708	6.066	168.0298	Full match	No results	No results	13358302.64	5892968.636	11952143.28	10419881.62	11284621.85	8767681.244	1267258.556	1690643.198	1210017.095	2121665.704	1452290.964	1083660	6.988312	2.804944	2.83E-07	1	1.283578	up
Com_1360_pos	4-methoxy-9-(2-methylbut-3-en-2-yl)-7H-furo[3,2-g]chromen-7-one	C17 H16 O4	284.10452	6.052	285.1118	Full match	No results	No results	78453016.35	66651131.43	87010736.15	117990238.6	57323114.56	82084716.4	12822145.27	10985107.87	13179050.48	13980831.59	6967486.391	12145953.3	6.985002	2.804261	1.02E-07	1	1.289418	up
Com_11654_pos	3-(4-nitrophenyl)[1,2,3]triazolo[1,5-a]quinazolin-5-amine	C15 H10 N6 O2	306.085	4.825	307.0922	Full match	No results	No results	5895164.286	5411444.441	4266750.89	3260657.613	6130231.278	3835513.732	639185.687	657365.9341	673743.2367	895723.6185	641458.8787	702027.462	6.841603	2.774334	4.74E-07	1	1.300495	up
Com_9797_pos	6-methyl-4-(morpholinomethyl)-2H-chromen-2-one	C15 H17 N O3	259.12067	6.054	260.128	Full match	No results	No results	2751774.546	3756542.131	3747163.876	1820262.704	8708471.765	10089162.57	649947.3504	540717.6436	887787.7115	981354.4061	827743.1189	685951.311	6.75049	2.754992	0.000817	1	1.176759	up
Com_1490_neg	Pyridoxine	C8 H11 N O3	169.07374	4.988	168.0665	Full match	No results	Full match	60607538.88	54651643.23	49462784.04	47845297.55	67639133.21	38893107.49	8491892.497	7223325.59	5045883.19	8892622.908	5700063.872	12485930.1	6.670179	2.737726	1.46E-06	1	1.283531	up
Com_701_pos	Styrene	C8 H8	104.06294	4.795	105.0703	No results	No results	Full match	98659672.65	174008441.1	109782866.3	74168681.02	281481126.6	320068618.8	11732206.15	29527551.91	24733481.47	31508874.97	9790732.626	52564968.4	6.619441	2.726709	0.000329	1	1.140411	up
Com_4362_neg	N6-Succinyl Adenosine	C14 H17 N5 O8	383.10716	5.06	382.0999	No results	No results	Full match	10231255.22	6361293.079	6313715.71	4849468.107	16670957.18	10306271.25	1031869.184	1505375.846	1249652.14	1436100.107	980101.7483	2097007.59	6.594248	2.721208	2.19E-05	1	1.241239	up
Com_4785_pos	LPE 18:2	C23 H44 N O7 P	477.285	9.077	478.2922	No results	Full match	Full match	18412238.84	33180863.8	19907366.11	8729340.589	22828706.15	14859673.92	2339162.187	8336303.925	3854536.445	710482.4615	1342439.026	1302795.24	6.592868	2.720906	0.000918	1	1.140615	up
Com_6112_pos	Gamma-Glu-Leu	C11 H20 N2 O5	260.13703	4.891	261.1443	No results	No results	Full match	17334389.87	18055322.92	14147571.57	17265106.56	15381076.32	10579153.23	1466626.532	3410163.213	2165933.323	3245019.155	2011233.098	1904275.62	6.531084	2.707322	1.38E-06	1	1.28228	up
Com_7761_pos	(3R)-8-hydroxy-3-(4-methoxyphenyl)-3,4-dihydro-1H-2-benzopyran-1-one	C16 H14 O4	252.07846	6.319	253.0857	Invalid mass	No results	No results	8330789.728	10671952.36	12416664.9	11591230.66	9359715.465	11134265.6	2146730.534	1635813.639	2066479.639	1508499.749	706439.5544	1691494.22	6.509651	2.70258	2.47E-05	1	1.272841	up
Com_11682_neg	alpha-D-Glucopyranosyl 2-O-(2-methylbutanoyl)-alpha-D-glucopyranoside	C17 H30 O12	426.16856	7.166	425.1613	Invalid mass	No results	No results	2483808.404	2028146.18	1530109.975	1498442.409	1604247.702	1128893.609	351266.1508	226243.0743	195866.3759	217311.8889	339582.0984	267784.694	6.428848	2.68456	2.31E-07	1	1.283967	up
Com_3901_neg	Docosatrienoic Acid	C22 H38 O2	334.28689	11.072	333.2797	Full match	No results	No results	15668148.41	22816185.39	13389432.1	6268117.058	18038342.03	8826367.39	2199067.695	4324831.253	1664687.013	736266.4041	1413574.006	2972185.82	6.386377	2.674998	0.000166	1	1.173799	up
Com_3338_neg	Pyridoxine O-Glucoside	C14 H21 N O8	331.12594	4.954	330.1181	No results	No results	Full match	13688495.3	28336419.34	19163036.81	11866827.61	7886825.706	10271311.62	5119073.932	1996673.299	2041003.21	1300764.54	915445.8467	2925208.43	6.379342	2.673408	0.000138	1	1.180028	up
Com_1424_neg	3-(3,4-Dihydroxyphenyl)-2-Methylalanine	C10 H13 N O4	211.08425	5.135	210.0769	No results	No results	Full match	56049987.03	45774118.29	69346755.85	57522533.98	63745928.77	50310347.31	10368840.68	6805516.119	7547412.402	9046245.418	12310574.67	8489443.5	6.281144	2.651027	3.41E-08	1	1.302875	up
Com_4859_pos	(2R,3S,4S,5R,6R)-2-(hydroxymethyl)-6-(propan-2-yloxy)oxane-3,4,5-triol	C9 H18 O6	260.06793	5.348	261.0743	Invalid mass	No results	No results	20627316	24719243.17	27545728.59	19677106.06	22499238.55	26962979.04	3987538.747	3363802.702	4357229.38	3953207.551	3252603.825	4146092.01	6.159093	2.622718	4.77E-10	1	1.31366	up
Com_1623_pos	Sebacic acid	C10 H18 O4	202.12042	5.942	203.1276	No results	No results	Full match	112645281.6	102347489.6	48252455.48	49696137.28	85486865.47	84206401.4	9886769.611	10845597.91	13899844.03	17896948.25	12966058.73	12998906.7	6.148672	2.620275	6.25E-06	1	1.26843	up
Com_1105_pos	Ciprostene	C22 H36 O4	346.25039	8.194	347.2576	Invalid mass	No results	No results	134221310.3	68370369.61	106188002.2	145381221.4	86494868.32	88231264.94	16052872.89	15004174.31	14419128.24	27078506.09	14557203.61	15990897.8	6.099613	2.608718	4.52E-07	1	1.279484	up
Com_3878_neg	N-Oleoyl Glycine	C20 H37 N O3	339.27705	10.231	338.2698	No results	No results	Full match	6317515.065	11787022.85	19449411.83	6608827.525	13770803.23	6211768.746	1838880.646	2787428.306	1496664.903	1987797.972	984200.983	1514953.11	6.045787	2.59593	4.99E-05	1	1.212552	up
Com_3745_neg	dopaquinone	C9 H9 N O4	195.05297	5.594	194.0456	No results	No results	Full match	20872534.89	24175315.34	18326787.12	14829467.77	12067728.65	15689679.16	3280615.812	3383661.881	3067316.251	2404114.028	3167094.833	2305960.42	6.017544	2.589175	2.18E-07	1	1.294336	up
Com_2682_pos	2-Phenylethylamine	C8 H11 N	121.08906	4.789	122.0963	No results	No results	Full match	22830310.76	42940936.1	26411917.3	17624466.83	63296998.95	72691572.97	3322111.674	8763630.222	7064299.188	7530932.715	2336962.262	12091643.8	5.979049	2.579916	0.000422	1	1.130724	up
Com_11707_pos	N1-[2-(2-pyridyl)ethyl]-2-aminobenzamide	C14 H15 N3 O	263.1014	2.25	264.1087	Invalid mass	No results	No results	4858118.003	5562084.469	3987290.541	4044165.746	5797021.735	6830472.131	707810.7442	721021.8355	914073.5059	1268414.334	871388.5385	806106.707	5.876392	2.554931	5.15E-08	1	1.293955	up
Com_9279_pos	N-[2-(4-methoxyphenoxy)ethyl]-2-furamide	C14 H15 N O4	261.10325	2.596	262.1106	Invalid mass	No results	No results	772851.7769	2095024.177	5561444.72	8325169.366	4479566.617	4870004.264	824623.1578	682160.7364	801412.0187	827165.1117	612003.544	730319.094	5.829814	2.54345	0.006845	0.917	1.070891	up
Com_10837_pos	6-methyl-7-nitro-2,3-dihydro-1,4-benzodioxine	C9 H9 N O4	195.05324	5.412	196.0605	Full match	No results	Full match	5387860.197	7955885.94	5902925.113	5737016.819	5868973.242	5211335.471	1060485.938	718809.6617	968879.1308	1006456.662	1415276.239	1031460.09	5.815491	2.539901	5.57E-08	1	1.300809	up
Com_9640_pos	(2S)-2-(2-hydroxypropan-2-yl)-2H,3H,7H-furo[3,2-g]chromen-7-one	C14 H14 O4	246.08468	1.375	247.092	Invalid mass	No results	No results	6255927.891	7014782.18	7241589.225	7675234.948	8445345.055	6401810.782	682282.9924	1201669.063	1295912.557	894262.0166	1657752.656	1680243.92	5.805987	2.537541	2.14E-05	1	1.280526	up
Com_1636_neg	1-(4-Methoxyphenyl)-2-propanone	C10 H12 O2	164.08358	5.498	163.0762	No results	No results	Full match	36919117.57	41478003.11	58998189.97	50119149.82	54262336.93	38581531.72	13758264.09	5991547.447	4628029.738	6651218.01	11665575.89	5904908.42	5.768744	2.528257	3.21E-05	1	1.256421	up
Com_655_pos	(2R)-2-[(2R,5S)-5-[(2S)-2-hydroxybutyl]oxolan-2-yl]propanoic acid	C11 H20 O4	216.13605	5.943	217.1432	Full match	No results	Full match	293828349.9	277206274.1	127514305.5	128090148.5	223061630	212514369	29733142.31	32540364.86	40520040.59	49684460.02	37469845.97	34480615.2	5.624131	2.49163	1.47E-05	1	1.264535	up
Com_1929_pos	PC (14:1e/4:0)	C26 H52 N O7 P	521.3477	9.629	522.3548	No results	Full match	Full match	31017445.38	67632103.6	88980225.99	25394396.53	64069949.7	39129746.58	15562784.15	7706973.148	6037896.684	13523992.74	8140735.542	5568465.32	5.592839	2.483581	8.59E-05	1	1.188448	up
Com_3796_pos	3,5-di(2-furylmethylidene)tetrahydro-2H-pyran-4-one	C15 H12 O4	256.07323	5.534	257.0805	Full match	No results	No results	28683751.98	39196821.46	36471670.23	34614077.19	36543501.01	43983451.75	9614422.982	5931377.123	6771947.635	6803495.007	3907903.326	6377588.22	5.569943	2.477663	2.43E-06	1	1.288307	up
Com_5194_pos	3-benzyl-4-hydroxy-5-(4-hydroxyphenyl)-2,5-dihydrofuran-2-one	C17 H14 O4	282.08901	5.296	283.0962	Full match	No results	No results	8028279.829	10981240.6	24988382.44	9885881.209	12189910.33	10519206.53	2281681.74	2759474.542	3082514.078	1911160.923	1204411.705	2569608.84	5.546652	2.471617	1.36E-05	1	1.232136	up
Com_11126_pos	Leukotriene B4 Ethanolamide	C22 H37 N O4	401.25629	5.237	402.2636	Invalid mass	No results	No results	6648445.483	5493197.807	2628806.585	2700901.924	3496277.181	2859497.619	467433.0535	471881.7206	431263.2337	523040.4054	2034237.447	373790.283	5.539072	2.469644	0.000221	1	1.177792	up
Com_2801_pos	2-(piperidinomethylidene)malononitrile	C9 H11 N3	161.09571	1.199	162.103	Full match	No results	No results	25591731.5	6727673.949	16030380.46	50899818.83	15449033.03	18243202.79	2909795.794	2855193.229	2854572.812	6450003.144	5681125.713	3359663.5	5.51389	2.46307	0.000981	1	1.12596	up
Com_10261_pos	Propionyl-L-carnitine	C10 H19 N O4	217.13117	4.953	218.1384	No results	No results	Full match	7435019.463	7006216.198	6693829.813	6211379.515	8114997.652	7011331.915	1018770.77	1178930.392	1075262.497	1923501.539	1241078.619	1295033.48	5.492706	2.457517	9.66E-07	1	1.30324	up
Com_5749_neg	5-Hydroxyindole-2-carboxylic acid	C9 H7 N O3	177.04227	4.799	176.035	No results	No results	Full match	4882964.293	12134739.7	4452999.399	2798079.792	3673903.903	4334535.263	1434368.75	820442.9548	1052467.558	810898.2508	847230.3638	911186.395	5.492505	2.457464	0.000189	1	1.21347	up
Com_5290_neg	Vitamin B2	C17 H20 N4 O6	376.13695	5.657	375.1297	No results	No results	Full match	12188628.35	12505438.14	4035509.057	4095446.61	2901940.605	6353624.626	1655705.893	1420834.654	930145.7301	1409797.793	1075398.302	1297719.11	5.402149	2.433533	0.001001	1	1.165959	up
Com_2231_pos	Epitestosterone	C19 H28 O2	288.20868	6.519	289.216	Full match	No results	Full match	78202605.1	70135793.36	31679635.81	29997922.45	40859185.88	40944729.6	9170322.247	6685152.126	8704435.221	14054544.06	10028197.29	5796367.46	5.360491	2.422365	1.79E-05	1	1.231643	up
Com_13742_pos	Gly-Phe	C11 H14 N2 O3	222.10042	4.908	223.1077	No results	No results	Full match	3123959.165	3719351.354	2874436.685	3368685.074	4049324.45	3934647.361	355784.3347	1182582.758	568326.4491	1021148.484	518927.8505	366429.209	5.250276	2.392393	0.000225	1	1.236327	up
Com_13099_pos	DL-Panthenol	C9 H19 N O4	205.13199	5.955	206.1389	No results	No results	Full match	2299073.412	5032975.576	1360352.536	1472297.013	2009155.082	2915362.356	393844.5838	459357.662	535753.4119	575600.6527	490629.2809	441844.012	5.208513	2.380872	0.000317	1	1.223223	up
Com_9193_pos	EKK	C17 H33 N5 O6	421.25479	6.601	422.2608	Invalid mass	No results	No results	7843358.218	7897379.375	3817545.205	8432919.62	4252554.462	5047677.932	695607.8462	1145874.084	1232846.918	1614291.992	1145597.065	1370428.36	5.176026	2.371845	6.58E-06	1	1.247062	up

Com_16197_pos	Pleuromutilin	C22 H34 O5	416.19598	5.842	417.2032	Invalid mass	No results	No results	1552094.102	564891.0919	721307.0542	1409178.143	2278686.908	1610766.609	214147.2883	259460.7383	277503.2063	245780.2833	439794.7676	142175.676	5.153664	2.365598	0.000208	1	1.172874	up
Com_2049_pos	All-Trans-13,14-Dihydroretinol	C20 H32 O	288.245	7.357	289.2523	No results	No results	Full match	20671991.5	17361856.85	58428435.73	73421084.67	19052793.64	19251895.75	5309579.17	4388575.863	7082732.177	15594196.95	4406209.429	3865448.42	5.121888	2.356676	0.000988	1	1.097289	up
Com_2270_neg	Epinephrine bitartrate	C13 H19 N O9	333.10571	5.279	314.0879	No results	No results	Full match	28937608.32	40477802.5	39621461.78	24620862.92	30952574.49	35877314.89	8258522.908	5526863.112	5108722.974	6182306.189	7261151.059	6862765.24	5.114437	2.354575	3.51E-08	1	1.296602	up
Com_2127_pos	LSD-d3	C20 H22 [2]H3 N3 O	304.24038	9.933	305.2481	Invalid mass	No results	No match	77152453.81	82228069.43	65500012.5	70317852.12	42805638.85	87626693.39	12524885.55	4850764.996	23236984.07	25354646.42	8366359.172	9433484.29	5.081119	2.345146	0.000506	1	1.185171	up
Com_6688_neg	Biotin	C10 H16 N2 O3 S	244.08787	5.452	243.0805	Full match	No results	Full match	7055624.156	7973552.508	6051178.732	6942249.272	6444106.618	6836369.603	1545329.809	1459523.727	1658857.19	783562.8327	1106659.418	1575455.51	5.080712	2.345031	1.10E-05	1	1.289453	up
Com_491_neg	16-Hydroxyhexadecanoic acid	C16 H32 O3	254.22426	9.825	253.217	Invalid mass	No results	No match	180976123	150334620.9	222214067.3	126265039.7	171590828.4	169369064.6	18288402.37	40590335.93	40732927.92	33457623.98	47320557.99	26078611.4	4.943853	2.305636	1.17E-05	1	1.262984	up
Com_714_neg	1-(1,8-dihydroxy-3,6-dimethyl-2-naphthyl)ethan-1-one	C14 H14 O3	230.09395	5.572	229.0866	Full match	No results	No results	115261409	150612812.1	145349999.2	101459406.6	131364588.9	151471531.8	41014860.97	28785253.47	41238092.24	9813469.454	10556095.21	30496443.6	4.913521	2.296757	0.000954	1	1.185992	up
Com_3188_neg	tetranor-PGDM	C16 H24 O7	310.14454	7.064	309.1373	Invalid mass	No results	No results	21315442.71	14664403.55	24870186.21	22634993.31	12099989.51	14838126.87	5280439.56	3187392.814	3484213.508	5588822.566	2934377.66	2891867.71	4.725579	2.240491	3.39E-06	1	1.252833	up
Com_498_neg	Valproic acid	C8 H16 O2	144.11484	5.999	143.1074	No results	No results	Full match	226518618.8	204477519.5	89461060.29	99208087.57	167476298.7	141465727.1	28494557.51	30870535.49	36172640.84	36288677.3	35491520.64	30394458.2	4.696758	2.231665	0.000108	1	1.254517	up
Com_10146_pos	APH	C14 H21 N5 O4	305.14669	1.36	306.1541	Invalid mass	No results	No results	5460657.468	5965909.579	5267643.992	5285495.185	8330691.214	6403446.675	1306010.916	915781.9328	1495323.571	1228882.297	1551767.592	1366896.83	4.668203	2.222867	4.74E-08	1	1.29475	up
Com_12306_pos	5-fluoro AB-PINACA N-(4-hydroxypentyl) metabolite	C18 H25 F N4 O3	386.18035	4.947	387.1876	Invalid mass	No results	No results	2954621.351	2340169.737	2137458.901	4475451.465	2327647.806	2245511.714	151424.4112	297799.9002	164501.892	2514198.85	321778.7612	135854.889	4.596455	2.200522	0.00394	0.889	1.100266	up
Com_2738_neg	23-Norcholic acid	C23 H38 O5	394.2719	7.062	393.2647	No results	No results	Full match	32226079.56	16828315.86	10827880.02	10104013.45	13436566.97	14336134.37	3904488.946	2254696.639	1591073.321	6048218.143	3927199.341	3748542.66	4.552389	2.186624	0.000161	1	1.169246	up
Com_1393_pos	Doxycycline	C22 H24 N2 O8	444.15672	5.924	445.1639	Invalid mass	No results	No results	48871385.88	45570688.2	83475380.37	115271294.7	61198935.36	66639408.82	45090782.32	15417092.4	6802604.429	10576762.47	1131677.476	14778108.7	4.488704	2.166299	0.010663	1	1.008407	up
Com_5769_pos	1-(3-methoxy-2-nitrostyryl)pyrrolidine	C13 H16 N2 O3	248.11602	4.873	249.1231	Full match	No results	Full match	13998143.66	21106708.38	21167082.56	18567262.45	10934268.38	7281870.771	3259907.335	2397397.617	1531478.167	2188378.015	9174315.315	2221739.33	4.479583	2.163364	0.000531	0.972	1.137159	up
Com_18102_pos	5'-Deoxy-5'-(Methylthio)Adenosine	C11 H15 N5 O3 S	297.08968	5.04	298.097	No results	No results	Full match	803907.3138	909939.7308	973269.9517	717043.3285	665467.3148	888104.074	254615.0608	145885.8548	175575.98	153362.9908	152579.4583	235733.871	4.435444	2.149079	8.19E-07	1	1.287484	up
Com_2767_neg	N-Tigloylglycine	C7 H11 N O3	157.07368	4.867	156.0665	Full match	No results	No results	31839832.39	20563247.02	17598231.16	25150011.84	15931985.41	14554748.78	14612315.17	2730112.335	1555118.238	1679039.4	5440619.058	2402886.23	4.420748	2.14429	0.002494	0.972	1.112823	up
Com_7513_neg	(+/-)7(8)-DiHDPA	C22 H34 O4	362.24531	7.071	361.238	Full match	No results	No results	5166942.299	5145676.62	5702208.728	5163507.976	6665082.381	6965636.372	1468044.208	960941.6642	1629707.687	1656576.557	1091244.607	1091539.39	4.407295	2.139893	8.32E-07	1	1.289643	up
Com_5756_pos	Mag (18:1)	C21 H40 O4	356.2914	9.294	357.298	No results	No results	Full match	7899896.756	3871009.138	7080058.818	9513899.401	22887794.78	23571211.45	3574686.321	2942889.918	3680102.864	2080496.941	2359643.499	2495468.68	4.367163	2.126697	0.005203	1	1.065046	up
Com_1389_pos	8-Isoprostaglandin F1α	C20 H36 O5	338.24291	9.32	339.2502	Invalid mass	No results	No results	85449477.92	152975068.4	39843189.46	34763140.18	107515097.2	96371563.98	20614703.1	24965297.15	17339727.35	14502392.35	20990932.36	20822914	4.335248	2.116115	0.001664	1	1.142623	up
Com_4875_pos	6,6-dimethyl-4-piperidino-5,6-dihydro-2H-thiine-2-thione	C12 H19 N S2	241.09459	1.358	242.1018	Invalid mass	No results	No results	18499496.34	32454462	23360832.14	23565466.08	27041583.6	23065800.04	12252782.29	4206179.689	4187275.327	3955504.069	4405871.691	5381979.26	4.303268	2.105433	0.000104	1	1.232316	up
Com_1029_pos	2,6-Di-tert-butyl-1,4-benzoquinone	C14 H20 O2	238.15689	5.991	239.1641	Invalid mass	No results	No results	186922602.2	172364770.3	130792333.6	116195986.7	96577965.72	94909591.92	30014631.1	23246374.38	35356422.62	42628332.21	27013598.64	28014884.7	4.282735	2.098532	3.26E-06	1	1.261635	up
Com_4281_pos	L-Dopa	C9 H11 N O4	197.0689	5.162	198.0762	Full match	No results	Full match	25006614.49	27871120.99	32689900.71	28865984.15	11801188.21	13168935.07	5475404.06	5230485.726	4917028.422	6136217.529	4461368.433	6409693.05	4.272231	2.09499	0.000314	1	1.219024	up
Com_9381_neg	Paracetamol	C8 H9 N O2	151.06299	4.731	150.0557	Full match	No results	Full match	4116022.628	4857778.517	3969349.883	2984987.341	4037633.657	3150846.181	1549736.482	757733.2029	865129.8944	880361.1167	657245.9358	762941.292	4.223642	2.078488	5.55E-06	1	1.265948	up
Com_7267_neg	3-(1,1,2,3,3,3-hexafluoropropyl)adamantane-1-carboxylic acid	C14 H16 F6 O2	330.11061	5.199	329.1033	Invalid mass	No results	No results	2524727.803	2382360.454	2732577.438	1859331.616	7020485.942	3243231.852	1091624.61	754276.155	684788.1263	762339.2114	620323.7289	773593.601	4.216545	2.076061	0.000343	1	1.197391	up
Com_1177_pos	PC (14:0e/2:0)	C24 H50 N O7 P	495.33419	9.661	496.3416	No results	Full match	Full match	57297499.03	64968515.12	105047995.3	136936022.8	93171119.42	97564242.28	13620347.37	15489569.59	27781639.87	44169108.71	14410928.47	17582834	4.171116	2.060433	0.000123	1	1.189608	up
Com_5957_pos	Cholecalciferol	C27 H44 O	384.33874	8.598	385.3458	Full match	No results	No results	7921114.36	9720643.873	17217212.99	17945007.39	17372896.94	15927146.34	3743764.657	3513658.157	3640656.486	5905322.291	1134950.656	2821374.83	4.147647	2.052293	0.000429	1	1.151322	up
Com_4274_neg	N'-(4-chlorophenyl)-4-ethylbenzohydrazide	C15 H15 Cl N2 O	274.08391	6.577	273.0766	Invalid mass	No results	No results	17258814.58	15941737.66	11387604.74	6957750.235	15551503.47	14212559.38	4061182.282	3366596.054	2125156.641	1141213.537	5424200.676	3667736.73	4.109452	2.038946	0.000469	1	1.153244	up
Com_9192_neg	Sorbic acid	C6 H8 O2	112.05233	4.864	111.0449	Full match	No results	Full match	3539537.182	3923755.847	4307307.102	3445316.111	3882175.681	4541514.101	1042472.677	878850.9724	871735.4798	999948.1785	1011593.026	958973.155	4.101554	2.036171	8.69E-10	1	1.315168	up
Com_17266_pos	N1-(3-pyridyl)-2,3,4,5,6-pentamethylbenzene-1-sulfonamide	C16 H20 N2 O2 S	304.11833	1.226	305.1256	Invalid mass	No results	No results	179730.6837	599412.7163	863935.1753	1348908.754	1002201.025	525671.0596	56998.45264	101087.0983	74838.87615	69621.25982	710503.4626	92225.2716	4.089355	2.031873	0.005022	0.917	1.001443	up
Com_3898_pos	Tetrahydroaldosterone	C21 H32 O5	364.2246	6.101	365.2318	No results	No results	Full match	30914821.06	44215379.33	24304807.49	30687211.57	30457646.06	25179971.4	7377505.281	6864508.425	6895764.918	9526667.311	8481395.545	6668755.82	4.054599	2.019559	4.35E-07	1	1.288833	up
Com_1240_neg	Kynurenic acid	C10 H7 N O3	189.04236	5.828	188.0351	Full match	Full match	Full match	85088655.43	80176667.47	53272918.52	59336171.29	79305828.51	76915027.31	55795239.65	4669970.057	10132441.4	5466581.134	24181684.5	7048231.77	4.045843	2.01644	0.005273	0.972	1.084893	up
Com_1551_neg	Docosahexaenoic Acid	C22 H32 O2	328.23977	9.912	327.2325	Full match	Full match	No results	50533783.99	57736176.16	62552090.85	47344267.39	51649954.19	59013492.53	13607322.24	11843550.96	16697797.32	20516410.69	8550383.319	10624012.9	4.017985	2.006472	3.81E-05	1	1.268466	up
Com_9344_pos	L-Homocystine	C8 H16 N2 O4 S2	268.05592	5.198	269.0631	No results	No results	Full match	7679853.778	10440938.51	9178518.753	3541984.07	5900615.94	10835775.13	2627540.226	1565280.097	1962065.741	2030615.91	1844745.29	1935891.4	3.976027	1.991327	0.00027	1	1.210131	up
Com_246_neg	Arachidonic acid	C20 H32 O2	304.23985	9.986	303.2325	Full match	No results	Full match	421725299.5	460362563	472568828.5	346690689.3	387063235.1	440852923.3	96238393.83	89276026.06	128532180.3	155964208.1	81086342.93	89189731.8	3.950204	1.981927	5.54E-06	1	1.282551	up
Com_2138_neg	D-Saccharic acid	C6 H10 O8	210.03734	1.514	209.03	Full match	Full match	Full match	19603679.3	14096374.17	31253977.5	25578942.46	44125341.83	28605909.17	2182179.446	11838993.43	3463080.461	5520028.043	9641842.308	8884602.99	3.931167	1.974958	0.001504	1	1.098201	up
Com_365_pos	Prostaglandin B1	C20 H32 O4	336.22971	6.816	337.237	Full match	No results	Full match	323498995	241992296.1	320831470.7	444173667	161212646.9	188526798.9	30616887.57	19418648.92	22231611.85	22690241.28	289469031	43406070.9	3.927322	1.973546	0.004526	0.917	1.066111	up
Com_541_pos	2-(2,6-dimethoxyphenyl)-5,6-dimethoxy-4H-chromen-4-one	C19 H18 O6	324.0994	5.594	325.1066	Invalid mass	No results	No results	307343609.9	343321124.3	305457580.7	307601706.8	329301093.7	361249591.4	73295827.54	88036077.43	85939556.01	92471313.39	66600459.66	94916548.1	3.898726	1.963003	7.09E-08	1	1.310395	up
Com_1386_neg	D-α-Hydroxyglutaric acid	C5 H8 O5	148.0371	1.434	147.0299	Full match	Full match	Full match	30187271.43	26084404.52	36303102.95	33435035.41	73891701.02	35276031.85	9947412.594	12550492.75	7826310.515	13382116.92	9156929.951	9478897.43	3.772368	1.91547	7.14E-05	1	1.222483	up
Com_4943_pos	L-Tyrosine methyl ester	C10 H13 N O3	195.08941	6.065	196.0967	Full match	No results	Full match	15176613.89	23533452.77	26897434.99	17243339.43	20021858.83	21323114.41	9573501.75	3833115.544	5183063.492	4053982.806	4643134.023	5798427.62	3.753815	1.908358	2.16E-05	1	1.241253	up
Com_15679_pos	Kaempferitrin	C27 H30 O14	278.09682	5.568	579.1829	Invalid mass	No results	No results	1461623.336	1950483.376	2428511.983	1771800.698	1423944.382	2292287.941	508877.9087	470524.6757	649121.3443	530208.3629	321888.2504	559293.73	3.726635	1.897874	1.79E-06	1	1.261949	up
Com_6296_pos	3-(4-hydroxy-3-methoxyphenyl)propanoic acid	C10 H12 O4	218.05769	5.299	219.0649	Invalid mass	No results	No results	16941645.15	21577115.56	16673729.65	16470099.88	18023924.29	16888797.07	5533547.873	5088100.475	5366972.066	4084979.236	3569239.555	5066476.05	3.712221	1.892282	2.06E-07	1	1.299746	up
Com_1754_neg	Homogentisic Acid	C8 H8 O4	168.04209	5.588	167.0348	No results	No results	Full match	31393090.27	32699879.86	33438947.58	48217959.08	20965718.35	21701856.98	7733737.8	10541544.44	7381492.128	7328993.986	8791069.896	9151217.72	3.699679	1.8874	3.62E-05	1	1.252602	up
Com_10961_pos	1-Methylguanosine	C11 H15 N5 O5	297.10742	4.752	298.1147	No results	No results	Full match	5899330.293	3478939.995	4331885.664	4528040.629	7015245.443	7625653.222	655650.565	2382048.877	1690848.882	1286077.72	959916.3239	1927701.21	3.697086	1.886389	0.000303	1	1.166633	up
Com_15407_pos	Melatonin	C13 H16 N2 O2	232.1203	5.309	233.1276	No results	No results	Full match	1522819.186	2021913.507	2596987.734	1669309.576	1683149.451	1911217.4	616588.7324	437268.8175	222121.845	378036.1584	949936.0549	483629.459	3.693959	1.885168	0.000455	1	1.191391	up
Com_3938_pos	1-(4-hydroxyphenyl)propane-1,2-diol	C9 H12 O3	190.06016	5.665	191.0674	Invalid mass	No results	No results	38009349.44	38692516.97	32078528.39	33006047.85	23378667.96	34811036.08	9458682.584	8149049.39	9799918.79	9872547.343	7477113.739	9554631.06	3.681992	1.880487	2.75E-07	1	1.294921	up
Com_2667_pos	TMK	C15 H30 N4 O5 S	189.09717	1.356	190.1045	Invalid mass	No results	No results	63586210.91	21551867.47	40074358.49	34704383.15	29105142.99	53515557.84	8581317.741	7150518.257	9440189.023	12946323.35	15630201.32	12297807.8	3.672232	1.876657	0.000125	1	1.185089	up
Com_1836_pos	Stiripentol	C14 H18 O3	216.1148	5.692	217.1221	Invalid mass	No results	No results	98073704.93	84264610.59	80272332.66	80182821.46	73617488.13	79540368.53	22745696.53	20535999.62	23721381.44	28300571.72	20941072.39	19613601.5	3.650504	1.868096	9.89E-09	1	1.307476	up
Com_1030_pos	Alloxan	C4 H4 N2 O5	160.0127	1.031	161.0201	No results	No results	No results	186761958.6	196551444.8	121244784.8	98665152.21	144883336.2	111639098.7	39525825.61	32919558.16	54574093.78	51739047.1	36085904.39	21823768.8	3.632705	1.861044	3.04E-05	1	1.216009	up
Com_872_neg	trans-10-Heptadecenoic Acid	C17 H32 O2	268.2399	10.209	267.2325	Full match	No results	Full match	94645267.49	105625392.6	120351911.6	69687819.61	106848056.2	93166561.96	19606761.84	30702782.35	22244609.12	19064897.6	50826723.27	21560900.1	3.599396	1.847755	8.75E-05	1	1.226943	up
Com_1299_pos	3-methoxy-2-phenyl-4H-furo[2,3-h]chromen-4-one	C18 H12 O4	292.07326	5.592	293.0806	Full match	No results	Full match	135111534.2	134051267.8	126378841	123796183.5	141476481.5	150014766.7	33396737.42	36444784.45	40887032.7	39835743.21	29611083.11	45996563.9	3.585012	1.841978	3.77E-07	1	1.305311	up
Com_5483_pos	N6-Me-Adenosine	C11 H15 N5 O4	281.11242	4.783	282.1196	Full match	Full match	Full match	19028820.18	16662544.81	22561410.67	20525544.26	16457063.21	14469253.13	3942011.145	5933367.425	8284324.111	5660588.887	2015377.297	5131225.55	3.542643	1.824826	0.000612	1	1.189221	up
Com_132_pos	Phenylpropiolic acid	C9 H6 O2	146.03741	1.037	147.0447	No results	No results	Full match	939868373.6	1048586998	823691127	751002453.8	903079874.6	717507534.8	199684549.9	179639070.3	273460786.4	396941876.3	228644717.4	186129436	3.539594	1.823584	2.53E-05	1	1.258002	up
Com_601_neg	Benzyl cinnamate	C16 H14 O2	238.09908	5.64	237.0917	Full match	No results	Full match	176307149.4	189516217.5	178823851.6	143192050.7	178245139.5	185163151.6	60037840.25	54228640.61	46752927.57	55758981.78	33478978.51	49647633.8	3.505269	1.809525	2.25E-06	1	1.289464	up
Com_5590_pos	Sedanolide	C12 H18 O2	194.13054	6.71	195.1378	Full match	No results	Full match	23466626.98	20892256.47	17740307.99	17169859.39	19152223.8	17436275.48	5478360.927	5150225.037	5424999.781	7522630.868	4580448.498	5082351.49	3.485589	1.801402	1.12E-07	1	1.295305	up
Com_5018_neg	5-Phenyl-N-(4-(trifluoromethyl)phenyl)oxazol-2-amine	C16 H11 F3 N2 O	304.07895	1.424	303.0718	Invalid mass	No results	No results	6917602.57	7188988.594	10103431.28	11304785.15	8245010.041	7945509.691	2575743.238	3958856.241	2020802.639	2026954.41	1025886.178	3230707.41	3.484433	1.800924	0.000482	1	1.186678	up
Com_7802_pos	Ecgonine	C9 H15 N O3	185.1024	4.936	186.1096	No results	Invalid mass	No results	11767467.62	8704407.407	9610256.419	11465714.28	11134271.14	9438653.433	2228988.151	1879290.954	2339189.289	3095405.902	6858732.785	2073437.05	3.362416	1.749498	0.000769	1	1.192824	up
Com_4193_pos	N'-hydroxy-4-(trifluoromethoxy)benzenecarboximidamide	C8 H7 F3 N2 O2	220.04837	5.297	221.0556	Invalid mass	No results	No results	35192646.32	35678674	27908056.42	28035563.94	28356836.14	31918517.53	10953958.78	6786910.088	9611911.258	9152650.036	10146798.83	9231621.29	3.347842	1.743231	1.31E-07	1	1.295615	up
Com_5094_neg	Dl-3-Hydroxy-kynurenine	C10 H12 N2 O4	224.07935	5.076	223.0718	No results	No results	Full match	10519854.37	9874886.755	9138407.728	10729176.18	11490891.18	14002498.34	4974573.504	3301673.527	670814.3351	1864945.839	5476794.981	3478825.43	3.326434	1.733977	0.006739	1	1.065581	up
Com_5783_pos	N-lactoyl-phenylalanine	C12 H15 N O4	237.09965	5.308	238.1071	No results	No results	Full match	21644292.03	17885596.73	21115395.54	14458276.18	19296452.18	17623361.62	11034696.49	3220747.083	1364132.216	7127127.66	5769443.96	5325456.37	3.310227	1.72693	0.00507	1	1.088277	up
Com_6569_neg	Sorbitan monopalmitate	C22 H42 O6	402.29769	8.109	401.2903	Full match	No results	Full match	8418327.201	7602260.886	6347204.952	6458261.685	4826220.863	4857126.077	1864746.816	1477262.561	2217249.4	2691699.545	1796173.058	1718258.88	3.273109	1.710662	3.04E-06	1	1.255344	up
Com_8022_pos	Isoproterenol	C11 H17 N O3	211.12044	1.353	212.1276	No results	No results	Full match	11904556.84	13577820.77	9956475.142	9957494.536	12958572.42	11531578.54	2291746.312	3147855.492	3438638.37	5138500.269	3598689.031	3761279.16	3.269282	1.708974	1.49E-05	1	1.264391	up

Com_6242_pos	7-{[(2E)-3,7-dimethylocta-2,6-dien-1-yl]oxy}-2H-chromen-2-one	C19 H22 O3	320.13703	4.726	321.1444	Invalid mass	No results	No results	11804378.99	9782421.484	9168158.874	10248705.84	8926915.533	11287183.29	1661024.142	1294543.707	1285031.158	12532757.99	797724.478	1196058.44	3.261966	1.705742	0.007298	0.833	1.070458	up
Com_5893_neg	5-Methoxyindole-3-Carbaldehyde	C10 H9 N O2	175.06309	5.228	174.0557	No results	No results	Full match	10097447.37	3849429.186	3411985.751	2816974.984	2558788.834	1882645.121	1268942.063	909173.741	1095508.48	1048831.732	1845284.048	1386512.21	3.25873	1.70431	0.004992	1	1.047071	up
Com_1494_pos	Methyl EudesMate	C11 H14 O5	226.08388	5.702	227.0913	No results	No results	Full match	111065050.9	110935593	119533346.9	93365851.19	95135727.28	105502366.1	37361678.16	30254455.04	30256563.85	29372879.97	27219850.02	41331254.9	3.245908	1.698622	2.35E-07	1	1.297966	up
Com_2505_neg	Citric acid	C6 H8 O7	192.02688	2.233	191.0196	Full match	Full match	Full match	20104358.64	13467019.94	20801669.74	21515091.3	36369018.11	28525156.8	6739833.429	12251503.41	6724329.631	5113760.325	5323945.916	7502300.53	3.224834	1.689225	0.000104	1	1.182344	up
Com_10451_pos	2-Deoxyuridine	C9 H12 N2 O5	228.07427	1.352	229.0816	No results	No results	Full match	4972664.181	7109865.512	6595386.122	6503582.863	7324708.928	7543271.876	1545497.199	1669988.325	1733181.685	1500880.273	3586347.917	2404387.99	3.219338	1.686764	9.73E-05	1	1.23206	up
Com_1338_neg	Erythronolactone	C4 H6 O4	118.02663	2.683	117.0193	No results	Full match	Full match	54227310.08	48797127.54	46402664.39	46317706.47	77591584.93	56645717.99	11964409.75	17026233.91	14061407.72	31529608.23	12949734.77	15999493.1	3.187282	1.672327	8.76E-05	1	1.217329	up
Com_3283_neg	N-Acetyl-DL-glutamic acid	C7 H11 N O5	189.06356	1.452	188.0563	Full match	Full match	Full match	25248793.29	13640893.1	12195991.9	12465481.71	23975415	14858049.4	3990020.272	5469759.755	4706522.004	5962784.203	3810995.686	8225981.05	3.183001	1.670388	7.89E-05	1	1.189724	up
Com_7651_pos	(5-L-Glutamyl)-L-Amino Acid	C8 H14 N2 O5	218.08986	1.361	219.097	No results	No results	Full match	8162867.685	9688503.771	9370644.858	11894766.77	12473427.77	12303006.92	1967649.087	2592046.169	1790801.797	2924676.281	7256901.262	3630472.33	3.168906	1.663985	0.001078	1	1.161176	up
Com_1459_neg	3-[4-methyl-1-(2-methylpropanoyl)-3-oxocyclohexyl]butanoic acid	C15 H24 O4	268.16717	6.496	267.1598	Full match	No results	No results	59589201.99	70727383.9	58045122.3	59168503.53	44193552.38	55964864.14	27609651.62	10497041.31	22821368.72	25236557.15	14076727.19	9627850.9	3.164569	1.662009	0.000792	1	1.179942	up
Com_17376_pos	(2R,3S,4S,5R,6S)-2-(hydroxymethyl)-6-phenoxyoxane-3,4,5-triol	C12 H16 O6	273.12074	6.005	274.128	Invalid mass	No results	No results	1516943.299	1184396.872	1100757.272	1041801.734	965582.927	1069469.824	393258.7246	303935.3564	336241.6677	505645.2194	334725.6861	314879.966	3.142959	1.652123	6.19E-07	1	1.277163	up
Com_8564_pos	Pro-Leu	C11 H20 N2 O3	228.14707	1.357	229.1544	No results	No results	Full match	10330682.12	12028904.32	8585905.324	8518283.317	11241297.79	9751671.314	1682321.098	2689883.391	2936276.455	4616207.78	4343621.181	3121089.18	3.118031	1.640635	0.000225	1	1.218311	up
Com_18_pos	5,6-dimethoxy-2-(2-methoxyphenyl)-4H-chromen-4-one	C18 H16 O5	136.0534	5.703	295.096	Invalid mass	No results	No results	9126167976	9042092836	7995601295	8460435909	7888814471	8630387928	3088142360	2365688245	2842399572	2879739020	2408997297	3061543732	3.072326	1.619331	6.13E-08	1	1.30979	up
Com_8556_pos	Celastrol	C29 H38 O4	450.27458	6.045	451.2819	Invalid mass	No results	No results	9079254.382	8973607.618	4697974.038	9678145.876	9176130.082	6669393.129	2549914.739	1756387.561	2331546.479	4317419.763	3004325.858	1778978.33	3.067273	1.616957	9.95E-05	1	1.186106	up
Com_13186_pos	L-(-)-alpha-Amino-epsilon-Caprolactam	C6 H12 N2 O	128.09492	1.348	129.1021	No results	No results	Full match	3509885.912	4955201.523	1601312.253	1941390.472	3111218.698	2979459.589	698746.9239	868503.8837	861249.5131	719262.3054	1627094.739	1138649.21	3.060531	1.613782	0.000503	0.972	1.130023	up
Com_3900_pos	16α-Hydroxydehydroepiandrosterone	C19 H28 O3	321.23586	8.542	322.2431	Invalid mass	No results	No results	38545083.86	38517650.46	30652982.4	29710264.78	40117611.12	40151517.18	13109583.51	7498538.727	15695956.37	11450127.21	15041006.99	8837057.14	3.039065	1.603627	5.61E-05	1	1.242218	up
Com_3149_neg	2-[(carboxymethyl)(methyl)amino]-5-methoxybenzoic acid	C11 H13 N O5	193.07365	5.248	192.0665	Invalid mass	No results	No match	27049623.26	27034757.16	18732795.44	22525638.21	24991728.39	25312934.99	5276817.808	5693642.868	8133678.966	5541170.765	14948823.39	8525224.54	3.026796	1.597791	0.000419	1	1.201501	up
Com_11033_pos	Hydrocortisone acetate	C23 H32 O6	404.21838	5.75	405.2258	No results	No results	Full match	6558929.418	7554815.316	2582208.514	5754247.208	4554641.477	6334315.484	1816004.904	1640289.48	1726214.256	2538019.102	1799912.91	1552165.28	3.010959	1.590223	0.000498	1	1.177907	up
Com_1398_pos	alpha-Benzylsuccinic acid	C11 H12 O4	208.07343	5.716	209.0806	No results	No results	Full match	127005312.2	113927930.8	128483341.4	101120927.1	115241702	110185603.8	45872851.1	37664236.74	40508246.14	36222372.6	30875920.22	42950025.2	2.973019	1.571929	1.11E-07	1	1.299922	up
Com_5876_pos	Cholest-4-en-3-one	C27 H44 O	384.33906	8.376	385.3462	Full match	No results	No results	13389709.14	15158323.17	18345560.17	18339376.7	20786756.86	18943508.89	6062603.7	5245382.516	7877534.336	8920950.867	2520417.588	4730394.14	2.968645	1.569805	0.000874	1	1.168343	up
Com_7858_pos	Cinnamoylglycine	C11 H11 N O3	205.07368	5.688	206.0808	No results	No results	Full match	9987242.177	8546948.764	9351348.746	11294742.78	11545037.25	9267451.107	3552360.061	4072469.847	3286442.091	3630936.183	2436523.15	3274720.47	2.962101	1.566621	5.49E-07	1	1.285867	up
Com_792_neg	Hydrocinnamic acid	C9 H10 O2	150.06788	6.299	299.1286	Full match	No results	Full match	104790534	118152026.6	97454238.86	116545648	99391143.17	92774549.4	29560673.79	29688282.11	45848609.11	49609002.44	29636517.46	28695504.6	2.953024	1.562193	3.42E-05	1	1.263521	up
Com_3578_pos	N6-Acetyl-L-lysine	C8 H16 N2 O3	188.11725	7.88	189.1245	Invalid mass	No results	No results	20056009.67	21546797.12	32242809.58	37693742.19	24246002.75	17415696.85	7819776.413	6170192.979	9561400.037	12670391.43	8937965.323	6729916.77	2.95244	1.561908	5.81E-05	1	1.197785	up
Com_6833_pos	N'-{6-[(5-chloro-3-pyridyl)oxy]-3-pyridyl}-N,N-dimethyliminoformamide	C13 H13 Cl N4 O	276.07781	5.696	277.085	Full match	No results	No results	14689306.89	16148792.17	14136213.5	13806120.09	17289058.28	13782470.91	6941765.466	2607030.79	6134715.147	5373859.832	4085601.782	5386134.98	2.943157	1.557365	0.000358	1	1.221845	up
Com_7150_pos	Ala-Gln	C8 H15 N3 O4	217.10605	1.362	218.1134	No results	No results	Full match	10272834.33	15774677.64	13031163.57	13358819	14486085.48	13240586.91	2718350.734	3075377.869	3157924.248	4916260.05	9652784.968	4028203.7	2.909886	1.540963	0.001147	1	1.16512	up
Com_6424_neg	2-Phenylglycine	C8 H9 N O2	151.06323	5.266	150.0558	No results	No results	Full match	7779964.251	8503231.198	7452692.937	7445573.006	7542571.497	6971474.497	2931102.73	2778399.541	2744603.382	2962957.682	2261351.924	2028290.51	2.909299	1.540672	1.50E-06	1	1.298458	up
Com_13506_neg	2-Hydroxy-6-Aminopurine	C5 H5 N5 O	151.04904	5.499	150.0419	No results	No results	Full match	1550742.127	750313.8189	923895.4192	1119483.481	1333288.976	924490.4215	384892.792	390358.3319	384290.0549	441001.5744	268287.2111	404922.202	2.903665	1.537875	2.82E-05	1	1.233547	up
Com_2452_neg	2-Isopropylmalic acid	C7 H12 O5	176.06824	4.87	175.061	Full match	Full match	Full match	32530695.92	42517390.61	29560148.08	12260533.18	37031251.02	12306505.49	6868404.693	11469357.42	6873329.51	10089911.74	8838392.759	13226483.7	2.897306	1.534712	0.00563	0.944	1.027992	up
Com_1861_pos	7α-Hydroxytestosterone	C19 H28 O3	304.20353	6.722	305.2108	Full match	No results	Full match	73112083.95	85076338.63	73656140.29	82154116.95	63077613.96	76078136.6	28138758.51	18356302.29	21735228.63	26146301.43	38384720.5	24058339.4	2.889653	1.530896	3.32E-05	1	1.260239	up
Com_8553_pos	Boc-beta-cyano-L-alanine	C9 H14 N2 O4	214.09501	1.366	215.1024	No results	No results	Full match	7222498.359	10518866.43	8466968.495	8118589.05	10095470.24	9572379.037	2213414.8	2081726.755	2101141.135	1987038.197	7459332.902	2848767.88	2.888746	1.530443	0.00172	0.972	1.147683	up
Com_1853_neg	1-Caffeoylquinic Acid	C16 H18 O9	354.09476	5.649	353.0879	No results	No results	Full match	38727186.02	46861924.82	50957391.57	38583624.06	39182353.42	15519087.88	19292924.65	5293455.227	11026253.59	13123820.08	12630707.1	18535644	2.876389	1.524259	0.002037	0.944	1.053311	up
Com_6856_pos	Ecgonine methyl ester	C10 H17 N O3	199.12058	1.37	200.1278	Full match	No results	No results	12404143.58	13873539.83	15604761.51	14279509.52	13012902.82	10637658.45	4221773.141	4627177.65	4454869.156	5276505.285	5285755.265	4016044.46	2.862498	1.517274	5.26E-08	1	1.295169	up
Com_13125_pos	Choline bitartrate	C9 H19 N O7	253.11632	1.352	254.124	No results	No results	Full match	2517255.262	3495869.348	3526481.715	3785230.357	3802660.879	3710784.323	1078829.397	1027547.149	1041031.67	1227546.23	1521968.784	1395344.83	2.857586	1.514797	5.11E-07	1	1.275876	up
Com_9193_neg	LPE 12:0	C17 H36 N O7 P	397.22243	7.894	396.2152	No results	Full match	No results	4445301.211	1347620.81	1804404.909	974949.854	2169458.634	1915438.526	1212759.997	645417.6793	514353.9677	627441.1288	730267.3368	736734.717	2.8335	1.502585	0.003957	0.972	1.041002	up
Com_2680_pos	L-Tyrosine	C9 H11 N O3	181.07125	5.926	182.0786	Invalid mass	Invalid mass	No results	38327353.54	58312345.23	59992397.56	38674622.26	56950792.79	65504844.42	31380397.13	13723054.94	19396423.42	16739003	13261560.13	20834696.9	2.755122	1.462116	0.000111	1	1.188323	up
Com_4455_pos	Kahweol	C20 H26 O3	314.18789	6.613	315.1951	Full match	No results	Full match	32479163.03	28603190.56	25073104.79	19337560.05	28509135.35	30741348.29	11619521.16	6877083.668	10297739.12	10675270.22	10745836.7	9788021.75	2.745566	1.457104	2.81E-06	1	1.255721	up
Com_10576_pos	D-Glucuronic acid	C6 H10 O7	194.04261	0.982	195.0501	No results	No results	Full match	6215401.465	6296859.318	3555519.731	4723462.681	5059426.332	6501868.474	2043459.688	1764659.449	2738376.015	2281330.009	1574597.402	1413837.4	2.737968	1.453105	2.56E-05	1	1.216285	up
Com_821_neg	19(R)-Hydroxy-prostaglandin E2	C20 H32 O6	350.20918	6.667	349.2019	Invalid mass	No results	No results	131439731.1	130361029.4	91351434.56	72517879.19	106216445.6	95881004.34	111831430.7	23724686.05	27146493.5	24945777.58	21035721	21674092.7	2.72518	1.446351	0.004417	0.889	1.07128	up
Com_6176_neg	4-Butylresorcinol	C10 H14 O2	166.0991	5.864	165.092	No results	No results	Full match	9142902.476	8515603.437	8650981.518	7400066.008	8685975.517	7256539.478	1885946.428	3044619.805	3334828.466	3499312.947	3317991.301	3162049.27	2.721444	1.444373	2.93E-05	1	1.263409	up
Com_1522_neg	Syringic acid	C9 H10 O5	198.05251	5.352	197.0451	No results	No results	Full match	54444551.45	64747694.72	63825851.18	50939300.4	46030397.35	44928480.63	30169521.22	16000013.41	24083571.96	15101460.72	14954429.88	19220980	2.718283	1.442696	6.76E-05	1	1.225492	up
Com_7887_pos	13,14-dihydro-15-keto-tetranor Prostaglandin E2	C16 H26 O5	320.16015	6.72	321.1672	Invalid mass	No results	No results	6313456.13	6705932.51	6045902.833	5247298.635	5553092.146	15227000.54	2968257.553	2583484.087	2927420.729	3349213.349	2796183.655	2033801.59	2.70691	1.436647	0.001213	1	1.138764	up
Com_2592_neg	Pyrogallol	C6 H6 O3	126.03144	5.153	125.024	Full match	No results	Full match	33918968.94	39582365.54	16596028.93	10302757.02	11884122.86	15408761.13	7988759.842	8803104.773	7034827.044	7259748.085	5925072.103	10164343.7	2.706745	1.436559	0.009882	1	1.00536	up
Com_9852_neg	Methyl 3-indolyacetate	C11 H11 N O2	189.07865	5.582	188.0716	No results	No results	Full match	2335201.104	1694252.765	1400420.614	3269541.548	1489353.332	993883.5864	653275.7388	601238.3927	735214.805	731501.881	525134.6386	888527.035	2.70446	1.435341	0.001622	1	1.121328	up
Com_8308_pos	2,6-di(2-furyl)-3-propyltetrahydropyridin-4(1H)-one hydrochloride	C16 H19 N O3	273.13199	1.345	274.1392	Invalid mass	No results	No results	6628444.65	9301280.528	9639794.309	5904084.154	12256918.01	6799700.627	1813697.002	2641940.02	3239223.047	3742284.313	4315071.919	3001433.8	2.694421	1.429975	0.000152	1	1.168142	up
Com_5536_neg	5-Hydroxyindole	C8 H7 N O	133.05279	5.239	132.0455	No results	No results	Full match	11335421.27	12664208.82	9721822.338	7564964.328	10484321.24	9882517.768	2927561.703	3257589.024	4288063.823	4343180.528	3637524.306	4447698.76	2.692092	1.428728	1.78E-06	1	1.261672	up
Com_6264_pos	2-oxo-2H-chromene-3-carboxylic acid	C10 H6 O4	190.02952	1.023	191.0367	Invalid mass	No results	No results	13918148.6	15955311.9	11551637.91	11676648.37	13431991.89	16057984.92	4934270.439	4543355.853	7048894.035	6194337.022	4855876.697	3461781.33	2.660943	1.411938	2.80E-05	1	1.241473	up
Com_1048_neg	Phloretin	C15 H14 O5	274.08404	5.572	273.0768	Full match	No results	No results	52962879.13	99208843.16	90439960.4	58787901.51	100435445.6	104338042.3	40859908	34027104.05	37069436.81	13316781.35	35391156.45	30324474.7	2.650275	1.406142	0.000919	1	1.109788	up
Com_8762_pos	D-Glucarate	C6 H10 O8	210.03743	0.993	211.0448	No results	No results	Full match	8311262.132	10461331.58	7623820.702	9292292.775	7638616.976	10516083.71	3832651.241	2184540.422	4416231.358	4637000.19	2915065.961	2382029.42	2.643592	1.402499	0.000213	1	1.20727	up
Com_790_pos	α-Aspartylphenylalanine	C13 H16 N2 O5	280.10963	5.652	281.1168	Invalid mass	No results	No results	222692950.2	222536288.1	139275355	143065734.9	236305880.9	238961162.6	57694315.9	74084502.63	73807379.37	114819317.5	61961451.46	75811906.7	2.625257	1.392459	4.98E-05	1	1.200957	up
Com_4570_neg	5-Aminoimidazole-4-carboxamide-1-beta-d-ribofuranosyl 5-monophosphate	C9 H15 N4 O8 P	338.06326	5.998	337.0559	No results	No results	Full match	10861941.11	12891063.78	14554404.44	13202583.15	12294601.27	13658651.07	6819386.019	4108302.959	5099820.612	3564594.147	4779761.027	5141817	2.624655	1.392128	2.34E-05	1	1.26242	up
Com_12544_pos	Val-Ser	C8 H16 N2 O4	204.11065	1.218	205.1179	No results	No results	Full match	3638775.126	2942848.62	3638352.121	2830209.065	5121969.515	3837045.221	496818.74	1082312.501	1781070.894	1325798.569	1479855.489	2223658.64	2.623417	1.391447	0.003102	1	1.086242	up
Com_4785_neg	7-Methylxanthine	C6 H6 N4 O2	166.0488	3.085	165.0415	Full match	No results	Full match	9539913.743	9638017.446	13227167.97	12158579.93	12327364.91	9579811.241	3489758.695	3087734.019	4374411.308	5367843.843	5258988.532	3921266.64	2.6067	1.382224	1.12E-05	1	1.247502	up
Com_227_neg	Bisphenol A	C15 H16 O2	228.1148	5.69	227.1074	Full match	No results	Full match	427115576.3	398021898.4	413570589.2	442830459.3	400255607.9	417123330.7	184245277.1	153320714.4	171918561.5	148634153.7	123025775.8	177554577	2.606571	1.382153	7.37E-06	1	1.297445	up
Com_9533_pos	methyl 3-(1H-indol-3-yl)-2-[(phenylmethylene)amino]propanoate	C19 H18 N2 O2	306.14219	1.339	307.1495	Invalid mass	No results	No results	6260680.385	8455596.385	6648783.976	6081635.772	9442300.676	6187213.183	2168871.298	3933568.505	2142486.168	2769200.23	1960050.329	3802122.66	2.567683	1.360467	0.000139	1	1.195981	up
Com_7516_pos	JNJ-1661010	C19 H19 N5 O S	365.1316	1.349	366.1389	No results	No results	Full match	7823873	6069690	10646087.88	7899474.751	14656860.59	15430172.07	2206865.055	5858352.776	2264673.661	5915059.46	4404797.207	3720356.51	2.565691	1.359347	0.002365	1	1.046134	up
Com_13152_neg	S-Adenosyl-L-methionine	C15 H22 N6 O5 S	398.13893	6.254	397.1317	No results	No results	Full match	1700853.918	643185.0533	989971.0437	935957.539	489113.339	441839.386	207775.7246	336956.9446	425076.5249	408058.7555	347931.423	303378.053	2.563068	1.357872	0.006861	1	1.006965	up
Com_1007_neg	Prostaglandin D1	C20 H34 O5	336.2299	6.692	335.2227	Invalid mass	No results	No match	84121340.15	72233090.16	94449745.42	90061220.2	73294008.7	86040186.79	23436810.79	58179333.99	22504901.29	35263943.89	27997906.28	28748650.4	2.550327	1.350682	0.000589	1	1.195319	up
Com_5417_neg	3-Methoxytyramine	C9 H13 N O2	167.09452	5.204	166.0871	No results	No results	Full match	10691908.06	12228665.53	8766869.592	10030610.61	8908043.883	8147287.998	8685647.421	3422695.289	2434590.339	3106939.07	2995415.399	2545908.34	2.534297	1.341586	0.002132	0.972	1.127965	up
Com_7128_pos	Cortodoxone	C21 H30 O4	346.214	5.72	347.2216	No results	No results	Full match	15807980.85	13005812.56	12298553.62	11596395.39	14037181.14	16839343.9	6939558.97	4206054.778	4436613.893	6866712.628	5440116.663	5153483.92	2.529626	1.338924	9.96E-06	1	1.249562	up

Com_8_neg	Phenylpyruvic acid	C9 H8 O3	164.04714	5.657	163.0401	No results	No results	Full match	6795970203	6468418822	6342998441	6874292960	7030067673	7329959950	2729911776	2791219653	2698263448	2563535589	2216150065	3220975334	2.517976	1.332264	7.03E-07	1	1.302864	up
Com_8920_pos	4-Hydroxyretinoic Acid	C20 H28 O3	316.20378	5.581	317.2111	No results	No results	Full match	9489138.387	10090062.71	7397634.677	8685379.047	10806370.59	9077663.914	4185006.078	2883856.612	4454318.184	5717075.877	1589590.071	3442859.29	2.493916	1.318413	0.002209	1	1.130727	up
Com_11274_neg	2-(3,4-dimethoxyphenyl)ethanamine	C10 H15 N O2	181.11029	5.494	180.1031	No results	No results	Full match	2122548.969	2408327.572	2662034.241	1811680.206	1627957.97	2091599.064	824215.9834	878079.5502	952583.4424	917578.4954	637908.0817	896582.059	2.491537	1.317036	3.14E-06	1	1.258306	up
Com_12590_pos	Irganox 259	C40 H62 O6	660.44491	9.031	661.4529	Invalid mass	No results	No results	2846674.11	4033520.987	2361936.33	4219612.37	4500169.155	2613042.874	1063006.736	1380415.733	1398585.699	1630489.36	932888.8087	1866739.53	2.487263	1.314559	0.000164	1	1.165405	up
Com_4638_pos	Carbaprostacyclin	C21 H34 O4	332.23475	8.799	333.242	Invalid mass	No results	No match	22807958.9	23119106.09	21453280.82	26234114.82	17105918.99	20021637.25	10805645.22	8716528.842	9153143.463	5892758.773	8065452.57	10137263.1	2.477545	1.308911	1.35E-05	1	1.245461	up
Com_969_neg	2,3-Dinor-11β-prostaglandin F2α	C18 H30 O5	362.19358	6.054	361.1865	Invalid mass	No results	No results	95946563.71	96318825.62	82239852.61	93403024.3	83684171.66	76380389.61	43426747.58	35228895.09	28586831.66	40433346.31	27215634.83	41153661.1	2.443808	1.289131	1.66E-05	1	1.263664	up
Com_14482_pos	Ala-trp	C14 H17 N3 O3	275.12703	5.045	276.1343	No results	No results	Full match	3095518.515	2491674.307	2040725.601	2017349.733	3279556.193	2619219.716	465384.5798	795775.2944	1347595.794	2149660.776	890537.8271	723013.563	2.439442	1.286551	0.004354	0.944	1.068505	up
Com_4326_neg	N-Acetylvaline	C7 H13 N O3	159.08934	4.981	158.082	Full match	Full match	Full match	11007011.23	6667403.37	10362619.38	14465522.66	15261049.17	16512509.06	5278220.082	5135176.343	5451268.739	6454460.246	3018577.691	5336954.99	2.421416	1.275851	0.000662	1	1.11909	up
Com_6910_pos	4-acetyl-4-(ethoxycarbonyl)heptanedioic acid	C12 H18 O7	296.08873	5.233	297.0956	Invalid mass	No results	No results	16606561.32	16882872.35	8143247.032	8132335.889	9086910.726	7819895.07	4517614.713	3553782.995	4552891.614	4444690.036	6292848.796	4352806.16	2.405654	1.266429	0.001394	1	1.118458	up
Com_3336_pos	Porphobilinogen	C10 H14 N2 O4	226.09541	4.933	227.1027	No results	No results	Full match	40802510.2	48897667.12	39666899.65	41080364.36	40461757.55	33315399.51	17153675.29	12243329.74	17839049.13	20774985.63	18060777.23	16199627.8	2.388004	1.255805	3.90E-06	1	1.263527	up
Com_3525_pos	5-trans prostaglandin F2β	C20 H34 O5	376.2232	6.311	377.2307	Invalid mass	No results	No results	40226298.56	39403805.08	34815013.55	38442549.01	30059360.27	37807768.88	20273861.7	11002320.04	15847260.17	23849516.87	11648991.15	10054610.1	2.381992	1.252168	0.000947	1	1.175847	up
Com_3384_pos	Biliverdin	C33 H34 N4 O6	582.2472	6.36	583.2543	No results	Full match	No results	21858752.75	20733203.49	39537939.9	40273392.22	32708368.75	39521466.85	14197564.94	12738859.67	13289113.42	22775750.51	8819969.958	11364905.6	2.33973	1.226342	0.000746	0.944	1.104823	up
Com_86_neg	Delta-Tridecalactone	C13 H24 O2	212.1774	7.258	211.1702	No results	No results	Full match	1321805727	1279062273	1342101393	1222895735	1293189537	1365731083	853716674.1	251047026.2	651940284.3	930448769.7	375744049.5	292566716	2.331954	1.221539	0.008128	1	1.062477	up
Com_82_neg	Tetradecanedioic acid	C14 H26 O4	258.18276	7.261	257.1753	Full match	No results	Full match	1360898780	1320756263	1383375227	1261440517	1333696485	1407578919	877208089	258631041.1	676027161.2	967282979.5	383874741	303976655	2.32701	1.218478	0.008264	1	1.061017	up
Com_798_neg	Spiculisporic Acid	C17 H28 O6	328.18844	6.132	327.1814	No results	No results	Full match	130312100.9	146683638.9	114601534.7	113068403.8	135631482.6	104268746.2	51956881.66	53465060.18	36911023.43	61587026.72	71610862.12	45109496.5	2.322122	1.215444	5.36E-05	1	1.230287	up
Com_1471_pos	o-Cresol	C7 H8 O	108.05747	5.694	109.0651	No results	No results	Full match	120901822.6	113657587.7	105248743.2	103199344.4	93450887.51	104935810.7	51211520.29	37891100	50754941.94	55675462.36	42259461.12	40240845.9	2.306897	1.205953	3.07E-06	1	1.278074	up
Com_5363_pos	(R)-Equol	C15 H14 O3	242.09414	6.224	243.1016	Full match	Full match	Full match	19272264.99	18976047.51	16876997.35	17189082.64	12121889.99	15962892.97	3562206.111	3658507.791	7392969.433	8410484.871	16384564.47	4398514.03	2.291839	1.196506	0.00885	0.944	1.026899	up
Com_1372_pos	LNH	C16 H26 N6 O5	382.19649	5.967	383.2037	Full match	No results	No results	123369937.9	115718260.4	130415813.1	96514112.17	102127931.5	107942888.1	42183796.08	48510080.76	49593660.18	61106603.84	43860663.03	52860543.7	2.267877	1.181342	5.68E-07	1	1.276777	up
Com_1397_pos	5-[(E)-2-(3,5-dihydroxyphenyl)ethenyl]-2-methoxybenzene-1,3-diol	C15 H14 O5	274.08385	5.519	275.0912	Full match	No results	No results	82559203.1	145664169.8	128522457	110999525	101384411.6	137401182.3	49516040.04	43014097.77	67729450.19	53685412.95	45553862.6	53226275.9	2.259271	1.175858	3.37E-05	1	1.219091	up
Com_7854_neg	2,3-Dinor-8-epi-prostaglandin F2α	C18 H30 O5	652.41847	7.08	651.4112	Invalid mass	No results	No results	6124992.639	5235425.427	3611834.554	4313880.551	4741342.117	4097904.82	2175198.097	2325267.076	1581800.734	3348387.641	1759481.223	1375607.92	2.238258	1.162377	0.000529	1	1.151413	up
Com_1220_pos	4-methoxy-6-(prop-2-en-1-yl)-2H-1,3-benzodioxole	C11 H12 O3	192.07863	5.846	193.0859	Full match	No results	No results	138342323.1	121432533.7	93235790.31	132342017.8	107507849.1	119126385.9	54632211.29	47521394.64	60789947.76	51183629.63	52794222.61	52235981.4	2.230833	1.157583	2.69E-06	1	1.279573	up
Com_1153_neg	Homovanillic acid	C9 H10 O4	182.05773	5.235	181.0504	Full match	Full match	Full match	60041253.53	77900352.39	88036439.64	73095549.2	47205022.27	51034132.35	23428358.75	33798339.48	30686675	29780621.1	39624000.61	20784870.3	2.230805	1.157564	0.000194	1	1.159407	up
Com_973_neg	Resveratrol	C14 H12 O3	228.07842	5.906	227.0711	Full match	Full match	No results	82751454.44	86364391.65	87962657.19	93159557.34	75590578.65	78457895.59	52636612.02	32381875.61	36813808.01	37760675.45	36357038.71	31351183.1	2.218583	1.149638	3.02E-05	1	1.262162	up
Com_588_neg	Suberic acid	C8 H14 O4	174.08886	5.767	173.0815	Full match	Full match	Full match	188565396.2	172377546.4	79015458.4	82808959.42	161387337.9	159950502.5	63882088.91	39576742.01	72192468.74	60977388.87	95421738.89	49615769.2	2.211632	1.145111	0.003599	0.944	1.024228	up
Com_6359_neg	Oxaceprol	C7 H11 N O4	173.06859	1.426	172.0613	No results	Full match	No results	5836493.364	5432841.564	7175337.503	6869689.567	8563906.326	7021861.751	2265501.878	2380819.687	2105478.577	2489207.718	6944929.856	2396738.67	2.200982	1.138147	0.003621	0.917	1.085151	up
Com_5706_neg	N-Acetyl-Asp-Glu	C11 H16 N2 O8	304.0906	1.375	303.0834	No results	No results	Full match	7144532.829	7238213.903	8663003.907	9170254.146	10384751.91	8363419.803	2523488.072	4195126.737	4004016.604	5749393.432	3382799.863	3852947.93	2.149682	1.104123	0.000287	1	1.184205	up
Com_3904_neg	19(R)-hydroxy Prostaglandin A2	C20 H30 O5	332.19858	7.096	331.1914	Invalid mass	No results	No results	12244238.93	13402416.31	15021196.78	16111443.5	19891801.88	17392593.42	6090494.391	7170806.492	7981207.45	8565190.89	7160909.441	6803758.93	2.148929	1.103618	1.26E-05	1	1.245404	up
Com_7313_neg	Glycylproline	C7 H12 N2 O3	172.08454	1.363	171.0775	Full match	No results	Full match	3337520.033	5585737.841	5646384.737	5305241.674	4601233.818	6308399.25	1910439.152	2027071.651	1291700.784	1551713.008	5446011.644	2176247.41	2.137341	1.095817	0.006333	0.917	1.024141	up
Com_14835_pos	Tomatidine	C27 H45 N O2	393.36015	9.383	416.3494	Invalid mass	No results	No results	1179227.513	2357063.818	2952838.479	1750235.565	1789854.323	2059458.329	1010746.193	963038.361	1018021.843	755958.9608	752352.0293	1201559.24	2.120197	1.084198	0.001103	0.972	1.115854	up
Com_476_neg	1-(2,4-dihydroxyphenyl)-2-(3,5-dimethoxyphenyl)propan-1-one	C17 H18 O5	302.11498	5.673	301.1077	Full match	No results	No results	217479658.1	207121750.7	231007611.6	198163820	226545735.6	223018294.9	132239917.1	120307292.8	102945631.6	99451606.36	78537531.7	82703672.7	2.115169	1.080773	0.000138	1	1.247693	up
Com_15208_pos	EOH	C16 H24 N6 O7	394.16054	5.625	395.1678	Invalid mass	No results	No results	2442539.931	3153532.731	2530255.195	2227808.712	2598661.044	2626075.864	1437632.334	1025341.294	1249100.725	1413590.763	1075743.238	1208623.56	2.102403	1.072039	1.76E-06	1	1.265301	up
Com_3786_pos	Dehydrocholic acid	C24 H34 O5	402.23997	6.272	403.247	Full match	Full match	Full match	38861331.05	39649069.43	33976331.88	34727682.68	29914026.62	32693719.42	17744620.21	17430256.39	12266874.44	14774854.78	23939958.47	14175582.5	2.091276	1.064383	0.000168	1	1.214301	up
Com_10230_neg	5-Phenylvaleric Acid	C11 H14 O2	178.09918	6.034	177.0919	No results	No results	Full match	2840966.584	2989324.537	3390775.78	2538551.782	2545527.936	3178826.001	1260805.345	1285341.742	1203094.945	1755869.456	1430193.853	1458979.51	2.082842	1.058554	1.95E-06	1	1.263183	up
Com_282_pos	(+/-)12(13)-DiHOME	C18 H34 O4	296.23486	8.414	297.2422	Invalid mass	No results	No match	467003655.8	679636630.2	592259725.4	565285810	660457097.6	763777297.1	382827996.6	231787738.7	322728180.9	240643984.9	332497456.2	310225259	2.047783	1.034063	5.24E-05	1	1.200964	up
Com_2205_neg	3,8,9-trihydroxy-10-propyl-3,4,5,8,9,10-hexahydro-2H-oxecin-2-one	C12 H20 O5	244.13072	6.171	243.1236	Full match	No results	No results	35482839.15	37747172.15	41110397.05	31255437.11	36182284.6	34572295.52	20077943.7	14794719.45	18374729.92	20723640.95	15602657.62	16170420.1	2.045981	1.032793	3.15E-06	1	1.269384	up
Com_2177_pos	p-Mentha-1,3,8-triene	C10 H14	134.10949	5.68	135.1168	No results	No results	Full match	78036149.6	63012405.84	63123734.55	49715601.56	67354422.1	59084276.24	37079771.11	23846093.65	23457499.14	51292941.98	30569315.99	22617592.8	2.013767	1.009897	0.001451	0.972	1.126702	up
Com_5409_neg	L-Phenylalanine	C9 H11 N O2	165.07869	5.123	164.0714	Full match	Full match	Full match	11763610.86	10351438.7	10889638.38	7691853.084	10340426.99	10637770.18	4589987.044	4728654.279	4484752.908	5180978.502	6486575.327	5213296.62	2.009981	1.007182	6.55E-06	1	1.243109	up
Com_4412_neg	trans-Cinnamic acid	C9 H8 O2	148.05216	5.54	147.0447	Full match	Full match	Full match	16260469.78	17293370.72	15455844.75	13032437.29	12520505.08	12744571.06	9658969.108	6130653.731	8302066.876	7070366.518	6612937.518	5817559.96	2.002801	1.002019	4.57E-05	1	1.213712	up
Com_2654_pos	15-Deoxy-Δ12,14-prostaglandin A1	C20 H30 O3	300.2087	8.257	301.216	Invalid mass	No match	No match	12031982.3	15050784.02	19500766.76	20234037.34	11300102.56	12165649.41	33022459.6	25674578.96	31859006.13	40723466.95	26288102.92	25314322.9	0.49367	-1.01838	0.000336	1	1.151179	down
Com_1944_pos	(1E,4E)-1,5-bis(4-methoxyphenyl)penta-1,4-dien-3-one	C19 H18 O3	316.10563	5.494	317.1129	Invalid mass	No results	No results	26918872.27	25273821.77	20547940.04	21629560.32	22479167.62	27021163.89	50187699.54	35895620.13	63080043.27	39948669.14	43654472.56	62659795	0.486993	-1.03803	0.000254	1	1.193315	down
Com_1082_pos	3-hydroxy-2-octylpentanedioic acid	C13 H24 O5	242.15178	6.095	243.1589	Invalid mass	No results	No results	54252724.97	58500918.29	35808688.08	37253707.48	42119147.65	42385975.5	109188740.9	79352034.03	125585469.1	67553652.82	78099128.78	97059457.6	0.485457	-1.04258	0.00021	1	1.159508	down
Com_3369_pos	Homo-Gamma-Linolenic Acid (C20:3)	C20 H34 O2	306.25537	7.973	307.2627	No results	No results	Full match	10629926.45	12163330.26	12323476.33	15027107.3	8363323.639	8764859.872	20098050.19	26406794.69	18785779.33	24355722.34	25185020.19	25028467.7	0.480996	-1.0559	9.50E-05	1	1.205585	down
Com_5835_neg	3-Hydroxydecanoic acid	C10 H20 O3	188.14096	6.988	187.1336	Full match	No results	Full match	2670661.749	2205128.944	2312968.315	1966809.247	2591803.638	3030530.559	2757937.129	6380044.921	4127932.947	6592000.556	6209566.673	5066077.1	0.474662	-1.07503	0.002258	0.972	1.098814	down
Com_2418_pos	trans-Cinnamaldehyde	C9 H8 O	132.05747	6.02	133.0647	Full match	No results	No results	11369776.9	20142934.97	9643002.37	9040029.075	18879632.7	17250044.3	20877450.85	24692298.93	27885591.39	45345058.43	40172462.07	23012215.1	0.474354	-1.07596	0.003102	1	1.031479	down
Com_6129_pos	Cafestol	C20 H28 O3	316.20262	6.775	317.2099	Full match	No results	Full match	5805465.359	5372647.653	4356783.078	4723052.161	5265976.585	5197596.521	10621321.25	7366241.385	11424337.13	10297221.94	13254958.97	11964101	0.473162	-1.0796	6.88E-05	1	1.232372	down
Com_4262_pos	2-{[(4,5-dimethoxy-2-nitrophenethyl)imino]methyl}phenol	C17 H18 N2 O5	330.12118	5.753	331.1285	Full match	No results	No results	6958941.254	7556769.693	7614837.507	6059972.454	7930708.052	7221727.436	11109265.88	12899660.41	17181535.32	22050730.78	14710483.55	14479410.5	0.468922	-1.09258	0.000267	1	1.210655	down
Com_1843_pos	5-(6-hydroxy-6-methyloctyl)-2,5-dihydrofuran-2-one	C13 H22 O3	208.14615	6.117	209.1535	Invalid mass	No results	No results	20873749.7	19548083.74	16479483.6	18574148.92	17863749.79	17939784.1	37872154.68	25527605.52	40178908.8	34829276.72	37409583.3	65874234.9	0.460417	-1.11899	0.001512	1	1.158077	down
Com_8507_neg	DL-o-Tyrosine	C9 H11 N O3	181.07377	6.273	180.0666	No results	No results	Full match	1487261.036	1347405.584	1589592.009	1485666.903	1375322.788	1390274.744	3975844.473	2578267.389	3540274.215	2158182.834	4089183.781	3404551.98	0.439349	-1.18656	0.000408	1	1.220083	down
Com_3225_pos	Flunitrazepam-d3	C16 H9 [2]H3 F N3 O3	316.10563	6.675	317.1129	Full match	No results	No results	10109330.38	11858223.06	10552278.84	12426562.67	10663234.61	9708368.507	17327587.45	20832083.21	25200895.09	32122240.56	31979966.63	23733241.2	0.432009	-1.21087	0.000166	1	1.22694	down
Com_3368_pos	1,4-dihydroxyheptadec-16-en-2-yl acetate	C19 H36 O4	310.24849	9.575	311.2558	Invalid mass	No results	No results	10773515.47	12275694.51	9850316.882	5668296.684	10864622.37	10107977.61	17505229.61	22449866.79	23454122.46	27938467.65	31233741.22	16937830.4	0.426754	-1.22852	0.000202	1	1.160787	down
Com_1933_pos	Testosterone	C19 H28 O2	288.20859	7.669	289.2158	Full match	No results	Full match	16537740.79	13938103.05	14524681.94	14415245.91	10273078.97	14439132.45	35510069.93	51643066.15	17039657.96	28193836.75	30823698.26	36204769.5	0.421874	-1.24512	0.001613	1	1.122106	down
Com_5685_pos	2,3-dihydroxypropyl 12-methyltridecanoate	C17 H34 O4	284.23479	8.61	285.242	Invalid mass	No results	No results	5609376.637	5295468.177	4007464.743	3754985.733	5868364.792	4566320.982	13757031.42	10287413.22	13656079.18	3901040.479	13507612.45	15476820.2	0.412291	-1.27826	0.010149	0.861	1.001938	down
Com_475_neg	7-Ketodeoxycholic acid	C24 H38 O5	406.27161	6.885	405.2644	No results	No results	Full match	29694849.8	27906190.74	39519211.61	49101505.21	31261357.28	45946737.43	77496043.26	66197545.41	64733166.75	152450998.1	127599601.5	65786627.2	0.403111	-1.31075	0.001229	1	1.105551	down
Com_4196_pos	beta-Estradiol 17-Acetate	C20 H26 O3	314.1876	7.88	315.1949	No results	No results	Full match	4557739.686	4216003.797	4357641.403	4438466.49	5122917.102	6147612.397	8257272.7	7531613.382	13515986.42	13261268.5	23068325.81	7914531.15	0.392125	-1.35062	0.003713	1	1.091286	down
Com_13051_pos	Cortisol	C21 H30 O5	724.4135	7.269	725.4208	Invalid mass	No results	No results	644792.1777	783254.2643	717453.0286	1064546.928	624508.7917	857911.1092	2598177.026	2497857.647	1955053.078	641880.111	2551627.584	2357920.97	0.372344	-1.42529	0.007696	0.861	1.028087	down
Com_7726_neg	4-Pyridoxic acid	C8 H9 N O4	183.05296	2.228	182.0457	Full match	Full match	Full match	897375.4337	1111184.455	1236961.766	831315.2673	1249640.044	701349.9354	1452712.285	3178140.572	2659406.07	4051642.74	2735984.287	2203340.59	0.370232	-1.4335	0.000346	1	1.156459	down
Com_708_pos	Oleanolic acid	C30 H48 O3	478.34198	9.035	479.3492	Invalid mass	No results	No results	52751266.63	60030169.44	21605379.92	38613772.57	70485440.26	60347493.54	148410400.5	127063846.9	196429840.7	122960112.5	87544207.39	139886888	0.369494	-1.43638	0.000974	1	1.118586	down
Com_3673_pos	Linolelaidic Acid (C18:2N6T)	C18 H32 O2	280.24158	9.516	281.2482	No results	No results	Full match	8966712.887	6989578.093	8611618.137	7208887.701	5145203.092	6228908.852	12483853.97	23218129.49	20424380.48	27296620.31	17101779.6	22264590.8	0.351422	-1.50872	3.60E-05	1	1.217696	down
Com_206_neg	8Z,11Z,14Z-Eicosatrienoic acid	C20 H34 O2	306.25541	10.247	305.2481	Full match	No results	Full match	106018847.1	108813098.3	85846568.94	70099043.62	84787771.05	70689077.46	284412669.2	253436569.3	314409086.3	363200085.5	86703734.77	232158900	0.342988	-1.54377	0.003668	0.944	1.082667	down
Com_3446_neg	Isorhamnetin	C16 H12 O7	316.05814	6.119	315.0509	No results	Full match	No results	3972192.711	6731878.895	5874671.251	4224639.648	3476250.355	4803497.143	13781854.09	19282641.1	16005037.66	9259286.223	7953340.194	18793533.1	0.34185	-1.54856	0.000314	1	1.160537	down
Com_2559_neg	Palmitic Acid	C16 H32 O2	302.24518	7.994	301.2379	Invalid mass	No results	No results	4318100.907	4542279.041	4283652.279	7429639.519	6660817.629	3660317.14	22098225.78	12352964.22	16910816.72	22501417.47	6978661.868	9830954.11	0.340728	-1.55331	0.001648	0.972	1.092429	down
Com_7455_neg	trans-2-Butene-1,4-dicarboxylic Acid	C6 H8 O4	144.04204	1.312	143.0347	No results	No results	Full match	941523.1772	1013341.473	1417296.605	831129.9075	1078918.495	739217.5537	1192229.827	5478440.672	3600163.639	2674407.138	3368077.288	1522168.58	0.337609	-1.56657	0.006593	0.972	1.031193	down
Com_9331_pos	6-(Methylthio)purine	C6 H6 N4 S	166.03071	5.14	167.0379	Full match	No results	No results	1130735.572	877855.9679	1767258.774	888831.5129	1190909.307	975897.3336	6355719.726	2967330.83	3127253.412	3072841.991	1229030.336	3531226.99	0.336802	-1.57003	0.003579	0.972	1.059533	down

Com_297_neg	11(Z),14(Z)-Eicosadienoic Acid	C20 H36 O2	308.27114	10.769	307.2637	Full match	No results	No results	65891722.11	90419631.88	62047320.52	26782817.13	48798733.15	34169331.2	122688364.5	141972587.5	170434425.6	256127088.3	163530077.2	133427219	0.332034	-1.5906	0.000612	1	1.147137	down
Com_9910_neg	L-Cysteic acid	C3 H7 N O5 S	169.00421	1.564	167.9969	Full match	Full match	No results	249251.367	503542.59	607133.2499	570183.249	853068.6385	682980.0704	1537526.48	2441536.406	1409137.792	1079358.001	894754.7635	3088867.83	0.331652	-1.59226	0.002043	1	1.05537	down
Com_486_pos	(11E,15Z)-9,10,13-trihydroxyoctadeca-11,15-dienoic acid	C18 H32 O5	350.20681	7.143	351.214	Invalid mass	No results	No results	57663347.63	63397526.52	52078542.23	54850923.63	49265992.66	52023114.62	120526297.9	103960401.5	290702494.3	241281799.2	130933691.1	123666828	0.325674	-1.6185	0.001444	1	1.170167	down
Com_1457_neg	3-Methyl-2-oxobutanoic acid	C5 H8 O3	116.04717	4.896	115.0399	No results	No results	Full match	9520124.815	8384996.552	6649699.171	8367726.499	9013083.112	9398870.285	14653422.72	19152268.36	19529038.37	29592575.55	54210421.85	24552813.5	0.317486	-1.65523	0.001733	1	1.14714	down
Com_2481_neg	(±)8(9)-DiHET	C20 H34 O4	338.2455	10.506	337.2382	Full match	No results	No results	9200956.74	4594900.67	5158202.923	5167213.24	4769593.12	2033693.653	20080548.75	13059032.52	12274281.99	23491138.16	9397407.745	20725122.6	0.312282	-1.67908	0.000819	1	1.111515	down
Com_9754_pos	Oxymorphone	C17 H19 N O4	301.13112	4.998	302.1384	Full match	No results	No results	1461351.919	1412338.671	1321331.582	1089493.002	983176.0578	1124533.084	5785965.015	4398621.775	4290471.478	2610106.764	1368483.53	5477266.74	0.308899	-1.6948	0.003887	0.944	1.093462	down
Com_2494_neg	(+/-)-Equol	C15 H14 O3	242.09433	5.86	241.0873	No results	No results	Full match	4880718.848	8027433.719	6701423.924	6835300.427	6415133.814	6651557.284	19133023.86	20878671.35	23143044.46	23336953.72	20633039.72	21068066.7	0.30822	-1.69797	7.34E-07	1	1.300421	down
Com_9808_pos	5,7-dihydroxy-2-(2,3,4-trihydroxyphenyl)-4H-chromen-4-one	C15 H10 O7	302.04225	5.576	303.0495	Full match	No results	No results	999719.296	990932.2495	1156606.478	1025973.163	1077175.348	1002118.141	3399195.424	3795620.563	2588823.174	5561025.572	2381810.426	3025811.96	0.301293	-1.73076	0.000176	1	1.252647	down
Com_2382_pos	6-Hydroxyflavone	C15 H10 O3	238.06285	5.818	239.0702	Full match	No results	No results	9765703.996	11262886.05	9906217.434	9348151.616	11369125.13	12131088.16	33747690.02	29370912.31	36070242.84	46277871.05	34486623.22	32135086.5	0.300739	-1.73342	9.75E-08	1	1.299546	down
Com_751_pos	13-OxoODE	C18 H30 O3	316.20122	7.821	317.2085	Invalid mass	No results	No results	31321866.25	28982125.56	27110809.22	33382459.53	40368827.93	34297151	76876367.28	98867205.11	160535895	166072312.3	76035329.65	73825174	0.299693	-1.73844	0.000333	1	1.207524	down
Com_169_neg	Ethyl chrysanthemumate	C12 H20 O2	196.14614	5.823	241.1444	No results	No results	Full match	140976954.1	209890456.4	110542181.1	47322457.37	41181799.65	127791457.8	329845385.8	480080538.7	261794560.2	449372899.9	325706948.4	448445898	0.295265	-1.75992	0.002511	1	1.108335	down
Com_2528_pos	Ergosterol peroxide	C28 H44 O3	428.32903	10.719	429.3366	Full match	No results	No results	10745258.81	7255812.438	7561803.707	5014460.729	12927582.97	4545199.212	27627313.68	20452122.79	23701169.02	12273198.04	44424627.62	35366413.7	0.293266	-1.76972	0.000636	0.972	1.113349	down
Com_949_neg	1a,1b-Dihomo prostaglandin E1	C22 H38 O5	364.26101	8.025	363.2537	Invalid mass	No results	No results	7756036.246	6949136.943	21848683.43	29174926.71	11697824.26	11469741.13	37869287.53	52481648.39	62760329.81	71693790.14	43561082.49	35286578.4	0.292757	-1.77223	0.001139	1	1.129114	down
Com_7134_neg	L-(-)-Arabitol	C5 H12 O5	152.0682	1.454	151.0609	Full match	Full match	Full match	598152.7587	494774.6368	565052.3904	598056.6697	1611763.743	910571.7239	1228083.713	2550733.995	3029503.177	1931735.497	5678180.724	2080206.7	0.289626	-1.78774	0.001395	0.972	1.076203	down
Com_1132_pos	Maslinic acid	C30 H48 O4	494.33681	9.045	495.3441	Invalid mass	No results	No results	16829327.3	14794731.55	14152235.9	15015427.26	25948916.36	21382964.52	64549309.32	53072032.27	77217463.31	106449416	22942307.68	51398957.8	0.287846	-1.79663	0.001476	0.972	1.121499	down
Com_2281_pos	2,4-dihydroxyheptadec-16-en-1-yl acetate	C19 H36 O4	350.24537	7.493	351.2528	Invalid mass	No results	No match	9253275.19	9808288.996	5939303.265	8763388.34	7944328.879	8162295.228	27958400.6	42517413.81	22059352.34	15383156.27	36819370.65	30101472.6	0.285239	-1.80976	0.000138	1	1.216529	down
Com_2784_pos	Etiocholanolone	C19 H30 O2	272.21367	8.899	273.2209	Invalid mass	No results	No results	5107614.425	5652741.445	5292863.161	5718066.825	6160751.616	5261304.468	14365967.9	13599469.65	24873245.1	38507147.31	9319350.001	16638728.5	0.282969	-1.82129	0.002202	1	1.155713	down
Com_1414_pos	Nootkatone	C15 H22 O	218.16692	8.835	219.1742	Full match	No results	No results	13627743.24	12824990.32	8784717.247	10052942.13	22037163.39	21481727.77	49240910.37	51562349.28	52018136.11	84743900.08	26810610.32	54231418.1	0.278742	-1.843	0.000143	1	1.170301	down
Com_605_pos	Linoleoyl ethanolamide	C20 H37 N O2	323.28207	9.254	324.2893	Full match	No results	No results	30660451.5	51467600.63	29225602.19	13657373.94	78558996.02	30679124.08	102147430.9	180682288.2	158851269.4	109668015.9	156224829.7	135221006	0.277943	-1.84714	0.001313	1	1.144406	down
Com_1816_pos	tetranor-12(R)-HETE	C16 H26 O3	248.17744	7.28	249.1847	Invalid mass	No results	No results	10722175.46	11921285.97	12726411.35	14090996.67	12815850.95	12145559.66	46148051.59	49353301.85	49407807.63	26271508	64970081.34	35214362.1	0.274251	-1.86643	9.76E-05	1	1.25597	down
Com_1716_neg	12,13-EODE	C18 H32 O3	296.23451	7.937	295.2271	No results	No results	Full match	5358291.651	3986764.792	3417472.041	4312013.645	5358747.024	3895976.799	6948920.802	15404467.17	17426862.73	37023287.08	9357282.469	10809491.6	0.271519	-1.88088	0.003652	1	1.096017	down
Com_5263_neg	Hydroxykynurenine	C10 H12 N2 O4	224.08331	5.723	223.0759	Invalid mass	No results	No results	4142173.505	1500623.995	1241895.053	1533571.22	989262.2676	1023801.793	7693960.596	5513901.188	7513476.098	7832556.692	4238946.312	6935308.53	0.262568	-1.92924	0.000461	1	1.177946	down
Com_1446_neg	5-hex-1-ynyl-2-furoic acid	C11 H12 O3	192.07841	5.671	191.0711	Full match	No results	No results	7774091.01	11070621.75	12647258.62	10166686.37	5274854.55	9480049.221	27184192.12	56630451.12	33896361.59	19257120.49	37852387.93	41050477	0.26133	-1.93606	5.97E-05	1	1.199446	down
Com_4042_pos	Trifolin	C21 H20 O11	448.09979	5.694	449.107	Full match	Full match	No results	2864033.686	4623729.195	4095617.682	6775767.839	3940136.497	3580033.602	9889655.281	18619034.7	26633172.28	9338663.897	11323553.37	23582663.8	0.26039	-1.94125	0.000321	1	1.166341	down
Com_10667_pos	3-[4-(4-methoxyphenyl)-1,3-thiazol-2-yl]-5-methylisoxazole	C14 H12 N2 O2 S	272.05731	5.314	273.0645	Invalid mass	No results	No results	957078.9688	981859.0224	894455.7234	834063.1169	806560.6573	880233.5025	3101138.352	3492921.379	3858113.68	2597007.55	4785127.654	3167826.68	0.254938	-1.97178	3.85E-06	1	1.295608	down
Com_4764_pos	9,10-Dihome	C18 H34 O4	314.24527	6.537	315.2523	No results	No results	Full match	5533346.871	3101121.776	3881831.19	2765733.084	3394822.575	2907684.53	13011112.56	10439017.11	17305372.19	18826873.25	14601197.2	10860332.1	0.253805	-1.97821	2.35E-06	1	1.259212	down
Com_1519_pos	Glycerol 1-hexadecanoate	C19 H38 O4	330.27669	8.937	331.2837	No results	No results	Full match	18710300.98	14844075.85	10053392.16	16707923.81	13698875.18	14626573.65	31912456.32	60513795.45	87828041.51	61729013.77	49822809.35	57985646.1	0.253411	-1.98045	2.16E-05	1	1.239662	down
Com_522_neg	Butylparaben	C11 H14 O3	194.09398	6.096	193.0866	Full match	No results	No results	35807163.3	35852435.53	14501248.92	12288721.73	26718039.96	24855948.81	56022005.84	60244884.49	134719361.2	138185982.4	142688109.7	60802506.6	0.253135	-1.98202	0.000449	1	1.129839	down
Com_990_pos	α-Linolenoyl ethanolamide	C20 H35 N O2	321.26639	8.795	322.2737	Full match	No results	No results	22011219.56	66081486.28	27305372.64	7839656.348	17159279.33	11508368.05	78271983.95	107518141.8	126905155.6	89401502.96	115429875.1	84461786.3	0.252339	-1.98656	0.002537	1	1.128668	down
Com_7669_pos	Biochanin A	C16 H12 O5	284.0683	6.45	285.0754	Full match	No results	No results	1155648.435	1160695.776	1410761.963	991633.8188	1307596.518	1402349.581	9147167.918	2768155.933	3727626.67	4728041.471	6179518.7	2922610.59	0.25205	-1.98822	0.000644	1	1.194837	down
Com_1344_neg	2-(1-benzothiophen-3-ylmethylene)-3,4-dihydronaphthalen-1(2H)-one	C19 H14 O S	145.03817	1.378	289.0692	Invalid mass	No results	No results	9306527.484	5384388.458	7873142.965	12690755.26	13590175	5841666.802	18890394.58	23134302.09	56094346.08	49283796.16	36236251.4	35551678.4	0.249493	-2.00293	0.000153	1	1.168192	down
Com_2058_pos	6-Acetylmorphine	C19 H21 N O4	654.29361	5.03	328.1541	Invalid mass	No results	No results	9066890.827	3655530.131	5620351.38	4351331.745	4535309.022	5978256.224	56418686.4	10619378.84	22262278.42	22501494.14	12933708.6	11590213.2	0.243591	-2.03747	0.002667	1	1.078518	down
Com_78_neg	Hesperetin	C16 H14 O6	302.07864	5.874	301.0712	Full match	No results	No results	200515141.7	180682047.2	179965076.7	189885324.3	213653950.9	203212772.5	762108691.8	845975762.5	839075167.4	1021172278	677429445.3	756705348	0.23823	-2.06957	5.15E-08	1	1.311953	down
Com_12810_pos	Citrinin	C13 H14 O5	272.06645	5.375	273.0737	Invalid mass	No results	No results	371640.7416	1411377.272	325779.2769	300823.7302	306030.2677	420559.4324	1019265.714	2624513.147	3159735.534	2430240.025	973890.6767	2971942.5	0.23796	-2.07121	0.00094	0.944	1.095101	down
Com_900_pos	11β-Hydroxyandrosterone	C19 H30 O3	306.21914	7.66	307.2264	Full match	No results	No results	19515174.51	17662768.04	16737628.15	16954473.33	15731379.22	17095847.51	73050189.91	117837765.5	42148030.75	60995339.35	59618583.12	83515142.1	0.237204	-2.0758	0.000133	1	1.257928	down
Com_9304_neg	5(S),15(S)-DiHETE	C20 H32 O4	318.21882	7.145	317.2116	Invalid mass	No results	No results	456787.7776	585346.3943	500158.2034	704120.0971	451928.6584	422618.8284	3515723.052	1048859.672	3612042.08	1968876.201	860991.6961	2151417.09	0.237193	-2.07587	0.002118	1	1.127992	down
Com_4050_neg	Hydroquinone	C6 H6 O2	110.03654	7.543	109.0293	No results	No results	Full match	2989020.963	2090622.931	2427797.387	2528471.09	1831853.337	2045685.874	4603170.505	10389048.28	7017000.979	11855740.23	10118948.03	15434439.8	0.234161	-2.09443	0.000189	1	1.218573	down
Com_6949_pos	4-(methylthio)-6-phenyl-2-(3-pyridyl)pyrimidine-5-carbonitrile	C17 H12 N4 S	304.07634	1.321	305.0837	Invalid mass	No results	No results	1272075.734	1902430.687	1050222.983	1485705.312	1307805.206	2230937.405	5868705.979	9128725.737	5219222.255	7363617.089	3654191.947	8439098.2	0.233132	-2.10078	1.47E-05	1	1.232345	down
Com_1756_pos	Anandamide (AEA)	C22 H37 N O2	353.29261	10.299	354.2999	Invalid mass	No results	No results	4333498.087	4263190.253	15429121.94	10961872.79	8346858.311	6769626.91	13981190.58	47106131.39	31094965.08	65479046.52	10474220.64	48014258.8	0.231803	-2.10903	0.004646	0.917	1.012665	down
Com_95_neg	Arachidic acid	C20 H40 O2	312.30239	11.348	311.2951	Full match	No results	Full match	128101181.8	193808478	220513706	113713282.7	339351854.7	139451536.8	1014846015	767111324.1	603774126	846825042.5	844290639.8	909243225	0.227621	-2.13529	7.43E-05	1	1.241087	down
Com_15156_pos	Thymidine 3,5-cyclic monophosphate	C10 H13 N2 O7 P	304.04543	5.307	305.0528	No results	No results	Full match	400175.4281	410536.5047	373991.294	348739.8383	337240.4648	368044.6757	1277257.462	1596266.46	2002639.424	1644099.947	1746877.247	1611010.07	0.226634	-2.14156	4.35E-08	1	1.311161	down
Com_1649_neg	Thromboxane B3	C20 H32 O6	322.21397	7.449	321.2068	Invalid mass	No results	No results	5007058.692	4936754.59	4486216.637	4454700.416	6764374.706	5361892.14	23010627.75	16198779.19	17356475.73	16233463.12	47241924.24	17012637.1	0.226269	-2.14389	0.000191	1	1.22604	down
Com_510_neg	(±)9-HpODE	C18 H32 O4	312.22963	7.531	311.2223	Full match	No results	Full match	31912194.54	26537299.94	28279470.26	25221419.03	41791078.25	34895948.24	115048302.7	134844670.9	146374545.1	142286820.7	151983537.3	146494523	0.225365	-2.14967	2.32E-07	1	1.302742	down
Com_3526_neg	(2S)-4-Oxo-2-phenyl-3,4-dihydro-2H-chromen-7-yl beta-D-glucopyranoside	C21 H22 O8	402.1341	6.684	401.1268	Invalid mass	No results	No results	3059846.667	2464162.116	1604394.765	1360269.77	2110084.308	2254429.56	6949784.22	6185218.142	10699461.75	14701931.91	11467445.65	7307048.56	0.224271	-2.15668	1.25E-05	1	1.235281	down
Com_367_neg	9-HpOTrE	C18 H30 O4	310.21405	7.318	309.2069	No results	No results	Full match	29859130.8	25572642.14	20151355.5	25912885.73	29160890	24273374.64	60184952.74	62627724.58	168679464.7	202768295.7	137535901.1	73701286	0.219604	-2.18702	0.000967	1	1.182751	down
Com_996_neg	8,15-Dihete	C20 H32 O4	336.2299	7.788	335.2226	No results	No results	Full match	9862077.342	9387743.939	9763997.815	10041975.11	11294376.37	10702432.27	42323636.43	30538318.6	43138282.38	58053088.15	83500290.43	25555889.5	0.21565	-2.21324	0.00034	1	1.237522	down
Com_2789_pos	5beta-Androstane-3,17-dione	C19 H28 O2	288.20938	9.731	599.4092	No results	No results	Full match	1915432.493	3877589.114	3810864.853	1546540.827	3845785.437	2499823.849	16101537.03	33409752.1	5768972.095	10010871.07	4590469.303	11300225.9	0.215517	-2.21413	0.00357	1	1.049001	down
Com_5956_neg	acetoacetate	C4 H6 O3	102.03152	1.335	101.0243	No results	No results	Full match	991596.1579	728800.1162	896250.6636	753977.4419	732766.3006	934296.5139	1814849.263	8088714.548	3024396.247	2966343.21	4422486.223	3092649.67	0.215199	-2.21626	0.000614	1	1.200103	down
Com_5019_neg	15-Deoxy-Δ12,14-prostaglandin D2	C20 H30 O4	316.20381	7.605	315.1965	Invalid mass	No results	No match	1589010.927	1123894.159	1624417.421	1551073.908	1454206.088	1004853.424	7890797.797	3711884.157	5670254.173	5840055.064	10354282.05	5934364.65	0.211856	-2.23885	1.39E-05	1	1.254139	down
Com_6470_pos	5,7-dihydroxy-2-phenyl-4H-chromen-4-one	C15 H10 O4	254.05763	6.836	255.0649	Full match	No results	No results	529975.1922	826709.8343	645754.7615	852520.3678	1083765.14	1717653.769	12165640.38	2767581.28	3544923.286	3700900.826	1665220.948	3159072.27	0.20947	-2.25519	0.001776	0.972	1.083691	down
Com_8933_neg	Soyasaponin I	C48 H78 O18	942.51895	7.781	941.5123	Full match	No results	No results	362113.2781	525189.4527	490851.2119	507451.7767	588948.9169	654678.3955	1454950.255	3114201.635	3826650.022	1632730.444	1252273.32	3852925.26	0.206772	-2.27389	0.00041	1	1.195685	down
Com_4868_pos	6-(3-hydroxybutan-2-yl)-5-(hydroxymethyl)-4-methoxy-2H-pyran-2-one	C11 H16 O5	250.08396	6.312	251.0914	Invalid mass	No results	No results	2379965.757	2943246.601	2553162.475	2584052.93	2284058.833	2769374.406	6677743.664	12381090.53	14849594.72	17038998.58	4640845.68	19549595	0.206472	-2.27598	0.001303	1	1.180708	down
Com_1842_pos	(9cis)-Retinal	C20 H28 O	284.21362	7.224	285.2208	Full match	No results	Full match	5004002.077	3779306.456	6400743.399	5376151.496	4639750.333	4852124.502	9400852.839	19697218.77	31554216.61	61979670.03	7513738.535	17639393.9	0.20335	-2.29796	0.007257	1	1.055126	down
Com_4472_neg	(+/-)9(10)-EpOME	C18 H32 O3	342.23995	7.889	341.2332	Invalid mass	No results	No results	2327248.414	2353798.981	1391495.184	1307517.902	1386015.011	1930858.928	13118613.66	13025385.69	9109014.747	6534414.932	4069941.926	8835902.92	0.19558	-2.35417	5.99E-05	1	1.219654	down
Com_11_pos	Oleoyl ethylamide	C20 H39 N O	309.30274	10.566	310.31	Full match	No results	No results	1039517582	222105641.3	745075009.4	282649734.5	1339094252	242410726.5	4697313388	6165832227	1446650262	890550260.6	2058401316	4712738868	0.193819	-2.36722	0.004172	0.944	1.007731	down
Com_5172_pos	17alpha-Ethinyl estradiol	C20 H24 O2	296.17706	7.427	297.1844	No results	No results	Full match	1980684.553	2391565.2	2348839.031	2575669.693	1667113.251	2404397.713	11934824.58	9011476.916	12267274.64	16790133.63	10944247.11	8497260.86	0.192501	-2.37706	3.74E-07	1	1.290894	down
Com_8655_pos	Riboflavin-5-phosphate	C17 H21 N4 O9 P	456.10493	6.053	457.112	No results	No results	Full match	938233.6912	854559.0687	764169.6055	675650.9703	698914.1108	828701.2875	1842328.85	6173796.479	3985685.852	7023025.825	995680.5092	5129808.28	0.189271	-2.40147	0.005038	1	1.093377	down
Com_9607_pos	dimethyl 2-(3-nitro-2-pyridyl)malonate	C10 H10 N2 O6	216.1009	5.315	255.0641	Invalid mass	No results	No results	489685.9618	1001569.815	661004.3736	974417.5442	584892.7391	434452.9422	4375362.427	923746.8345	1558470.372	4467436.849	4504643.363	6158180.66	0.18856	-2.40691	0.002596	0.944	1.089321	down
Com_13323_pos	P-Coumaroyl Agmatine	C14 H20 N4 O2	276.15848	4.961	277.1656	No results	No results	Full match	296080.0684	278131.3827	197994.0595	186682.4542	236655.8507	229371.952	1428279.929	949030.0812	721788.1842	905197.2828	2802492.393	840070.737	0.18634	-2.42399	0.00028	1	1.214554	down
Com_102_pos	MAG (18:3)	C21 H36 O4	352.26093	9.075	353.2681	No results	No results	Full match	66487752.58	167502076.7	66850462.25	40580532.83	86680119.71	96184589.43	567594554.5	858013463.8	406989138.4	97412314.15	318133988.9	627135691	0.182342	-2.45528	0.002207	0.972	1.074181	down
Com_1517_neg	20-Hydroxy-(5Z,8Z,11Z,14Z)-eicosatetraenoic acid	C20 H32 O3	320.23471	8.318	319.2273	Full match	No results	Full match	5706118.056	4445001.722	5495159.682	5972854.683	7436947.197	4505830.998	24625090.54	53621021.07	23295736.27	38168794.16	20624350.93	32842315.3	0.173736	-2.52503	9.65E-06	1	1.266072	down
Com_1740_neg	4-Methylphenol	C7 H8 O	108.05744	5.344	107.0502	Full match	No results	Full match	2713326.043	3552170.022	2562867.298	2457988.061	2352238.263	2493295.382	7286375.27	12470096.62	7718286.707	7678595.348	44513226.44	13347802.8	0.173434	-2.52754	0.002499	1	1.135979	down

Com_5524_neg	17(S)-HpDHA	C22 H32 O4	360.22727	7.898	359.2199	Invalid mass	No results	No results	1441201.982	1505668.347	1029229.037	1281138.251	1079932.788	1217315.61	9638044.134	9228803.505	9037990.029	6644072.6	1457240.588	7683731.17	0.172912	-2.53189	0.002441	0.972	1.138577	down
Com_3003_pos	2-[(4-nitrobenzyl)thio]pyridine	C12 H10 N2 O2 S	246.05043	5.374	247.0578	Invalid mass	No results	No results	4848692.312	3414483.739	4342203.319	5639532.934	5231928.363	4615198.437	17965506.91	30061986.62	30185394.06	22533051.99	26078929.81	37175265.5	0.171293	-2.54546	2.63E-07	1	1.291543	down
Com_6710_pos	Eriodictyol	C15 H12 O6	288.06316	7.853	289.0704	Full match	No results	No results	629545.0475	665163.6979	942167.9577	894808.4201	683084.0959	662463.8794	3269297.899	9612885.009	2992399.535	2146071.098	821050.6786	7309479.25	0.171206	-2.5462	0.00843	0.944	1.045816	down
Com_7871_pos	6β-Oxycodol N-oxide	C18 H23 N O5	333.15746	5.21	334.1647	Full match	No results	No results	1292830.485	1027345.662	1288964.257	827797.3664	1013680.781	1035254.6	8626824.034	6848671.496	6449550.972	8431006.056	834307.2977	6907953.32	0.17024	-2.55435	0.007251	0.861	1.059168	down
Com_3012_neg	O-Phospho-L-serine	C3 H8 N O6 P	185.00872	1.288	184.0013	No results	No results	Full match	1335005.068	1415414.215	2321968.818	2145995.856	2620705.131	4257365.874	7814137.787	21111479.95	10452101.2	18357913.34	4495412.063	20654328.6	0.170072	-2.55579	0.000401	1	1.149008	down
Com_4077_pos	6-amino-4-(3,4-dimethoxyphenyl)-2-thioxo-2H-thiine-5-carbonitrile	C14 H12 N2 O2 S2	168.0326	5.185	169.0398	Invalid mass	No results	No results	3003694.645	2421761.27	3780625.035	4246669.405	3268930.458	3689635.161	24031797.27	19926388.26	20804981.88	14203623.83	23768544.02	20988074.7	0.164975	-2.59968	1.91E-08	1	1.299598	down
Com_1574_pos	17beta-Trenbolone	C18 H22 O2	270.16165	7.111	271.1689	Full match	No results	Full match	8251525.379	9122424.055	5414577.387	5788103.982	8189761.213	9171097.965	44083615.92	64746709.11	56228243.47	54292455.47	26332070.65	35214626.3	0.163538	-2.6123	2.22E-06	1	1.270644	down
Com_4558_neg	Chrysin	C15 H10 O4	254.05754	6.908	253.0503	Full match	No results	No results	715358.6034	642209.2128	696886.253	704459.9873	619637.1594	1273354.037	14236792.45	2962835.109	3769400.289	3775974.066	763978.1541	3307614.92	0.161431	-2.63101	0.009292	0.972	1.020625	down
Com_1566_pos	2-Thio-acetyl MAGE	C21 H42 O3 S	396.26583	7.612	397.2731	Invalid mass	No results	No results	4109170.722	3990727.513	3252394.572	4119111.178	4216936.127	3684860.413	18590063.57	11680562.67	12038936.18	12275261.01	76821831.86	13635721.1	0.161147	-2.63355	0.00346	1	1.124871	down
Com_330_neg	Cyclohexaneacetic acid	C8 H14 O2	142.09912	5.308	187.0973	No results	No results	Full match	19298101.02	15603950.95	13017930.53	12000041.48	15147666.06	13216737.93	41880328.81	46466350.57	51655255.05	52606862.46	277384438.5	79509690.3	0.160662	-2.6379	0.002356	1	1.13627	down
Com_2591_pos	Heptadecanoic Acid	C17 H34 O2	292.23989	7.821	293.2471	Invalid mass	No results	No results	4945236.088	4326665.67	4587844.378	6251632.109	4599280.456	4506421.234	43330344.13	36311455.74	27886117.09	36761240.27	15379310.57	23369592.1	0.159623	-2.64726	2.77E-05	1	1.272194	down
Com_11209_pos	Glycohyocholic acid Sodium salt	C26 H42 N Na O6	487.29158	9.662	488.2989	No results	No results	Full match	457702.2371	358529.6182	472148.038	246780.8711	404560.3746	342184.2463	1779243.502	3423521.661	1334282.396	2160922.946	4278246.132	1692338.46	0.155564	-2.68442	3.23E-05	1	1.244804	down
Com_3483_neg	Benzyl 6-O-beta-D-glucopyranosyl-beta-D-glucopyranoside	C19 H28 O11	432.16282	5.586	431.1554	Full match	No results	No results	1141986.631	4708603.246	1191912.416	1239027.612	1293031.226	1366026.461	10543975.57	19025029.15	12981012.77	3536280.928	10527671.29	15756044.1	0.151176	-2.7257	0.000158	0.972	1.16733	down
Com_3383_pos	2-Amino-1,3-octadecanediol	C18 H39 N O2	301.29768	6.696	302.3049	Full match	Full match	Full match	2521048.246	1418682.043	1323136.794	1235545.933	2886425.743	1604337.279	13423448.61	26235795.92	4956751.359	4990363.547	8258552.586	15235740.9	0.150329	-2.7338	0.000506	1	1.161191	down
Com_5029_pos	Ethyl oleate	C20 H38 O2	310.28687	7.49	311.2942	Full match	No results	Full match	903164.1413	1123384.784	1775606.231	1423756.84	940465.282	955844.1809	4039588.734	6040851.111	9629331.438	17559831.45	3813154.433	6467912.77	0.149782	-2.73907	0.000216	1	1.204565	down
Com_5702_pos	2-morpholino-1-phenyl-1-ethanol	C12 H17 N O2	189.11592	6.269	190.1231	Invalid mass	No results	No results	567909.4963	779897.6745	568518.666	568816.0216	697392.2077	466158.7606	3364600.439	12589233.03	1073484.859	2973948.688	1297790.765	3712726.69	0.145879	-2.77716	0.006179	1	1.070592	down
Com_4256_pos	RNK	C16 H32 N8 O5	208.1249	7.167	209.1322	Invalid mass	No results	No results	1481828.088	1536461.414	1411152.066	2535102.64	1483108.424	1358184.245	12512928.39	10913570.43	6405228.709	4628699.173	22712529.15	10270784.1	0.145393	-2.78197	0.000173	1	1.220267	down
Com_8879_neg	N-Methylthreonine	C5 H11 N O3	133.0736	1.339	132.0663	No results	Full match	Full match	240539.3558	324833.3686	292194.9623	235361.0397	373413.0713	437656.011	916366.3431	3890018.345	1379739.859	1677949.882	2163134.487	3096205.21	0.145084	-2.78504	0.000113	1	1.225509	down
Com_3003_neg	Allantoin	C4 H6 N4 O3	158.04373	1.366	157.0363	No results	Full match	Full match	1003617.295	1288232.471	2406687.692	1742859.163	2075553.838	1881955.895	2424282.85	23452647	6536085.197	9391754.549	8718320.672	21814107.9	0.143756	-2.79831	0.002739	1	1.099402	down
Com_1207_neg	2-Hydroxyphenylacetic acid	C8 H8 O3	152.04704	5.478	151.0398	Full match	Full match	Full match	5667469.649	6909195.14	6134270.434	6614160.987	5407482.373	5163646.068	20408883.31	48829479.88	59598064.33	55650862.07	18126405.78	54598364	0.139559	-2.84105	0.000282	1	1.236747	down
Com_5753_pos	4-morpholinobenzoic acid	C11 H13 N O3	207.0893	1.352	208.0966	Full match	No results	Full match	1077450.313	1931931.067	1120084.108	1023037.713	1448634.881	1655169.031	13964913.36	9920364.371	6871242.135	9277083.536	4079765.568	15165980.1	0.139278	-2.84396	3.63E-05	1	1.241829	down
Com_6766_pos	FQH	C20 H26 N6 O5	860.40109	8.249	861.4089	Invalid mass	No results	No results	665386.2384	899678.0075	1109193.065	1301444.551	569310.7851	686514.2777	10596734.71	9486973.536	5785570.039	1015099.874	2006068.807	8718087.9	0.139105	-2.84576	0.005821	0.944	1.057026	down
Com_233_pos	(+/-)11(12)-EET	C20 H32 O3	302.22429	7.633	303.2316	Invalid mass	No match	No match	19258421.81	17610783.89	26932285.05	25945021.68	16205643.01	19728280.79	103653138.6	41121720.85	63218349.27	129729170.5	522226941.5	53404024	0.137603	-2.86141	0.007513	1	1.051883	down
Com_1845_pos	(+)-ar-Turmerone	C15 H20 O	216.1512	7.409	217.1585	Full match	No results	No results	4038907.089	3723232.676	3431854.799	4142712.588	3959705.111	3988704.477	7732131.791	13440414.5	40604169.84	61921587.63	38288660.28	9500392.97	0.135783	-2.88062	0.004771	1	1.107652	down
Com_4867_neg	Epoxomicin	C28 H50 N4 O7	554.36665	8.665	553.3589	No results	No results	Full match	834950.9957	878045.7761	681714.1002	1108670.645	956660.9528	1183610.695	7087879.14	4089304.334	7945503.188	8852801.963	8739859.393	5116759.92	0.134912	-2.88991	6.89E-07	1	1.28753	down
Com_14038_pos	4-Hexyloxyaniline	C12 H19 N O	193.1465	1.428	194.1537	Full match	No results	No results	148105.6744	196087.9686	125878.7016	151133.9427	217725.2857	230377.0635	1055652.88	1746215.587	472001.0646	714326.5037	1456995.552	2523461.71	0.134189	-2.89766	0.000287	1	1.207696	down
Com_2891_pos	Tetranor-12R-HETE	C16 H26 O3	248.17735	9.728	249.1846	Invalid mass	No results	No results	2478085.196	2332522.442	1559456.717	1343907.072	3481146.016	3739978.769	37772262.27	22487338.88	13191774.5	12299031.68	9835762.911	17271508.2	0.132336	-2.91773	2.10E-05	1	1.223364	down
Com_574_pos	Avocadyne 1-acetate	C19 H34 O4	308.23478	7.95	309.2421	Invalid mass	No results	No results	13842507.38	10810959.8	11930078.31	19268137.68	9876114.515	9828967.277	75143036.86	190043224.4	52949047.13	122383351.6	45780194.74	97431423.3	0.129438	-2.94967	7.21E-05	1	1.233702	down
Com_338_neg	Azelaic acid	C9 H16 O4	188.10452	5.291	187.0972	Full match	No results	Full match	16704120.82	10125785.82	8012778.162	7419030.953	9832188.7	8989258.559	36410605.88	37886244.1	42349944.56	50254590.82	272020168.8	35012068.8	0.128885	-2.95584	0.002115	1	1.121149	down
Com_2543_neg	5-[4-(2-furylcarbonyl)piperazino]-5-oxo-3-phenylpentanoic acid	C20 H22 N2 O5	370.14723	1.447	369.1401	Invalid mass	No results	No results	798248.0503	780534.2029	1557441.567	1245324.212	1076055.751	1000717.907	5063375.591	5831034.795	7199627.128	2420608.621	27701323.93	3719778.11	0.124352	-3.0075	0.002524	1	1.117963	down
Com_1945_pos	cis-7-Hexadecenoic Acid	C16 H30 O2	254.22434	8.157	255.2316	No results	No results	Full match	4350420.024	3929872.458	3652678.636	4012444.118	5126039.12	4444989.151	60683260.86	25000715.11	35337231.24	28458190.11	29411116.25	27413975	0.123683	-3.01528	4.25E-06	1	1.294551	down
Com_7903_pos	Meperidine-d5	C15 H16 [2]H5 N O2	252.18763	10.211	253.1949	Full match	No results	No results	555716.5802	499452.2854	644712.1841	384262.0084	1003780.981	470388.0714	6137055.958	7290401.531	1751760.292	5751467.903	1540103.221	6386775.59	0.123306	-3.01968	0.000423	1	1.180059	down
Com_2715_pos	cis-gondoic acid	C20 H38 O2	310.28684	9.199	311.2941	No results	No results	Full match	2290411.626	2786476.405	3792662.208	5332542.127	1144307.921	1257739.95	24591412.16	14497283.01	24883564.44	39623316.77	19149779.16	13128690.2	0.122202	-3.03265	5.26E-05	1	1.216513	down
Com_2864_neg	Glycoursodeoxycholic acid	C26 H43 N O5	449.31378	7.14	448.3069	Full match	No results	Full match	2538886.168	1380054.756	2936107.505	3693231.641	1315408.007	1090408.926	25547677.61	24567765.35	8943939.917	5354561.469	20003244.75	24931945.1	0.118465	-3.07746	0.000143	1	1.179498	down
Com_10879_pos	2-(2,4-dihydroxyphenyl)-3,5,7-trihydroxy-4H-chromen-4-one	C15 H10 O7	302.04547	5.319	303.0523	Invalid mass	No results	No results	460138.6994	472052.3053	430031.0703	400995.8743	387773.4063	423193.396	3655921.722	3869130.056	4246274.462	2975968.952	3409859.062	3650273.91	0.118042	-3.08263	1.23E-10	1	1.31976	down
Com_596_pos	RKK	C18 H38 N8 O4	430.30535	11.35	431.3126	Invalid mass	No results	No results	10568995	13255531.75	17161203.07	11054703.97	24764993.57	9445963.073	141231357.4	63719976.6	154647579.4	16349827.53	132734942.2	224509852	0.117638	-3.08757	0.002891	0.944	1.098219	down
Com_1643_neg	8-iso-15-keto Prostaglandin E2	C20 H30 O5	332.1986	7.872	331.1911	Invalid mass	No results	No results	5046604.868	3944805.003	3932470.915	3290977.883	5047868.862	4675345.765	35052428.46	49206050.58	42106446.67	28140004.58	27996104.01	39557829.4	0.116807	-3.0978	1.20E-08	1	1.30571	down
Com_144_neg	13(S)-HOTrE	C18 H30 O3	294.21919	7.891	293.2119	Full match	No results	Full match	47744788.39	44423526.65	30445586.58	42825433.57	61457156.12	41489573.91	347235763.1	338852731.2	520561820.2	505348926.9	346927623.7	264869123	0.115495	-3.1141	3.71E-08	1	1.297259	down
Com_4064_pos	Phenylacetylglycine	C10 H11 N O3	193.07381	1.911	194.081	Full match	Full match	Full match	1510096.081	1838684.001	1374149.118	1345329.659	1589783.008	1650106.456	6083967.541	20392446.93	9351689.694	13433853.07	8373726.092	23161583.4	0.115204	-3.11774	0.00014	1	1.254769	down
Com_5131_neg	(3R)-4,4-Dimethyl-2-oxotetrahydro-3-furanyl beta-D-glucopyranoside	C12 H20 O8	338.11483	7.285	337.1074	Invalid mass	No results	No results	657467.4773	607647.9756	738318.7415	764337.3878	666517.6409	671906.1766	4676082.48	6030607.422	7731348.282	8143070.62	5854166.798	3862767.99	0.113124	-3.14402	2.74E-06	1	1.304013	down
Com_6974_pos	2-deoxyglucose-6-phosphate	C6 H13 O8 P	244.03475	5.461	245.0419	No results	No results	Full match	705545.1446	726901.6974	707473.7811	650850.6902	1038453.35	854963.8443	4734422.199	8422301.245	7130843.034	6049816.4	4053630.452	11101993.8	0.112891	-3.147	3.99E-06	1	1.285896	down
Com_7706_neg	Narasin	C43 H72 O11	746.50859	9.333	747.5159	Invalid mass	No results	No results	395361.6125	455592.3631	368424.0448	227462.1799	1044156.285	345953.9043	4763876.868	1422535.535	3201504.141	1614717.369	9049301.773	5431963.53	0.111323	-3.16717	0.000244	1	1.166214	down
Com_12902_pos	LDGTS 20:0	C30 H59 N O6	529.43406	10.29	530.4412	No results	No results	Full match	127437.2731	142288.4561	124176.7303	112643.6335	139145.8208	132015.5766	938770.7765	758174.3403	416252.8653	914655.0874	3045670.622	956144.702	0.110632	-3.17616	0.00056	1	1.221936	down
Com_6572_neg	Deoxycorticosterone	C21 H30 O3	330.21909	9.134	329.2119	No results	No results	Full match	339309.8327	323841.2717	344410.0692	406988.2053	343749.7917	369175.1882	5829098.698	1094006.501	5313636.224	5378101.105	786041.1914	859553.211	0.110458	-3.17843	0.006105	1	1.086361	down
Com_5834_pos	1-hydroxy-1-(4-methoxyphenyl)propan-2-yl 4-methoxybenzoate	C18 H20 O5	338.11485	7.202	339.1221	Invalid mass	No results	No results	892340.8176	730481.5248	1070929.008	1349629.342	1333638.311	1039643.621	10784405.94	7805754.942	11097284.72	13910909.38	9541828.328	6409947.31	0.107752	-3.21421	4.52E-08	1	1.296335	down
Com_2685_pos	N-Acetylsphingosine	C20 H39 N O3	363.2742	8.48	364.2814	Invalid mass	No results	No results	2012356.336	2143289.846	1621070.722	1745296.013	4395081.71	2127433.261	41311007.84	29792834.4	21795110.85	2210120.221	21816939.24	14814283.8	0.106608	-3.22962	0.003969	0.972	1.084479	down
Com_721_neg	D-(-)-Mannitol	C6 H14 O6	182.07882	1.328	181.0715	Full match	Full match	Full match	282974.3171	286214.0669	3928192.542	872592.6178	20260843.17	1018655.484	22115809.62	36394873.56	34838049.55	22753167.51	116387676.3	35311808.1	0.099512	-3.32898	0.003111	1	1.094625	down
Com_5190_neg	Bilirubin	C33 H36 N4 O6	584.26466	7.571	583.2574	No results	No results	Full match	508168.7127	484159.3628	597439.421	715171.1509	920250.7463	536326.6727	6711459.851	6437135.694	7784269.038	7983891.413	4968221.958	5092341.52	0.096505	-3.37325	8.69E-09	1	1.304925	down
Com_2504_pos	Betulin	C30 H50 O2	442.38113	10.672	443.3879	Full match	No results	No results	1928068.837	2232438.004	2383912.331	2244335.492	3965322.374	1544355.132	44875993.73	32381173.65	14782469.49	15577130.61	18746131.39	25943962.5	0.093879	-3.41305	2.20E-06	1	1.269067	down
Com_7420_neg	FAHFA (4:0/16:0)	C20 H38 O4	342.27674	11.817	341.2694	No results	Full match	No results	217068.3566	200624.9523	321844.8042	240189.1173	316236.2962	173220.3558	1002752.873	3765831.439	1415877.539	503645.9577	5316471.281	3827137.62	0.0928	-3.42973	0.001979	1	1.143112	down
Com_8603_pos	(2E)-1-(2-hydroxy-3,4,5,6-tetramethoxyphenyl)-3-phenylprop-2-en-1-one	C19 H20 O6	366.11002	7.684	367.1173	Invalid mass	No results	No results	209601.8229	224162.4297	221280.0625	318530.8244	306617.2437	258716.1158	699144.7956	1499219.523	6098472.168	7177532.099	427054.4213	928417.243	0.091439	-3.45104	0.010829	1	1.026178	down
Com_2899_pos	Orlistat	C29 H53 N O5	533.343	9.886	534.3503	Invalid mass	No results	No results	1439316.944	1196499.63	1170728.975	770978.5999	1258650.371	1022873.705	4385112.597	7413703.122	10596242.37	36563570.88	13642407.6	5384310.83	0.087953	-3.50712	0.000628	1	1.200354	down
Com_159_neg	β-Muricholic acid	C24 H40 O5	816.57505	7.586	797.5573	Invalid mass	No results	No results	5567093.727	5149430.814	20788410.54	38187024.59	8426103.248	12465597.1	71646537.3	151065896.2	70616946.79	476310079.3	160386803.1	126858731	0.085708	-3.54442	0.000179	1	1.163823	down
Com_2385_pos	alpha-Farnesene	C15 H24	204.18765	7.732	205.1949	No results	No results	Full match	1804679.188	1914513.246	1387378.558	2013379.694	1789749.776	1829293.313	19519429.78	11906853.61	22129798.64	46207270.18	6752285.241	20711242.1	0.084408	-3.56647	0.000239	1	1.244	down
Com_987_neg	FAHFA (24:4/6:0)	C30 H50 O4	474.37039	9.734	473.363	No results	Full match	No results	3174110.87	2699196.058	2664742.763	2261109.352	2750933.907	2289118.064	17395877.65	22487124.69	49283087.65	69059936.39	9970891.808	24391682.2	0.082244	-3.60395	0.00039	1	1.230524	down
Com_2596_pos	Fmoc-L-Isoleucine	C21 H23 N O4	353.1626	5.37	354.1698	No results	No results	Full match	1989356.321	1874976.389	1968752.782	1803417.425	1740870.617	1920641.843	43148062.71	11508901.53	36426230.36	23126530.11	11833248.67	12023986	0.08183	-3.61123	0.000198	1	1.257576	down
Com_5206_pos	N-allyl-2-(2-methyl-1-benzofuran-3-yl)acetamide	C14 H15 N O2	251.09471	5.001	252.102	Invalid mass	No results	No results	634302.6653	581229.7003	398979.1077	688626.7236	550256.5788	505590.7615	4569346.17	3419358.622	6654540.851	16647216.45	6185021.16	5156493.48	0.07879	-3.66584	3.78E-05	1	1.267883	down
Com_4492_pos	Kaempferol	C15 H10 O6	286.04744	5.905	287.0547	Full match	Full match	No results	1055370.202	1277404.599	836803.6037	731806.9977	1069418.932	1251865.945	9643384.496	17821817.2	19335724.82	3622060.507	9888210.751	20772472.6	0.076744	-3.70381	0.000129	1	1.242783	down
Com_76_neg	Glycochenodeoxycholic acid	C26 H43 N O5	449.31351	7.745	448.3062	Full match	No results	Full match	41580149.31	37836552.88	110678906.9	84940354.91	62940593.36	70582879.44	1523521007	907008538.4	873079366.7	288614726	915265880.6	908110531	0.075441	-3.7285	6.41E-06	1	1.251676	down
Com_2368_pos	1,7-bis(4-hydroxyphenyl)heptan-3-one	C19 H22 O3	298.1566	6.526	299.164	Full match	No results	No results	1744290.354	2006469.678	1388710.928	1212790.525	1155855.292	1430649.926	25990615.36	17375956.33	22751943.55	46658224.2	2742304.856	10999800.5	0.070652	-3.82313	0.001571	1	1.164391	down
Com_2074_pos	3-Methoxy prostaglandin F1α	C21 H38 O6	368.25595	8.386	369.2629	Invalid mass	No results	No results	2224806.548	2006462.728	2828081.181	3494025.465	2326836.312	1912817.26	50801878.83	38991866.79	23873586.87	7076428.151	31516901.37	57821307.9	0.070416	-3.82796	0.000284	1	1.225567	down
Com_1331_neg	Tretinoin	C20 H28 O2	300.2086	9.208	299.2015	Full match	No results	Full match	1741977.107	1234406.12	875025.5204	828975.3378	1065627.78	1717539.035	12565043.55	6397682.17	8188026.245	8988912.106	60997776.46	10602358.6	0.069274	-3.85155	0.000401	1	1.195467	down

Com_3019_neg	4-morpholino-3-nitrobenzene-1-sulfonamide	C10 H13 N3 O5 S	288.06068	1.657	287.0535	Invalid mass	No results	No match	799963.8237	710835.1509	875043.6825	1165180.134	721851.6493	892276.6153	21340840.94	16338434.97	7489232.741	1268549.426	4912957.573	23404210	0.069095	-3.85527	0.00358	1	1.121728	down
Com_2504_neg	DGMG (18:2)	C33 H58 O14	678.38273	8.92	677.3739	No results	No results	Full match	756039.9639	1600810.724	1447197.794	1481496.042	1273339.851	1026313.266	24641174.59	22081025.13	30417976.38	632127.7695	13521038.98	19435030.3	0.068503	-3.86769	0.011848	0.833	1.016419	down
Com_4443_pos	(6E,10E)-3,7,11,15-tetramethylhexadeca-1,6,10,14-tetraene-3,5,9-triol	C20 H34 O3	339.27685	6.153	340.2841	Invalid mass	No results	No match	919241.3028	963321.0231	808116.0694	769485.4843	725174.8827	953949.4996	9326300.837	18066032.35	14598204.91	4908771.748	12814267.16	16370332.9	0.067548	-3.88795	2.18E-05	1	1.286751	down
Com_3806_pos	Trehalose	C12 H22 O11	342.11776	5.603	685.2429	No results	No results	Full match	714566.5607	725743.7151	632672.2355	607296.7144	695549.0878	703121.1107	26569638.75	7417740.432	7432945.934	12596462.63	2045488.403	4740264.89	0.067085	-3.89786	0.001019	1	1.200187	down
Com_14_neg	Glycodeoxycholic acid	C26 H43 N O5	449.3136	7.915	448.3064	Full match	No results	Full match	113777871.7	94898007.85	284378415.8	224571516.1	96713681.16	122343555.5	2877109611	2718484246	607871183	228766714.6	4893011013	2687367197	0.066846	-3.90302	0.002783	0.972	1.097844	down
Com_13530_pos	N1-pyrazin-2-yl-4-chlorobenzamide	C11 H8 Cl N3 O	215.0252	1.883	216.0326	Invalid mass	No results	No results	101544.0181	129629.3488	72398.25716	156844.2105	126488.0578	98471.98945	695500.6663	1676374.085	2953568.307	2220037.676	773260.921	2010235.18	0.066355	-3.91366	2.33E-05	1	1.261349	down
Com_711_pos	7-(2-hydroxypropan-2-yl)-1,4a-dimethyl-decahydronaphthalen-1-ol	C15 H28 O2	222.19822	7.734	223.2055	Invalid mass	No results	No results	5078660.743	6339568.197	3901979.117	8110218.142	5522597.531	3935568.104	82785862.1	60015717.33	83041714.53	175462148.9	27493974.24	70357670.1	0.065888	-3.92383	2.42E-05	1	1.259387	down
Com_4156_neg	Xylitol	C5 H12 O5	152.06824	1.337	151.061	Full match	Full match	Full match	634709.2209	636003.0688	515669.2747	399880.4079	655506.7159	592676.337	4160915.218	7898238.643	11351217.21	6869422.896	14163376.29	8280827.85	0.06514	-3.94031	2.34E-06	1	1.292622	down
Com_9787_pos	2-[(3S)-1-(4-Chlorobenzyl)-3-pyrrolidinyl]-1-methyl-1H-benzimidazole	C19 H20 Cl N3	325.13132	5.187	326.1386	Invalid mass	No results	No results	235892.4483	241771.6285	218128.8305	190612.298	205162.6255	219463.6827	997755.1113	4850752.827	5845292.385	3153119.762	173701.4663	5272331.27	0.064605	-3.9522	0.010168	0.833	1.039069	down
Com_2137_pos	Luteolin	C15 H10 O6	286.04732	6.048	287.0545	Full match	Not the top hit	No results	2206485.223	2080173.749	2753168.731	1882680.066	1545082.156	1878511.277	34112018.16	45546789.77	37364658.2	27293782.67	15644870	31926485.4	0.06434	-3.95814	4.17E-07	1	1.299459	down
Com_5319_neg	FAHFA (20:0/22:2)	C42 H78 O4	646.58744	11.351	645.5801	No results	Full match	No results	197873.4911	329412.9179	752963.9655	100579.3036	700815.8312	143529.7859	7870728.705	5693188.063	3363983.675	7701389.755	5803758.915	5303605.83	0.062266	-4.00541	0.00012	1	1.2385	down
Com_182_neg	15(R),19(R)-Hydroxy prostaglandin F1α	C20 H36 O6	354.24035	7.414	353.2331	Invalid mass	No results	No match	8550108.675	7652818.172	7986856.305	8689373.455	8382052.036	7418700.914	110145938.4	96485965.43	25553412.13	25150883.32	483098700.1	66075132.4	0.060359	-4.05029	0.003941	1	1.121276	down
Com_1999_pos	Formononetin	C16 H12 O4	268.07316	7.158	269.0804	Full match	No results	No results	794295.8961	600567.2805	858389.8464	1121581.664	874352.4965	764218.1518	58478071.81	7037750.504	2641199.704	5009598.055	1315274.965	9524926.02	0.059679	-4.06664	0.011192	1	1.021374	down
Com_1470_pos	Nor-9-carboxy-δ9-THC	C21 H28 O4	344.19835	7.806	345.2058	Full match	No results	No results	2306765.449	2453162.226	1920238.246	2122038.096	2077770.866	1744872.55	64843158.01	12498049.04	37074842.03	81335267.19	4590773.68	12494957.4	0.059317	-4.07541	0.003214	1	1.133748	down
Com_4821_pos	THC	C21 H30 O2	314.22428	10.103	315.2315	Full match	No results	Full match	789207.198	624072.7588	569580.3253	878439.8702	601100.7582	675931.0812	16378959.35	16188522.04	10602414.98	12529215.42	3755255.53	12444449.7	0.057558	-4.11885	2.34E-05	1	1.279872	down
Com_345_neg	(±)9(10)-DiHOME	C18 H34 O4	314.24525	7.271	313.238	Full match	No results	Full match	9316406.047	8992702.989	9376271.015	13219483.23	9841517.991	8880578.959	141634177.7	167899013	248296751.2	217702562.1	129910361.3	141809919	0.056937	-4.1345	1.46E-08	1	1.312475	down
Com_3171_pos	ethyl 5-hydroxy-4-oxo-4H-chromene-2-carboxylate	C12 H10 O5	234.05264	6.391	235.06	Full match	No results	No results	1054571.548	1015028.21	757322.5492	784002.3242	967468.3816	983896.7821	4901133.787	17148112.08	20767083.75	17343079.55	4115869.302	34913909.3	0.056078	-4.15643	0.000539	1	1.219749	down
Com_1701_neg	D-3-Phenyllactic acid	C9 H10 O3	166.06347	5.817	331.1197	No results	No results	Full match	1214898.702	1995783.857	1522459.039	1200528.171	1483351.388	2262225.49	53748067.87	29032601.08	34695486.22	25715995.34	1212823.225	37897064.6	0.053095	-4.23529	0.006065	0.861	1.074974	down
Com_2735_pos	3,5,7-trihydroxy-2-phenyl-4H-chromen-4-one	C15 H10 O5	270.0526	6.275	271.0598	Full match	No results	No results	1164958.054	1375418.096	1365922.147	1075885.479	1522424.83	993213.1599	15751617.58	15935794.21	24446532.9	39275466.28	30745974.56	15561482.6	0.052907	-4.2404	1.26E-06	1	1.301161	down
Com_1617_pos	Diosmetin	C16 H12 O6	300.06317	6.264	301.0706	Full match	No results	No results	1989249.094	1550166.728	1836266.96	1407054.963	1388713.434	1303822.678	46390090.53	21861874.12	16279848.33	13256127.87	74322355.79	14739427.2	0.050711	-4.30157	0.000124	1	1.255801	down
Com_5294_pos	(5E)-7-methylidene-10-oxo-4-(propan-2-yl)undec-5-enoic acid	C15 H24 O3	274.15441	6.763	275.1617	Invalid mass	No results	No results	453682.4951	713161.7488	408579.8763	485344.85	374235.8788	476110.7239	2921536.905	6021245.446	11579080.03	16244184.25	13845675.06	7084414.4	0.050456	-4.30883	3.87E-05	1	1.267591	down
Com_258_pos	Beta-Muricholic acid	C24 H40 O5	408.28731	7.155	409.2946	No results	No results	Full match	9528948.825	8291752.185	20404475.48	18922847.62	14948196.61	10274935.46	387945182.8	275934289.1	143460579.4	52161851.28	483696740.4	292869460	0.050347	-4.31195	0.00011	1	1.226424	down
Com_7827_pos	RMK	C17 H35 N7 O4 S	433.24623	4.946	434.2535	Full match	No results	No results	208673.4651	210096.6895	187494.4839	188013.9639	219626.6242	216594.5357	8789537.992	1707647.375	2788048.818	4067415.456	5827490.287	1973329.04	0.04892	-4.35344	9.77E-05	1	1.272744	down
Com_9219_neg	17α-Hydroxyprogesterone	C21 H30 O3	330.21921	11.552	329.2118	Full match	No results	Full match	117837.4311	129396.5803	105431.2645	106224.2644	121648.2068	125698.0557	2184288.265	2496113.885	2130378.952	2822426.63	2112751.966	2913017.34	0.048178	-4.37549	5.40E-11	1	1.321367	down
Com_6260_pos	3'-Hydroxystanozolol	C21 H32 N2 O2	362.2605	10.781	363.2676	Invalid mass	No results	No results	304214.1218	239066.3346	223407.5143	228790.6662	280929.6898	211619.7516	11706700.93	10834325.18	371219.2858	659977.0516	2721815.607	5069658.51	0.047444	-4.39762	0.009177	1	1.047725	down
Com_1025_pos	Lysopa 18:0	C21 H43 O7 P	438.27477	8.218	439.2818	No results	No results	Full match	2028947.396	2418706.993	3414416.479	2964418.729	3136693.465	3304038.941	33780512.38	104141444.6	15142384.73	42930317.33	100157629.5	69544873.3	0.047217	-4.40454	0.000119	1	1.252416	down
Com_3902_pos	N1-(2-amino-2-oxoethyl)-2-(isopropylthio)acetamide	C7 H14 N2 O2 S	212.05596	5.784	213.0633	Invalid mass	No results	No results	1194115.9	1028394.551	790170.0537	652417.2907	1175134.774	998933.9048	13430583.47	17785473.3	28084862.58	21746928.2	21334451.03	21372624.4	0.047183	-4.40558	9.15E-10	1	1.311133	down
Com_1381_neg	Reserpine	C33 H40 N2 O9	608.2633	9.708	607.2564	Invalid mass	No results	No results	1481013.023	1680884.218	1668340.024	1376235.315	1321932.585	1458557.842	38364289.52	20501062.87	54253832.32	1943151.738	58142298.26	17857113.5	0.047037	-4.41006	0.003615	1	1.12801	down
Com_760_neg	2-{2-oxo-2-[(2-oxo-3-azepanyl)amino]ethoxy}acetic acid	C10 H16 N2 O5	244.10955	10.233	243.1022	Invalid mass	No results	No results	2416287.265	1647078.479	1872213.059	1873101.514	1117900.231	1187935.634	3354481.929	24906595.46	52365301.09	90618088.01	18703454.72	27652709	0.046482	-4.42718	0.001524	1	1.158823	down
Com_825_pos	2-(14,15-Epoxyeicosatrienoyl) glycerol	C23 H38 O5	394.26937	8.409	395.2766	Invalid mass	No results	No results	2467810.559	2249172.99	1612728.42	2215749.992	2094513.609	2988589.284	45368400.44	129797403.5	82179409.92	27077036.29	11304182.51	21614099.9	0.042946	-4.54133	0.000415	1	1.22238	down
Com_3546_pos	L-Glutathione (reduced)	C10 H17 N3 O6 S	307.08413	6.399	308.0914	Full match	No results	Full match	766152.4707	379039.5383	575551.8129	382847.4995	659183.4791	998310.5043	7183042.28	18142022.16	21844803.94	28612354.62	611386.0196	18970064	0.039439	-4.66422	0.004067	0.917	1.093896	down
Com_1057_pos	11-Deoxy prostaglandin F1α	C20 H36 O4	362.24265	8.292	363.25	Invalid mass	No results	No results	3977249.252	2484257.202	2104769.197	2628849.667	3925572.23	2135893.601	60853086.33	100278789	42343469.83	30229400.48	117501236	88010212.1	0.03929	-4.66971	1.78E-06	1	1.286424	down
Com_270_pos	Diosgenin	C27 H42 O3	414.31312	10.272	415.3206	Full match	No results	No results	6776008.682	8867419.088	8705847.119	6591870.592	9048762.089	7175923.842	198229126.5	387096297.7	156656429.7	121752332	30711773.05	339554142	0.038222	-4.70946	0.000405	1	1.230844	down
Com_3071_neg	Amitriptyline-d3	C20 H20 [2]H3 N	280.20136	6.412	281.2086	Full match	No results	No results	467100.4995	458508.3501	432647.4191	402075.8911	415562.0115	447386.5457	282051.0676	5802317.686	11066913.33	10986392.81	34948353.97	7385755.29	0.037225	-4.7476	0.010617	0.833	1.036168	down
Com_2581_neg	Protectin D1	C22 H32 O4	360.22723	8.09	359.2198	Invalid mass	No results	No results	703651.6953	1051990.977	985670.3074	799480.9057	827343.4028	827348.8242	31931974.28	27899227.98	24444237.61	18849595.78	14107584.04	23998252.6	0.036787	-4.76465	2.48E-08	1	1.31375	down
Com_1269_pos	(3beta,9xi)-3-(beta-D-Glucopyranosyloxy)-14-hydroxycard-20(22)-enolide	C29 H44 O9	536.29563	8.688	537.3028	Invalid mass	No results	No results	956627.202	4242613.181	1343897.398	952155.674	1768178.772	1083029.757	97937163.86	74617991.74	33473712.43	3228453.054	36129115.89	72503512.8	0.032547	-4.94131	0.000753	0.972	1.158654	down
Com_53_neg	Ursolic acid	C30 H48 O3	456.35975	9.556	455.3523	Full match	No results	No results	38221718.12	56472320.92	8314532.416	7110941.074	58166662.72	72788510.01	1966309124	1400478689	1200063406	919421454.5	925619154.1	1068205229	0.032229	-4.9555	0.000178	1	1.241679	down
Com_1145_pos	Enalaprilat	C18 H24 N2 O5	348.16976	5.115	349.177	Full match	No results	No results	986442.2073	1145380.418	926166.5406	774191.9028	1004744.675	1213198.86	108344424.6	3425315.65	36173861.78	28425080.7	12012749.23	1959848.23	0.031786	-4.97548	0.007357	1	1.068442	down
Com_810_neg	MAG (18:4)	C21 H34 O4	350.24524	8.087	349.2382	No results	No results	Full match	1256342.418	1566450.683	1293413.748	1706985.567	1938050.566	2130146.223	70499840.74	46569134.31	31560113.14	39225814.18	104316221	31770922.1	0.030534	-5.03342	1.03E-06	1	1.299486	down
Com_378_neg	MGMG (18:2)	C27 H48 O9	516.32881	7.114	515.322	No results	No results	Full match	5897131.134	4370102.235	2825436.065	3526431.197	5090370.792	3797110.119	135030847.3	139498883.4	164912978.5	194572306.6	150119898	105710143	0.028664	-5.12461	4.23E-10	1	1.315223	down
Com_3712_neg	LPC 20:0	C28 H58 N O7 P	597.40059	10.421	596.3936	No results	Invalid mass	No results	234101.8541	297390.6231	250937.5511	208449.8137	247905.9967	239570.355	11055072.77	10324117.89	2232921.414	3890946.338	16741709.41	12177845.2	0.026201	-5.25421	9.43E-05	1	1.270478	down
Com_2461_pos	15-Deoxy-Δ12,14-prostaglandin J2-2-glycerol ester	C23 H34 O5	390.23784	8.385	391.2452	Invalid mass	Invalid mass	No results	652748.0324	725950.7319	543460.9513	705232.8518	697358.323	835811.4843	38153479.02	32447570.25	20503264.09	2119035.112	27251908.95	47535238.4	0.024764	-5.33563	0.000711	1	1.213896	down
Com_2403_pos	Cynaropicrin	C19 H22 O6	368.12562	7.53	369.133	Invalid mass	No results	No results	551432.8918	603082.4397	537815.6238	686738.3949	725230.2944	507851.0163	12159994.14	39848494.7	24640793.72	34679932.14	5799748.37	31072625.5	0.024373	-5.35856	5.85E-05	1	1.276376	down
Com_2_neg	Glycocholic acid	C26 H43 N O6	465.30846	7.234	464.3008	Full match	No results	No results	145851483.5	129508510.6	287047360.8	266775004.3	205424286	154465731.6	12414064207	8968421448	3426768760	1382146179	13860952526	8847532838	0.024316	-5.36192	7.47E-05	1	1.250527	down
Com_20_neg	6-(4-methoxyphenyl)pyrimidine-2,4-diamine	C11 H12 N4 O	216.09945	5.527	215.0922	Invalid mass	No results	No results	68304287.74	66974962.37	56564496.5	52686091.28	44903983.69	44845813.07	1552383117	2710504640	3006123061	2974271747	1001218197	2938859135	0.023568	-5.407	7.09E-07	1	1.305198	down
Com_1743_neg	5α-Dihydrotestosterone	C19 H30 O2	290.22414	7.293	289.2169	Full match	No results	Full match	534015.7273	481777.8944	547919.2044	630079.2295	988797.6097	703266.2499	40473464.25	43553309.37	47424172.32	30233047.96	1069178.429	42235418.4	0.018956	-5.72117	0.001823	1	1.1612	down
Com_5071_pos	Norbutorphanol	C16 H21 N O2	259.15742	4.819	260.1648	Full match	No results	No results	106444.5969	103378.5931	90175.53596	86661.59409	108570.7034	112203.1572	6965060.074	342949.8232	7416961.545	17355213.13	243049.3918	499449.906	0.018507	-5.75582	0.012447	1	1.021601	down
Com_160_neg	1-acetyl-3-(2-furylmethylene)tetrahydropyrazine-2,5-dione	C11 H10 N2 O4	234.06766	7.856	233.0603	Invalid mass	No results	No results	6835989.185	6558128.162	6940872.489	5768413.566	6820620.937	6874248.535	239073902	420947440	557237714.4	472185277.2	115446398.8	400642786	0.018045	-5.79228	1.29E-05	1	1.300249	down
Com_2636_pos	2-hydroxy-6-[(8Z,11Z)-pentadeca-8,11,14-trien-1-yl]benzoic acid	C22 H30 O3	359.24585	5.041	360.2532	Invalid mass	No results	No results	203660.4059	214632.0245	183002.6166	188533.1308	218553.1681	230797.4209	15756597.78	763786.8755	12445822.77	12180708.78	42174531.54	840445.817	0.014724	-6.08571	0.003924	1	1.123376	down
Com_3090_pos	HKK	C18 H33 N7 O4	393.25136	4.873	394.2586	Invalid mass	No results	No results	163548.7494	173658.1919	154954.3067	148415.6389	154592.4984	150927.7802	1994163.581	1411797.803	8928528.044	16407027.9	34808143.81	1354901.78	0.014577	-6.10019	0.001626	1	1.179919	down
Com_2065_neg	Prostaglandin A3	C20 H28 O4	664.39785	7.877	663.3909	Invalid mass	No results	No results	233188.1745	270345.0324	273084.454	250697.8139	251934.4139	256533.3882	18310216.22	37642105.55	27623875.78	14349111.73	20042751.36	27772306.9	0.010538	-6.56828	3.42E-07	1	1.317922	down
Com_276_pos	Normorphine	C16 H17 N O3	271.12068	4.884	272.128	Full match	No results	No results	1909974.774	2166041.662	1641576.668	1409346.566	1726825.625	1324958.355	83597758.1	374000900.1	350165911	51136454.67	4306824.256	305415552	0.00871	-6.84311	0.00216	1	1.158617	down
Com_59_neg	18-β-Glycyrrhetinic acid	C30 H46 O4	470.33903	8.215	469.3317	Full match	No results	No results	8797463.576	6557869.029	3511923.809	3977134.902	12727368.64	13628343.05	757993834.1	1479299349	1028487345	1284941948	1184547205	755127988	0.00758	-7.0435	1.87E-07	1	1.306979	down
Com_500_pos	(+/-)5(6)-EET Ethanolamide	C22 H37 N O3	345.26665	5.175	346.2739	Invalid mass	No results	No results	196565.7109	205199.2977	186055.1179	170488.9355	185749.4097	198697.2502	37465744.06	22396689.23	10562708.82	13534395.47	255597800.1	24412388	0.00314	-8.31516	0.00011	1	1.271976	down
Com_324_pos	Morphine	C17 H19 N O3	285.13629	4.884	286.1435	Full match	No results	No results	166101.5274	317152.8388	313998.9109	318781.7537	367630.4407	316477.6766	54014785.52	329353343.7	271167571	64827290.4	2054170.002	252857558	0.001848	-9.08007	0.000773	1	1.207179	down

Supplemental Figure S1 Venn diagram of ASVs detected in captive and wild *Cervus elaphus kansuensis* based on 16S rDNA sequencing.

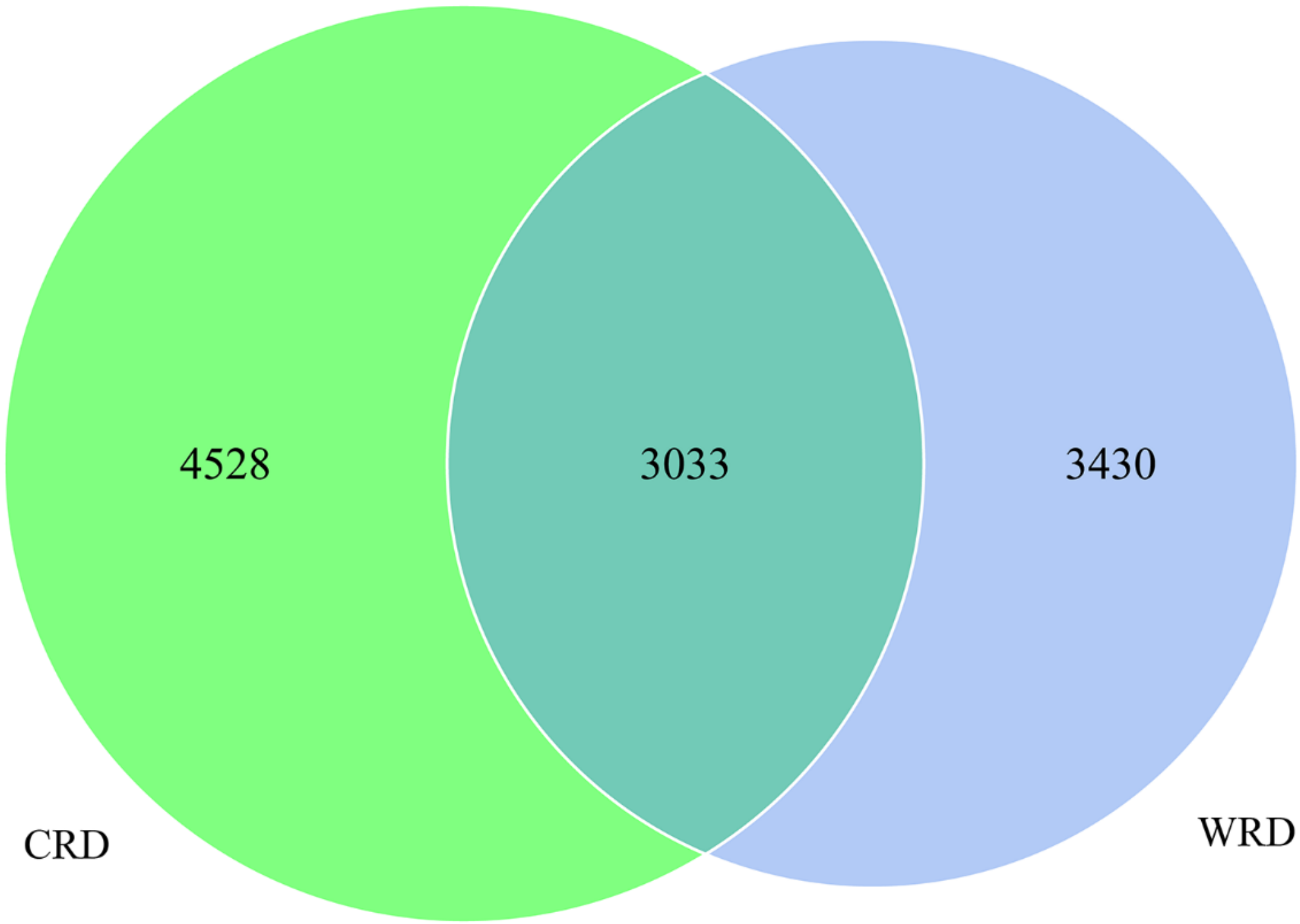

Supplemental Figure S1 Venn diagram of ASVs detected in captive and wild *Cervus elaphus kansuensis* based on 16S rDNA sequencing. Each circle represents a group of samples, and the numbers in the overlapping part of the circles represent the number of ASVs shared between different groups, while the numbers without overlapping part represent the number of ASVs specific to each group. There were 3,033 ASVs detected both in CRD and WRD. Otherwise, there were 4,528 and 3,430 specific ASVs detected in CRD and WRD, respectively.

Supplemental Figure S2 Boxplots of alpha diversity of gut microbiota detected by 16S rDNA sequencing.

S2A

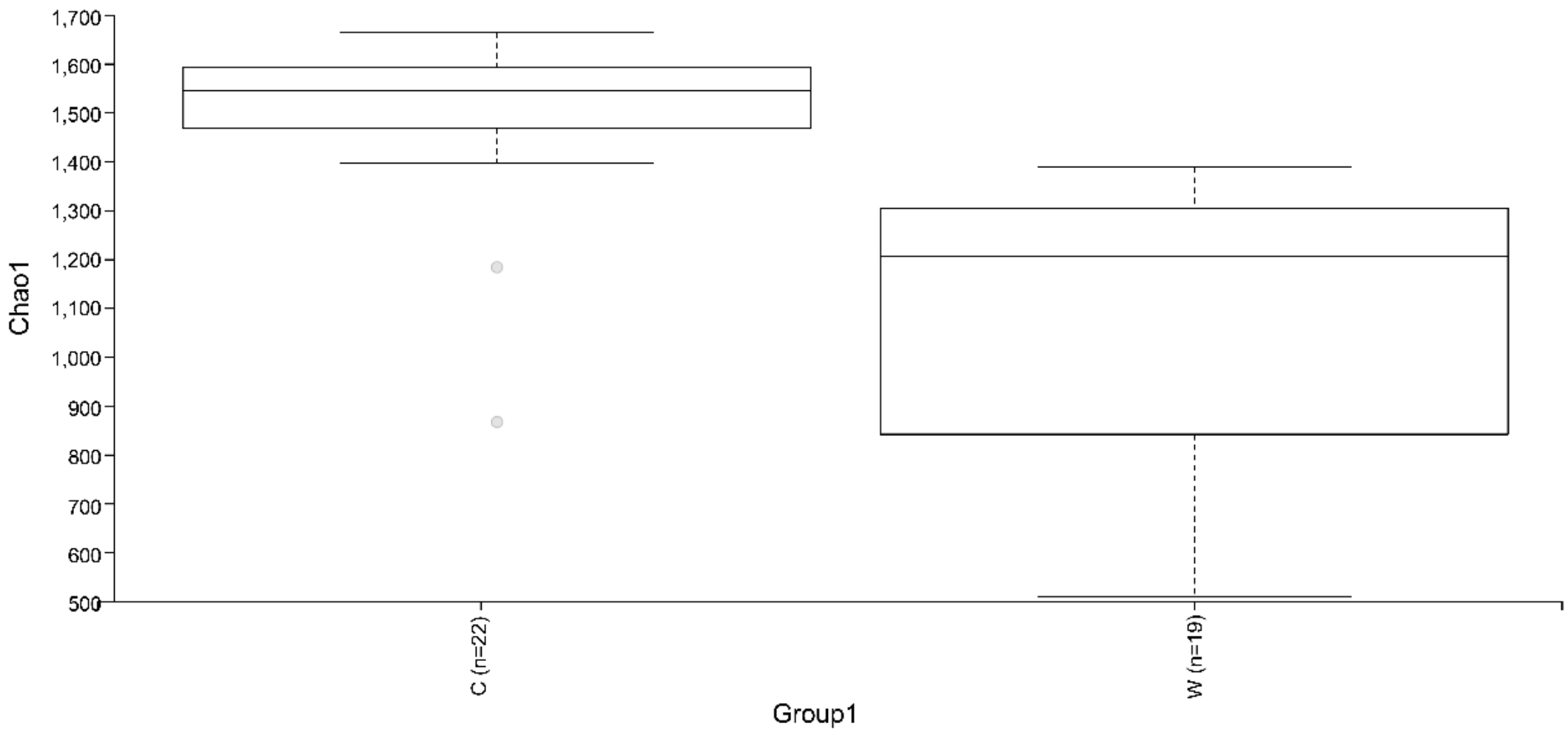

S2B

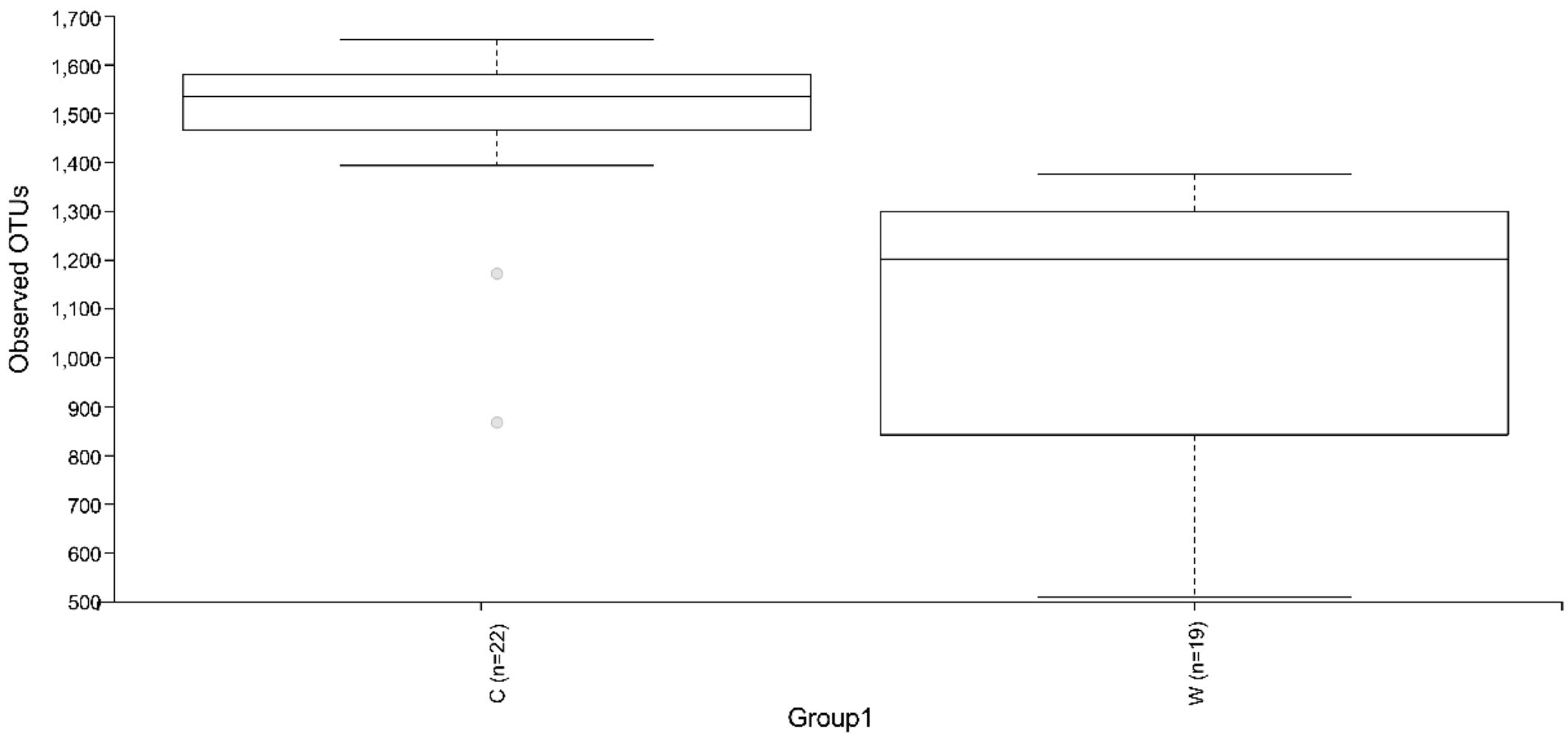

S2C

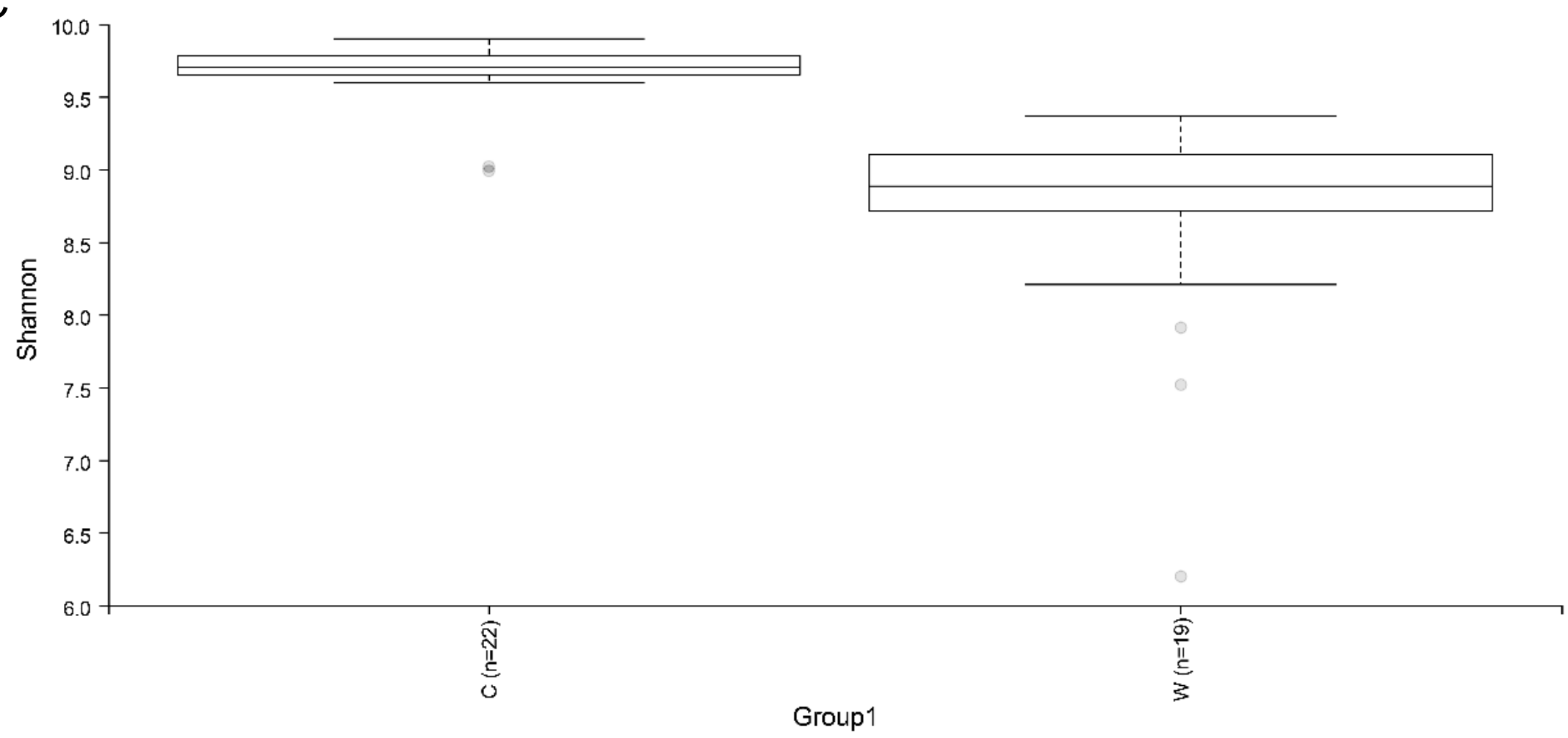

S2D

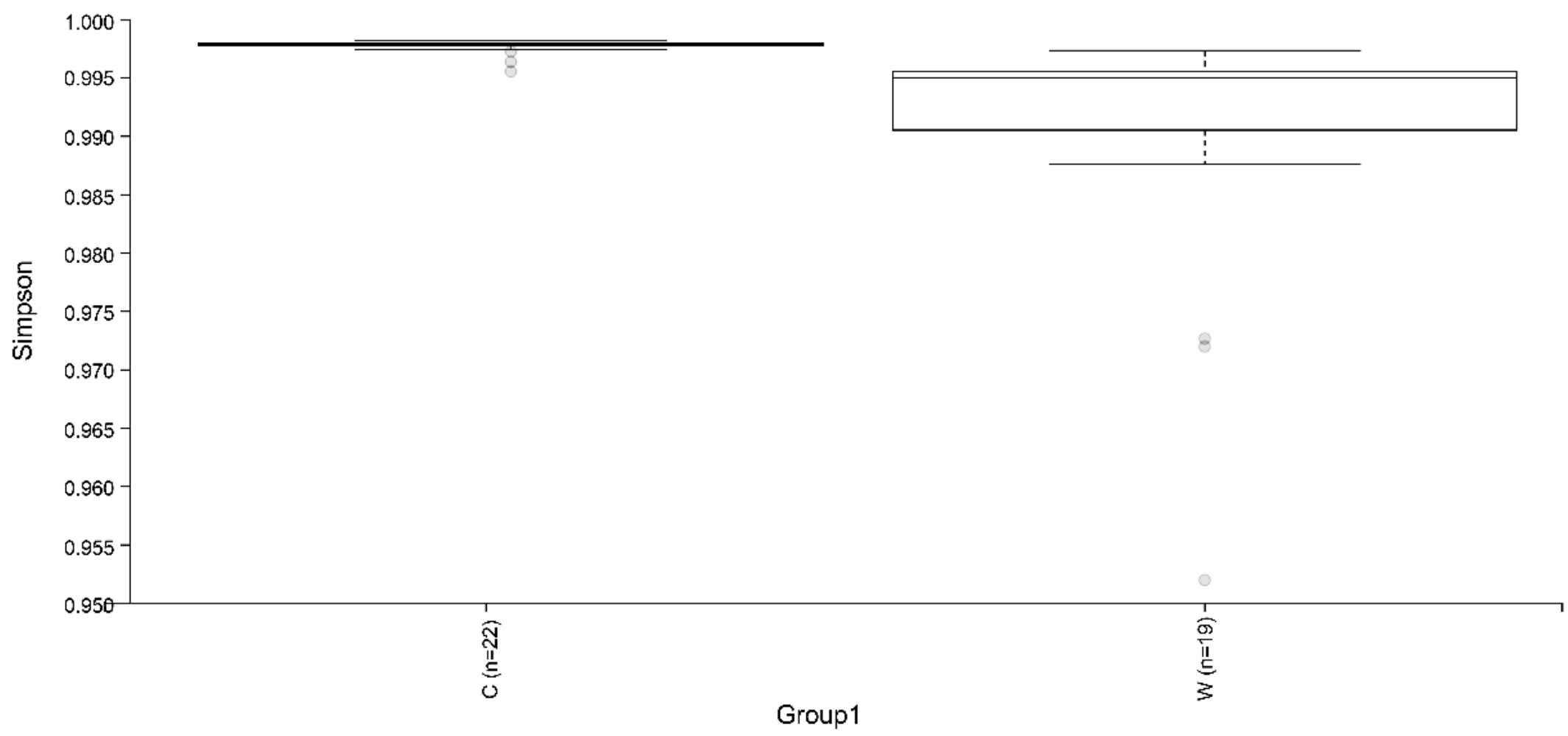

Supplemental Figure S2 Boxplots of alpha diversity of gut microbiota detected by 16S rDNA sequencing. **S2A.** Boxplot of Chao1 index. **S2B.** Boxplot of Observed OUTs index. **S2C.** Boxplot of Shannon index. **S2D.** Boxplot of Simpson index.

Supplemental Figure S3 PCoA and PCA analysis of gut microbiota detected by 16S rDNA sequencing.

S3A

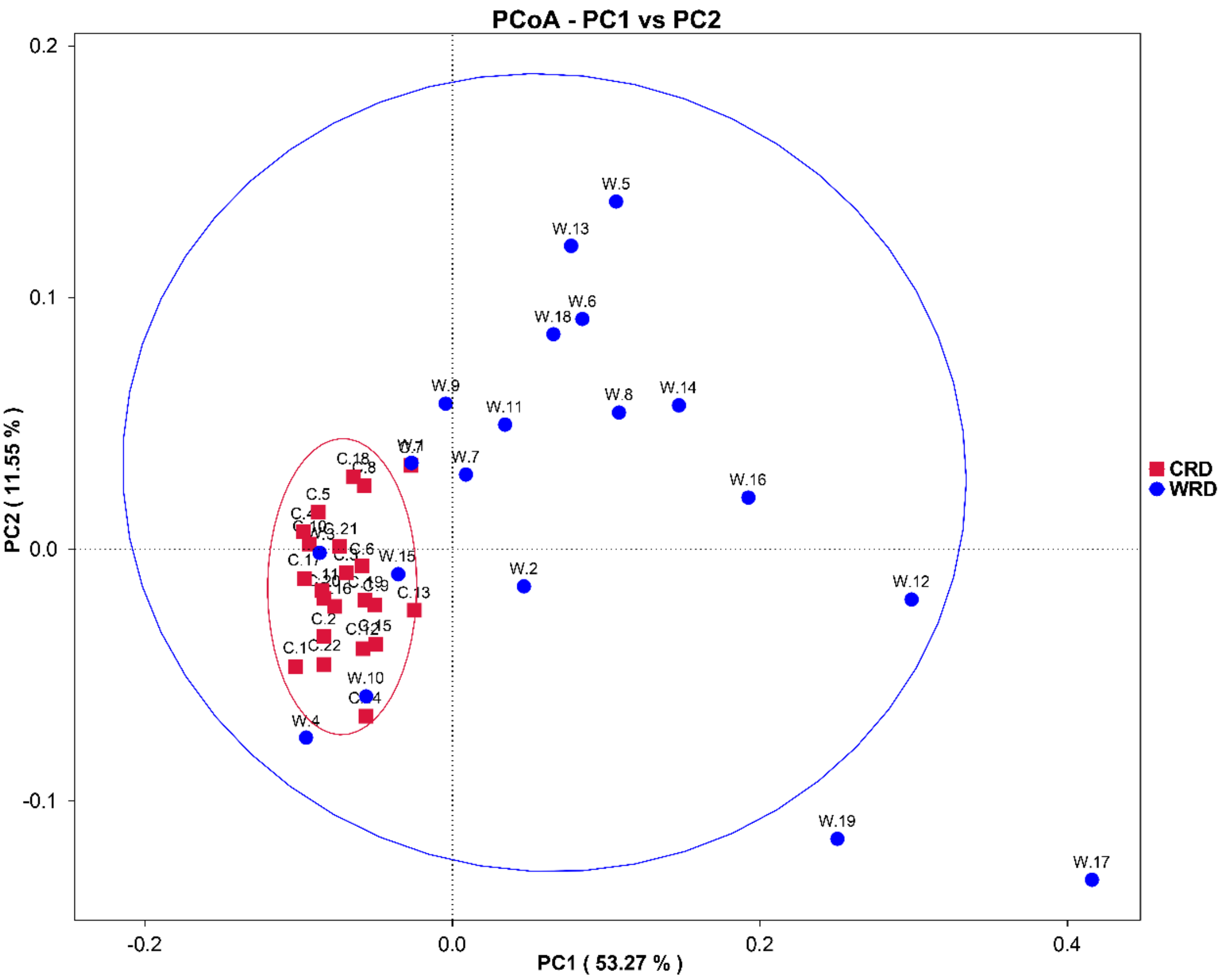

S3B

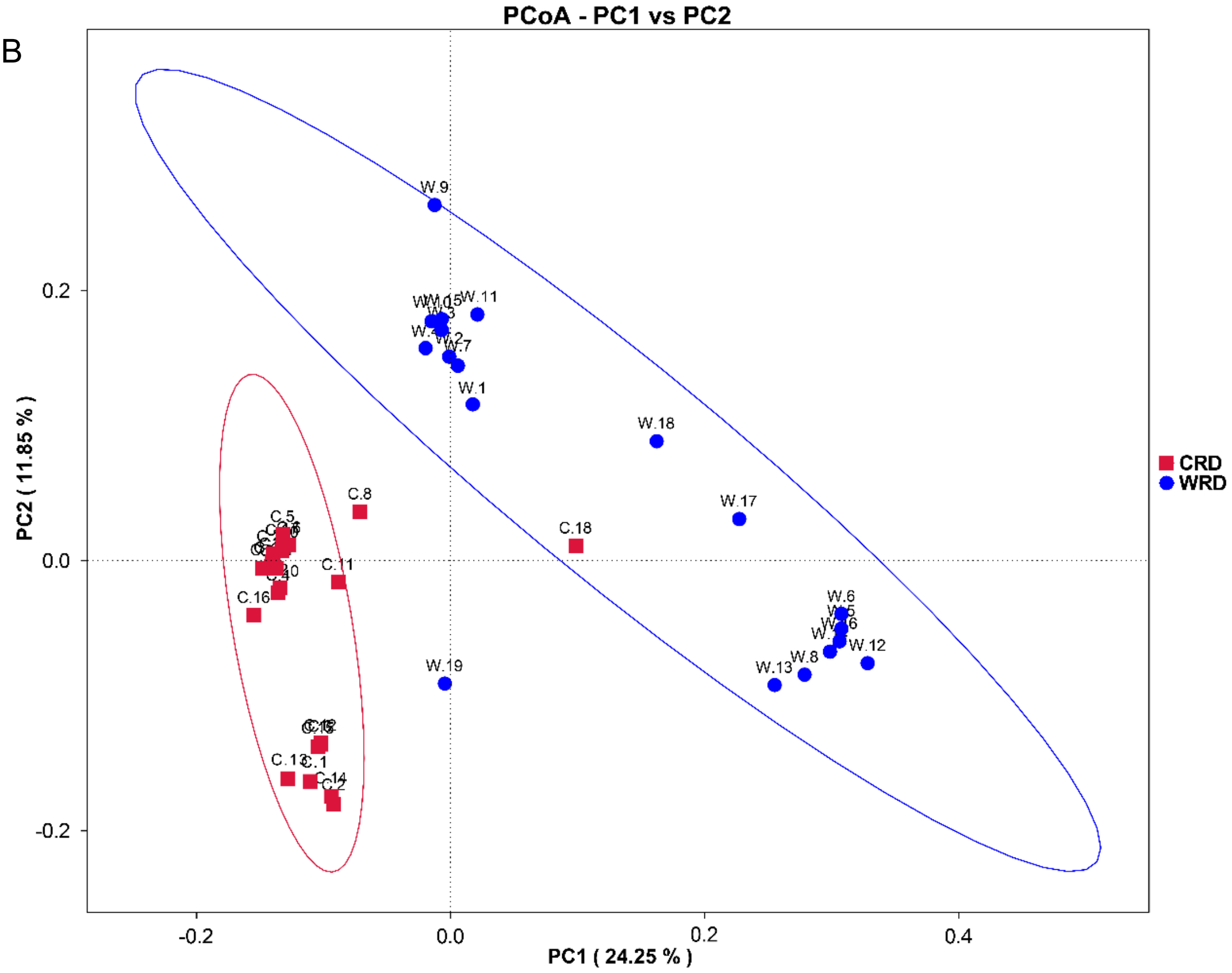
PCoA - PC1 vs PC2
PC2 (11.85 %)
PC1 (24.25 %)
CRD
WRD
0.2
0.0
-0.2
0.4

S3C

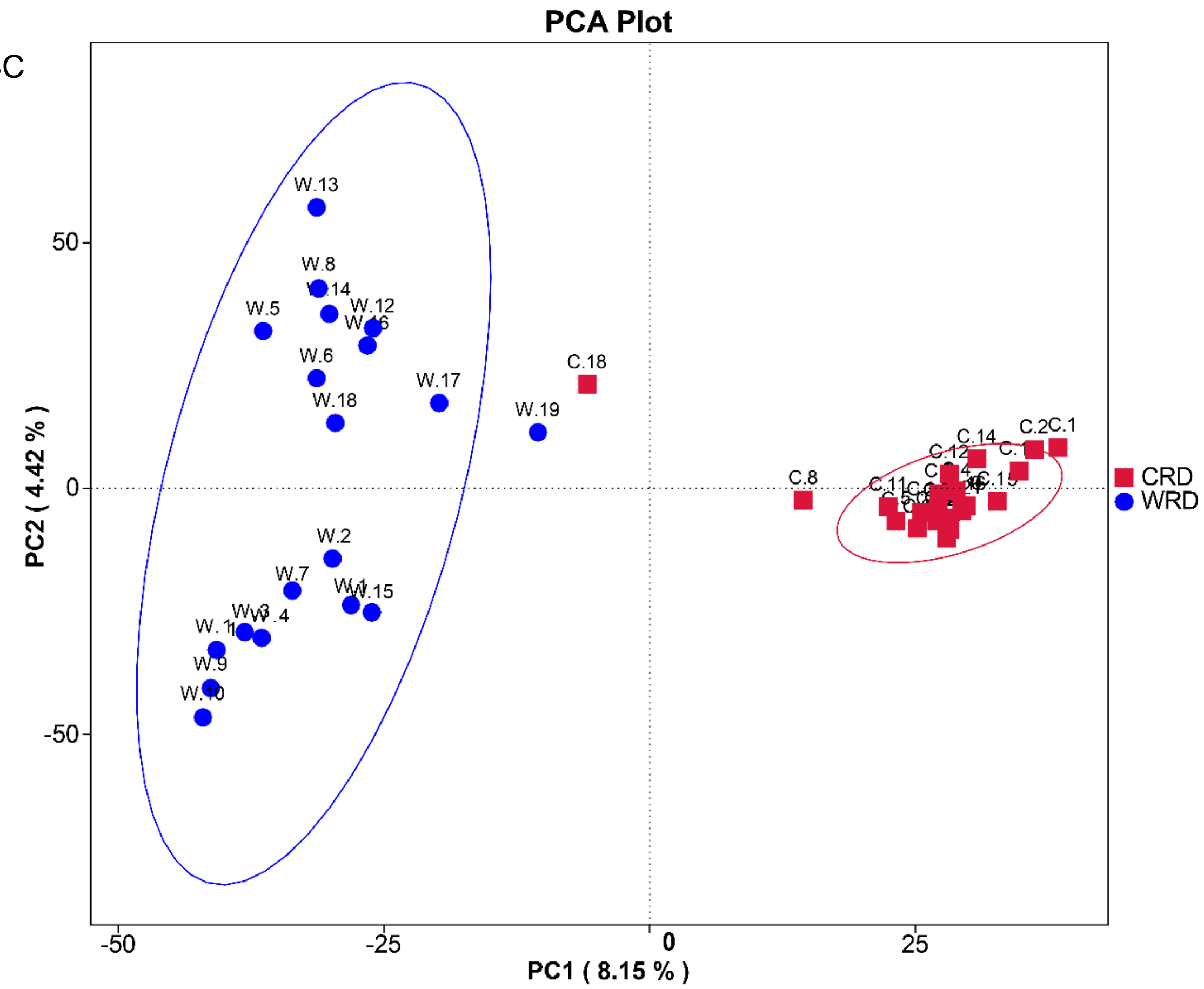
PCA Plot
PC2 (4.42 %)
PC1 (8.15 %)
CRD
WRD
50
0
-50
-25
25

Supplemental Figure S3 PCoA and PCA analysis of gut microbiota detected by 16S rDNA sequencing. **S3A.** PCoA analysis with weighted unifrac. **S3B.** PCoA analysis with unweighted unifrac. **S3C.** PCA analysis of gut microbiota detected by 16S rDNA sequencing. PCoA analysis (**S3A, S3B**) was made based on Weighted unifrac distance and Unweighted unifrac distance, and the principal coordinate combination with the largest contribution was selected for presentation. The results showed that the microbial community structure of samples within the CRD and WRD groups was very similar, but the microbial community structure of the two groups was significantly different, and the samples of the two groups could be clearly clustered into two clusters at the PC1 level. PCA analysis is also an important method to reflect the differences in microbial composition between samples. Figure **S3C** showed similar results to figure **S3A, S3B**, and CRD and WRD groups can be clearly distinguished at the PC1 level, with samples within the two groups being closer together and samples between the two groups being farther apart

Supplemental Figure S4 (A) Venn and (B) flower diagram of genes detected by metagenomic sequencing.

S4A

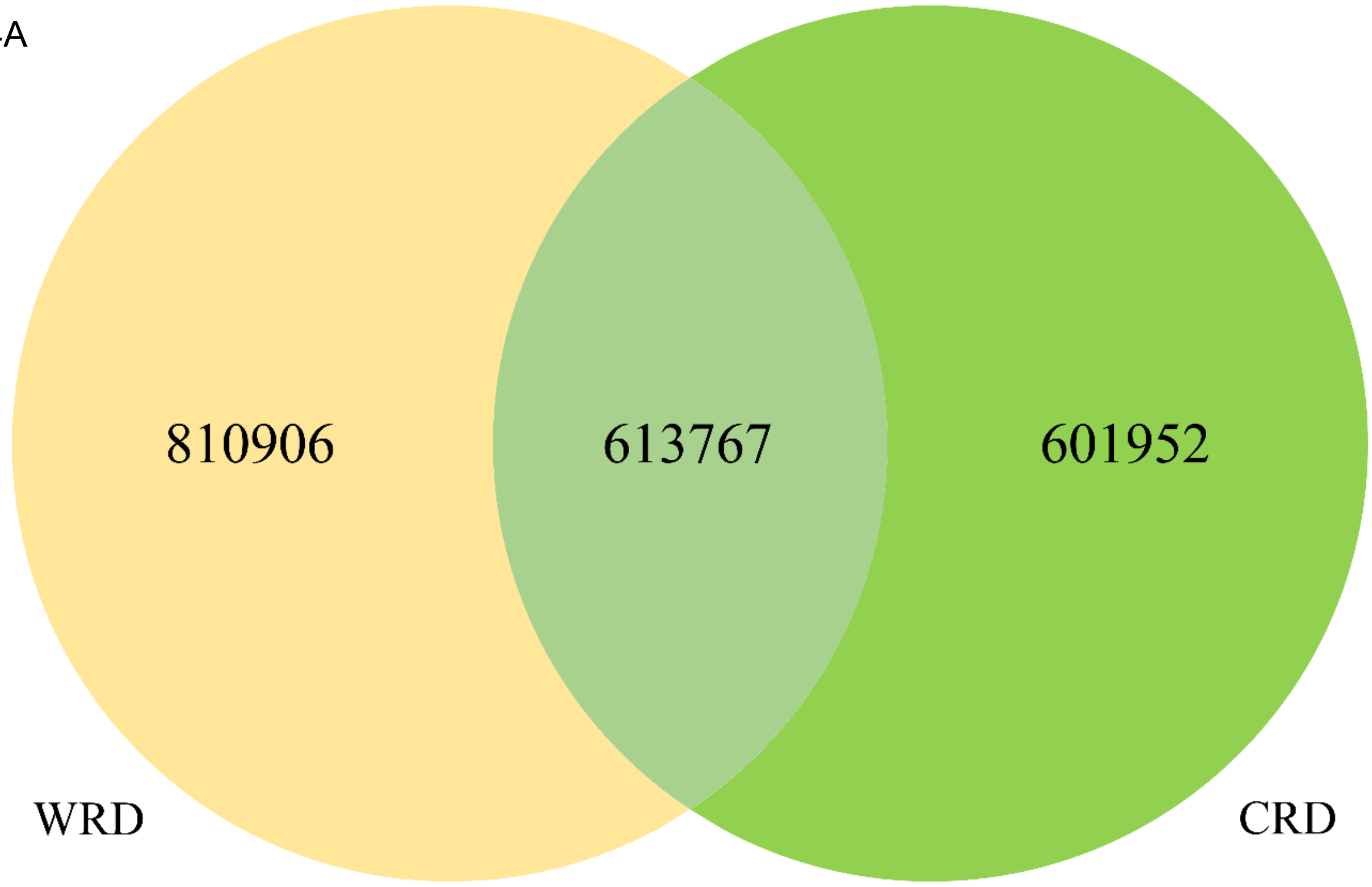

S4B

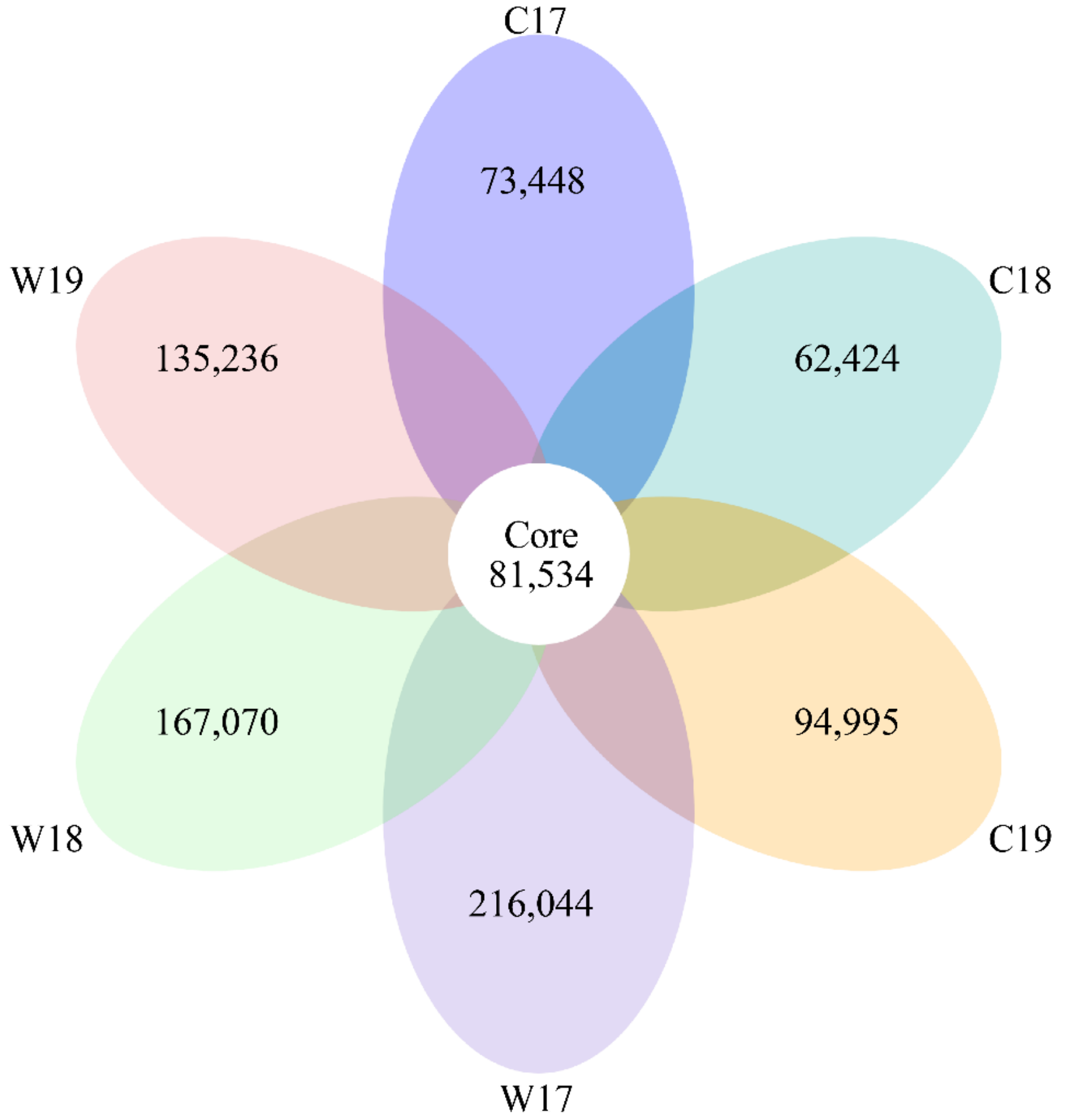

Supplemental Figure S4 Venn and flower diagrams of genes detected by metagenomic sequencing. **S4A.** Venn diagram of genes detected in captive and wild *Cervus elaphus kansuensis*. **S4B.** Flower diagram of genes detected in each sample.

Supplemental Figure S5 (A) PCoA and (B) PCA analysis of gut microbiota detected by metagenomic sequencing.

S5A

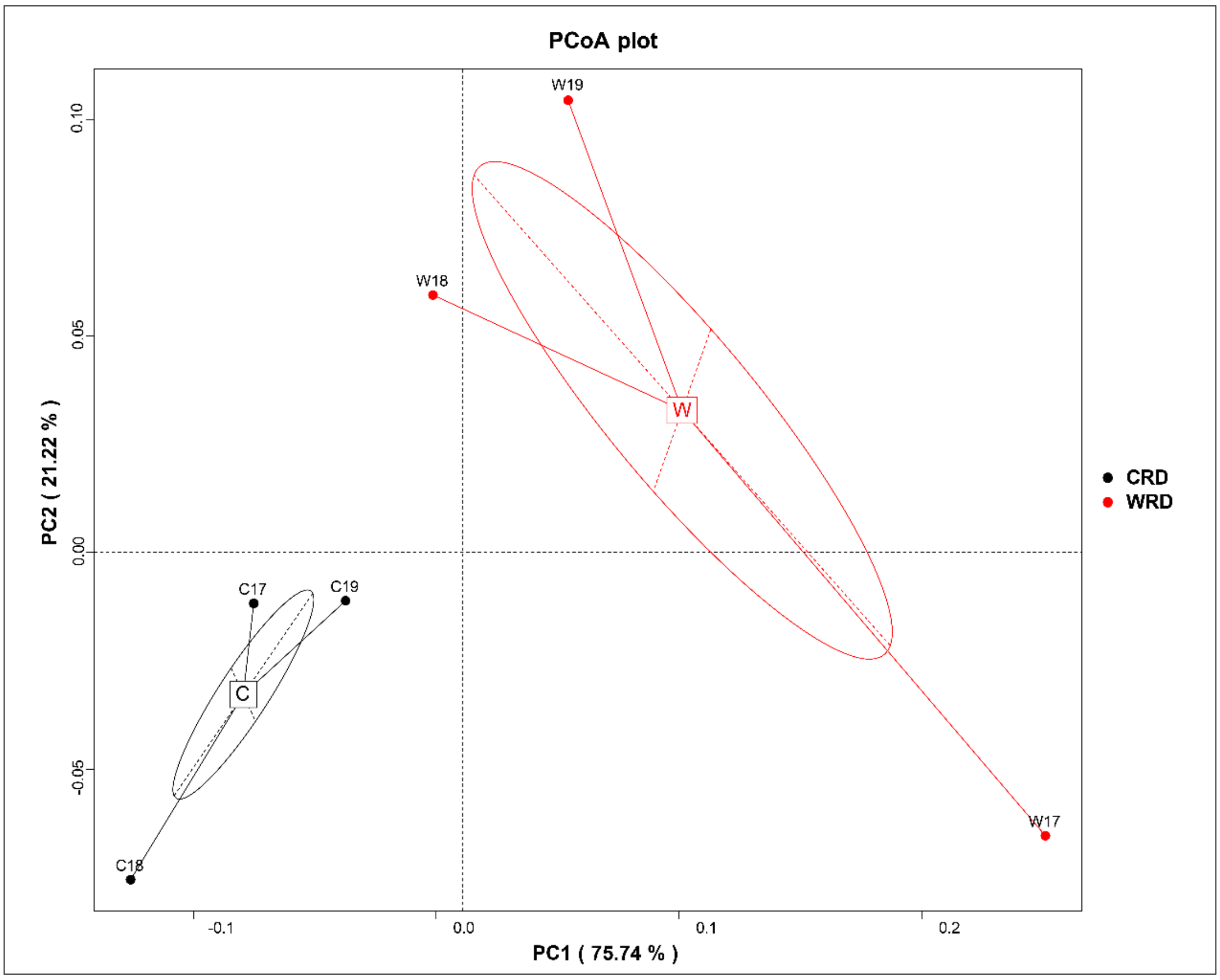

S5B

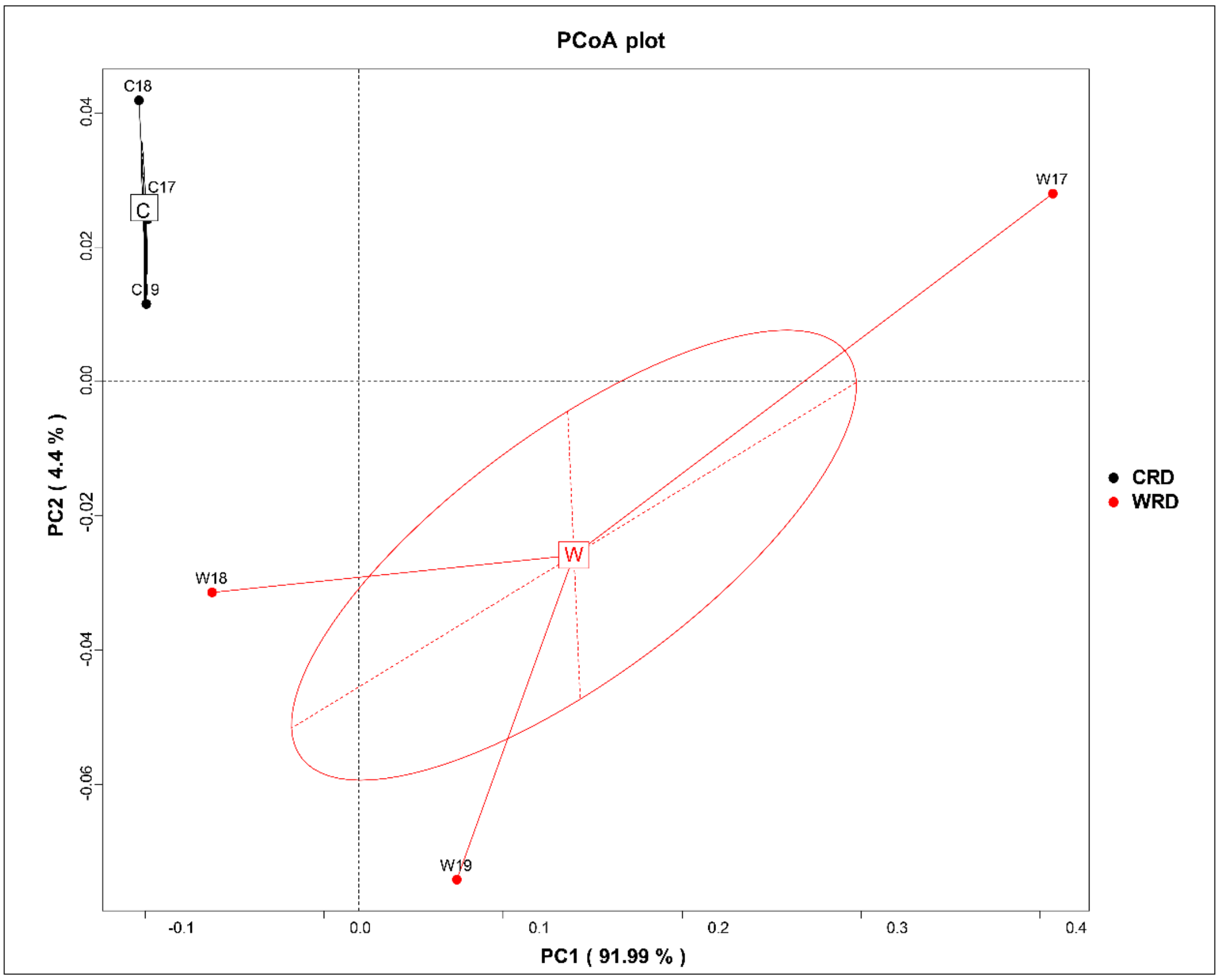
PCoA plot
C18
C17
C
C19
W17
W18
W
W19
PC2 (4.4 %)
0.04
0.02
0.00
-0.02
-0.04
-0.06
-0.1
0.0
0.1
0.2
0.3
0.4
PC1 (91.99 %)
CRD
WRD

S5C

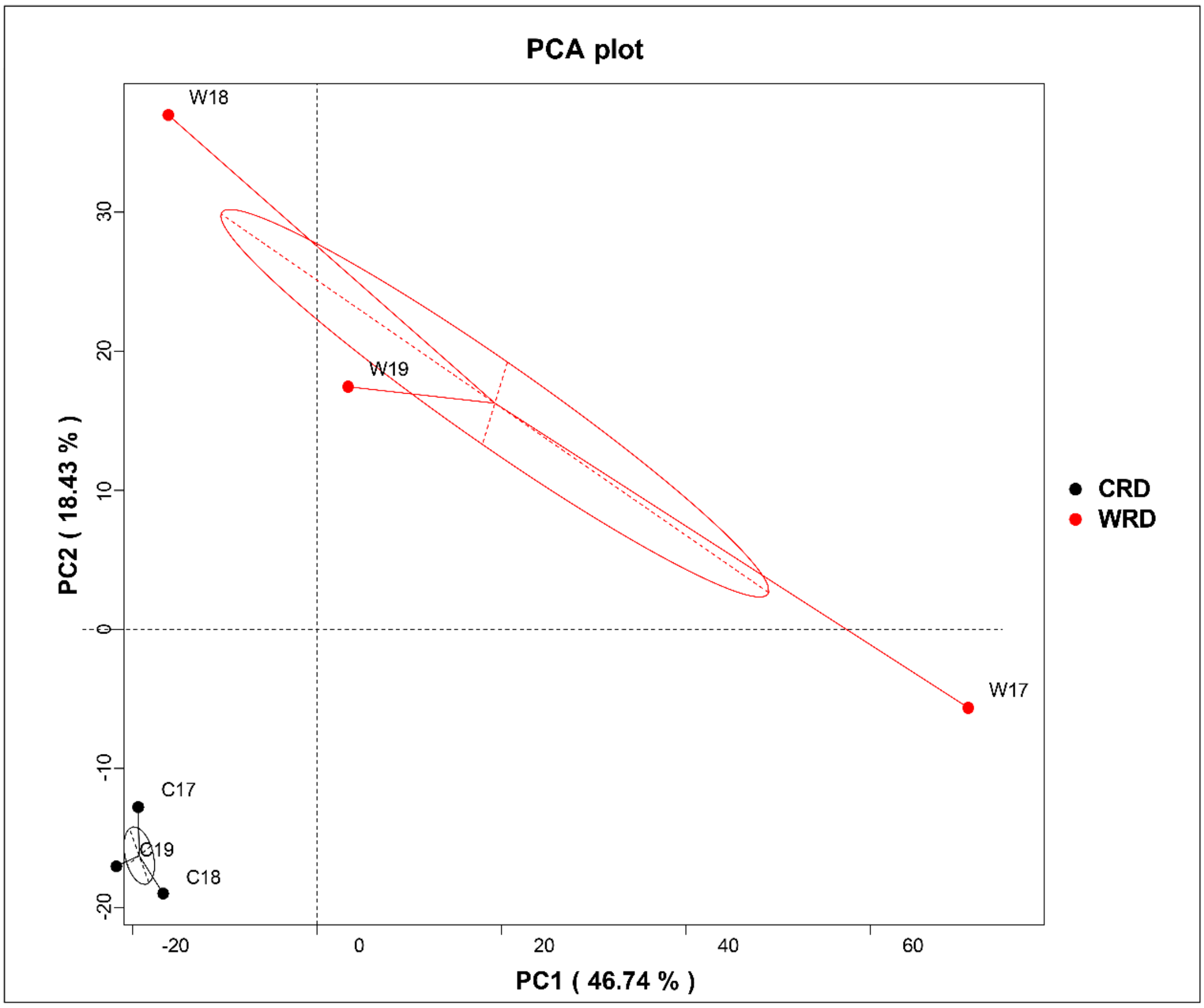
PCA plot
W18
W19
W17
C17
C19
C18
PC2 (18.43 %)
30
20
10
0
-10
-20
-20
0
20
40
60
PC1 (46.74 %)
CRD
WRD

Supplemental Figure S5 PCoA and PCA analysis of gut microbiota detected by metagenomic sequencing. **S5A.** PCoA analysis of gut microbiota at phylum level. **S5B.** PCoA analysis of gut microbiota at genus level. **S5C.** PCA analysis of gut microbiota detected by metagenomic sequencing. The results of PCoA analysis (**S5A, S5B**) showed that the microbial community structure of samples within the CRD and WRD groups was relatively similar, but the microbial community structure of the two groups was significantly different, and the samples of the two groups could be clearly clustered into two clusters at the PC1 level. The microbial community structure of samples in CRD group was relatively similar, while it in WRD group was relatively different. Figure **S5C** showed similar results to figure **S5A, S5B**, and CRD and WRD groups can be clearly distinguished at the PC2 level, with samples within the CRD group being closer together, while samples within the WRD group being farther apart. PCoA and PCA analysis showed that the gut microbial composition of captive red deer is more similar, whereas it varies more among different individuals of wild red deer.

Supplemental Figure S6 PCA and differential analysis of fecal metabolites detected in captive and wild *Cervus elaphus kansuensis* based on LC-MS/MS.

S6A

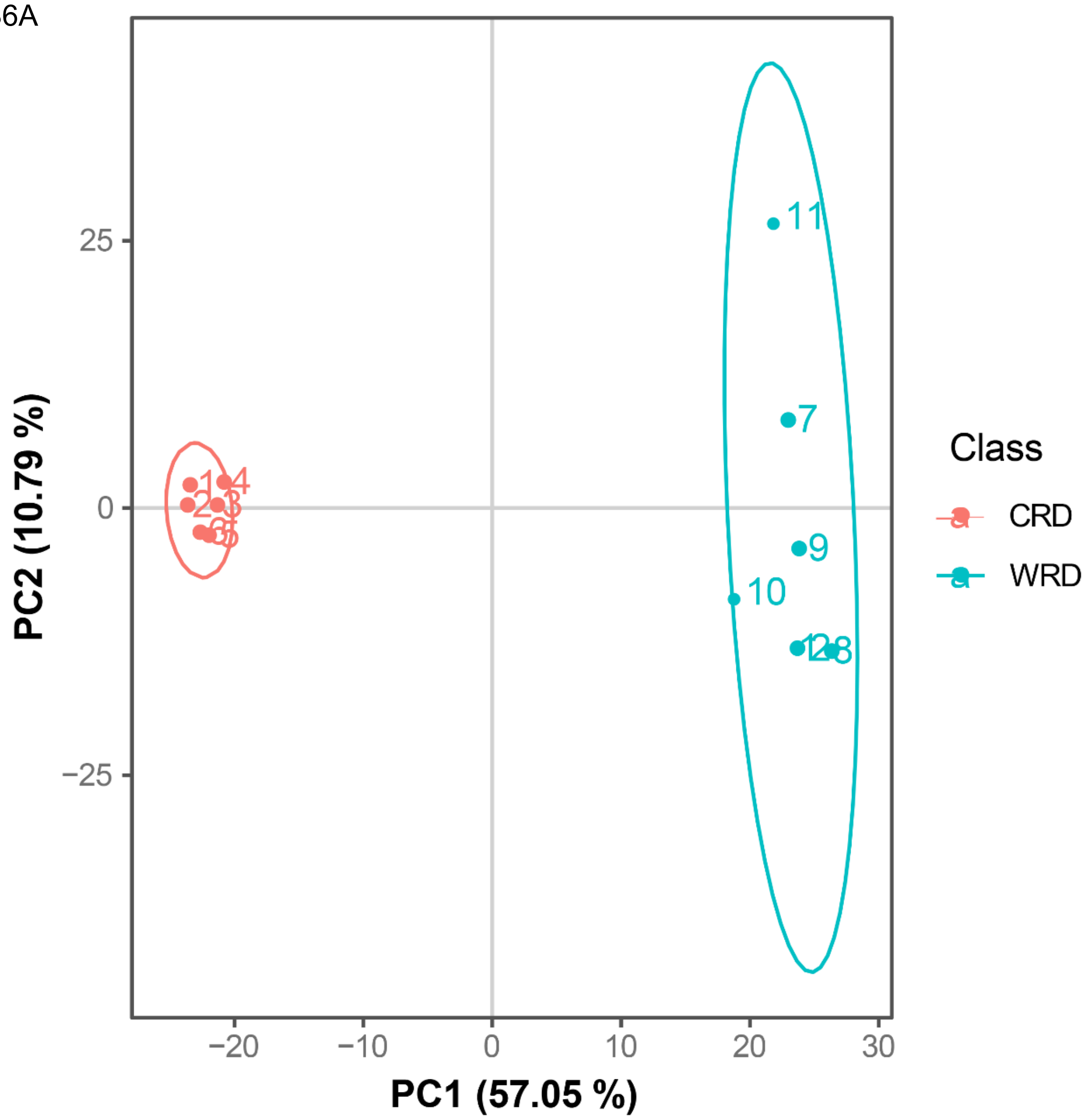

S6B

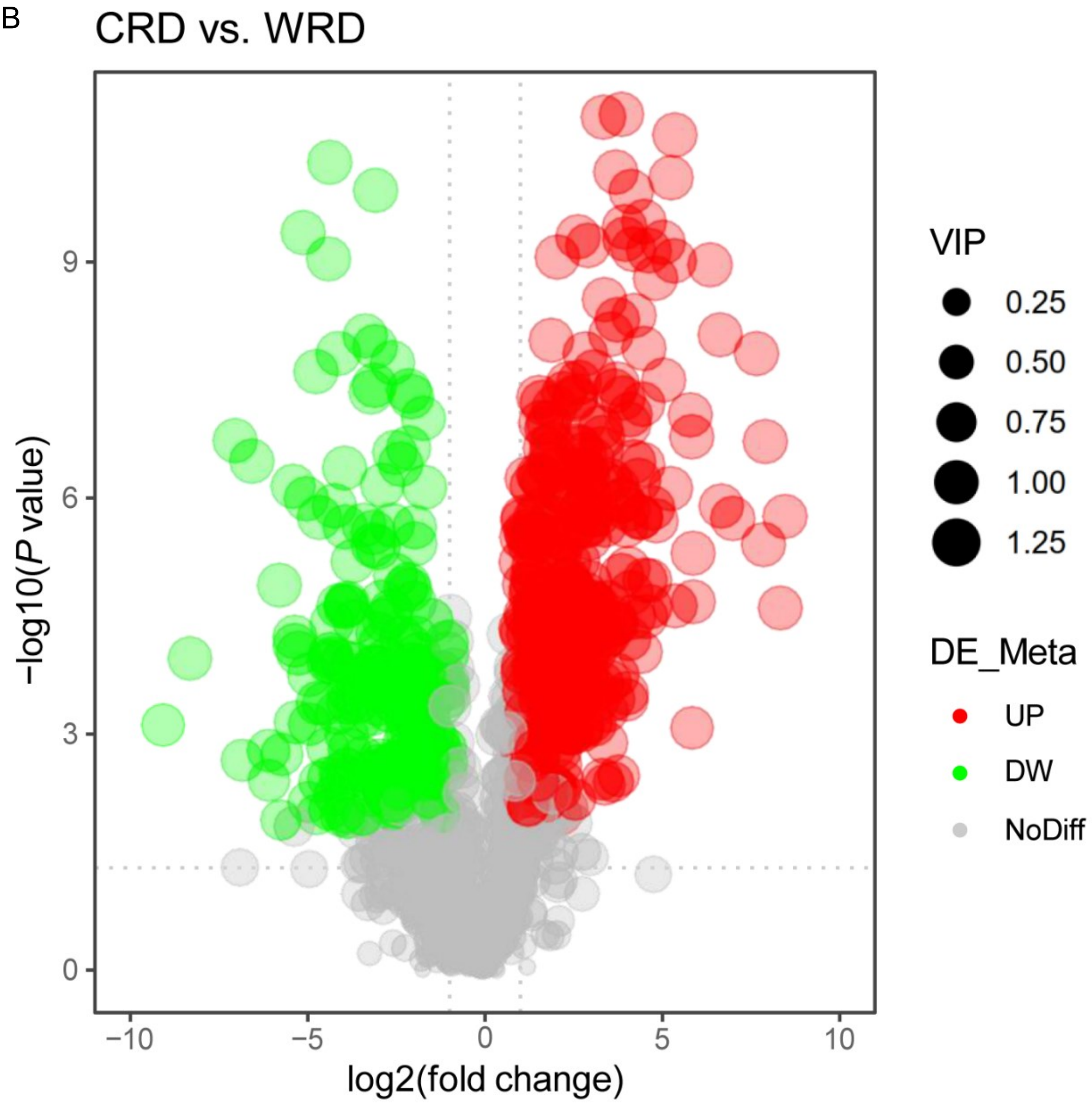
CRD vs. WRD
−log10(P value)
0
3
6
9
−10
−5
0
5
10
log2(fold change)
VIP
0.25
0.50
0.75
1.00
1.25
DE_Meta
UP
DW
NoDiff

S6C

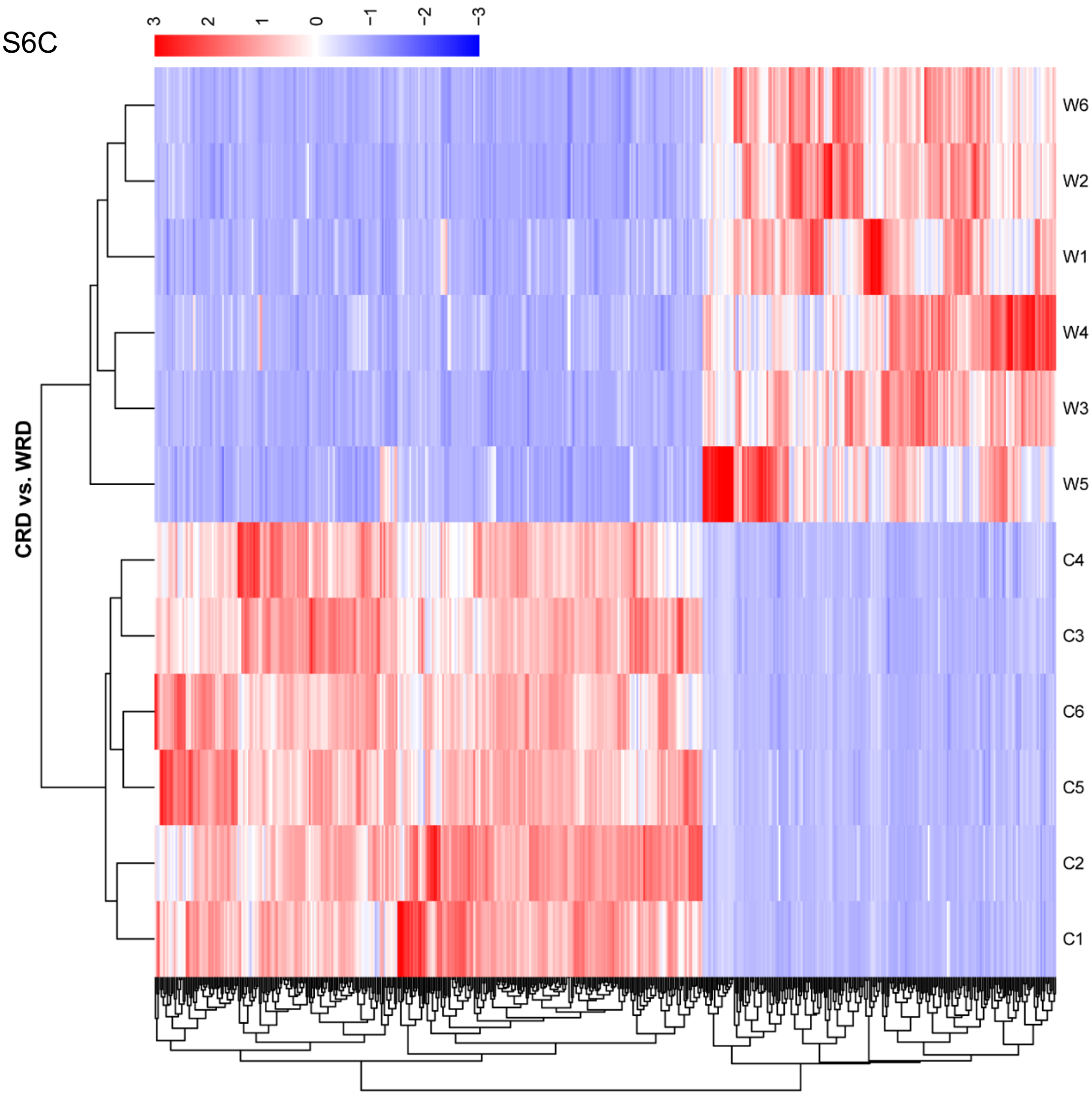

Supplemental Figure S6 PCA and differential analysis of fecal metabolites detected in captive and wild *Cervus elaphus kansuensis* based on LC-MS/MS. **S6A.** PCA analysis of gut microbiota detected by LC-MS/MS. PCA method was used to analyze the difference of fecal metabolites between the two groups. The results showed that the CRD and WRD groups were significantly clustered into two clusters, and the fecal metabolites of the two groups could be distinguished at the PC1 level. **S6B.** Volcano plot of differential metabolites. There were 549 differential metabolites were screened between CRD and WRD. Among them, 336 differential metabolites were significantly

upregulated in CRD, while 213 differential metabolites were significantly downregulated in CRD compared with WRD. **S6C.** Heat map of differential metabolites. Hierarchical clustering analysis of differential metabolites showed that the expression patterns of metabolites in the samples within the CRD and WRD groups differed less, whereas the expression patterns of metabolites between the two groups were more different.

Supplemental Figure S7 Annotation of differential fecal metabolites detected in captive and wild *Cervus elaphus kansuensis*. **S7A.** HMDB annotation of differential metabolites. **S7B**. Lipdmaps annotation of differential metabolites.

S7A

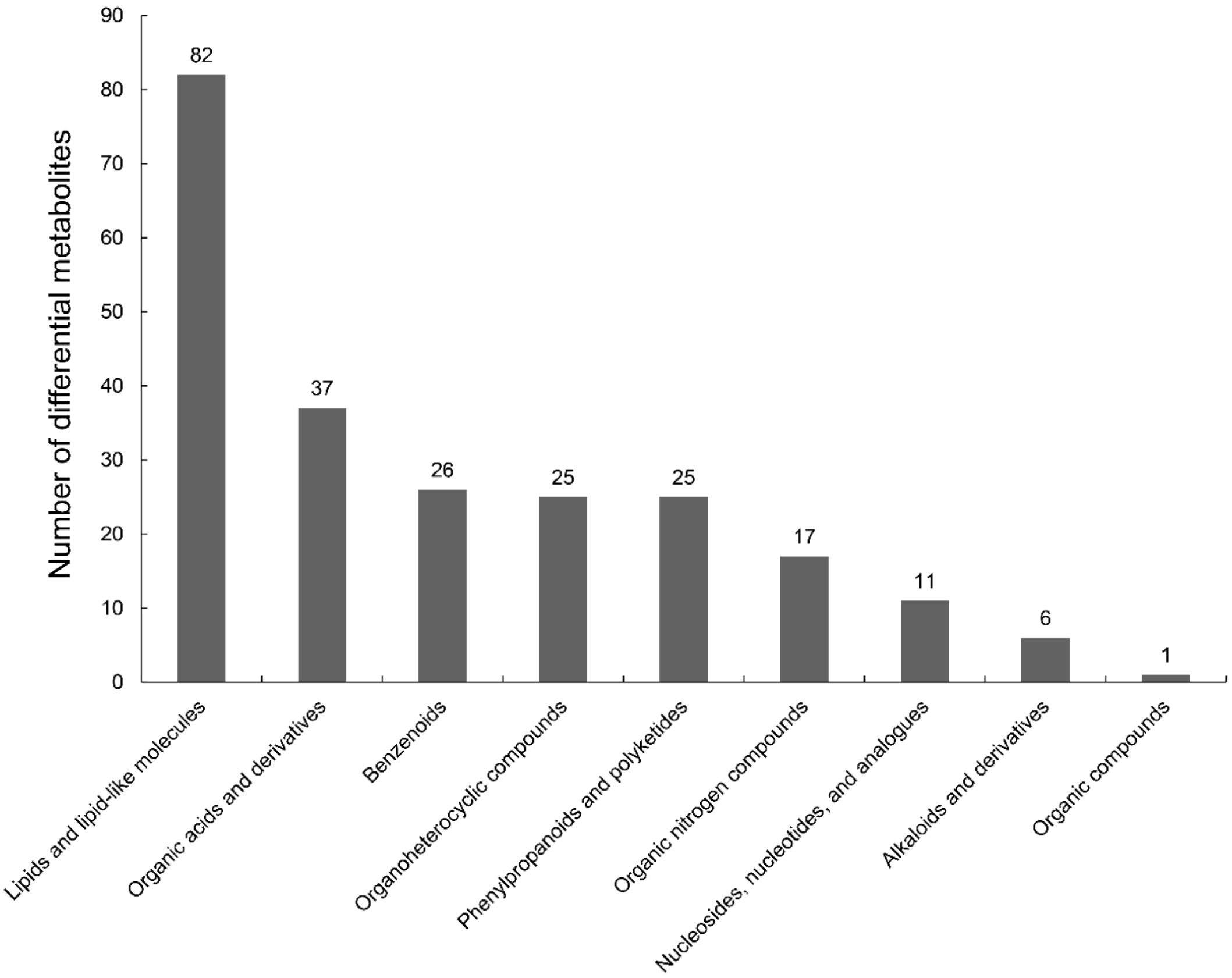

S7B

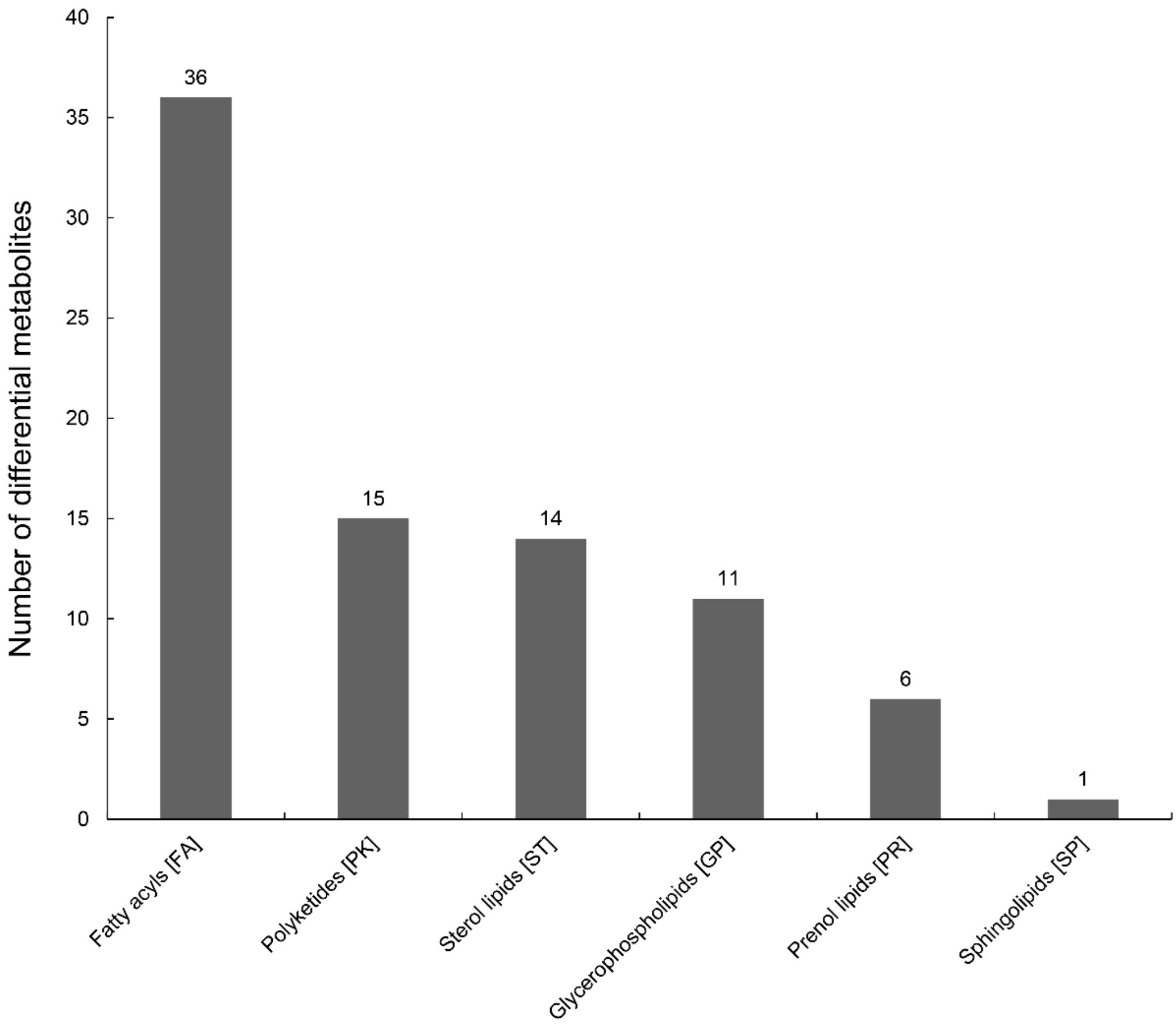

Supplemental Figure S7 Annotationofdifferentialfecalmetabolitesdetectedin captive and wild *Cervus elaphus kansuensis*. **S7A.** HMDB annotation of differential metabolites. Most differential metabolites were annotated to "Lipids and lipid-like molecules". **S7B.** Lipdmaps annotation of differential metabolites. Most differential metabolites were annotated to "Fatty acyls [FA]".
